# Supplementary material for: Engagement in meaningful activities post suicide loss: A scoping review
Source: PLoS One. 2025 Nov 17;20(11):e0336640. doi: 10.1371/journal.pone.0336640 (PMC12622850; doi:10.1371/journal.pone.0336640)
Supplement: S1 Appendix — (PDF) [file pone.0336640.s001.pdf]

S1 Appendix

Search strategy for MEDLINE via Ovid

1) Suicide/ or suicide, completed/
2) (kill* adj1 (onesel* or one-sel*)).ti,ab,kf.
3) (kill* adj1 themsel*).ti,ab,kf.
4) suicid*.ti,ab,kf.
5) 1 or 2 or 3 or 4
6) exp Bereavement/
7) (bereav* or mourn* or grie* or (loss* adj4 suicid*) or (expos* adj4 suicid*)).ti,ab,kf.
8) 6 or 7
9) 5 and 8
10) limit 9 to (english language and yr="2013-Current")
