# Supplementary material for: Engagement in meaningful activities post suicide loss: A scoping review
Source: PLoS One. 2025 Nov 17;20(11):e0336640. doi: 10.1371/journal.pone.0336640 (PMC12622850; doi:10.1371/journal.pone.0336640)
Supplement: S2 Appendix — (PDF) [file pone.0336640.s002.pdf]

S2 Appendix
Data Extraction Table

Authors	Publication Year, Country/Countries Data was Collected In, Type of Evidence, Methodology, Study Design, Sample Size of Bereaved Individuals, Recruited Age Range, Sex, Gender, Time Since Loss Range or Mean, Relationship with Lost One(s)	Aim of Study (verbatim)	Specific Activities of Everyday Living Discussed	Activities of Everyday Living Discussed	Engagement Status Discussed	Meanings Associated Discussed	Reference
Adams et al.	**Publication Year:** 2019<br>**Country/Countries Data was Collected In:** Australia<br>**Methodology:** Qualitative<br>**Study Design:** Interpretive Phenomenological Design<br>**Sample Size of Bereaved Individuals:** 7<br>**Recruited Age Range:** 20-27<br>**Sex:** 57% female<br>**Gender:** Unspecified<br>**Time Since Loss (Mean):** 3.75 years<br>**Relationship with Lost One(s):** Sibling	"This study sought to gain an understanding of the grief experiences of young adult siblings who had lost a young sibling to suicide, and the influence of family communications and relationships on those experiences." (p.329)	Caregiving and Parenting<br>Internet Sites and Social Media<br>Joining Advocacy Groups or Organizations<br>Long-Term Support Groups and Professional Support<br>Mental Health Advocacy Activities<br>Reading (general)<br>Religious Activities<br>School or Studies<br>Taking on Roles of Family Members, Change in Family Life<br>Work<br>Writing	Leisure<br>Productivity<br>Self-Care	Change Freq./Method of Engag.<br>Disengagement<br>New Engagement	Activities to Survive Loss<br>Activities to Manage and Process Loss<br>Activities to Move Forward	Adams, E., Hawgood, J., Bundock, A., & Kõlves, K. (2018). A phenomenological study of siblings bereaved by suicide: A shared experience. *Death studies, 43*(5), 324–332. https://doi.org/10.1080/07481187.2018.1469055
Adshead & Runacvres	**Publication Year:** 2022<br>**Country/Countries Data was Collected In:** England<br>**Methodology:** Qualitative<br>**Study Design:** Realist Evaluation Framework<br>**Sample Size of Bereaved Individuals:** 6<br>**Recruited Age Range:** Unspecified<br>**Sex:** 50% female<br>**Gender:** Unspecified<br>**Time Since Loss (Range or Mean):** Unspecified<br>**Relationship with Lost One(s):** Unspecified	"To understand the experiences of individuals attending a suicide bereavement social support group, and to explore the perceived impact of this on wellbeing" (p.3)	Long-Term Support Groups and Professional Support<br>Social Activities (General)<br>Writing (Journaling, Letter Writing)	Leisure<br>Self-Care	New Engagement	Activities to Manage and Process Loss	Adshead, C., & Runacres, J. (2022). Sharing is Caring: A Realist Evaluation of a Social Support Group for Individuals Who Have Been Bereaved by Suicide. *Omega, 89*(1), 172–190. https://doi.org/10.1177/00302228211070152
Ali, F. & Lucock	**Publication Year:** 2020<br>**Country/Countries Data was Collected In:** United Kingdom<br>**Methodology:** Qualitative<br>**Study Design:** Constructivist Grounded Theory<br>**Sample Size of Bereaved Individuals:** 24<br>**Recruited Age Range:** 23-67<br>**Sex:** 27% female<br>**Gender:** Unspecified<br>**Time Since Loss (Range or Mean):** 0.25-19 years<br>**Relationship with Lost One(s):** Child, Extended Family Member, Parent, Partner/Spouse, Sibling	"This study focussed on understanding the impact of suicide on families of people who were in receipt of mental health services in order to understand how this prior involvement influences how the bereaved person makes sense of the suicide, its impact on them and their willingness to seek support for themselves." (p.5)	Activities related to creation of a new life<br>Enjoyed Activities, Hobbies (General)<br>Long-Term Support Groups and Professional Support<br>Reading (To Heal)<br>Routines and Rituals Related to Lost one	Leisure<br>Self-Care	Change Freq./Method of Engag.<br>New Engagement	Activities to Manage and Process Loss<br>Activities to Move Forward	Ali, F., & Lucock, M. (2020). 'It's like getting a group hug and you can cry there and be yourself and they understand'. Family members' experiences of using a suicide bereavement peer support group. *Bereavement Care, 39*(2), 51–58. https://doi-org.myaccess.library.utoronto.ca/10.1080/02682621.2020.1771951
Ali, U. & Rehna	**Publication Year:** 2022<br>**Country/Countries Data was Collected In:** Pakistan<br>**Methodology:** Qualitative<br>**Study Design:** Interpretative Phenomenological Design<br>**Sample Size of Bereaved Individuals:** 6<br>**Recruited Age Range:** Unspecified<br>**Sex:** 50% female<br>**Gender:** Unspecified<br>**Time Since Loss (Range or Mean):** 2-7 years<br>**Relationship with Lost One(s):** Child	"This study explored lived experiences of suicide-bereaved parents and the stigma attached to a suicide death." (p.1)	Everyday Activities (General)<br>Religious Activities<br>Sleep	Self-Care	Change Freq./Method of Engag.<br>Disengagement	Activities to Survive Loss<br>Activities to Manage and Process Loss	Ali, U., & Rehna, T. (2023). Grief Reactions and Suicide Bereavement in the Context of Stigma among Parents: An Interpretative Phenomenological Analysis. *Annales Médico-Psychologiques, 181*(7), 598–603. https://doi.org/10.1016/j.amp.2022.04.016
Azorina et al.	**Publication Year:** 2019<br>**Country/Countries Data was Collected In:** United Kingdom<br>**Methodology:** Qualitative<br>**Study Design:** Qualitative Cross-sectional Study Design and Online Survey<br>**Sample Size of Bereaved Individuals:** 499<br>**Recruited Age Range:** 18-40<br>**Sex:** 83% female<br>**Gender:** Unspecified<br>**Time Since Loss (Range or Mean):** 0.0385-26 years<br>**Relationship with Lost One(s):** Blood relative, Colleague/Co-Worker, Children in law, Ex-Partner, Friend, Partner/Spouse, Step-Children	"Our objective was to explore the views of young adults bereaved by suicide on any changes in their relationships with family and friends since the bereavement, and the nature and quality of informal support available after suicide bereavement." (p.2)	Caregiving and Parenting<br>Social Activities (General)	Leisure<br>Productivity	Change Freq./Method of Engag.<br>Disengagement	Activities to Survive Loss<br>Activities to Manage and Process Loss	Azorina, V., Morant, N., Nesse, H., Stevenson, F., Osborn, D., King, M., & Pitman, A. (2019). The Perceived Impact of Suicide Bereavement on Specific Interpersonal Relationships: A Qualitative Study of Survey Data. *International journal of environmental research and public health, 16*(10), 1801. https://doi.org/10.3390/ijerph16101801
Bailey E et al.	**Publication Year:** 2017<br>**Country/Countries Data was Collected In:** United States, United Kingdom, Australia, Canada<br>**Methodology:** Quantitative<br>**Study Design:** Quantitative Cross-sectional Study Design and Online Survey<br>**Sample Size of Bereaved Individuals:** 222<br>**Recruited Age Range:** 17-81<br>**Sex:** 94.60% female<br>**Gender:** Unspecified<br>**Time Since Loss (Range or Mean):** 0.5-5 years<br>**Relationship with Lost One(s):** Aquaintance, Child, Colleague/Co-Worker, Extended Family Member, Friend, Parent, Partner/Spouse, Sibling, Other	"The aim of this study was to survey users of Internet forums and Facebook groups for the bereaved by suicide to answer the following questions:<br>1. How are Internet forums used by those bereaved by suicide?<br>2. Do those who use suicide bereavement forums seek help elsewhere?<br>3. What are the perceived benefits and limitations of this use?" (p.2)	Internet Sites and Social Media	Leisure	New Engagement		Bailey, E., Krysinska, K., O'Dea, B., & Robinson, J. (2017). Internet Forums for Suicide Bereavement. *Crisis, 38*(6), 393–402. https://doi.org/10.1027/0227-5910/a000471

Authors	Publication Year, Country/Countries Data was Collected In, Type of Evidence, Methodology, Study Design, Sample Size of Bereaved Individuals, Recruited Age Range, Sex, Gender, Time Since Loss Range or Mean, Relationship with Lost One(s)	Aim of Study (verbatim)	Specific Activities of Everyday Living Discussed	Activities of Everyday Living Discussed	Engagement Status Discussed	Meanings Associated Discussed	Reference
Bailey L et al.	**Publication Year:** 2015<br>**Country/Countries Data was Collected In:** United Kingdom<br>**Methodology:** Qualitative<br>**Study Design:** Unspecified Qualitative: Semi structured interviews<br>**Sample Size of Bereaved Individuals:** 11<br>**Recruited Age Range:** 30-60<br>**Sex:** 73% female<br>**Gender:** Unspecified<br>**Time Since Loss (Range or Mean):** 0.42-4 years<br>**Relationship with Lost One(s):** Child, Friend, Sibling	"This paper presents interim findings from ongoing research which focuses on two aspects of suicide memorial websites. First, we explore the extent to which such sites help us understand how the Internet is enabling new ways of grieving and is, in effect, making new cultural scripts. Second, although there is a large body of writing on the management of trauma there is little evidence-based research. The paper draws on face-to-face interviews with owners of suicide memorial sites (family members and friends) and explores how the establishment and maintenance of such a site is an important part of the therapeutic process and how, for grieving relatives, making or contributing to such sites provides ways of managing trauma in the aftermath of a death by suicide." (p.1)	Internet Sites and Social Media<br>Joining Advocacy Groups or Organizations	Leisure<br>Productivity	New Engagement	Activities to Manage and Process Loss<br>Activities to Move Forward	Bailey, L., Bell, J., & Kennedy, D. (2015). Continuing social presence of the dead: exploring suicide bereavement through online memorialisation. *New Review of Hypermedia and Multimedia, 21*(1–2), 72–86. https://doi-org.myaccess.library.utoronto.ca/10.1080/13614568.2014.983554
Bartik et al.	**Publication Year:** 2020<br>**Country/Countries Data was Collected In:** Australia<br>**Methodology:** Mixed Methods<br>**Study Design:** Unspecified Mixed Methods<br>**Sample Size of Bereaved Individuals:** 18<br>**Recruited Age Range:** 12-23<br>**Sex:** 61% female<br>**Gender:** Unspecified<br>**Time Since Loss (Range or Mean):** Unspecified<br>**Relationship with Lost One(s):** Friend	"The aim of this study was to better understand the impact of bereavement on rural young people who lose a friend to suicide." (p.484)	Alcohol and Drug Use, Risky and Sexual Behaviours	Self-Care	Change Freq./Method of Engag.	Activities to Survive Loss	Bartik, W. J., Maple, M., & McKay, K. (2020). Youth Suicide Bereavement and the Continuum of Risk. *Crisis: The Journal of Crisis Intervention and Suicide Prevention, 41*(6), 483–489. https://doi.org/10.1027/0227-5910/a000653
Bartik et al.	**Publication Year:** 2013<br>**Country/Countries Data was Collected In:** Australia<br>**Methodology:** Qualitative<br>**Study Design:** Qualitative Pilot Study<br>**Sample Size of Bereaved Individuals:** 10<br>**Recruited Age Range:** 18-29<br>**Sex:** 80% female<br>**Gender:** Unspecified<br>**Time Since Loss (Range or Mean):** 1-8 years<br>**Relationship with Lost One(s):** Friend	"The aim of this initial pilot study was to investigate the impact of bereavement for young people who lose a friend to suicide given the limited empirical research in this area, and to gain insight into, and understanding of, their grief experience." (p.212)	Alcohol and Drug Use, Risky and Sexual Behaviours<br>Caregiving and Parenting<br>Self-Care (General)	Productivity<br>Self-Care	Change Freq./Method of Engag.<br>Disengagement	Activities to Survive Loss<br>Activities to Manage and Process Loss	Bartik, W., Maple, M., Edwards, H., & Kiernan, M. (2013). Adolescent survivors after suicide: Australian young people's bereavement narratives. *Crisis: The Journal of Crisis Intervention and Suicide Prevention, 34*(3), 211-217. doi:https://doi-org.myaccess.library.utoronto.ca/10.1027/0227-5910/a000185
Bell et al.	**Publication Year:** 2015<br>**Country/Countries Data was Collected In:** United Kingdom<br>**Methodology:** Qualitative<br>**Study Design:** Qualitative Interpretative Approach<br>**Sample Size of Bereaved Individuals:** 11<br>**Recruited Age Range:** 20-60<br>**Sex:** 73% female<br>**Gender:** Unspecified<br>**Time Since Loss (Range or Mean):** 0.42-4 years<br>**Relationship with Lost One(s):** Child, Friend, Sibling	"this research focuses on better understanding of how digital memorialisation on Facebook in particular impacts surviving friends and family members in the aftermath of a suicide" (p.375)	Joining Advocacy Groups or Organizations<br>Internet Sites and Social Media	Leisure<br>Productivity	Change Freq./Method of Engag.<br>New Engagement	Activities to Survive Loss<br>Activities to Manage and Process Loss<br>Activities to Move Forward	Bell, J., Bailey, L., & Kennedy, D. (2015). 'We do it to keep him alive': bereaved individuals' experiences of online suicide memorials and continuing bonds. *Mortality (Abingdon, England), 20*(4), 375–389. https://doi.org/10.1080/13576275.2015.1083693
Bjornsdottir et al.	**Publication Year:** 2024<br>**Country/Countries Data was Collected In:** Iceland<br>**Methodology:** Qualitative<br>**Study Design:** Phenomenological Study<br>**Sample Size of Bereaved Individuals:** 10<br>**Recruited Age Range:** 40 - 65<br>**Sex:** 70% female<br>**Gender:** Identified as mothers (70%) and fathers (30%)<br>**Time Since Loss (Range or Mean):** 3 - 24 years<br>**Relationship with Lost One(s):** Child	The study aimed to explore parents' experience of a son's or daughter's suicide, the prelude, effects on the parents, as well as the subsequent grief processing and services available to them. The main research question was: What is the essence of parents' experience of a son's or daughter's suicide? The sub-questions were: Was there a prelude? What was their reaction and grieving process, and what was their experience of the services available? (p.975)	Eating<br>Mental Health Advocacy Activities<br>Sleep	Productivity<br>Self-Care	Change Freq./Method of Engag.<br>New Engagement	Activities to Survive Loss<br>Activities to Move Forward	Bjornsdottir, E. A., Sigurdardottir, S., & Halldorsdottir, S. (2024). Excruciating existential suffering and complicated grief: The essence of surviving the suicide of a son or daughter. *Scandinavian Journal of Caring Sciences, 38*(4), 973–983. https://doi.org/10.1111/scs.13289
Black & Heo	**Publication Year:** 2023<br>**Country/Countries Data was Collected In:** United States<br>**Methodology:** Qualitative<br>**Study Design:** Interpretative Phenomenological Analysis<br>**Sample Size of Bereaved Individuals:** 25<br>**Recruited Age Range:** 35 - 78<br>**Sex:** 100% female<br>**Gender:** Identified as mothers (100%)<br>**Time Since Loss (Range or Mean):** 1 - 15 years<br>**Relationship with Lost One(s):** Child	The aim of this interpretative phenomenological (IPA) study was to explore the lived experiences of parents whose child(ren) died by suicide, including parental experiences of suicide, the process of grieving, and the process of making meaning of the death. The research question for this study was "what are the lived experiences of parents whose child(ren) died by suicide?" (p.4)	Everyday Activities (General)<br>Mental Health Advocacy Activities<br>Work	Productivity	Change Freq./Method of Engag.<br>Disengagement<br>New engagement	Activities to Survive Loss<br>Activities to Move Forward	Black, V., & Heo, S. (2023). When a Child Dies by Suicide: An Interpretative Phenomenological Analysis Study. *Omega*, 302228231194213. Advance online publication. https://doi.org/10.1177/00302228231194213

Authors	Publication Year, Country/Countries Data was Collected In, Type of Evidence, Methodology, Study Design, Sample Size of Bereaved Individuals, Recruited Age Range, Sex, Gender, Time Since Loss Range or Mean, Relationship with Lost One(s)	Aim of Study (verbatim)	Specific Activities of Everyday Living Discussed	Activities of Everyday Living Discussed	Engagement Status Discussed	Meanings Associated Discussed	Reference
Blaze & Roberts	**Publication Year:** 2023<br>**Country/Countries Data was Collected In:** Australia<br>**Methodology:** Qualitative<br>**Study Design:** Thematic Analysis<br>**Sample Size of Bereaved Individuals:** 10<br>**Recruited Age Range:** 21 - 49<br>**Sex:** 90% female<br>**Gender:** Unspecified<br>**Time Since Loss (Range or Mean):** 1 - 14 years (mean: 4 years)<br>**Relationship with Lost One(s):** Sibling	This study aimed to gain an understanding of the experiences of siblings bereaved through suicide, and the support they found helpful in their journey. (p.12)	Activities to connect with lost one (hobbies enjoyed by lost one)<br>Caregiving and Parenting<br>Long-term Support Groups and Professional Support<br>Work	Leisure<br>Productivity<br>Self-Care	New Engagement<br>Re-engagement	Activities to Manage and Process Loss<br>Activities to Move Forward	Blaze, P., & Roberts, R. M. (2023). Support After Suicide: A Thematic Analysis of Siblings' Experience. *Omega*, 302228231195922. Advance online publication. https://doi.org/10.1177/00302228231195922
Bottomley et al.	**Publication Year:** 2019<br>**Country/Countries Data was Collected In:** United States<br>**Methodology:** Qualitative<br>**Study Design:** Consensual Qualitative Research Methods<br>**Sample Size of Bereaved Individuals:** 8<br>**Recruited Age Range:** 24-67<br>**Sex:** 100% female<br>**Gender:** Identified as women (90%) and men (10%)<br>**Time Since Loss (Range or Mean):** 0.58-17.83 years<br>**Relationship with Lost One(s):** Child, Friend, Parent, Sibling	First, we attempted to use the MLC (meaning loss codebook) to illuminate the meaning making efforts of one group of survivors who typically face quite daunting challenges to making sense of the loss, namely those bereaved by the suicide of a loved one. Second, by performing an analysis of in-depth interviews with recent as well as long-term survivors, we sought to determine whether additional codes might be added to the MLC to provide a fuller depiction of their quest for meaning over time,	Long-Term Support Groups and Professional Support<br>Peer Group Facilitation<br>Research Participation<br>Routines and Rituals Related to Lost one	Productivity<br>Self-Care	New Engagement	Activities to Move Forward	Bottomley, J. S., Smigelsky, M. A., Bellet, B. W., Flynn, L., Price, J., & Neimeyer, R. A. (2019). Distinguishing the meaning making processes of survivors of suicide loss: An expansion of the meaning of loss codebook. *Death Studies, 43*(2), 92–102. https://doi.org/10.1080/07481187.2018.1456011
Causer et al.	**Publication Year:** 2024<br>**Country/Countries Data was Collected In:** United Kingdom<br>**Methodology:** Qualitative<br>**Study Design:** Unspecified Qualitative: Semi-structured, Grounded Theory<br>**Sample Size of Bereaved Individuals:** 29<br>**Recruited Age Range:** 25 - 61<br>**Sex:** 83% female<br>**Gender:** Unspecified<br>**Time Since Loss (Range or Mean):** <1 - 12 years (mean: 2.5 years)<br>**Relationship with Lost One(s):** Colleague	We asked, "how is the impact of colleague suicide experienced by NHS staff and what helps and/or hinders affected colleagues in seeking support?" (p.2)	Long-term Support Groups and Professional Support<br>Routines and Rituals Related to Lost one<br>Social Activities (General)<br>Work	Leisure<br>Productivity<br>Self-Care	Change Freq./Method of Engag.<br>Disengagement<br>New engagement	Activities to Survive Loss<br>Activities to Manage and Process Loss<br>Activities to Move Forward	Causer, H., Spiers, J., Chew-Graham, C. A., Efstathiou, N., Gopfert, A., Grayling, K., Maben, J., van Hove, M., Riley, R. (2024). Filling in the gaps: A grounded theory of the experiences and needs of healthcare staff following a colleague death by suicide in the UK. *Death Studies*, 1–12. https://doi.org/10.1080/07481187.2024.2337202
Čepulienė et al.	**Publication Year:** 2022<br>**Country/Countries Data was Collected In:** Lithuania<br>**Methodology:** Qualitative<br>**Study Design:** Unspecified Qualitative: Semi structured Interviews<br>**Sample Size of Bereaved Individuals:** 11<br>**Recruited Age Range:** 28-62<br>**Sex:** 100% female<br>**Gender:** Identified as women (100%)<br>**Time Since Loss (Range or Mean):** 2-5 years<br>**Relationship with Lost One(s):** Partner/Spouse	"the current study aimed to qualitatively explore the role of spirituality during suicide bereavement." (p.3)	Religious Activities<br>Research Participation<br>Routines and Rituals Related to Lost one	Leisure<br>Productivity<br>Self-Care	Change Freq./Method of Engag.<br>Continued Engagement<br>New Engagement	Activities to Manage and Process Loss<br>Activities to Move Forward	Čepulienė, A. A. & Skruibis, P. (2022). The Role of Spirituality during Suicide Bereavement: A Qualitative Study. *International Journal of Environmental Research and Public Health*, *19*, 8740. https://doi.org/10.3390/ijerph19148740
Čepulienė et al.	**Publication Year:** 2023<br>**Country/Countries Data was Collected In:** Lithuania<br>**Methodology:** Qualitative<br>**Study Design:** Exploratory Study<br>**Sample Size of Bereaved Individuals:** 9<br>**Recruited Age Range:** 28 - 62<br>**Sex:** 100% female<br>**Gender:** Identified as women (100%)<br>**Time Since Loss (Range or Mean):** 2 - 5 years<br>**Relationship with Lost One(s):** Partner/Spouse	We explored the dream about the deceased in a sample of women bereaved by their partners' suicide with the aim to understand their functions. (p.1030)	Religious Activities<br>Sleep	Self-Care	Change Freq./Method of Engag.	Activities to Survive Loss<br>Activities to Manage and Process Loss	Čepulienė, A. A., & Skruibis, P. (2023). The functions of the dreams of the deceased: A qualitative study of women bereaved by partner's suicide. *Death Studies, 48*(10), 1025–1034. https://doi.org/10.1080/07481187.2023.2297063
Čepulienė et al.	**Publication Year:** 2023<br>**Country/Countries Data was Collected In:** Lithuania<br>**Methodology:** Qualitative<br>**Study Design:** Unspecified Qualitative: Semi-structured Interviews and Reflexive Thematic Analysis<br>**Sample Size of Bereaved Individuals:** 11<br>**Recruited Age Range:** 28 - 62<br>**Sex:** 100% female<br>**Gender:** Unspecified<br>**Time Since Loss (Range or Mean):** 2 - 5 years<br>**Relationship with Lost One(s):** Partner/Spouse	Our study aimed to explore how women, who are bereaved by their partner's suicide, describe spirituality. (p.1)	Gardening<br>Religious Activities<br>Yoga	Leisure<br>Self-Care	Change Freq./Method of Engag.<br>New Engagement	Activities to Survive Loss<br>Activities to Manage and Process Loss<br>Activities to Move Forward	Čepulienė, A. A., & Skruibis, P. (2023). What is spirituality for women bereaved by a partner's suicide: A qualitative study. *Cogent Psychology, 10*(1). https://doi.org/10.1080/23311908.2023.2183676

Authors	Publication Year, Country/Countries Data was Collected In, Type of Evidence, Methodology, Study Design, Sample Size of Bereaved Individuals, Recruited Age Range, Sex, Gender, Time Since Loss Range or Mean, Relationship with Lost One(s)	Aim of Study (verbatim)	Specific Activities of Everyday Living Discussed	Activities of Everyday Living Discussed	Engagement Status Discussed	Meanings Associated Discussed	Reference
Chan & Cheung	**Publication Year:** 2022<br>**Country/Countries Data was Collected In:** China<br>**Methodology:** Qualitative<br>**Study Design:** Unspecified Qualitative: Semi-structured Interviews<br>**Sample Size of Bereaved Individuals:** 10<br>**Recruited Age Range:** 30-60<br>**Sex:** 0% female<br>**Gender**: Unspecified<br>**Time Since Loss (Range or Mean):** Unspecified<br>**Relationship with Lost One(s):** Child, Partner/Spouse	"This study investigated how Chinese men communicated their sorrow and sought help." (p.1846)	Caregiving and Parenting<br>Housework, Chores<br>Social Activities (General)<br>Taking on Roles of Family Members, Change in Family Life	Leisure<br>Productivity	Change Freq./Method of Engag.<br>Disengagement<br>New Engagement	Activities to Survive Loss<br>Activities to Manage and Process Loss	Chan, T. M. S. & Cheung, M. (2022) The "men in grief" phenomenon<br>among suicide bereaved Chinese men in Hong Kong, *Death Studies*, *46*(8), 1845-1852, DOI: 10.1080/07481187.2020.1855609
Chen M et al.	**Publication Year:** 2023<br>**Country/Countries Data was Collected In:** China<br>**Methodology:** Qualitative<br>**Study Design:** Interpretative Phenomenological Analysis<br>**Sample Size of Bereaved Individuals:** 5<br>**Recruited Age Range:** Unspecified<br>**Sex:** 60% female<br>**Gender**: Unspecified<br>**Time Since Loss (Range or Mean):** Unspecified<br>**Relationship with Lost One(s):** Parent, Sibling	The aim of the study is to provide insight and facilitate a deeper understanding of family members who have experienced their older family member's suicide. (p. 4424)	Caregiving and Parenting<br>Work	Productivity	Change Freq./Method of Engag.<br>Disengagement	Activities to Survive Loss<br>Activities to Manage and Process Loss	Chen, M., Zhang, X., & McCormack, B. (2023). The lived experience of family members of older people who have died by suicide in rural China. *Nursing open*, *10*(7), 4424–4431. https://doi.org/10.1002/nop2.1684
Chen Y & Laitila	**Publication Year:** 2024<br>**Country/Countries Data was Collected In:** China<br>**Methodology:** Qualitative<br>**Study Design:** Case Study<br>**Sample Size of Bereaved Individuals:** 1<br>**Recruited Age Range:** 30 - 39<br>**Sex:** 0% female<br>**Gender:** Identified as a man (100%)<br>**Time Since Loss (Range or Mean):** 0.25 - 0.25 years<br>**Relationship with Lost One(s):** Partner/Spouse	"This study aimed to shed light on the initial-stage bereavement experiences of an individual bereaved by suicide, at three months from the loss of his spouse to suicide. (p.1)"	Reading (To Heal)<br>Work	Leisure<br>Productivity	Change Freq./Method of Engag.<br>Continued Engagement	Activities to Survive Loss	Chen, Y., & Laitila, A. (2024). Initial-Stage Suicide Bereavement Experiences: A Case Study. *OMEGA - Journal of Death and Dying*, *89*(4), 1514-1534. https://doi.org/10.1177/00302228221095905
Chen Y & Laitila	**Publication Year:** 2023<br>**Country/Countries Data was Collected In:** China<br>**Methodology:** Qualitative<br>**Study Design:** Case Study<br>**Sample Size of Bereaved Individuals:** 2<br>**Recruited Age Range:** Unspecified<br>**Sex:** 50% female<br>**Gender**: Unspecified<br>**Time Since Loss (Range or Mean):** Unspecified<br>**Relationship with Lost One(s):** Partner/Spouse, Sibling	"We used assimilation analysis to analyze a single bereavement category, i.e., suicide bereavement, in specifically non-therapeutic research interviews. The research questions were:<br>1. What changes occur in suicide bereavement experiences over the first 18-month period after loss?<br>2. What are the strengths and challenges of using assimilation analysis to analyze changes in the suicide bereavement process? (p.2)"	Caregiving and Parenting<br>Long-term Support Groups and Professional Support<br>Reading (To Heal)<br>Social Activities (General)<br>Work	Leisure<br>Productivity<br>Self-Care	Change Freq./Method of Engag.<br>Continued Engagement<br>New Engagement	Activities to Survive Loss<br>Activities to Manage and Process Loss	Chen, Y., & Laitila, A. (2023). Longitudinal Changes in Suicide Bereavement Experiences: A Qualitative Study of Family Members over 18 Months after Loss. *International Journal of Environmental Research and Public Health*, *20*(4), 3013. https://doi.org/10.3390/ijerph20043013
Contessa et al.	**Publication Year:** 2023<br>**Country/Countries Data was Collected In:** Brazil<br>**Methodology:** Qualitative<br>**Study Design:** Unspecified Qualitative: Semi-structured Interviews<br>**Sample Size of Bereaved Individuals:** 41<br>**Recruited Age Range:** 16 - 75<br>**Sex:** 78% female<br>**Gender:** Identified as men (22%) and women (78%)<br>**Time Since Loss (Range or Mean):** 0.167 - 1 year<br>**Relationship with Lost One(s):** Parent, Sibling, Child, Grandparent, Extended family member (Niece/Cousin/Aunt), Partner/Spouse, Grandchild, Children in law, Ex-partner	This study aimed to explore trauma-related phenomena after exposure to suicide. (p.739)	Eating<br>Everyday Activities (General)<br>Sleep<br>Social Activities (General)<br>Work	Leisure<br>Productivity<br>Self-Care	Change Freq./Method of Engag.<br>Disengagement	Activities to Survive Loss	Contessa, J. C., Padoan, C. S., Silva, J. L. G. D., & Magalhães, P. V. S. (2023). A Qualitative Study on Traumatic Experiences of Suicide Survivors. *Omega*, *87*(3), 730–744. https://doi.org/10.1177/00302228211024486
Creuzé et al.	**Publication Year:** 2022<br>**Country/Countries Data was Collected In:** France<br>**Methodology:** Qualitative<br>**Study Design:** Unspecified Qualitative: Semi-structured Interviews<br>**Sample Size of Bereaved Individuals:** 16<br>**Recruited Age Range:** 35-84<br>**Sex:** 75% female<br>**Gender:** 25% identified as male, 75% identified as female<br>**Time Since Loss (Range or Mean):** 1-27 years<br>**Relationship with Lost One(s):** Child, Partner/Spouse, Sibling	"Our objectives were thus to qualitatively (a) assess the impact of suicide on different types of family members, (b) evaluate the interactions between the familial and individual bereavement processes, and (c) obtain precise insights into the familial interactions that occur following a suicide." (p.1)	Caregiving and Parenting<br>Taking on Roles of Family Members, Change in Family Life	Productivity	Change Freq./Method of Engag.<br>Re-engagement	Activities to Manage and Process Loss<br>Activities to Move Forward	Creuzé, C., Lestienne, L., Vieux, M., Chalancon, B., Poulet, E. & Leaune, E. (2022). Lived Experiences of Suicide Bereavement within Families: A Qualitative Study. *International Journal of Environmental Research and Public Health*, *19*, 13070. https://doi.org/10.3390/ijerph 192013070

Authors	Publication Year, Country/Countries Data was Collected In, Type of Evidence, Methodology, Study Design, Sample Size of Bereaved Individuals, Recruited Age Range, Sex, Gender, Time Since Loss Range or Mean, Relationship with Lost One(s)	Aim of Study (verbatim)	Specific Activities of Everyday Living Discussed	Activities of Everyday Living Discussed	Engagement Status Discussed	Meanings Associated Discussed	Reference
Cutrer-Párraga et al.	**Publication Year:** 2022<br>**Country/Countries Data was Collected In:** United States<br>**Methodology:** Qualitative<br>**Study Design:** Collective Case Study Design<br>**Sample Size of Bereaved Individuals:** 3<br>**Recruited Age Range:** Unspecified<br>**Sex:** 67% female<br>**Gender**: Unspecified<br>**Time Since Loss (Range or Mean):** Unspecified<br>**Relationship with Lost One(s):** Parent	"The current study focuses on the under-researched population of CSoPS [children survivors of parental suicide], investigating their perceptions of children's books that may provide postvention support (bibliotherapy)." (p.1840)	Caregiving and Parenting<br>School or Studies	Productivity	Continued Engagement<br>New Engagement<br>Re-engagement	Activities to Survive Loss<br>Activities to Manage and Process Loss	Cutrer-Párraga, E. A., Cotton, C., Heath, M. A., Miller, E. E., Young, T. A., & Wilson, S. N. (2022). Three Sibling Survivors' Perspectives of their Father's Suicide: Implications for Postvention Support. *Journal of child and family studies, 31*(7), 1838–1858. https://doi.org/10.1007/s10826-022-02308-y
Doehring et al.	**Publication Year:** 2019<br>**Country/Countries Data was Collected In:** United States<br>**Methodology:** Qualitative<br>**Study Design:** Pastoral Theological Method<br>**Sample Size of Bereaved Individuals:** 1<br>**Recruited Age Range:** Unspecified<br>**Sex:** 100% female<br>**Gender**: Unspecified<br>**Time Since Loss (Range or Mean):** Unspecified<br>**Relationship with Lost One(s):** Child	"This article argues that spiritual practices revealing compassion and benevolence in embodied, relational, and transcendent ways help people search for meanings that are flexible, integrated, and complex enough to bear the weight of suffering." (p.242)	Music, Radio, TV	Leisure	Continued Engagement	Activities to Survive Loss<br>Activities to Manage and Process Loss<br>Activities to Move Forward	Doehring, C. (2019). Searching for wholeness amidst traumatic grief: The role of spiritual practices that reveal compassion in embodied, relational, and transcendent ways. *Pastoral Psychology, 68*(3), 241–259. https://doi.org/10.1007/s11089-018-0858-5
Dransart et al.	**Publication Year:** 2017<br>**Country/Countries Data was Collected In:** Switzerland<br>**Methodology:** Qualitative<br>**Study Design:** Grounded Theory<br>**Sample Size of Bereaved Individuals:** 50<br>**Recruited Age Range:** Unspecified<br>**Sex:** 78% female<br>**Gender**: Unspecified<br>**Time Since Loss (Range or Mean):** Unspecified-16 years<br>**Relationship with Lost One(s):** Child, Extended Family Member (Niece / Nephew), Friend, Parent, Partner/Spouse, Sibling	"The present study aims to identify patterns of reconstruction (if any) for survivors (processes and components)." (p.995)	Baking<br>Housework, Chores<br>Long-term Support Groups and Professional Support<br>Religious Activities<br>Peer Group Facilitation<br>Social Activities (General)<br>Taking on Roles of Family Members, Change in Family Life	Leisure<br>Productivity<br>Self-Care	Change Freq./Method of Engag.<br>Disengagement<br>New Engagement	Activities to Survive Loss<br>Activities to Manage and Process Loss<br>Activities to Move Forward	Dransart D. A. C. (2017). Reclaiming and Reshaping Life: Patterns of Reconstruction After the Suicide of a Loved One. *Qualitative health research, 27*(7), 994–1005. https://doi.org/10.1177/1049732316637590
Dransart et al.	**Publication Year:** 2013<br>**Country/Countries Data was Collected In:** Switzerland<br>**Methodology:** Qualitative<br>**Study Design:** Grounded Theory<br>**Sample Size of Bereaved Individuals:** 48<br>**Recruited Age Range:** 20-79<br>**Sex:** 77% female<br>**Gender**: Unspecified<br>**Time Since Loss (Range or Mean):** 0.25-19 years<br>**Relationship with Lost One(s):** Child, Extended Family Member (Niece / Nephew), Friend, Parent, Partner/Spouse, Sibling	"Our study investigated how survivors were able (or were not able) to construct comprehensibility (finding some sort of explanation) and significance (meaning, finding something of personal value in the experience) (JanoffBulman and Frantz, 1997) after the suicide event, both on a personal and on a social level." (p.319)	Joining Advocacy Groups or Organizations	Productivity	New Engagement	Activities to Move Forward	Dransart, D. A. C. (2013). From sense-making to meaning-making: Understanding and supporting survivors of suicide. *British Journal of Social Work, 43*(2), 317–335. https://doi.org/10.1093/bjsw/bct026
Eng et al.	**Publication Year:** 2019<br>**Country/Countries Data was Collected In:** England<br>**Methodology:** Qualitative<br>**Study Design:** Qualitative Cross-Sectional Study Design and Online Survey<br>**Sample Size of Bereaved Individuals:** 346<br>**Recruited Age Range:** 18-40<br>**Sex:** 81% female<br>**Gender:** 19% identified as male, 81% identified as female<br>**Time Since Loss (Range or Mean):** Unspecified<br>**Relationship with Lost One(s):** Child, Colleague/Co-worker, Ex-Partner, Extended Family Member (aunt/uncle/cousin/niece/nephew), Friend, Grandparent, Parent, Parent in law, Partner/Spouse, Sibling, Sibling in law, Undisclosed, Other	"Our aim was to use qualitative approaches in analysing data collected from an anonymous online survey to gain a more nuanced understanding of any changes in use of alcohol or drugs after a suicide bereavement, and the motivations behind any changes." (p.2)	Alcohol and Drug Use, Risky and Sexual Behaviours<br>Sleep	Self-Care	Change Freq./Method of Engag.<br>Continued Engagement<br>Disengagement	Activities to Survive Loss<br>Activities to Move Forward	Eng, J., Drabwell, L., Stevenson, F., King, M., Osborn, D., & Pitman, A. (2019). Use of Alcohol and Unprescribed Drugs after Suicide Bereavement: Qualitative Study. *International journal of environmental research and public health, 16*(21), 4093. https://doi.org/10.3390/ijerph16214093
Entilli et al.	**Publication Year:** 2021<br>**Country/Countries Data was Collected In:** Australia<br>**Methodology:** Qualitative<br>**Study Design:** Qualitative Longitudinal Study<br>**Sample Size of Bereaved Individuals:** 11<br>**Recruited Age Range:** 50-78<br>**Sex:** 45% female<br>**Gender**: Unspecified<br>**Time Since Loss (Range or Mean):** Unspecified<br>**Relationship with Lost One(s):** Child	"The present study aims to extend the longitudinal qualitative study to include a follow-up analysis of the next wave of data—parents' experiences at 24 months taking into consideration the overall 2-year window and the 3 separate time observations of parents at 6, 12, and 24 months after the suicide of their child." (p.2)	Alcohol and Drug Use, Risky and Sexual Behaviours<br>Enjoyed Activities, Hobbies (General)<br>Exercise<br>Gaming<br>Long-term Support Groups and Professional Support<br>Religious Activities<br>Routines and Rituals Related to Lost one<br>Sleep<br>Work<br>Writing (Journaling, Letter Writing)	Leisure<br>Productivity<br>Self-Care	Change Freq./Method of Engag.<br>Disengagement<br>New Engagement	Activities to Survive Loss<br>Activities to Manage and Process Loss<br>Activities to Move Forward	Entilli, L., Ross, V., De Leo, D., Cipolletta, S. & Kõlves, K. (2021). Experiences of Parental Suicide-Bereavement: A Longitudinal Qualitative Analysis Over Two Years. *International Journal of Environmental Research and Public Health. 18*, 564. https://doi.org/10.3390/ ijerph18020564

Authors	Publication Year, Country/Countries Data was Collected In, Type of Evidence, Methodology, Study Design, Sample Size of Bereaved Individuals, Recruited Age Range, Sex, Gender, Time Since Loss Range or Mean, Relationship with Lost One(s)	Aim of Study (verbatim)	Specific Activities of Everyday Living Discussed	Activities of Everyday Living Discussed	Engagement Status Discussed	Meanings Associated Discussed	Reference
Entilli et al.	**Publication Year:** 2023<br>**Country/Countries Data was Collected In:** Italy<br>**Methodology:** Quantitative<br>**Study Design:** Cross-sectional Study<br>**Sample Size of Bereaved Individuals:** 132<br>**Recruited Age Range:** 18 - Unspecified<br>**Sex:** 79% female<br>**Gender**: Unspecified<br>**Time Since Loss (Mean):** 5.52 years<br>**Relationship with Lost One(s):** Partner/Spouse, Close Relative, Non-relative	This cross-sectional study explores the psychological state and perceived social support of Italian survivors, including those who have not sought for help, and investigates differences for gender or kinship with the departed. (p.1)	Long-term Support Groups and Professional Support	Self-Care	New Engagement	Activities to Manage and Process Loss	Entilli, L., Leo, D. D., Aiolli, F., Polato, M., Gaggi, O., & Cipolletta, S. (2023). Social Support and Help-Seeking Among Suicide Bereaved: A Study With Italian Survivors. *OMEGA - Journal of Death and Dying*, *87*(2), 534-553. https://doi.org/10.1177/00302228211024112
Eskin et al.	**Publication Year:** 2024<br>**Country/Countries Data was Collected In:** Turkey<br>**Methodology:** Qualitative<br>**Study Design:** Unspecified Qualitative: Thematic Analysis<br>**Sample Size of Bereaved Individuals:** 73<br>**Recruited Age Range:** 19 - 56<br>**Sex:** 86% female<br>**Gender**: Unspecified<br>**Time Since Loss (Range or Mean):** 0.1 - 30 years (mean: 7.8 years)<br>**Relationship with Lost One(s):** First degree relative, Second degree relative, Partner/Spouse, Ex-partner, Friend, Acquaintance	The present study aims to investigate the Turkish suicide-loss survivors' bereavement experiences by utilizing a qualitative methodology. In addition, this study further explores the support needs of suicide-loss survivors to understand how to help them and inform psychological interventions. (p.2)	Caregiving and Parenting<br>Everyday Activities (General)<br>Long-term Support Groups and Professional Support<br>School or Studies<br>Social Activities (General)<br>Taking on Roles of Family Members, Change in Family Life	Leisure<br>Productivity<br>Self-Care	Change Freq./Method of Engag.<br>Disengagement<br>New Engagement	Activities to Survive Loss<br>Activities to Manage and Process Loss	Eskin, M., Karkin, A. N., Eyisoylu, E., Seker, E., Yilmaz, E., Sevin, G., ... Ranjbar, H. A. (2024). The experiences and support needs of Turkish individuals bereaved by suicide: An online qualitative investigation. *Death Studies*, 1–13. https://doi.org/10.1080/07481187.2024.2386062
Feigelman et al.	**Publication Year:** 2019<br>**Country/Countries Data was Collected In:** United States<br>**Methodology:** Quantitative<br>**Study Design:** Quantitative Survey Design<br>**Sample Size of Bereaved Individuals:** 516<br>**Recruited Age Range:** Unspecified<br>**Sex:** Not Specified<br>**Gender**: Unspecified<br>**Time Since Loss (Mean):** 14 years<br>**Relationship with Lost One(s):** First-degree relative, Friend	"Aims: We sought to examine whether suicide-bereaved adults were any differently disposed to religious participation and observances compared with the nonbereaved and whether religiously involved bereaved had any better mental health compared with religiously disaffiliated bereaved." (p.176)	Religious Activities	Self-Care	Continued Engagement		Feigelman, W., Cerel, J., McIntosh, J. L., Brent, D., & Gutin, N. (2019). Suicide Bereavement and Differences in Religiosity. *Crisis*, *40*(3), 176–185. https://doi.org/10.1027/0227-5910/a000546
Feigelman et al.	**Publication Year:** 2024<br>**Country/Countries Data was Collected In:** United States<br>**Methodology:** Quantitative<br>**Study Design:** Unspecified Quantitative: Online Survey<br>**Sample Size of Bereaved Individuals:** 1132<br>**Recruited Age Range:** 20 - 65+<br>**Sex:** 69% female<br>**Gender**: Unspecified<br>**Time Since Loss (Range or Mean):** <1 - 6 years (mean: 2.96 years)<br>**Relationship with Lost One(s):** Parent, Child, Sibling, Partner/Spouse, Other	Focusing on the understudied question of substance misuse among suicide bereaved adults we investigated patterns of binge drinking and non-prescribed drug use among a recently bereaved sample. (p.1)	Alcohol and Drug Use, Risky and Sexual Behaviours	Self-Care	Change Freq./Method of Engag.		Feigelman, W., Cerel, J., Gutin, N., McIntosh, J. L., Gorman, B. S., Bottomley, J. S., & Edwards, A. (2024). Examining the Associations Between Substance Misuse and Suicide Bereavement. *OMEGA - Journal of Death and Dying*. https://doi.org/10.1177/00302228241254133
Ferlatte et al.	**Publication Year:** 2019<br>**Country/Countries Data was Collected In:** Canada<br>**Methodology:** Qualitative<br>**Study Design:** Descriptive Case Study and Photovoice Methods<br>**Sample Size of Bereaved Individuals:** 2<br>**Recruited Age Range:** 40-49<br>**Sex:** 0% female<br>**Gender**: Unspecified<br>**Time Since Loss (Range or Mean):** Unspecified<br>**Relationship with Lost One(s):** Partner/Spouse	"Meanwhile, attention to gay men bereaved by the suicide of a male partner has been conspicuously absent from the literature. [...] To address this gap this article presents a qualitative case study of two gay men who lost a partner to suicide and explores how stigma shapes gay men's bereavement while identifying avenues for supporting the survivors of suicide." (p.1273-1274)	Arts and Crafts<br>Long-term Support Groups and Professional Support<br>Work	Leisure<br>Productivity<br>Self-Care	Change Freq./Method of Engag.<br>Continued Engagement<br>New Engagement	Activities to Survive Loss<br>Activities to Manage and Process Loss	Ferlatte, O., Oliffe, J. L., Salway, T., & Knight, R. (2019). Stigma in the bereavement experiences of gay men who have lost a partner to suicide. *Culture, Health & Sexuality*, *21*(11), 1273–1289. https://doi.org/10.1080/13691058.2018.1556344
Fraccaro & Tosini	**Publication Year:** 2023<br>**Country/Countries Data was Collected In:** Italy<br>**Methodology:** Qualitative<br>**Study Design:** Unspecified Qualitative: Semi-structured Interviews<br>**Sample Size of Bereaved Individuals:** 20<br>**Recruited Age Range:** 10 - 79<br>**Sex:** 90% female<br>**Gender**: Unspecified<br>**Time Since Loss (Range or Mean):** Unspecified<br>**Relationship with Lost One(s):** Sibling, Child, Partner/Spouse, Ex-Partner, Extended family member (Nephew/Niece/Uncle/Aunt), Sibling in law	We examined social mechanisms of suicide support groups based on evidence from an Italian postvention program. [...] Our paper aimed to understand these interactional processes based on evidence from a suicide-specific group of an Italian postvention program. (p.1-2)	Long-term Support Groups and Professional Support	Self-Care	New Engagement	Activities to Manage and Process Loss	Fraccaro, D., & Tosini, D. (2023). The suicide support group as a signifying agent and emotion transformer: A contribution from a micro-sociological perspective. *Death Studies, 48*(6), 550–560. https://doi.org/10.1080/07481187.2023.2246037

Authors	Publication Year, Country/Countries Data was Collected In, Type of Evidence, Methodology, Study Design, Sample Size of Bereaved Individuals, Recruited Age Range, Sex, Gender, Time Since Loss Range or Mean, Relationship with Lost One(s)	Aim of Study (verbatim)	Specific Activities of Everyday Living Discussed	Activities of Everyday Living Discussed	Engagement Status Discussed	Meanings Associated Discussed	Reference
Froese & McDermott	**Publication Year:** 2024<br>**Country/Countries Data was Collected In:** Canada<br>**Methodology:** Qualitative<br>**Study Design:** Interpretative phenomenological design<br>**Sample Size of Bereaved Individuals:** 3<br>**Recruited Age Range:** 28 - 90<br>**Sex:** 100% female<br>**Gender:** Unspecified<br>**Time Since Loss (Range or Mean):** 7 - 25 years (mean: 16 years)<br>**Relationship with Lost One(s):** Parent, Sibling, Friend	The study's purpose was to explore the meanings and significance of leisure in the lives of suicide survivors, guided by the specific research question, "What is it like to journey towards healing within sui- cide grief?" We first begin by contextualizing suicide survivorship and grief in the litera- ture. Next, we situate this in relation to leisure. Third, we overview our methodological approach, followed by a presentation of our findings and their discussion.	Gardening<br>Religious Activities	Leisure<br>Self-Care	Continued Engagement	Activities to Manage and Process Loss	Froese, J., & McDermott, L. (2024). "When All the Wheels Fall off": Leisure's Potential Role in Living with Suicide Loss. *Leisure Sciences*, *46*(4), 405–424. https://doi.org/10.1080/01490400.2021.1985663
Gallardo-Flores et al.	**Publication Year:** 2023<br>**Country/Countries Data was Collected In:** Spain<br>**Methodology:** Mixed methods<br>**Study Design:** Interviews and exploratory analysis<br>**Sample Size of Bereaved Individuals:** 21<br>**Recruited Age Range:** 26 - 69<br>**Sex:** 86% female<br>**Gender:** Unspecified<br>**Time Since Loss (Range or Mean):** 0.66 - 23 years (mean: 4.3 years)<br>**Relationship with Lost One(s):** Partner, Sibling, Child, Parent	(1) To describe, with reference to Grotberg's model, the manifestations of resilient behaviors in the people comprising the sample and; (2) To describe, with reference to Knight's model, the manifestations of resilient behaviors by the people in the sample.	Social Activities (General)<br>Work	Leisure<br>Productivity	Change Freq./Method of Engag.<br>Disengagement	Activities to Survive Loss<br>Activities to Manage and Process Loss	Gallardo-Flores, A., Morán-Carrillo, J.-M., & García-Carmona, M. (2023). The Detection of Resilience in Families Grieving Over a Suicide. *Omega: Journal of Death and Dying*, 302228231219047–302228231219047. https://doi.org/10.1177/00302228231219047
Genest et al.	**Publication Year:** 2021<br>**Country/Countries Data was Collected In:** Canada<br>**Methodology:** Qualitative<br>**Study Design:** Grounded Theory<br>**Sample Size of Bereaved Individuals:** 17<br>**Recruited Age Range:** Unspecified<br>**Sex:** Unspecified<br>**Gender:** Unspecified<br>**Time Since Loss (Range or Mean):** 4-10 years<br>**Relationship with Lost One(s):** Child, Sibling, Step-Children, Step-Sibling	"The present qualitative study was undertaken to more fully explore the process of post-traumatic growth in bereaved families of an adolescent's suicide so family nurses could build upon this knowledge to work with newly bereaved family. [...] The specific research question was how do families transform themselves after an adolescent suicide?" (p.296)	Long-term Support Groups and Professional Support<br>Reading (To Heal)<br>Routines and Rituals Related to Lost one<br>Social Activities (General)<br>Taking on Roles of Family Members, Change in Family Life<br>Writing (Journaling, Letter Writing)	Leisure<br>Productivity<br>Self-Care	Change Freq./Method of Engag.<br>New Engagement<br>Re-engagement	Activities to Survive Loss<br>Activities to Manage and Process Loss<br>Activities to Move Forward	Genest, C., Gratton, F., O'Reilly, T., Allard, É., & Maltais, N. (2021). Emerging Despite the Indelible Wound: A Grounded Theory of Family Transformation Following Adolescent Suicide. *Journal of family nursing*, *27*(4), 295–303. https://doi.org/10.1177/10748407211006183
George, M.	**Publication Year:** 2023<br>**Country/Countries Data was Collected In:** Japan<br>**Methodology:** Quantitative<br>**Study Design:** Case Study (Personal Account)<br>**Sample Size of Bereaved Individuals:** 1<br>**Recruited Age Range:** Unspecified<br>**Sex:** 100% female<br>**Gender:** Identified as women (100%)<br>**Time Since Loss (Range or Mean):** 3 years<br>**Relationship with Lost One(s):** Parent	This article aims to discuss what happened when my mother completed suicide and the fallout caused by it as well.	Long-term Support Groups and Professional Support<br>Work	Productivity<br>Self-Care	New Engagement	Activities to Manage and Process Loss<br>Activities to Move Forward	George, M. (2023). Flipping up the board: Losing my mother to suicide. *Archives of Psychiatric Nursing*, *47*, 7–9. https://doi.org/10.1016/j.apnu.2023.10.003
Ghetti et al.	**Publication Year:** 2024<br>**Country/Countries Data was Collected In:** Norway<br>**Methodology:** Quantitative<br>**Study Design:** Action Research Single Case Study<br>**Sample Size of Bereaved Individuals:** 1<br>**Recruited Age Range:** Unspecified<br>**Sex:** 0% female<br>**Gender:** Unspecified<br>**Time Since Loss (Range or Mean):** Unspecified<br>**Relationship with Lost One(s):** Child	We used dialogic reflection among a recipient of ACPR music therapy, his music therapist, and a music therapy researcher to explore an exceptional example of what the ACPR process can afford a bereaved parent across time and contexts, to inspire change in care systems.	Music, Radio, TV	Leisure	Change Freq./Method of Engag.	Activities to Manage and Process Loss	Ghetti, C. M., Schreck, B., & Bennett, J. (2024). Heartbeat recordings in music therapy bereavement care following suicide: Action research single case study of amplified cardiopulmonary recordings for continuity of care. *Action Research (London, England)*, *22*(4), 362–380. https://doi.org/10.1177/14767503231207993
Gordon, E., & Mcelvaney, R.	**Publication Year:** 2022<br>**Country/Countries Data was Collected In:** Ireland<br>**Methodology:** Qualitative<br>**Study Design:** Interpretive Phenomenological Design<br>**Sample Size of Bereaved Individuals:** 5<br>**Recruited Age Range:** 19-66<br>**Sex:** 100% female<br>**Gender:** Unspecified<br>**Time Since Loss (Range or Mean):** 1.25-3.17 years<br>**Relationship with Lost One(s):** Child, Children in law, Extended Family Member (Cousin), Extended Family Member (Nephew/Niece)	"Building on this work, this study sought to qualitatively examine suicide bereavement and help-seeking experiences among a range of female relatives who engaged with bereavement support to address the research question "How do women in Ireland experience the suicide of a male relative?"" (p.397-398)	Caregiving and Parenting<br>Gardening<br>Long-term Support Groups and Professional Support<br>Religious Activities<br>Routines and Rituals Related to Lost one	Leisure<br>Productivity<br>Self-Care	Change Freq./Method of Engag.<br>Continued Engagement<br>New Engagement	Activities to Survive Loss<br>Activities to Manage and Process Loss<br>Activities to Move Forward	Gordon, E., & Mcelvaney, R.. (2022). Directing from the shadows: Women's experiences of male relative suicide bereavement. *Journal of Family Therapy*, *44*(3), 396–407. https://doi.org/10.1111/1467-6427.12388

Authors	Publication Year, Country/Countries Data was Collected In, Type of Evidence, Methodology, Study Design, Sample Size of Bereaved Individuals, Recruited Age Range, Sex, Gender, Time Since Loss Range or Mean, Relationship with Lost One(s)	Aim of Study (verbatim)	Specific Activities of Everyday Living Discussed	Activities of Everyday Living Discussed	Engagement Status Discussed	Meanings Associated Discussed	Reference
Goulah-Pabst et al.	**Publication Year:** 2021<br>**Country/Countries Data was Collected In:** United States<br>**Methodology:** Qualitative<br>**Study Design:** Interpretive Phenomenological Design<br>**Sample Size of Bereaved Individuals:** 14<br>**Recruited Age Range:** 36-93<br>**Sex:** 64% female<br>**Gender:** Unspecified<br>**Time Since Loss (Range or Mean):** 2-35 years<br>**Relationship with Lost One(s):** Child, Extended Family Member (Nephew), Parent, Partner/Spouse, Sibling	"The purpose of this study is to grasp how suicide loss survivors navigate stigma, threats to social bonds, and difficult emotions associated with suicide loss and what coping strategies and meaning making processes contribute to their healing through social interactions." (p.772)	Caregiving and Parenting<br>Exercise<br>Film Production<br>Joining Advocacy Groups or Organizations<br>Long-term Support Groups and Professional Support<br>Peer Group Facilitation<br>Religious Activities<br>School or Studies<br>Self-Care (General)<br>Social Activities (General)<br>Volunteer work (Advocacy)<br>Work<br>Yoga	Leisure<br>Productivity<br>Self-Care	Change Freq./Method of Engag.<br>Continued Engagement<br>Disengagement<br>New Engagement<br>Re-engagement	Activities to Survive Loss<br>Activities to Manage and Process Loss<br>Activities to Move Forward	Goulah-Pabst, D. M. (2021). Suicide Loss Survivors: Navigating Social Stigma and Threats to Social Bonds. *OMEGA - Journal of Death and Dying, 87*(3), 769-792. https://doi.org/10.1177/00302228211026513
Groos & Shakespeare-Finch	**Publication Year:** 2013<br>**Country/Countries Data was Collected In:** Australia<br>**Methodology:** Qualitative<br>**Study Design:** Grounded Theory<br>**Sample Size of Bereaved Individuals:** 13<br>**Recruited Age Range:** 21-60<br>**Sex:** 92% female<br>**Gender:** Unspecified<br>**Time Since Loss (Range or Mean):** <1-7 years<br>**Relationship with Lost One(s):** Child, Extended Family Member (Nephew), Partner/Spouse, Sibling, Other: son of a friend	"Cerel et al. (2009) identified that it is important to evaluate support groups to determine the most helpful approaches and benefits gained from participating in such groups. This study addresses this call for further research by examining the experience of people attending suicide bereavement groups offered by Lifeline Community Care Brisbane, Australia." (p.2)	Activities to connect with lost one (hobbies enjoyed by lost one)<br>Caregiving and Parenting<br>Religious Activities<br>Self-Care (General)<br>Taking on Roles of Family Members, Change in Family Life<br>Work	Leisure<br>Productivity<br>Self-Care	Change Freq./Method of Engag.<br>New Engagement<br>Re-engagement	Activities to Manage and Process Loss<br>Activities to Move Forward	Groos, A. D., & Shakespeare-Finch, J. (2013). Positive experiences for participants in suicide bereavement groups: a grounded theory model. *Death studies, 37*(1), 1–24. https://doi.org/10.1080/07481187.2012.687898
Hafford-Letchfield et al.	**Publication Year:** 2022<br>**Country/Countries Data was Collected In:** United Kingdom<br>**Methodology:** Qualitative<br>**Study Design:** Phenomenological Design<br>**Sample Size of Bereaved Individuals:** 24<br>**Recruited Age Range:** 60-94<br>**Sex:** 88% female<br>**Gender:** Unspecified<br>**Time Since Loss (Range or Mean):** 1-20 years<br>**Relationship with Lost One(s):** Extended Family Member (Aunt/Uncle), Grandparent, Parent, Parent in law, Sibling, Spouse/Partner	"This study sought to explore the experience of being bereaved by suicide on the individual in later life, the implications for help-seeking, support needs and how bereavement by suicide interacts with ageing experiences." (p.1)	Caregiving and Parenting<br>Joining Advocacy Groups or Organizations<br>Long-term Support Groups and Professional Support<br>Mental Health Advocacy Activities<br>Work	Productivity<br>Self-Care	Change Freq./Method of Engag.<br>Disengagement<br>New Engagement	Activities to Survive Loss<br>Activities to Manage and Process Loss<br>Activities to Move Forward	Hafford-Letchfield, T., Hanna, J., Grant, E., Ryder-Davies, L., Cogan, N., Goodman, J., Rasmussen, S., & Martin, S. (2022). "It's a Living Experience": Bereavement by Suicide in Later Life. *International journal of environmental research and public health, 19*(12), 7217. https://doi.org/10.3390/ijerph19127217
Heffel et al.	**Publication Year:** 2015<br>**Country/Countries Data was Collected In:** United States<br>**Methodology:** Qualitative<br>**Study Design:** Consensual Qualitative Research<br>**Sample Size of Bereaved Individuals:** 10<br>**Recruited Age Range:** Unspecified<br>**Sex:** 60% female<br>**Gender:** Unspecified<br>**Time Since Loss (Range or Mean):** Unspecified<br>**Relationship with Lost One(s):** Classmate	"The purpose of this study was to explore the experiences of adolescents following a suicide cluster, with a specific focus on the role of online social networking." (p.286)	Caregiving and Parenting<br>Housework, Chores<br>Internet Sites and Social Media<br>Religious Activities<br>Routines and Rituals Related to Lost one<br>School or Studies<br>Social Activities (General)<br>Taking on Roles of Family Members, Change in Family Life	Leisure<br>Productivity<br>Self-Care	Change Freq./Method of Engag.<br>Disengagement<br>New Engagement	Activities to Survive Loss<br>Activities to Manage and Process Loss<br>Activities to Move Forward	Heffel, C. J., Riggs, S. A., Ruiz, J. M., & Ruggles, M.. (2015). The Aftermath of a Suicide Cluster in the Age of Online Social Networking: a Qualitative Analysis of Adolescent Grief Reactions. *Contemporary School Psychology, 19*(4), 286–299. https://doi.org/10.1007/s40688-015-0060-z
Honoré et al.	**Publication Year:** 2024<br>**Country/Countries Data was Collected In:** France<br>**Methodology:** Qualitative<br>**Study Design:** Grounded Theory Informed Qualitative Study<br>**Sample Size of Bereaved Individuals:** 15<br>**Recruited Age Range:** 27 - 75<br>**Sex:** 80% female<br>**Gender:** Unspecified<br>**Time Since Loss (Range or Mean):** 2 - 42 years (mean: 11.6 years)<br>**Relationship with Lost One(s):** Parent, Child, Sibling, Partner/Spouse	Our study aimed to explore the role of spirituality after suicide bereavement, considering the scarcity of data in the scientific literature on this topic. A grounded theory-informed qualitative design was the most suitable method for our exploratory study on how and why people use spirituality to cope with suicide loss. (p.2)	Gardening<br>Meditation<br>Religious Activities<br>Walking	Leisure<br>Self-Care	Change Freq./Method of Engag.<br>Continued Engagement<br>New Engagement	Activities to Manage and Process Loss	Honoré, F., Lestienne, L., Vieux, M., Bislimi, K., Chalancon, B., & Leaune, E. (2024). "A sign that I am not alone": A grounded theory-informed qualitative study on spirituality after suicide bereavement. *Death Studies*, 1–12. https://doi.org/10.1080/07481187.2024.2355250
Hultsjö et al.	**Publication Year:** 2022<br>**Country/Countries Data was Collected In:** Sweden<br>**Methodology:** Qualitative<br>**Study Design:** Phenomenological Design<br>**Sample Size of Bereaved Individuals:** 10<br>**Recruited Age Range:** 34-77<br>**Sex:** 90% female<br>**Gender:** Unspecified<br>**Time Since Loss (Range or Mean):** 1-13 years<br>**Relationship with Lost One(s):** Child, Parent, Parent in law, Partner/Spouse	"Purpose: To explore the loss of a relative due to suicide. [...] This article explores survivors' experiences of both living through a suicidal progression with their loved one and becoming a suicide survivor, to understand the lived experience of witnessing a suicidal progression." (p.1-2)	Eating<br>Exercise<br>Long-term Support Groups and Professional Support<br>Routines and Rituals Related to Lost one<br>Social Activities (General)<br>Work	Leisure<br>Productivity<br>Self-Care	Change Freq./Method of Engag.<br>Continued Engagement<br>New Engagement<br>Re-engagement	Activities to Survive Loss<br>Activities to Manage and Process Loss<br>Activities to Move Forward	Hultsjö, S., Ovox, S. M., Olofsson, C., Bazzi, M., & Wärdig, R. (2022). Forced to move on: An interview study with survivors who have lost a relative to suicide. *Perspectives in psychiatric care, 58*(4), 2215–2223. https://doi.org/10.1111/ppc.13049

Authors	Publication Year, Country/Countries Data was Collected In, Type of Evidence, Methodology, Study Design, Sample Size of Bereaved Individuals, Recruited Age Range, Sex, Gender, Time Since Loss Range or Mean, Relationship with Lost One(s)	Aim of Study (verbatim)	Specific Activities of Everyday Living Discussed	Activities of Everyday Living Discussed	Engagement Status Discussed	Meanings Associated Discussed	Reference
Hunt et al.	**Publication Year:** 2019<br>**Country/Countries Data was Collected In:** United States<br>**Methodology:** Qualitative<br>**Study Design:** Unspecified Qualitative: Interviews and Thematic Analysis informed by Grounded Theory methods<br>**Sample Size of Bereaved Individuals:** 10<br>**Recruited Age Range:** 30-72<br>**Sex:** 40% female<br>**Gender:** Unspecified<br>**Time Since Loss (Range or Mean):** 5-30 years<br>**Relationship with Lost One(s):** Child, Ex-partner, Extended Family Member (Cousin), Grandfather, Sibling	"The purpose of this study is twofold: (1) to develop understanding of the experience of long-term suicide loss survivors and (2) begin to develop a theory of the process." (p.336)	Alcohol and Drug Use, Risky and Sexual Behaviours<br>Eating<br>Religious Activities<br>School or Studies<br>Taking on Roles of Family Members, Change in Family Life<br>Work	Productivity<br>Self-Care	Change Freq./Method of Engag.<br>Disengagement<br>New Engagement	Activities to Survive Loss	Hunt, Q. A., Young, T. A., & Hertlein, K. M.. (2019). The Process of Long-Term Suicide Bereavement: Responsibility, Familial Support, and Meaning Making. *Contemporary Family Therapy, 41*(4), 335–346. https://doi.org/10.1007/s10591-019-09499-5
Hybholt et al.	**Publication Year:** 2020<br>**Country/Countries Data was Collected In:** Denmark<br>**Methodology:** Qualitative<br>**Study Design:** Unspecified Qualitative: Semi structured Interviews<br>**Sample Size of Bereaved Individuals:** 20<br>**Recruited Age Range:** 60-79<br>**Sex:** 65% female<br>**Gender:** Unspecified<br>**Time Since Loss (Range or Mean):** 0.58-5.5 years<br>**Relationship with Lost One(s):** Child, Child-in-law, Grandchild, Parent, Parent-in-law, Partner/Spouse, Step-children	"The aim of this study was to investigate how people bereaved by suicide at age ≥60 conducted their everyday lives in the first 5 years following the loss of a loved one." (p.1)	Caregiving and Parenting<br>Enjoyed Activities, Hobbies (General)<br>Everyday Activities (General)<br>Housework, Chores<br>Long-term Support Groups and Professional Support<br>Reading (To Heal)<br>Religious Activities<br>Routines and Rituals Related to Lost one<br>Social Activities (General)<br>Volunteer work (General)	Leisure<br>Productivity<br>Self-Care	Change Freq./Method of Engag.<br>New Engagement<br>Re-engagement	Activities to Survive Loss<br>Activities to Manage and Process Loss<br>Activities to Move Forward	Hybholt, L., Berring, L. L., Erlangsen, A., Fleischer, E., Toftegaard, J., Kristensen, E., Toftegaard, V., Havn, J., & Buus, N. (2020). Older Adults' Conduct of Everyday Life After Bereavement by Suicide: A Qualitative Study. *Frontiers in Psychology, 11*. https://doi.org/10.3389/fpsyg.2020.01131
Hybholt et al.	**Publication Year:** 2022<br>**Country/Countries Data was Collected In:** Denmark, Ireland<br>**Methodology:** Qualitative<br>**Study Design:** Qualitative Descriptive Study<br>**Sample Size of Bereaved Individuals:** 27<br>**Recruited Age Range:** Unspecified<br>**Sex:** 70% female<br>**Gender:** Unspecified<br>**Time Since Loss (Range or Mean):** 1-44 years<br>**Relationship with Lost One(s):** Child, Partner/Spouse, Sibling	"The aim of this study was to explore participants' perspectives on peer-led support groups for people bereaved by suicide." (p.3)	Everyday Activities (General)<br>Long-term Support Groups and Professional Support	Self-Care	New Engagement<br>Re-engagement	Activities to Survive Loss<br>Activities to Manage and Process Loss	Hybholt, L., Higgins, A., Buus, N., Berring, L. L., Connolly, T., Erlangsen, A., & Morrissey, J.. (2022). The Spaces of Peer-Led Support Groups for Suicide Bereaved in Denmark and the Republic of Ireland: A Focus Group Study. *International Journal of Environmental Research and Public Health, 19*(16), 9898. https://doi.org/10.3390/ijerph19169898
Jackson et al.	**Publication Year:** 2015<br>**Country/Countries Data was Collected In:** Australia<br>**Methodology:** Qualitative<br>**Study Design:** Narrative Case Study<br>**Sample Size of Bereaved Individuals:** 1<br>**Recruited Age Range:** Unspecified<br>**Sex:** 0% female<br>**Gender:** Unspecified<br>**Time Since Loss (Range or Mean):** Unspecified<br>**Relationship with Lost One(s):** Extended Family Member (Uncle)	sought to explore the experiences of immediate family survivors of completed suicide. Previous findings pertaining to events prior to the completed suicide are reported elsewhere (Peters et al., 2013). In this current article, we provide an in-depth account from a young man who as a child experienced the completed suicide of a close family member, after a long period of suicidality. This article provides an important first-person retrospective account of suicide survivorship through the eyes of a child." (p.496)	Caregiving and Parenting<br>Social Activities (General)	Leisure<br>Productivity	Change Freq./Method of Engag.<br>Disengagement	Activities to Survive Loss<br>Activities to Manage and Process Loss	Jackson, D., Peters, K., & Murphy, G. (2015). Suicide of a close family member through the eyes of a child: A narrative case study report. *Journal of child health care : for professionals working with children in the hospital and community, 19*(4), 495–503. https://doi.org/10.1177/1367493513519297
Karatay & Gürarslan Baş	**Publication Year:** 2023<br>**Country/Countries Data was Collected In:** Turkey<br>**Methodology:** Qualitative<br>**Study Design:** Phenomenological Study<br>**Sample Size of Bereaved Individuals:** 14<br>**Recruited Age Range:** 25 - 75<br>**Sex:** 50% female<br>**Gender:** Unspecified<br>**Time Since Loss (Range or Mean):** Unspecified<br>**Relationship with Lost One(s):** Parent, Child, Sibling, Partner/Spouse	This qualitative research aims to deeply understand the perceptions, experiences, and coping mechanisms of families who have experienced completed suicide in the pre- and post-suicide periods. (p. 732)	Alcohol and Drug Use, Risky and Sexual Behaviours<br>Social Activities (General)	Leisure<br>Self-Care	Change Freq./Method of Engag.<br>Disengagement	Activities to Survive Loss	Karatay, G., & Gürarslan Baş, N. (2023). Understanding Suicides through the Processes of Bereaved Relatives: A Phenomenological Study: Understanding Suicides. *Journal of Loss & Trauma, 28*(8), 727–744. https://doi.org/10.1080/15325024.2023.2217032

Authors	Publication Year, Country/Countries Data was Collected In, Type of Evidence, Methodology, Study Design, Sample Size of Bereaved Individuals, Recruited Age Range, Sex, Gender, Time Since Loss Range or Mean, Relationship with Lost One(s)	Aim of Study (verbatim)	Specific Activities of Everyday Living Discussed	Activities of Everyday Living Discussed	Engagement Status Discussed	Meanings Associated Discussed	Reference
Kasahara-Kiritani et al.	**Publication Year:** 2017<br>**Country/Countries Data was Collected In:** Japan<br>**Methodology:** Qualitative<br>**Study Design:** Unspecified Qualitative: Semi-structured Interviews<br>**Sample Size of Bereaved Individuals:** 24<br>**Recruited Age Range:** 40-60<br>**Sex:** 62.5% female<br>**Gender:** Unspecified<br>**Time Since Loss (Range or Mean):** 0-10 years<br>**Relationship with Lost One(s):** Child, Parent, Partner/Spouse, Sibling	"The present purpose was to depict daily actions/behaviors of the person whose loved one died by suicide." (p.448)	Caregiving and Parenting<br>Everyday Activities (General)<br>Housework, Chores<br>Long-term Support Groups and Professional Support<br>Mental Health Advocacy Activities<br>School or Studies<br>Social Activities (General)<br>Swimming<br>Work<br>Writing (Journaling, Letter Writing)	Leisure<br>Productivity<br>Self-Care	Change Freq./Method of Engag.<br>Disengagement<br>New Engagement<br>Re-engagement	Activities to Survive Loss<br>Activities to Manage and Process Loss<br>Activities to Move Forward	Kasahara-Kiritani, M., Ikeda, M., Yamamoto-Mitani, N., & Kamibeppu, K. (2017). Regaining my new life: Daily lives of suicide-bereaved individuals. *Death Studies, 41*(7), 447–454. https://doi-org.myaccess.library.utoronto.ca/10.1080/07481187.2017.1297873
Kaur H & Singh	**Publication Year:** 2023<br>**Country/Countries Data was Collected In:** India<br>**Methodology:** Qualitative<br>**Study Design:** Case Study<br>**Sample Size of Bereaved Individuals:** 3<br>**Recruited Age Range:** 42 - 53<br>**Sex:** 100% female<br>**Gender:** Unspecified<br>**Time Since Loss (Range or Mean):** 0.42 - 0.75 years (mean: 0.55 years)<br>**Relationship with Lost One(s):** Partner/Spouse	The study examines the efficacy of "Six Thinking Dupatta Skills" as a problem-solving skill for the prevention of suicide in wives who were grieving of Bathinda district (Punjab) who had lost their husbands because of suicide. It was hypothesized that this method would be effective in alleviating suicidal ideation, grief, depression and build resilience in them. (Abstract)	Work	Productivity	New Engagement	Activities to Manage and Process Loss	Kaur, H., & Singh, A. (2023). Six Thinking Dupatta Skills for Problem Solving: Case Study of Suicide Prevention of Wives in Grief. *Journal of Creativity in Mental Health, 18*(4), 477–492. https://doi.org/10.1080/15401383.2021.2000910
Kaur R & Stedmon	**Publication Year:** 2022<br>**Country/Countries Data was Collected In:** England<br>**Methodology:** Qualitative<br>**Study Design:** Interpretative Phenomenological Design<br>**Sample Size of Bereaved Individuals:** 8<br>**Recruited Age Range:** 27-69<br>**Sex:** Unspecified<br>**Gender:** Unspecified<br>**Time Since Loss (Range or Mean):** 5-33 years<br>**Relationship with Lost One(s):** Child, Parent, Sibling	"The study on which this paper is based aimed to:<br>• Expand the research focus beyond acute grief via investigating suicide bereavement related experiences (of any length) that occur over the life course.<br>• Determine the applicability of existing grief theories to understanding suicide bereavement and uncover new theoretical understandings.<br>• Inform evidence-based treatments for suicide-loss-survivors which are currently lacking (Omerov et al., 2014)." (p.55)	Caregiving and Parenting<br>Long-term Support Groups and Professional Support<br>School or Studies<br>Shopping<br>Volunteer work (General)<br>Work	Leisure<br>Productivity<br>Self-Care	Change Freq./Method of Engag.<br>New Engagement	Activities to Survive Loss<br>Activities to Manage and Process Loss<br>Activities to Move Forward	Kaur, R., & Stedmon, J. (2022). A phenomenological enquiry into the impact of bereavement by suicide over the life course. *Mortality, 27*(1), 53–74. https://doi.org/10.1080/13576275.2020.1823351
Leaune et al.	**Publication Year:** 2024<br>**Country/Countries Data was Collected In:** France<br>**Methodology:** Qualitative<br>**Study Design:** Questionnaires<br>**Sample Size of Bereaved Individuals:** 401<br>**Recruited Age Range:** 18 - 80<br>**Sex:** 88.3% female<br>**Gender:** Identified as women (88.3%), men (11.5%) and non-binary (0.2%)<br>**Time Since Loss (Range or Mean):** Unspecified<br>**Relationship with Lost One(s):** Partner/Spsoue, Sibling, Child, Parent	The objective of our study was to evaluate the use of social media in French people bereaved by suicide and to assess their expectations toward social media. (p. 2)	Internet Sites and Social Media	Leisure	Change Freq./Method of Engag.	Activities to Manage and Process Loss<br>Activities to Move Forward	Leaune, E., Rouzé, H., Lestienne, L., Bislimi, K., Morgiève, M., Chalancon, B., Lau-Taï, P., Vaiva, G., Grandgenèvre, P., Haesebaert, J., & Poulet, E. (2024). The use of social media after bereavement by suicide: results from a French online survey. *BMC Psychiatry, 24*(1), 306–310. https://doi.org/10.1186/s12888-024-05761-9
Lee E	**Publication Year:** 2022<br>**Country/Countries Data was Collected In:** South Korea<br>**Methodology:** Qualitative<br>**Study Design:** Descriptive Phenomenological Design<br>**Sample Size of Bereaved Individuals:** 7<br>**Recruited Age Range:** 42-65<br>**Sex:** 57% female<br>**Gender:** Unspecified<br>**Time Since Loss (Range or Mean):** 0.083-2.15 years<br>**Relationship with Lost One(s):** Child, Partner/Spouse	"This study aimed to explore the experiences of suicidally bereaved families in South Korea" (p.1)	Caregiving and Parenting<br>Eating<br>Meditation<br>Religious Activities<br>Work<br>Yoga	Leisure<br>Productivity<br>Self-Care	Change Freq./Method of Engag.<br>Continued Engagement<br>New Engagement<br>Re-engagement	Activities to Survive Loss<br>Activities to Manage and Process Loss	Lee E. (2022). Experiences of Bereaved Families by Suicide in South Korea: A Phenomenological Study. *International journal of environmental research and public health, 19*(5), 2969. https://doi.org/10.3390/ijerph19052969
Lee E et al.	**Publication Year:** 2019<br>**Country/Countries Data was Collected In:** South Korea<br>**Methodology:** Qualitative<br>**Study Design:** Interpretative Phenomenological Design<br>**Sample Size of Bereaved Individuals:** 11<br>**Recruited Age Range:** 20-69<br>**Sex:** 82% female<br>**Gender:** Unspecified<br>**Time Since Loss (Range or Mean):** 0.33-18 years<br>**Relationship with Lost One(s):** Child, Parent, Partner/Spouse, Sibling	"It is our intent to begin filling the gaps in this scientific knowledge of PTG (posttruamatic growth) in the context of a loved one's suicide in Korea. [...] This study seeks to additionally address this aspect to understand how families can cope positively in the aftermath of a traumatic event such as suicide of a loved one." (p.418)	Caregiving and Parenting<br>Long-term Support Groups and Professional Support<br>Mental Health Advocacy Activities	Productivity<br>Self-Care	Change Freq./Method of Engag.<br>New Engagement	Activities to Manage and Process Loss<br>Activities to Move Forward	Lee, E., Kim, S. won, & Enright, R. D. (2019). Beyond Grief and Survival: Posttraumatic Growth Through Immediate Family Suicide Loss in South Korea. *OMEGA - Journal of Death and Dying, 79*(4), 414-435. https://doi.org/10.1177/0030222817724700

Authors	Publication Year, Country/Countries Data was Collected In, Type of Evidence, Methodology, Study Design, Sample Size of Bereaved Individuals, Recruited Age Range, Sex, Gender, Time Since Loss Range or Mean, Relationship with Lost One(s)	Aim of Study (verbatim)	Specific Activities of Everyday Living Discussed	Activities of Everyday Living Discussed	Engagement Status Discussed	Meanings Associated Discussed	Reference
Lee Y et al.	**Publication Year:** 2023<br>**Country/Countries Data was Collected In:** South Korea<br>**Methodology:** Quantitative<br>**Study Design:** Unspecified Quantitative<br>**Sample Size of Bereaved Individuals:** 42<br>**Recruited Age Range:** Unspecified<br>**Sex:** 76.2% female<br>**Gender**: Identified as mothers (76.2%) and fathers (23%)<br>**Time Since Loss (Range or Mean):** Unspecified<br>**Relationship with Lost One(s):** Child	"This study aims to evaluate suicidal ideation, depression, and insomnia in surviving parents and examine the psychological factors related to the deceased adolescents using psychological autopsy data from Korea." (p. 2)	Caregiving and Parenting<br>Everyday Activities (General)<br>Sleep	Productivity<br>Self-Care	Change Freq./Method of Engag.	Activities to Survive Loss	Lee, Y. J., Kweon, Y. S., & Hong, H. J. (2023). Suicidal Ideation, Depression, and Insomnia in Parent Survivors of Suicide: Based on Korean Psychological Autopsy of Adolescent Suicides. *Journal of Korean Medical Science*, *38*(5), e39–e39. https://doi.org/10.3346/jkms.2023.38.e39
Leichtentritt et al.	**Publication Year:** 2018<br>**Country/Countries Data was Collected In:** Israel<br>**Methodology:** Qualitative<br>**Study Design:** Constructivist-Narrative Approach<br>**Sample Size of Bereaved Individuals:** 12<br>**Recruited Age Range:** 32-54<br>**Sex:** 100% female<br>**Gender**: Unspecified<br>**Time Since Loss (Range or Mean):** Unspecified<br>**Relationship with Lost One(s):** Child	"We set out to examine and to reveal, for the first time, the mothering experiences of bereaved women survivors of maternal suicide. We wanted to provide the participants with the opportunity to talk about their own experiences. Our primary and central research question was: What can the narratives of mothers whose own mothers suicided (when their daughters were children or adolescents) tell us about the mothering experiences of the daughter-survivor?" (p.552)	Caregiving and Parenting<br>Taking on Roles of Family Members, Change in Family Life	Productivity	Change Freq./Method of Engag.	Activities to Manage and Process Loss	Leichtentritt, R. D., Leichtentritt, J., & Mahat Shamir, M. (2018). Shadows from the past: The mothering experience of women survivors of maternal suicide. *Journal of Social Work, 18*(5), 548-577. https://doi.org/10.1177/1468017316656091
Leichtentritt et al.	**Publication Year:** 2015<br>**Country/Countries Data was Collected In:** Israel<br>**Methodology:** Qualitative<br>**Study Design:** Narrative Design<br>**Sample Size of Bereaved Individuals:** 9<br>**Recruited Age Range:** 29-63<br>**Sex:** 66% female<br>**Gender**: Unspecified<br>**Time Since Loss (Range or Mean):** 5-7 years<br>**Relationship with Lost One(s):** Sibling	"This study is the first to examine the characteristics of the ongoing bond maintained by bereaved Israeli sibling survivors of suicide with their deceased<br>brother or sister." (p.1104)	Driving<br>Routines and Rituals Related to Lost one	Self-Care	Change Freq./Method of Engag.<br>New Engagement	Activities to Move Forward	Leichtentritt, R. D., Yerushalmi, A., & Barak, A. (2015). Characteristics of the Ongoing Bond. *British Journal of Social Work*, *45*(4), 1102–1118. https://doi.org/10.1093/bjsw/bct171
Machado & Swank	**Publication Year:** 2019<br>**Country/Countries Data was Collected In:** United States<br>**Methodology:** Qualitative<br>**Study Design:** Case Study<br>**Sample Size of Bereaved Individuals:** 1<br>**Recruited Age Range:** 55-55<br>**Sex:** 100% female<br>**Gender**: Idenfied as women (100%)<br>**Time Since Loss (Range or Mean):** Unspecified<br>**Relationship with Lost One(s):** Child	"To demonstrate the model in clinical practice, the authors present the following case study in which a counselor incorporates the survivor-sensitive therapeutic gardening model within individual counseling." (p.631)	Gardening<br>Long-term Support Groups and Professional Support<br>Sleep<br>Travelling<br>Work	Leisure<br>Productivity<br>Self-Care	Change Freq./Method of Engag.<br>New Engagement	Activities to Survive Loss<br>Activities to Manage and Process Loss<br>Activities to Move Forward	Machado, M. M., & Swank, J. M. (2019). Therapeutic gardening: A counseling approach for bereavement from suicide. *Death Studies*, *43*(10), 629–633. https://doi.org/10.1080/07481187.2018.1509908
Maple et al.	**Publication Year:** 2013<br>**Country/Countries Data was Collected In:** Australia<br>**Methodology:** Qualitative<br>**Study Design:** Narrative Inquiry<br>**Sample Size of Bereaved Individuals:** 22<br>**Recruited Age Range:** Unspecified<br>**Sex:** 73% female<br>**Gender**: Unspecified<br>**Time Since Loss (Range or Mean):** 0.5-20 years<br>**Relationship with Lost One(s):** Child	""I would like to hear about your experience of losing a young adult child to suicide. You can tell your story in any way you feel comfortable, perhaps beginning with telling me a bit about before [child's name]'s death, and then your journey since the suicide."" This statement was directly derived from the aims of the study, which were to explore the ways in which parents live through and with the suicide death of their child." (p.59)	Everyday Activities (General)<br>Gardening<br>Peer Group Facilitation<br>Routines and Rituals Related to Lost one	Leisure<br>Productivity	Disengagement<br>New engagement	Activities to Survive Loss<br>Activities to Move Forward	Maple, M., Edwards, H. E., Minichiello, V., & Plummer, D. (2013). Still part of the family: The importance of physical, emotional and spiritual memorial places and spaces for parents bereaved through the suicide death of their son or daughter. *Mortality, 18*(1), 54–71. https://doi.org/10.1080/13576275.2012.755158

Authors	Publication Year, Country/Countries Data was Collected In, Type of Evidence, Methodology, Study Design, Sample Size of Bereaved Individuals, Recruited Age Range, Sex, Gender, Time Since Loss Range or Mean, Relationship with Lost One(s)	Aim of Study (verbatim)	Specific Activities of Everyday Living Discussed	Activities of Everyday Living Discussed	Engagement Status Discussed	Meanings Associated Discussed	Reference
Marek & Oexle	**Publication Year:** 2024<br>**Country/Countries Data was Collected In:** Germany<br>**Methodology:** Qualitative<br>**Study Design:** Unspecified Qualitative: Online Qualitative Interviews<br>**Sample Size of Bereaved Individuals:** 18<br>**Recruited Age Range:** 23 - 64<br>**Sex:** 50% female<br>**Gender:** Identified as women (50%) and men (50%)<br>**Time Since Loss (Range or Mean):** 0.5 - 25 years<br>**Relationship with Lost One(s):** Sibling, Parent, Child, Extended Family Member (uncle/grandfather/godfather), Friend, Partner/Spouse	"Extending our focus beyond the immediate social circles of SLS to their broader social environments, this study aims to deepen our understanding of their social experiences and how these influence their perceptions of social support... Through qualitative interviews, we aim to identify characteristics that define both supportive and non-supportive social experiences of SLS. This approach will allow us to better understand the social dynamics and uncover facilitators and barriers of social support in the context of suicide bereavement, guiding the development of targeted interventions to enhance social support for SLS." (p. 2-3)	Long-term Support Groups and Professional Support<br>Mental Health Advocacy Activities<br>Work	Productivity<br>Self-Care	Change Freq./Method of Engag.<br>New Engagement	Activities to Manage and Process Loss<br>Activities to Move Forward	Marek, F., & Oexle, N. (2024). Supportive and non-supportive social experiences following suicide loss: a qualitative study. *BMC Public Health*, *24*(1), 1190–13. https://doi.org/10.1186/s12889-024-18545-3
McDaniel et al.	**Publication Year:** 2022<br>**Country/Countries Data was Collected In:** United States<br>**Methodology:** Qualitative<br>**Study Design:** Qualitative Descriptive Study<br>**Sample Size of Bereaved Individuals:** 28<br>**Recruited Age Range:** 20-66<br>**Sex:** 75% female<br>**Gender:** Unspecified<br>**Time Since Loss (Range or Mean):** 0.5-44 years<br>**Relationship with Lost One(s):** Child, Extended Family Member (Cousin), Friend, Parent, Partner/Spouse, Sibling	"To enhance the understanding of the experiences, a qualitative descriptive study was employed. In-depth interviews with suicide loss survivors living in the rural and urban areas were used to answer the question of: "What are the experiences of loved ones following a loss by suicide?" (p. 1492)	Mental Health Advocacy Activities<br>School or Studies<br>Taking on Roles of Family Members, Change in Family Life<br>Work	Productivity	Change Freq./Method of Engag.<br>New Engagement	Activities to Manage and Process Loss<br>Activities to Move Forward	McDaniel, B. M., Daly, P., Pacheco, C. L., & Crist, J. D. (2022). Experiences With Suicide Loss: A Qualitative Study. *Clinical Nursing Research*, *31*(8), 1491–1499. https://doi.org/10.1177/10547738221119344
Mead	**Publication Year:** 2020<br>**Country/Countries Data was Collected In:** United States<br>**Methodology:** Qualitative<br>**Study Design:** Qualitative Group Case Study<br>**Sample Size of Bereaved Individuals:** 150<br>**Recruited Age Range:** 18-25<br>**Sex:** 76% female<br>**Gender:** Unspecified<br>**Time Since Loss (Range or Mean):** Unspecified<br>**Relationship with Lost One(s):** Extended Family Member (Cousins, In-Laws), Friend, Parent, Partner/Spouse, Sibling, Undisclosed	"This qualitative group case study was ascertained to conceptualize theory on the topic of grief and emerging adulthood. A literature review proved no research to this specific experience. The aim of this study was to understand the suicide grief experience in this developmental stage." (p.155)	Alcohol and Drug Use, Risky and Sexual Behaviours<br>School or Studies<br>Taking on Roles of Family Members, Change in Family Life<br>Work	Productivity<br>Self-Care	Change Freq./Method of Engag.<br>Disengagement<br>Re-engagement	Activities to Survive Loss<br>Activities to Manage and Process Loss<br>Activities to Move Forward	Mead, J. (2020). Competing Developmental Demands Among Suicide-Bereaved Emerging Adults. *Omega: Journal of Death and Dying*, *81*(1), 155–169. https://doi.org/10.1177/0030222818764528
Michaud-Dumont et al.	**Publication Year:** 2020<br>**Country/Countries Data was Collected In:** Canada<br>**Methodology:** Qualitative<br>**Study Design:** Qualitative Pilot Study<br>**Sample Size of Bereaved Individuals:** 3<br>**Recruited Age Range:** 20-65<br>**Sex:** 67% female<br>**Gender:** Unspecified<br>**Time Since Loss (Range or Mean):** 2-15 years<br>**Relationship with Lost One(s):** Extended Family Member (Great Aunt), Grandparent, Parent	"The objective of this pilot study was to assess the feasibility of a qualitative research on the psychosocial experience of adults bereaved by the suicide of an elderly relative." (p.1)	Caregiving and Parenting<br>Eating<br>Reading (General)<br>Reading (To Heal)<br>School or Studies<br>Self-Care (General)<br>Tai Chi<br>Work	Leisure<br>Productivity<br>Self-Care	Change Freq./Method of Engag.<br>Disengagement<br>New Engagement	Activities to Survive Loss<br>Activities to Move Forward	Michaud-Dumont, G., Lapierre, S., & Viau-Quesnel, C. (2020). The Experience of Adults Bereaved by the Suicide of a Close Elderly Relative: A Qualitative Pilot Study. *Frontiers in Psychology*, *11*, 2331–2331. https://doi.org/10.3389/fpsyg.2020.538678
Mirick & Berkowitz	**Publication Year:** 2023<br>**Country/Countries Data was Collected In:** United States<br>**Methodology:** Qualitative<br>**Study Design:** Unspecified Qualitative: Semi-structured Interviews<br>**Sample Size of Bereaved Individuals:** 13<br>**Recruited Age Range:** 18 - 26<br>**Sex:** Unspecified<br>**Gender:** Unspecified<br>**Time Since Loss (Range or Mean):** 1.5 - 10 years (mean: 5 years)<br>**Relationship with Lost One(s):** Peer	"This research project aims to fill this gap, exploring the meaning-making process of adolescents following a peer's suicide death." (p. 339)	Caregiving and Parenting<br>Mental Health Advocacy Activities<br>Religious Activities<br>School or Studies	Productivity<br>Self-Care	Change Freq./Method of Engag.<br>Disengagement<br>New Engagement	Activities to Survive Loss<br>Activities to Manage and Process Loss<br>Activities to Move Forward	Mirick, R. G. & Berkowitz, L. (2023) After a Suicide Death in a High School: Exploring Students' Perspectives, *Journal of Social Work in End-of-Life & Palliative Care*, *19*(4), 336-353. doi: 10.1080/15524256.2023.2256481

Authors	Publication Year, Country/Countries Data was Collected In, Type of Evidence, Methodology, Study Design, Sample Size of Bereaved Individuals, Recruited Age Range, Sex, Gender, Time Since Loss Range or Mean, Relationship with Lost One(s)	Aim of Study (verbatim)	Specific Activities of Everyday Living Discussed	Activities of Everyday Living Discussed	Engagement Status Discussed	Meanings Associated Discussed	Reference
Mirick & Berkowitz	**Publication Year:** 2023<br>**Country/Countries Data was Collected In:** United States<br>**Methodology:** Mixed Methods<br>**Study Design:** Sequential Mixed-methods Design<br>**Sample Size of Bereaved Individuals:** 47<br>**Recruited Age Range:** 18 - 26<br>**Sex:** 76.50% female<br>**Gender**: Identified as female (76.5%), non-binary (11.8%), male (2.9%), and undisclosed (8.8%)<br>**Time Since Loss (Range or Mean):** 1.5 - 10 years (mean: 5.31 years)<br>**Relationship with Lost One(s):** Friend, Acquaintance, Peer/Classmate	"The current study builds on these few previous studies, seeking to better understand the ways social media is used and the impact of social media following a suicide death." (p. 4)	Internet Sites and Social Media	Leisure	Change Freq./Method of Engag.	Activities to Survive Loss<br>Activities to Manage and Process Loss<br>Activities to Move Forward	Mirick, R. G., & Berkowitz, L. (2023). "Their Facebook posts were going viral": Social media in the aftermath of an adolescent suicide. *School Social Work Journal*, *48*(1), 1–18.
Mirick & Berkowitz	**Publication Year:** 2023<br>**Country/Countries Data was Collected In:** United States<br>**Methodology:** Mixed Methods<br>**Study Design:** Exploratory Mixed-methods Design<br>**Sample Size of Bereaved Individuals:** 40<br>**Recruited Age Range:** 18 - 28<br>**Sex:** 62.50% female<br>**Gender**: Identified as female (62.5%), male (2.5%), non-binary / queer (10%) and prefer not to say (25%)<br>**Time Since Loss (Range or Mean):** 1 - 10 years<br>**Relationship with Lost One(s):** Friend, Peer	" This project explored this topic, examining self-reported student reactions to the death, school responses, and students' perspectives on the acceptability and helpfulness of these responses." (p. 234)	Everyday Activities (General)<br>School or Studies	Productivity	Change Freq./Method of Engag.<br>Disengagement	Activities to Survive Loss<br>Activities to Manage and Process Loss	Mirick, R. G., & Berkowitz, L. (2023). School-Based Postvention Services: Exploring the Perspectives of Students. *Children & Schools*, *45*(4), 233–242. https://doi.org/10.1093/cs/cdad020
Morrissey et al.	**Publication Year:** 2024<br>**Country/Countries Data was Collected In:** Denmark / Ireland<br>**Methodology:** Qualitative<br>**Study Design:** Qualitative Descriptive<br>**Sample Size of Bereaved Individuals:** 27<br>**Recruited Age Range:** 32 - 80<br>**Sex:** 70% female<br>**Gender**: Unspecified<br>**Time Since Loss (Range or Mean):** 0.5 - 40 years<br>**Relationship with Lost One(s):** Child, Partner/Spouse, Sibling	The previous paper from the study identified how the peer support groups offered "alternative," "transformative" and "conflicted" spaces for belonging and participating, which aided people recovery process (Hybholt et al., 2022) the researchers believed that the micro-processes needed to be covered in more detail as presented in this paper. (p.2)	Long-term Support Groups and Professional Support	Self-Care	New Engagement	Activities to Manage and Process Loss	Morrissey, J., Higgins, A., Buus, N., Berring, L. L., Connolly, T., & Hybholt, L. (2024). The gift of peer understanding and suicide bereavement support groups: A qualitative study. *Death Studies*, 1–12. https://doi.org/10.1080/07481187.2024.2378354
Murray-Swank	**Publication Year:** 2019<br>**Country/Countries Data was Collected In:** United States<br>**Methodology:** Qualitative<br>**Study Design:** Unspecified Qualitative<br>**Sample Size of Bereaved Individuals:** 1<br>**Recruited Age Range:** Unspecified<br>**Sex:** 0% female<br>**Gender**: Unspecified<br>**Time Since Loss (Range or Mean):** Unspecified<br>**Relationship with Lost One(s):** Close family member	"The goal of this article is to share my experience and process of transformation as a family member survivor and psychotherapist after experiencing the loss of a close family member to suicide." (p.189)	Work	Productivity	Change Freq./Method of Engag.	Activities to Move Forward	Murray-Swank, A. B. (2019). The Cracks Where the Light Gets In: Exploring Therapist Transformation Following the Loss of a Family Member to Suicide. *Journal of Psychotherapy Integration*, *29*(2), 188–196. https://doi.org/10.1037/int0000147
Nolan	**Publication Year:** 2020<br>**Country/Countries Data was Collected In:** United States<br>**Methodology:** Mixed Methods<br>**Study Design:** Case Study - Descriptive Mixed Methods<br>**Sample Size of Bereaved Individuals:** 1<br>**Recruited Age Range:** 36-36<br>**Sex:** 0% female<br>**Gender**: 100% transgender<br>**Time Since Loss (Range or Mean):** 5 years<br>**Relationship with Lost One(s):** Partner/Spouse	"To this end, the purpose of this case study was to identify how the bereaved partner of a TGNC person described his experience of bereavement and to understand what effect this experience might have had on his interpersonal relationships and subsequent partnerships." (p.522)	Alcohol and Drug Use, Risky and Sexual Behaviours<br>Religious Activities<br>Sleep<br>Social Activities (General)<br>Work	Leisure<br>Productivity<br>Self-Care	Change Freq./Method of Engag.<br>Disengagement	Activities to Survive Loss	Nolan, R. D. (2020). Transgender and gender non-conforming bereavement (TGNC): A case study on complicated grief experienced and the effect of partner suicide on interpersonal relationships and subsequent partnerships of the bereaved. *Death Studies*, *44*(8), 521–530. https://doi.org/10.1080/07481187.2019.1586796
O'Connell et al.	**Publication Year:** 2024<br>**Country/Countries Data was Collected In:** Death Studies<br>**Methodology:** Qualitative<br>**Study Design:** Unspecified Qualitative: Semi-structured Interviews and Thematic Analysis<br>**Sample Size of Bereaved Individuals:** 12<br>**Recruited Age Range:** Unspecified<br>**Sex:** 83% female<br>**Gender**: Identified as men (17%) and women (83%)<br>**Time Since Loss (Range or Mean):** Unspecified<br>**Relationship with Lost One(s):** Parent, Sibling, Child, Ex-partner/spouse, Partner/Spouse, Friend	"The aim of this study was to explore individuals' experiences of participating in peer-facilitated support groups for suicide bereavement." (p.177)	Long-term Support Groups and Professional Support<br>Taking on Roles of Family Members, Change in Family Life	Productivity<br>Self-Care	Change Freq./Method of Engag.<br>New Engagement	Activities to Manage and Process Loss<br>Activities to Move Forward	O'Connell, S., Troya, M. I., Arensman, E., & Griffin, E. (2023). "That feeling of solidarity and not being alone is incredibly, incredibly healing": A qualitative study of participating in suicide bereavement peer support groups. *Death Studies*, *48*(2), 176–186. https://doi.org/10.1080/07481187.2023.2201922

Authors	Publication Year, Country/Countries Data was Collected In, Type of Evidence, Methodology, Study Design, Sample Size of Bereaved Individuals, Recruited Age Range, Sex, Gender, Time Since Loss Range or Mean, Relationship with Lost One(s)	Aim of Study (verbatim)	Specific Activities of Everyday Living Discussed	Activities of Everyday Living Discussed	Engagement Status Discussed	Meanings Associated Discussed	Reference
Okami et al.	**Country/Countries Data was Collected In:** Japan<br>**Methodology:** Quantitative<br>**Study Design:** Case Report<br>**Sample Size of Bereaved Individuals:** 1<br>**Recruited Age Range:** 27 - 27<br>**Sex:** 100% female<br>**Gender:** Identified as women (100%)<br>**Time Since Loss (Range or Mean):** 5 years<br>**Relationship with Lost One(s):** Sibling	"Herein, we present a patient with PGD who was diagnosed with PDD during IPT sessions for PGD, thereby resulting in improvement in PGD and PDD symptoms." (p.2)	Social Activities (General)<br>Work	Leisure<br>Productivity	Change Freq./Method of Engag.<br>New Engagement	Activities to Survive Loss<br>Activities to Manage and Process Loss	Okami, T., Toshishige, Y., Kondo, M., Okazaki, J., Mizushima, H., & Akechi, T.. (2023). Interpersonal psychotherapy for comorbid prolonged grief disorder and persistent depressive disorder in a Japanese patient: A case report. *Psychiatry and Clinical Neurosciences Reports, 2*(4). https://doi.org/10.1002/pcn5.161
Oulanova et al.	**Publication Year:** 2014<br>**Country/Countries Data was Collected In:** Canada, United States<br>**Methodology:** Qualitative<br>**Study Design:** Interpretative Phenomenological Design<br>**Sample Size of Bereaved Individuals:** 15<br>**Recruited Age Range:** Unspecified<br>**Sex:** 80% female<br>**Gender:** Unspecified<br>**Time Since Loss (Range or Mean):** Unspecified<br>**Relationship with Lost One(s):** Unspecified	"Our research aimed to address this gap in knowledge by examining the lived experiences of suicide survivors who support other individuals bereaved through suicide to understand how they conceptualize their volunteer work, and whether their volunteering in any way affects their own healing from their loss." (p.153)	Peer Group Facilitation<br>Work	Productivity	Disengagement<br>New Engagement	Activities to Survive Loss<br>Activities to Move Forward	Oulanova, O., Moodley, R., & Séguin, M. (2014). From Suicide Survivor to Peer Counselor: Breaking the Silence of Suicide Bereavement. *Omega: Journal of Death and Dying, 69*(2), 151–168. https://doi.org/10.2190/OM.69.2.d
Peters et al.	**Publication Year:** 2016<br>**Country/Countries Data was Collected In:** Australia<br>**Methodology:** Qualitative<br>**Study Design:** Qualitative Narrative Design<br>**Sample Size of Bereaved Individuals:** 10<br>**Recruited Age Range:** Unspecified<br>**Sex:** 70% female<br>**Gender:** Unspecified<br>**Time Since Loss (Range or Mean):** 2-20 years<br>**Relationship with Lost One(s):** Child, Extended Family Member (Uncle), Partner/Spouse	"This current paper explores what participants perceived as helpful and/<br>or unhelpful during interactions with services, family and<br>friends after a suicide death of a family member." (p.419)	Long-term Support Groups and Professional Support	Self-Care	New Engagement	Activities to Manage and Process Loss	Peters, K., Cunningham, C., Murphy, G., & Jackson, D. (2016). Helpful and unhelpful responses after suicide: Experiences of bereaved family members. *International Journal of Mental Health Nursing, 25*(5), 418–425. https://doi.org/10.1111/inm.12224
Peterson et al.	**Publication Year:** 2024<br>**Country/Countries Data was Collected In:** United States<br>**Methodology:** Quantitative<br>**Study Design:** Latent Profile Analysis<br>**Sample Size of Bereaved Individuals:** 2570<br>**Recruited Age Range:** 18 - 88<br>**Sex:** 82.10% female<br>**Gender:** Identified as female (17.2%), male (82.1%), transgender (0.4%), and other (0.1%)<br>**Time Since Loss (Range or Mean):** Unspecified<br>**Relationship with Lost One(s):** Unspecified	The purpose of the present study was to address these critical knowledge gaps by (1) deriving distinct profiles of suicide-exposed SMVs via a comprehensive list of theoretically and empirically based suicide risk factors (Ringer et al., 2018) from a large sample collected across the United States, and (2) investigating the clinical utility of these classes by examining associations with suicidal thoughts and behaviors. (p.67)	Alcohol and Drug Use, Risky and Sexual Behaviours<br>Sleep	Self-Care	Change Freq./Method of Engag.		Peterson, A., Chen, J., Bozzay, M., Bender, A., & Chu, C. (2024). Suicide risk profiles among service members and veterans exposed to suicide. *Journal of Clinical Psychology, 80*(1), 65–85. https://doi.org/10.1002/jclp.23592
Pettersen et al.	**Publication Year:** 2015<br>**Country/Countries Data was Collected In:** Sweden<br>**Methodology:** Qualitative<br>**Study Design:** Content Analysis<br>**Sample Size of Bereaved Individuals:** 18<br>**Recruited Age Range:** 15-38<br>**Sex:** 72% female<br>**Gender:** Unspecified<br>**Time Since Loss (Range or Mean):** 2-17 years<br>**Relationship with Lost One(s):** Sibling	"To explore the subjective experiences that underlie help- seeking, we chose a qualitative approach in this study, the aim of which was to increase the understanding of (a) suicide-bereaved siblings' reported reasons for seek- ing or not seeking professional support, (b) factors determining their reported satisfaction or dissatisfaction with the help received, and (c) suicide-bereaved siblings' experience-based recommendations to health providers." (p.324)	Long-term Support Groups and Professional Support<br>Sleep<br>Taking the Transit<br>Work	Productivity<br>Self-Care	Change Freq./Method of Engag.<br>New Engagement	Activities to Survive Loss	Pettersen, R., Omerov, P., Steineck, G., Dyregrov, A., Titelman, D., Dyregrov, K., & Nyberg, U. (2015). Suicide-Bereaved Siblings' Perception of Health Services. *Death Studies, 39*(6), 323–331. https://doi.org/10.1080/07481187.2014.946624
Pitman et al.	**Publication Year:** 2018<br>**Country/Countries Data was Collected In:** United Kingdom<br>**Methodology:** Qualitative<br>**Study Design:** Qualitative Cross-sectional Study Design and Online Survey<br>**Sample Size of Bereaved Individuals:** 420<br>**Recruited Age Range:** 20-30<br>**Sex:** 83% female<br>**Gender:** Unspecified<br>**Time Since Loss (Range or Mean):** 1-8 years<br>**Relationship with Lost One(s):** Extended family member (Uncle/Aunt/Niece/Nephew/Cousin), Ex-partner, Friend, Grandparent, In-law, Parent, Partner/Spouse, Sibling, Undisclosed	"Our objective was to elicit the views of a national sample of young adults bereaved by the suicide of a close friend or relative, to explore the nature of their experiences of bereavement support and their suggestions regarding appropriate support provision." (p.2)	Caregiving and Parenting<br>Long-term Support Groups and Professional Support<br>School or Studies<br>Taking on Roles of Family Members, Change in Family Life	Productivity<br>Self-Care	Change Freq./Method of Engag.<br>Continued Engagement<br>Disengagement	Activities to Survive Loss<br>Activities to Manage and Process Loss	Pitman, A., De Souza, T., Khrisna Putri, A., Stevenson, F., King, M., Osborn, D., & Morant, N. (2018). Support Needs and Experiences of People Bereaved by Suicide: Qualitative Findings from a Cross-Sectional British Study of Bereaved Young Adults. *International Journal of Environmental Research and Public Health, 15*(4), 666-. https://doi.org/10.3390/ijerph15040666

Authors	Publication Year, Country/Countries Data was Collected In, Type of Evidence, Methodology, Study Design, Sample Size of Bereaved Individuals, Recruited Age Range, Sex, Gender, Time Since Loss Range or Mean, Relationship with Lost One(s)	Aim of Study (verbatim)	Specific Activities of Everyday Living Discussed	Activities of Everyday Living Discussed	Engagement Status Discussed	Meanings Associated Discussed	Reference
Pitman et al.	**Publication Year:** 2018<br>**Country/Countries Data was Collected In:** United Kingdom<br>**Methodology:** Qualitative<br>**Study Design:** Qualitative Cross-sectional Study Design and Online Survey<br>**Sample Size of Bereaved Individuals:** 460<br>**Recruited Age Range:** 18-40<br>**Sex:** 83% female<br>**Gender:** Unspecified<br>**Time Since Loss (Range or Mean):** 1-7 years<br>**Relationship with Lost One(s):** Extended family member (Uncle/Aunt/Niece/Nephew/Cousin), Ex-partner, Friend, Grandparent, In-law, Parent, Partner/Spouse, Sibling, Undisclosed	"Our aim was to elicit the views of a national sample of young adults bereaved by the suicide of a close friend or relative on whether and how the bereavement had affected their educational or work performance. In analyzing their responses, our objective was to identify key themes in their experiences, whether positive or negative, and infer from this how occupational support might be improved." (p. 2-3)	Eating<br>School or Studies<br>Taking on Roles of Family Members, Change in Family Life<br>Work	Productivity<br>Self-Care	Change Freq./Method of Engag.<br>Disengagement<br>New Engagement	Activities to Survive Loss<br>Activities to Manage and Process Loss<br>Activities to Move Forward	Pitman, A., Khrisna Putri, A., De Souza, T., Stevenson, F., King, M., Osborn, D., & Morant, N. (2018). The Impact of Suicide Bereavement on Educational and Occupational Functioning: A Qualitative Study of 460 Bereaved Adults. *International Journal of Environmental Research and Public Health, 15*(4), 643-. https://doi.org/10.3390/ijerph15040643
Powell & Matthys	**Publication Year:** 2013<br>**Country/Countries Data was Collected In:** Canada, United States<br>**Methodology:** Qualitative<br>**Study Design:** Interpretative Phenomenological Design<br>**Sample Size of Bereaved Individuals:** 45<br>**Recruited Age Range:** 20-75<br>**Sex:** 84% female<br>**Gender**: Unspecified<br>**Time Since Loss (Range or Mean):** 0.17-24 years<br>**Relationship with Lost One(s):** Sibling	"To further understand sibling survivor's loss, the goals of this study are to (a) provide insight into sibling suicide survivors' loss, and (b) better understand sibling survivor's responses to their uncertainty and unresolved grief. The following research questions explore sibling suicide survivors' uncertainty and loss." (p.325)	Long-term Support Groups and Professional Support<br>Mental Health Advocacy Activities<br>Work	Productivity<br>Self-Care	New Engagement	Activities to Manage and Process Loss<br>Activities to Move Forward	Powell, K. A., & Matthys, A. (2013). Effects of Suicide on Siblings: Uncertainty and the Grief Process. *Journal of Family Communication*, *13*(4), 321–339. https://doi.org/10.1080/15267431.2013.823431
Praetorius & Rivedal	**Publication Year:** 2020<br>**Country/Countries Data was Collected In:** United States<br>**Methodology:** Qualitative<br>**Study Design:** Qualitative Group Case Study<br>**Sample Size of Bereaved Individuals:** 6<br>**Recruited Age Range:** Unspecified<br>**Sex:** 84% female<br>**Gender**: Unspecified<br>**Time Since Loss (Range or Mean):** Unspecified<br>**Relationship with Lost One(s):** Child, Extended Family Member (Uncle), Friend, Partner/Spouse, Sibling	"The purpose of this study was to add to the emergent literature by analyzing already published, naturally occurring, stories of six SOS to answer the follow- ing question: What can we learn from the experiences of these storytellers bereaved by suicide about the grieving process?" (p.349)	Alcohol and Drug Use, Risky and Sexual Behaviours<br>Attending Conferences<br>Housework, Chores<br>Long-term Support Groups and Professional Support<br>Mental Health Advocacy Activities<br>Music, Radio, TV<br>Peer Group Facilitation<br>Reading (To Heal)<br>School or Studies<br>Self-Care (General)	Leisure<br>Productivity<br>Self-Care	Change Freq./Method of Engag.<br>New Engagement<br>Re-engagement	Activities to Survive Loss<br>Activities to Manage and Process Loss<br>Activities to Move Forward	Praetorius, R. T., & Rivedal, J. (2020). Navigating Out of the Ocean of "Why"—A Qualitative Study of the Trajectory of Suicide Bereavement. *Illness, Crisis, and Loss, 28*(4), 347–362. https://doi.org/10.1177/1054137317741714
Quayle et al.	**Publication Year:** 2023<br>**Country/Countries Data was Collected In:** United Kingdom<br>**Methodology:** Qualitative<br>**Study Design:** Unspecified Qualitative: Semi-structured Interviews and Thematic Analysis<br>**Sample Size of Bereaved Individuals:** 18<br>**Recruited Age Range:** 18 - 85<br>**Sex:** 83.30% female<br>**Gender**: Unspecified<br>**Time Since Loss (Range or Mean):** 0.5 - 23 years<br>**Relationship with Lost One(s):** Parent, Child, Sibling, Partner/Spouse, Grandchild, Ex-partner/Spouse, Friend, Other	In this study we aimed to investigate the phenomenon of intrusive mental imagery after suicide bereavement in a community sample by analysing data from interviews exploring individual experiences of such imagery following the suicide of a close contact. (p.2)	Everyday Activities (General)<br>Internet Sites and Social Media<br>Sleep<br>Work	Leisure<br>Productivity<br>Self-Care	Change Freq./Method of Engag.<br>New Engagement<br>Re-engagement	Activities to Survive Loss<br>Activities to Move Forward	Quayle, K., Jones, P., Di Simplicio, M., Kamboj, S., & Pitman, A.. (2023). Exploring the phenomenon of intrusive mental imagery after suicide bereavement: A qualitative interview study in a British sample. *PLOS One, 18*(8), e0284897. https://doi.org/10.1371/journal.pone.0284897
Ratnarajah et al.	**Publication Year:** 2014<br>**Country/Countries Data was Collected In:** Australia<br>**Methodology:** Qualitative<br>**Study Design:** Unspecified Qualitative: Semi-structured Interviews<br>**Sample Size of Bereaved Individuals:** 18<br>**Recruited Age Range:** Unspecified<br>**Sex:** Unspecified<br>**Gender**: Unspecified<br>**Time Since Loss (Range or Mean):** Unspecified<br>**Relationship with Lost One(s):** Child, Parent, Partner/Spouse, Sibling	"The aim of this study was to explore the recollections of adults who had experienced the suicide death of a family member at some time in their lives. The purpose was to provide the opportunity for the participants to speak of their meaning making endeavors in coming to understand the context in which the suicide occurred within their family. The research question guiding this study was: How do individuals within suicide-bereaved families narrate their experience of loss in the context of familial relationships?" (p.44)	Long-term Support Groups and Professional Support<br>Religious Activities<br>Taking on Roles of Family Members, Change in Family Life	Productivity<br>Self-Care	Change Freq./Method of Engag.<br>New Engagement	Activities to Manage and Process Loss<br>Activities to Move Forward	Ratnarajah, D., Maple, M., & Minichiello, V. (2014). Understanding Family Member Suicide Narratives by Investigating Family History. *Omega: Journal of Death and Dying, 69*(1), 41–57. https://doi.org/10.2190/OM.69.1.c
Rivart et al.	**Publication Year:** 2021<br>**Country/Countries Data was Collected In:** United Kingdom<br>**Methodology:** Qualitative<br>**Study Design:** Exploratory Study<br>**Sample Size of Bereaved Individuals:** 227<br>**Recruited Age Range:** 18-72<br>**Sex:** 77% female<br>**Gender**: Unspecified<br>**Time Since Loss (Range or Mean):** Unspecified<br>**Relationship with Lost One(s):** Child, Friend, Extended Family Member (aunt/uncle, grandparent, cousin), Parent, Partner/Spouse, Sibling	"This study aimed to address these gaps in knowledge by exploring the experiences of individuals bereaved by suicide in people from a minority ethnic background. Ultimately, furthering our knowledge has the potential to inform research and practice in the suicide bereavement field with regards to ethnic minorities in the UK, notably to support the development of specialized postvention services if required." (p.2)	Alcohol and Drug Use, Risky and Sexual Behaviours	Self-Care	Change Freq./Method of Engag.	Activities to Survive Loss	Rivart, P., Wainwright, V., Flynn, S., Hunt, I. M., Shaw, J., Smith, S., McGale, B., & McDonnell, S. (2021). Ethnic Minority Groups' Experiences of Suicide Bereavement: A Qualitative Exploratory Study. *International Journal of Environmental Research and Public Health, 18*(22), 11860-. https://doi.org/10.3390/ijerph182211860

Authors	Publication Year, Country/Countries Data was Collected In, Type of Evidence, Methodology, Study Design, Sample Size of Bereaved Individuals, Recruited Age Range, Sex, Gender, Time Since Loss Range or Mean, Relationship with Lost One(s)	Aim of Study (verbatim)	Specific Activities of Everyday Living Discussed	Activities of Everyday Living Discussed	Engagement Status Discussed	Meanings Associated Discussed	Reference
Rogerson & Carson	**Publication Year:** 2017<br>**Country/Countries Data was Collected In:** United Kingdom<br>**Methodology:** Qualitative<br>**Study Design:** Narrative Case Study<br>**Sample Size of Bereaved Individuals:** 1<br>**Recruited Age Range:** Unspecified<br>**Sex:** 100% female<br>**Gender**: Unspecified<br>**Time Since Loss (Range or Mean):** Unspecified<br>**Relationship with Lost One(s):** Parent	"The purpose of this paper is to provide a profile of Suzanne Rogerson." (p.208)	Activities Related to Creation of a New Life<br>Long-term Support Groups and Professional Support<br>Sleep	Self-Care	Change Freq./Method of Engag.<br>New Engagement	Activities to Survive Loss<br>Activities to Manage and Process Loss	Rogerson, S., & Carson, J. (2017). Remarkable lives: Suzanne Rogerson in conversation with Jerome Carson. *Mental Health and Social Inclusion, 21*(4), 208–212. https://doi.org/10.1108/MHSI-06-2017-0026
Roitman	**Publication Year:** 2021<br>**Country/Countries Data was Collected In:** Israel<br>**Methodology:** Qualitative<br>**Study Design:** Clinical Case Study<br>**Sample Size of Bereaved Individuals:** 1<br>**Recruited Age Range:** 12-12<br>**Sex:** 0% female<br>**Gender**: Unspecified<br>**Time Since Loss (Range or Mean):** Unspecified<br>**Relationship with Lost One(s):** Sibling	"In this work, I have aimed to elucidate Winnicott's principle of 'mutual playing' with relation to seriously traumatised children who cannot creatively express their experience through play" (p.413)	Arts and Crafts<br>School or Studies<br>Social Activities (General)	Leisure<br>Productivity	Change Freq./Method of Engag.<br>Disengagement<br>New Engagement	Activities to Survive Loss	Roitman, Y. (2021). A play for a bereaved brother: mutually playing with the child survivor of a sibling suicide. *Journal of Child Psychotherapy, 47*(3), 402–414. https://doi.org/10.1080/0075417X.2021.2015797
Ross et al.	**Publication Year:** 2021<br>**Country/Countries Data was Collected In:** Australia<br>**Methodology:** Qualitative<br>**Study Design:** Focus Groups<br>**Sample Size of Bereaved Individuals:** 15<br>**Recruited Age Range:** 31-73<br>**Sex:** 80% female<br>**Gender**: Unspecified<br>**Time Since Loss (Range or Mean):** 1.25-5 years<br>**Relationship with Lost One(s):** Family Member, Partner/Spouse	"This study aimed to qualitatively examine the experiences and support needs of the suicide-bereaved with the goal of informing future postvention efforts." (p.2)	Caregiving and Parenting<br>Internet Sites and Social Media<br>Long-term Support Groups and Professional Support	Leisure<br>Productivity<br>Self-Care	Change Freq./Method of Engag.<br>New Engagement	Activities to Survive Loss<br>Activities to Manage and Process Loss	Ross, V., Kõlves, K., & De Leo, D. (2021). Exploring the Support Needs of People Bereaved by Suicide: A Qualitative Study. Omega: *Journal of Death and Dying, 82*(4), 632–645. https://doi.org/10.1177/0030222819825775
Ross et al.	**Publication Year:** 2018<br>**Country/Countries Data was Collected In:** Australia<br>**Methodology:** Mixed Methods<br>**Study Design:** Unspecified Mixed Methods: Semi-structured Interviews<br>**Sample Size of Bereaved Individuals:** 14<br>**Recruited Age Range:** 50-78<br>**Sex:** 50% female<br>**Gender**: Unspecified<br>**Time Since Loss (Range or Mean):** 0.5-1 years<br>**Relationship with Lost One(s):** Child	"The current study aims to examine the individual experiences of both mothers and fathers bereaved by suicide over time, specifically at the six month and 12 month time points after the death of their child." (p.2)	Alcohol and Drug Use, Risky and Sexual Behaviours<br>Camping<br>Driving<br>Long-term Support Groups and Professional Support<br>Mental Health Advocacy Activities<br>Religious Activities<br>Routines and Rituals Related to Lost one<br>Sleep<br>Social Activities (General)<br>Travelling<br>Volunteer work (General)<br>Walking<br>Work<br>Writing (Journaling, Letter Writing)	Leisure<br>Productivity<br>Self-Care	Change Freq./Method of Engag.<br>Continued Engagement<br>New Engagement<br>Re-engagement	Activities to Survive Loss<br>Activities to Manage and Process Loss<br>Activities to Move Forward	Ross, V., Kõlves, K., Kunde, L., & De Leo, D. (2018). Parents' Experiences of Suicide-Bereavement: A Qualitative Study at 6 and 12 Months after Loss. *International Journal of Environmental Research and Public Health, 15*(4), 618-. https://doi.org/10.3390/ijerph15040618
Salom et al.	**Publication Year:** 2024<br>**Country/Countries Data was Collected In:** Spain<br>**Methodology:** Qualitative<br>**Study Design:** Semi-structured Individual Interviews<br>**Sample Size of Bereaved Individuals:** 10<br>**Recruited Age Range:** 26 - 65<br>**Sex:** 70% female<br>**Gender**: Unspecified<br>**Time Since Loss (Range or Mean):** 3 - 33 years (mean: 14 years)<br>**Relationship with Lost One(s):** Partner/Spouse, Sibling, Child, Parent	"The main objective of this research was to extend this effort by exploring the experiences of Spanish mourners after losing a loved one by suicide<br>by coding those meaning categories that played an important role in their process of meaning reconstruction of loss. (p. 89)"	Attending Conferences<br>Meditation<br>Routines and Rituals Related to Lost One<br>School or Studies<br>Work	Leisure<br>Productivity<br>Self-Care	Disengagement<br>New Engagement	Activities to Survive Loss<br>Activities to Manage and Process Loss<br>Activities to Move Forward	Salom, R., Layrón, J. E., Neimeyer, R. A., & Pérez, S. (2024). Construction of meaning in survivors of suicide loss: A Spanish translation and application of the Meaning in Loss Codebook. *Death Studies, 49*(1), 87–99. https://doi.org/10.1080/07481187.2024.2438412
Sanford et al.	**Publication Year:** 2023<br>**Country/Countries Data was Collected In:** Australia<br>**Methodology:** Mixed methods<br>**Study Design:** Cross-sectional Study Design and Online Survey<br>**Sample Size of Bereaved Individuals:** 50<br>**Recruited Age Range:** 19 - 74<br>**Sex:** 80% female<br>**Time Since Loss (Mean):** 12.1 years<br>**Gender**: Unspecified<br>**Relationship with Lost One(s):** Parent, Friend, Other non-family (e.g. community members, colleagues, etc.), Friend of a friend	This study aims to contribute to the existing literature on impact to suicide exposure by exploring the following research questions:<br>· What are the reasons for or meanings associated with low closeness among people highly impacted by a suicide death?<br>· How do people with low closeness to the person who died and high impact resulting from the death describe their experience of exposure to suicide? (p. 3)	Caregiving and Parenting<br>Driving<br>Long-term Support Groups and Professional Support<br>Work	Productivity<br>Self-Care	Change Freq./Method of Engag.<br>Disengagement<br>New Engagement	Activities to Survive Loss<br>Activities to Manage and Process Loss<br>Activities to Move Forward	Sanford, R. L., Frey, L. M., Thind, N., Butcher, B., & Maple, M. (2023). Unpacking the meaning of closeness, reconsidering the concept of impact in suicide exposure, and expanding beyond bereavement: "Just, I hope you don't forget about us." *OMEGA: Journal of Death and Dying, 0*(0), 1–29. https://doi.org/10.1177/00302228231196616

Authors	Publication Year, Country/Countries Data was Collected In, Type of Evidence, Methodology, Study Design, Sample Size of Bereaved Individuals, Recruited Age Range, Sex, Gender, Time Since Loss Range or Mean, Relationship with Lost One(s)	Aim of Study (verbatim)	Specific Activities of Everyday Living Discussed	Activities of Everyday Living Discussed	Engagement Status Discussed	Meanings Associated Discussed	Reference
Sanford et al.	**Publication Year:** 2016<br>**Country/Countries Data was Collected In:** Australia, United States<br>**Methodology:** Mixed Methods<br>**Study Design:** Mixed Methods Survey Design<br>**Sample Size of Bereaved Individuals:** 197<br>**Recruited Age Range:** 19-75<br>**Sex:** 12% female<br>**Gender**: Unspecified<br>**Time Since Loss (Range or Mean):** Range: unspecified-40 years, Mean: 6.01 years<br>**Relationship with Lost One(s):** Child, Parent, Partner/Spouse, Sibling	"The aims of this study were to: (1) provide a description of loss survivors who sought therapy after the loss; and (2) examine overall perceptions of therapy helpfulness and individual, therapy, and therapist related factors to perceived benefit." (p.552)	Long-term Support Groups and Professional Support	Self-Care	New Engagement		Sanford, R., Cerel, J., McGann, V., & Maple, M. (2016). Suicide Loss Survivors' Experiences with Therapy: Implications for Clinical Practice. *Community Mental Health Journal, 52*(5), 551–558. https://doi.org/10.1007/s10597-016-0006-6
Schotanus-Dijkstra et al.	**Publication Year:** 2014<br>**Country/Countries Data was Collected In:** Netherlands<br>**Methodology:** Qualitative<br>**Study Design:** Content Analysis<br>**Sample Size of Bereaved Individuals:** 165<br>**Recruited Age Range:** 14-63<br>**Sex:** 70% female<br>**Time Since Loss (Range or Mean):** Unspecified<br>**Gender**: Unspecified<br>**Relationship with Lost One(s):** Child, Friend, Parent, Partner, Sibling, Unknown, Other	"The aim of this article was to examine self-help mechanisms, grief reactions, and experiences with health-care services communicated in online forums for the bereaved by suicide." (p.33)	Arts and Crafts<br>Long-term Support Groups and Professional Support<br>Music, Radio, TV<br>Writing (Journaling, Letter Writing)	Leisure<br>Self-Care	New Engagement	Activities to Manage and Process Loss	Schotanus-Dijkstra, M., Havinga, P., van Ballegooijen, W., Delfosse, L., Mokkenstorm, J., & Boon, B. (2014). What do the bereaved by suicide communicate in online support groups? A content analysis. *Crisis: The Journal of Crisis Intervention & Suicide Prevention, 35*(1), 27–35. https://doi.org/10.1027/0227-5910/a000225
Shields et al.	**Publication Year:** 2019<br>**Country/Countries Data was Collected In:** Ireland<br>**Methodology:** Qualitative<br>**Study Design:** Interpretative Phenomenological Design<br>**Sample Size of Bereaved Individuals:** 4<br>**Recruited Age Range:** 45-60<br>**Sex:** 0% female<br>**Gender**: Unspecified<br>**Time Since Loss (Range or Mean):** 0.58-3 years<br>**Relationship with Lost One(s):** Parent	"The current study aims to examine the experiences of mothers who have been bereaved by suicide and to explore how mothers bereaved by suicide make sense of the event within the context of support groups." (p.179)	Caregiving and Parenting<br>Long-term Support Groups and Professional Support<br>Reading (To Heal)<br>Regular Visits to Mediums<br>Social Activities (General)	Leisure<br>Productivity<br>Self-Care	Change Freq./Method of Engag.<br>Continued Engagement<br>New Engagement	Activities to Survive Loss<br>Activities to Manage and Process Loss<br>Activities to Move Forward	Shields, C., Russo, K., & Kavanagh, M. (2019). Angels of Courage: The Experiences of Mothers Who Have Been Bereaved by Suicide. *Omega: Journal of Death & Dying, 80*(2), 175–201. https://doi.org/10.1177/0030222817725180
Silvén Hagström	**Publication Year:** 2021<br>**Country/Countries Data was Collected In:** Sweden<br>**Methodology:** Qualitative<br>**Study Design:** Narrative Inquiry<br>**Sample Size of Bereaved Individuals:** 22<br>**Recruited Age Range:** 6-unspecified<br>**Sex:** 68% female<br>**Gender**: Unspecified<br>**Time Since Loss (Range or Mean):** 0.5-5 years<br>**Relationship with Lost One(s):** Parent	"This narrative evaluation of a grief support camp for families affected by a parent's suicide arranged by the non-profit organization Children's Rights in Society in Sweden investigates whether children [N = 11] and parents [N = 11] perceived their participation as meaningful and, if so, in what way, and the changes to which the program was said to have contributed." (p.1)	Arts and Crafts<br>Housework, Chores<br>Long-term Support Groups and Professional Support<br>Music, Radio, TV	Leisure<br>Productivity<br>Self-Care	Change Freq./Method of Engag.<br>Continued Engagement<br>New Engagement	Activities to Survive Loss<br>Activities to Manage and Process Loss	Silvén Hagström, A. (2021). A Narrative Evaluation of a Grief Support Camp for Families Affected by a Parent's Suicide. *Frontiers in Psychiatry, 12*, 783066–783066. https://doi.org/10.3389/fpsyt.2021.783066
Silvén Hagström	**Publication Year:** 2019<br>**Country/Countries Data was Collected In:** Sweden<br>**Methodology:** Qualitative<br>**Study Design:** Narrative Design<br>**Sample Size of Bereaved Individuals:** 4<br>**Recruited Age Range:** Unspecified<br>**Sex:** 100% female<br>**Gender**: Unspecified<br>**Time Since Loss (Range or Mean):** Unspecified<br>**Relationship with Lost One(s):** Parent	"The aim of the article is to discuss the practical implications of this research from a social constructivist grief perspective and to introduce methods to support a "meaning-searching approach" in interventions with parentally suicide-bereaved young people" (p.114)	Routines and Rituals Related to Lost one		New Engagement	Activities to Move Forward	Silvén Hagström, A. (2019). "Why did he choose to die?": A meaning-searching approach to parental suicide bereavement in youth. *Death Studies, 43*(2), 113–121. https://doi.org/10.1080/07481187.2018.1457604
Silvén Hagström	**Publication Year:** 2017<br>**Country/Countries Data was Collected In:** Sweden<br>**Methodology:** Qualitative<br>**Study Design:** Unspecified Qualitative<br>**Sample Size of Bereaved Individuals:** Not Applicable<br>**Recruited Age Range:** Unspecified<br>**Sex:** Not Specified<br>**Gender**: Unspecified<br>**Time Since Loss (Range or Mean):** Unspecified<br>**Relationship with Lost One(s):** Parent	"The overall aim of this article is to investigate how a net community can offer social support with grieving." (p.777)	Internet Sites and Social Media	Leisure	New Engagement	Activities to Move Forward	Silvén Hagström, A. (2017). 'Suicide stigma' renegotiated: Storytelling, social support and resistance in an Internet-based community for the young suicide-bereaved. *Qualitative Social Work : QSW : Research and Practice, 16*(6), 775–792. https://doi.org/10.1177/1473325016644039

Authors	Publication Year, Country/Countries Data was Collected In, Type of Evidence, Methodology, Study Design, Sample Size of Bereaved Individuals, Recruited Age Range, Sex, Gender, Time Since Loss Range or Mean, Relationship with Lost One(s)	Aim of Study (verbatim)	Specific Activities of Everyday Living Discussed	Activities of Everyday Living Discussed	Engagement Status Discussed	Meanings Associated Discussed	Reference
Spillane et al.	**Publication Year:** 2018<br>**Country/Countries Data was Collected In:** Ireland<br>**Methodology:** Mixed Methods<br>**Study Design:** Case-control Study<br>**Sample Size of Bereaved Individuals:** 18<br>**Recruited Age Range:** 18-unspecified<br>**Sex:** 39% female<br>**Gender:** Unspecified<br>**Time Since Loss (Range or Mean):** 1.25-3.17 years<br>**Relationship with Lost One(s):** Unspecified	"The aim of this study was to examine how family members have been physically and psychologically affected following suicide bereavement. A secondary objective of the study was to describe the needs of family members bereaved by suicide" (p.1)	Alcohol and Drug Use, Risky and Sexual Behaviours<br>Dancing<br>Eating<br>Gardening<br>Music, Radio, TV<br>Photography<br>Physical Activity<br>Sleep<br>Social Activities (General)<br>Walking<br>Work<br>Yoga	Leisure<br>Productivity<br>Self-Care	Change Freq./Method of Engag.<br>Disengagement<br>New Engagement	Activities to Survive Loss<br>Activities to Manage and Process Loss<br>Activities to Move Forward	Spillane A., Matvienko-Sikar K., Larkin C., Corcoran P., & Arensman E. (2018). What are the physical and psychological health effects of suicide bereavement on family members? An observational and interview mixed-methods study in Ireland. *BMJ Open*, *8*(1), e019472. https://doi.org/10.1136/bmjopen-2017-019472
Stewart & Thomas	**Publication Year:** 2020<br>**Country/Countries Data was Collected In:** United Kingdom<br>**Methodology:** Qualitative<br>**Study Design:** Heuristic Inquiry<br>**Sample Size of Bereaved Individuals:** 3<br>**Recruited Age Range:** Unspecified<br>**Sex:** 100% female<br>**Gender:** Unspecified<br>**Time Since Loss (Range or Mean):** Unspecified<br>**Relationship with Lost One(s):** Parent	"This heuristic inquiry aims to explore the lived experiences of trainee psychotherapists bereaved by maternal suicide. It examines the lived experiences of the researcher and two participants, with data collected through interviews, dreams and journalling." (p.552)	Work	Productivity	Change Freq./Method of Engag.	Activities to Survive Loss	Stewart, K., & Thomas, V. (2020). A trainee psychotherapist's heuristic exploration of losing a mother to suicide. *British Journal of Guidance & Counselling*, *48*(4), 552–562. https://doi.org/10.1080/03069885.2018.1519181
Stirling	**Publication Year:** 2016<br>**Country/Countries Data was Collected In:** United Kingdom<br>**Methodology:** Qualitative<br>**Study Design:** Autoethnographical Exploration<br>**Sample Size of Bereaved Individuals:** 1<br>**Recruited Age Range:** Unspecified<br>**Sex:** 100% female<br>**Gender:** Unspecified<br>**Time Since Loss (Range or Mean):** Unspecified<br>**Relationship with Lost One(s):** Friend	"For the purpose of this study, I began to engage with regular yoga practice for the first time in my life. This continued for 4 months, and I built a collection of experiential notes throughout. I took part in various style of yoga, across several locations, to gather an eclectic array of practice. I wrote about each session as fully as I could. Even at times of injury, wrote about the impact of not doing yoga. I also wrote about life outside of yoga, in order to make connections with how the practice was affecting me in real ways, and to establish macro- and microlinkages (Laslett, 1999)." (p.281)	Meditation<br>Writing (Journaling, Letter Writing)<br>Yoga	Leisure<br>Self-Care	New Engagement	Activities to Survive Loss	Stirling, F. J. (2016). Yoga and Loss: An Autoethnographical Exploration of Grief, Mind, and Body. *Illness, Crisis, and Loss*, *24*(4), 279–291. https://doi.org/10.1177/1054137316659396
Strouse et al.	**Publication Year:** 2021<br>**Country/Countries Data was Collected In:** United Kingdom<br>**Methodology:** Mixed Methods<br>**Study Design:** Pre-test Post-test Single Group Design<br>**Sample Size of Bereaved Individuals:** 39<br>**Recruited Age Range:** Unspecified<br>**Sex:** 87% female<br>**Gender:** Unspecified<br>**Time Since Loss (Mean):** 3.92 years<br>**Relationship with Lost One(s):** Child, Parent, Sibling	"The purpose of this mixed methods study was to evaluate the Artful Grief Studio (AGS) at the 2019 Tragedy Assistance Program for Survivors (TAPS) National Suicide Survivors Conference" (p.1)	Arts and Crafts<br>Long-term Support Groups and Professional Support	Leisure<br>Self-Care	New Engagement	Activities to Move Forward	Strouse, S., Hass-Cohen, N., & Bokoch, R. (2021). Benefits of an open art studio to military suicide survivors. *The Arts in Psychotherapy*, *72*, 101722-. https://doi.org/10.1016/j.aip.2020.101722
Sugrue et al.	**Publication Year:** 2014<br>**Country/Countries Data was Collected In:** Ireland<br>**Methodology:** Qualitative<br>**Study Design:** Exploratory Study<br>**Sample Size of Bereaved Individuals:** 7<br>**Recruited Age Range:** 50-79<br>**Sex:** 100% female<br>**Gender:** Unspecified<br>**Time Since Loss (Range or Mean):** 2-5 years<br>**Relationship with Lost One(s):** Child	"The aim of this study was to explore the bereavement experience of mothers following their child's death by suicide." (p.118)	Alcohol and Drug Use, Risky and Sexual Behaviours<br>Caregiving and Parenting<br>Everyday Activities (General)	Productivity<br>Self-Care	Change Freq./Method of Engag.<br>Disengagement	Activities to Survive Loss<br>Activities to Manage and Process Loss	Sugrue, J. L., McGilloway, S., & Keegan, O. (2014). The experiences of mothers bereaved by suicide: an exploratory study. *Death studies*, *38*(1-5), 118–124. https://doi.org/10.1080/07481187.2012.738765
Supiano et al.	**Publication Year:** 2017<br>**Country/Countries Data was Collected In:** United States<br>**Methodology:** Quantitative<br>**Study Design:** Randomized Controlled Attention Wait-list Clinical Trial<br>**Sample Size of Bereaved Individuals:** 21<br>**Recruited Age Range:** 34-73<br>**Sex:** 29% female<br>**Gender:** Unspecified<br>**Time Since Loss (Range or Mean):** Unspecified<br>**Relationship with Lost One(s):** Unspecified	"In this study, we chose to examine the therapeutic process of grief change in participants in an ongoing randomized controlled trial evaluating the impact of CGGT on clinical outcomes of suicide survivors experiencing CG. We examined therapeutic change processes through the lens of meaning reconstruction theory and meaning making (Neimeyer, 2001a, 2001b, 2001c) in participants in CGGT for suicide survivors." (p.554)	Eating<br>Long-term Support Groups and Professional Support<br>Sleep	Self-Care	Change Freq./Method of Engag.<br>New Engagement	Activities to Survive Loss<br>Activities to Manage and Process Loss	Supiano, K. P., Haynes, L. B., & Pond, V. (2017). The transformation of the meaning of death in complicated grief group therapy for survivors of suicide: A treatment process analysis using the meaning of loss codebook. *Death Studies*, *41*(9), 553–561. https://doi.org/10.1080/07481187.2017.1320339

Authors	Publication Year, Country/Countries Data was Collected In, Type of Evidence, Methodology, Study Design, Sample Size of Bereaved Individuals, Recruited Age Range, Sex, Gender, Time Since Loss Range or Mean, Relationship with Lost One(s)	Aim of Study (verbatim)	Specific Activities of Everyday Living Discussed	Activities of Everyday Living Discussed	Engagement Status Discussed	Meanings Associated Discussed	Reference
Tosini & Fraccaro	**Publication Year:** 2022<br>**Country/Countries Data was Collected In:** Italy<br>**Methodology:** Qualitative<br>**Study Design:** Unspecified Qualitative: Semi structured Interviews<br>**Sample Size of Bereaved Individuals:** 25<br>**Recruited Age Range:** 30-69<br>**Sex:** 16% female<br>**Gender:** Unspecified<br>**Time Since Loss (Range or Mean):** Unspecified<br>**Relationship with Lost One(s):** Cousin, Niece, Parent, Partner/Spouse, Sibling, Sister-in-law, Uncle	"We aimed to understand how survivors perceived and handled suicide events, how their identity changed, and what difficulties they encountered in their social circles. Our additional goal was to identify how the survivors' involvement in mutual aid groups influenced their grieving process, with our main focus on the stories of participants in a suicide-specific group." (p.987)	Long-term Support Groups and Professional Support<br>Routines and Rituals Related to Lost one<br>Volunteer work (Advocacy)	Productivity<br>Self-Care	New Engagement	Activities to Survive Loss<br>Activities to Manage and Process Loss<br>Activities to Move Forward	Tosini, D., & Fraccaro, D. (2022). "Like climbing a glass wall": Suicide survivors in an Italian province. *Death Studies*, *46*(4), 987–995. https://doi.org/10.1080/07481187.2020.1795746
Turner et al.	**Publication Year:** 2019<br>**Country/Countries Data was Collected In:** United States<br>**Methodology:** Mixed Methods<br>**Study Design:** Narrative Mixed Methods Design<br>**Sample Size of Bereaved Individuals:** 8<br>**Recruited Age Range:** Unspecified<br>**Sex:** 88% female<br>**Gender:** Unspecified<br>**Time Since Loss (Range or Mean):** 0.75-20 years<br>**Relationship with Lost One(s):** Unspecified	"The purpose of this study was to determine if Integral Breath Therapy (IBT) is an appropriate intervention for facilitating meaning-making out of a traumatic loss and reducing symptoms of complicated grief for individuals bereaved by suicide." (p.425)	Alcohol and Drug Use, Risky and Sexual Behaviours<br>Exercise<br>Long-term Support Groups and Professional Support<br>Religious Activities<br>Self-Care (General)<br>Sleep<br>Social Activities (General)<br>Volunteer work (Advocacy)<br>Work	Leisure<br>Productivity<br>Self-Care	Change Freq./Method of Engag.<br>Disengagement<br>New Engagement<br>Re-engagement	Activities to Survive Loss<br>Activities to Manage and Process Loss<br>Activities to Move Forward	Turner, R., Wooten, H. R., & Chou, W.-M. (2019). Changing Suicide Bereavement Narrative Through Integral Breath Therapy. *Journal of Creativity in Mental Health*, *14*(4), 424–435. https://doi.org/10.1080/15401383.2019.1625839
Wardle	**Publication Year:** 2024<br>**Country/Countries Data was Collected In:** Scotland<br>**Methodology:** Qualitative<br>**Study Design:** Unspecified: Semi-structured Interviews and Narrative Inquiry<br>**Sample Size of Bereaved Individuals:** 1<br>**Recruited Age Range:** 6 - 6<br>**Sex:** 0% female<br>**Gender:** Unspecified<br>**Time Since Loss (Range or Mean):** Unspecified<br>**Relationship with Lost One(s):** Relative	"This paper will focus on outdoor art therapy sessions with a child who will be referred to as Alex. This paper will focus on the impact of taking the sessions outdoors and nature based art therapy. (p. 175)"	Long-term Support Groups and Professional Support<br>Routines and Rituals Related to Lost One<br>School or Studies	Productivity<br>Self-Care	Change Freq./Method of Engag.<br>New Engagement	Activities to Survive Loss<br>Activities to Manage and Process Loss<br>Activities to Move Forward	Wardle, A. (2024). Landscape of loss: Art therapy outdoors and traumatic bereavement. *International Journal of Art Therapy*, *29*(3), 174–180. https://doi.org/10.1080/17454832.2023.2267109
Watson et al.	**Publication Year:** 2021<br>**Country/Countries Data was Collected In:** United States<br>**Methodology:** Qualitative<br>**Study Design:** Collective Case Study Design<br>**Sample Size of Bereaved Individuals:** 7<br>**Recruited Age Range:** Unspecified<br>**Sex:** 57% female<br>**Gender:** Unspecified<br>**Time Since Loss (Range or Mean):** Unspecified<br>**Relationship with Lost One(s):** Parent	"Noting these needs, the primary purpose of this study was to explore how adults who, as very young children, lost a father to suicide thereafter perceived their father's suicide, and obtain their recommendations regarding children's literature (picture books) that may or may not support young CSoPS. More specifically, the goal of this study was to lay the foundations for gathering information that will inform interventions, including potential bibliotherapy-based intervention [7], to support young CSoPS." (p.4)	Alcohol and Drug Use, Risky and Sexual Behaviours<br>Reading (To Heal)<br>School or Studies	Leisure<br>Productivity<br>Self-Care	Change Freq./Method of Engag.<br>New Engagement<br>Re-engagement	Activities to Survive Loss<br>Activities to Manage and Process Loss	Watson, C., Cutrer-Párraga, E. A., Heath, M., Miller, E. E., Young, T. A., & Wilson, S. (2021). Very Young Child Survivors' Perceptions of Their Father's Suicide: Exploring Bibliotherapy as Postvention Support. *International Journal of Environmental Research and Public Health*, *18*(21), 11384-. https://doi.org/10.3390/ijerph182111384
Williams & Gubi	**Publication Year:** 2024<br>**Country/Countries Data was Collected In:** England / Wales<br>**Methodology:** Qualitative<br>**Study Design:** Semi-structured Interviews<br>**Sample Size of Bereaved Individuals:** 4<br>**Recruited Age Range:** 10 - 19<br>**Sex:** 100% female<br>**Gender:** Unspecified<br>**Time Since Loss (Range or Mean):** Unspecified<br>**Relationship with Lost One(s):** Parent	The aim of the research was to ascertain if, and how, paternal suicide, in a young woman's adolescence, affects her intimate, heterosexual relationships, in adulthood. (p. 659)	Alcohol and Drug Use, Risky and Sexual Behaviours<br>Long-term Support Groups and Professional Support	Self-Care	Change Freq./Method of Engag.<br>New Engagement	Activities to Survive Loss<br>Activities to Manage and Process Loss	Williams, S. A., & Gubi, P. M. (2024). The self-perceived impact of parental suicide in adolescence, of a father, on his daughter's intimate, heterosexual relationships in adulthood. *Illness, Crisis & Loss, 32*(4), 659–676. https://doi.org/10.1177/10541373231186608
Zavrou et al.	**Publication Year:** 2023<br>**Country/Countries Data was Collected In:** Cyprus<br>**Methodology:** Qualitative<br>**Study Design:** Unspecified Qualitative: Semi-structured Interviews and Interpretive Phenomenological Analysis<br>**Sample Size of Bereaved Individuals:** 12<br>**Recruited Age Range:** 45 - 63<br>**Sex:** 17% female<br>**Gender:** Unspecified<br>**Time Since Loss (Range or Mean):** 3 - 19 years<br>**Relationship with Lost One(s):** Child	The aim of the present study was to explore the experiences of Greek-speaking Cypriot parents whose child died by suicide, with focus on a) their interpretations of the incidence, b) the impact of suicide on them and their immediate family, and c) the healing strategies and supportive systems used to cope with their loss. (p. 2)	Gardening<br>Music, Radio, TV<br>Religious Activities<br>Routines and Rituals Related to Lost One<br>Sleep<br>Social Activities (General)<br>Swimming<br>Walking<br>Writing (Journaling, Letter Writing)	Leisure<br>Self-Care	Change Freq./Method of Engag.<br>Continued Engagement<br>Disengagement<br>New Engagement	Activities to Survive Loss<br>Activities to Manage and Process Loss	Zavrou, R., Charalambous, A., Papastavrou, E., Koutroubas, A., & Karanikola, M. (2023). Qualitative inquiry into the experience of suicide loss, aftereffects and coping strategies of suicide-bereaved Greek-speaking parents in Cyprus. *International Journal of Qualitative Studies on Health and Well-Being*, *18*, 2265671. https://doi.org/10.1080/17482631.2023.2265671

Authors	Publication Year, Country/Countries Data was Collected In, Type of Evidence, Methodology, Study Design, Sample Size of Bereaved Individuals, Recruited Age Range, Sex, Gender, Time Since Loss Range or Mean, Relationship with Lost One(s)	Aim of Study (verbatim)	Specific Activities of Everyday Living Discussed	Activities of Everyday Living Discussed	Engagement Status Discussed	Meanings Associated Discussed	Reference
Zuromski et al.	**Publication Year:** 2024<br>**Country/Countries Data was Collected In:** United States<br>**Methodology:** Mixed Methods<br>**Study Design:** Structured and Open-ended Questions<br>**Sample Size of Bereaved Individuals:** 168<br>**Recruited Age Range:** Unspecified<br>**Sex:** Unspecified<br>**Gender**: Unspecified<br>**Time Since Loss (Range or Mean):** 0.16 - 0.25 years<br>**Relationship with Lost One(s):** Parent, Sibling, Partner/Spouse	This study was carried out as part of the Army Study to Assess Risk and Resilience in Service members (Army STARRS) (Ursano et al., 2014). We sought to better understand: The risk factors and perceived causes of the suicide, perspectives on suicide prevention efforts, and the impact of the suicide on others. (p. 438)	Long-term Support Groups and Professional Support	Self-Care	New Engagement	Activities to Manage and Process Loss	Zuromski, K. L., Wilks, C. R., Al-Suwaidi, M., Wittler, E., Scherban, L., Hite, B., Raymond, L., Dempsey, C. L., Stein, M. B., Ursano, R. J., Benedek, D., & Nock, M. K. (2024). Perspectives of suicide loss survivors: Qualitative analysis of data from a psychological autopsy study of U.S. Army soldiers. *Suicide and Life-Threatening Behavior, 54*(3), 437–449. https://doi.org/10.1111/sltb.13052
