# Supplementary material for: Engagement in meaningful activities post suicide loss: A scoping review
Source: PLoS One. 2025 Nov 17;20(11):e0336640. doi: 10.1371/journal.pone.0336640 (PMC12622850; doi:10.1371/journal.pone.0336640)
Supplement: S3 Appendix — (PDF) [file pone.0336640.s003.pdf]

S3 Appendix
Data Extraction Table: Direct Quotes for Inductive Content Analysis

Authors	Direct Quotes from Included Studies Related to Meaningful Activities of Everyday Living (the red text was considered the unit of analysis for content analysis)	Reference
Adams et al.	Example of subtheme 1.1 Initial Reactions - "Life interrupted: "I gave myself a certain amount of time and I wrote off the rest of my year at uni." (David)" (p. 326 - Results)<br><br>Example of subtheme 3.1 Relationship with the deceased pre-death - "Memories: "To help remember [him] in a positive way we got a little diary where we would write anecdotal stories about [him], funny things that he did." (Evan)" (p. 326 - Results)<br><br>Example of subtheme 4.2 Growing through grief - "Recognition of personal growth and responsibility to make constructive use of their experience to help others: "I'm a much more sensitive counselor." (Georgia)" (p. 326 - Results)<br><br>"Life was interrupted for all participants. They suspended studies and plans when the shocking news of their sibling's suicide reached them. Several siblings living away from home immediately returned home on receiving a call with the news of their sibling's death. All participants took time out from their lives to comfort and seek comfort from their families. Carol "had a month off work ... and moved home for three or four months ... to be in constant contact" with her mother, and allay her fear that "something may happen" to her mother." (p. 327 - Results)<br><br>"Participants acknowledged the benefit of professional support and accepting help. They expressed relief in being able to talk to trained professionals they did not have to protect, who unlike their parents would not overreact to their raw emotions or suicidal ideation. "I could tell him what I was thinking and he wouldn't get scared. I could say 'I want to be with my brother.' If I said that to Mum she would be distraught" (Carol). Participants had difficulties in finding, engaging, and accepting support services. One participant reported needing postvention support more than 12 months following the suicide, even after initially declining an offer of support" (p. 328 - Results)<br><br>"Participants highlighted the importance and comfort of keeping memories of their deceased sibling alive, with some expressing concern that memories of their deceased sibling were fading. "My memory about [my brother] feels like it's dwindling and that's pretty distressing" (Georgia). Participants described various methods of safekeeping these precious memories, such as creating diaries, Internet sites, and hard copies of images and stories as keepsakes. Participants often lapsed into present tense when speaking of their deceased sibling and were keen to relate their deceased sibling's positive attributes in a manner protective of their memory" (p. 328-329 - Results)<br><br>"Relationship with the deceased post-death included subthemes of responsibility of living for the deceased and spirituality, faith, and psychics. Participants voiced a sense of responsibility to live for sibling; to experience life on behalf of their deceased sibling and in some cases to replace roles once filled by them in the family. Participants often reported using spirituality, faith, and psychics to maintain an ongoing relationship with the deceased person. Two women sought to connect with their deceased brothers with the aid of mediums. One described how she intentionally changed her behavior and became the anchor of the family in line with her belief that her brother expected that of her. She also described how she began to emulate some of his positive qualities, such as his sense of humor and fun." (p. 329 - Results)<br><br>"Most participants were motivated to learn about mental health and suicide, through reading, joining groups, or organizations dedicated to mental health issues." (p. 329 - Results)<br><br>"Participants acknowledged their sense of personal growth and responsibility to make constructive use of their experience to help others. "I feel a lot better myself, it helps me by helping other people ... I have finally got to a place where I can channel it into something constructive." (Bianca) "I would like to think in the future I would be able to help others that have gone through a similar situation." (Evan)." (p. 329 - Results)	Adams, E., Hawgood, J., Bundock, A., & Kõlves, K. (2018). A phenomenological study of siblings bereaved by suicide: A shared experience. *Death studies, 43* (5), 324–332. https://doi.org/10.1080/07481187.2018.1469055
Adshead & Runacvres	"Healthy habits - Creative: Writing, journaling, art; Spiritual: meditation, mindfulness, prayer; Exercise: yoga, running, walking, gym, 5 a side football; Diversifying coping mechanisms; Social connection, sense of community (Table 1, p. 5 - Results)<br><br>""You build relationships when you come to the support group that often and you, I'd pop out and meet them for coffee sometimes" (Chloe)." (p. 8 - Results)<br><br>"Social connection was a top priority to negate emotional darkness replacing them with positive and helpful actions: and "get me out of the house and keep me moving and keep me socialised and give me a sense of normalcy" (Stephanie)." (p. 8 - Results)<br><br>"Attending a support group was just one way participants were managing their grief, instead participants opted to diversify their coping mechanisms in addition to support group attendance including creative hobbies (journaling, art): "Writing to him and getting it out really helps. But definitely talking about it and talking to other people is amazing. [...] I think probably the support group, and writing and art are the three best ones for me" (Stephanie); spiritual (meditation, mindfulness, prayer), physical activity (yoga, running, walking, gym, football), being around others and finding a sense of community: "Generally going to gym classes, going to play five a side football once a week, just being around people just definitely helps in a social setting" (Michael)." (p. 9 - Results)<br><br>"The support group provided meaningful connections and increased self-compassion and self-reliance (outcome). This is emphasised by the following extract: "I don't want to be a burden. [...] But [...] that's what you go to the support group to talk about, that's the only thing you talk about" (Charlotte)." (p. 9 - Results)<br><br>"Men feared interpretations of negative emotions, hoped their feelings would disappear without support, preferring to be seen as tough and brave rather than someone who needs help (mechanism). Social support groups can facilitate in partially reconditioning this response providing a safe space for individuals to express themselves to like-minded individuals providing a sense of release (outcome)." (p. 9 - Results)<br><br>"The group provided relief, connection with others providing a sense of routine, consistency and normalcy (outcome)." (p.10 - Results)<br><br>"by diversify coping mechanisms combining support group attendance with physical, creative and spiritual activities (outcome) providing an opportunity for routine, socialising and improvement in self-esteem and self-acceptance" (p.11 - Results)<br><br>"When assessing what improved the wellbeing of individuals bereaved by suicide, it appears the outcomes are consistent with existing literature. For example, previous research suggests exercise can reduce negative emotion and elevate self-esteem (Abraham et al., 2016; Khalsa & Gould, 2012; Khalsa, 2013; Riley, 2004; Steinberg & Sykes, 1985). Creative activities (Bat-Or & & Garti, 2019; Doka, 1989, 2008; Stroebe & Schut, 1999), spiritual and religious practices were found to be beneficial for processing disenfranchised grief (Doka, 1989, 2008; Stroebe & Schut, 1999); and spiritual and religious practices (Feigelman et al., 2019; Khalsa & Gould, 2012; Khalsa, 2013; Maltby et al., 2008; Muñoz, 2015) were considered beneficial coping mechanisms." (p.12 - Discussion)	Adshead, C., & Runacres, J. (2022). Sharing is Caring: A Realist Evaluation of a Social Support Group for Individuals Who Have Been Bereaved by Suicide. *Omega, 89* (1), 172–190. https://doi.org/10.1177/00302228211070152
Ali, F. & Lucock	"Therefore, participants wanted to talk to others to gain some insights into why their loved one may have died or how they coped and dealt with the death. Participants also perceived stigma from others, which was not the case from other types of bereavement. These experiences culminated in self-isolation for certain participants, further negatively impacting their mental health. Some participants lacked family and social support or wanted to avoid distressing and burdening others, so SOBS was considered a safer space that protected them from the judgement of others. Moreover, participants felt others in their social support networks or community had limited knowledge of suicide bereavement or were unresponsive to their needs." (p.7-8 - Results)<br><br>"'I found SOBS and the Compassionate Friends support people who've lost a child in any way. Sharing helps quite a lot with your grief, because you can talk about it and they can talk about theirs and you swap bits of information and feelings' (Mother whose son died by suicide). The importance of having a diverse range of peer support groups for the bereaved was exemplified by Christine. (p. 8 - Results)<br><br>"Over time, regular long-term members of SOBS learned to accept that they could not take responsibility for the death and this reduced some of the guilt they felt.<br>Participants reported their suicide bereavement did not follow a similar pattern to other bereavements and challenged the assumptive norms of grief and coping. Talking to other attendees at SOBS enabled them to empathise with each other and receive confirmation that<br>every individual's grief was unique. 'I say at SOBS you can laugh about things, where in a group away from SOBS I suppose if you're in a group of people say the hospital or something like that with professionals, if you laughed they'd all look at your like that, what's wrong with this person are they having a nervous breakdown? [...] but sometimes the laugh is a relief because you know somebody's understanding what you're talking about'" (p. 9 - Results)<br><br>"The findings identified the process of transitioning from life before the suicide to life after the suicide as they reconstructed a different social world. Christine stated, "the way your life was, is altered so you have to start doing something else and get a new type of normal". Participants described a changed sense of self and identity and defined themselves as 'survivors' - "because we are 'survivors or bereavement' not 'victims' as such." The reconceptualisation of 'survivor' was empowering for many participants because it challenged the negative societal connotations of the bereaved." (p. 9 - Results)<br><br>"The group took part in shared activities such as lighting candles in memory of the loved ones at Christmas, which became a ritual outside of the group for a few participants. Group members also shared their diverse ways of coping with the suicide, for instance, two participants disclosed visiting mediums after hearing a few members' experiences, although both did not believe in the afterlife." (p. 9-10 - Results)<br><br>"Many participants found their needs were also being met outside the group, for example by drawing on their family and social support networks, continued use of postvention, adopting new hobbies, reading on grief and bereavement or using coping strategies they developed after the death of significant others." (p. 10 - Results)<br><br>"The study also identified reasons for disengagement from SOBS. Some participants found their kinship and nature of the relationship with the decedents were not reflective of the majority of attendees which reduced the sense of commonality, as also reported by Groos and Shakespeare-Finch (2013)." (p. 12 - Discussion)	Ali, F., & Lucock, M. (2020). 'It's like getting a group hug and you can cry there and be yourself and they understand'. Family members' experiences of using a suicide bereavement peer support group. *Bereavement Care, 39* (2), 51–58. https://doi-org.myaccess.library.utoronto.ca/10.1080/02682621.2020.1771951

Ali, U. & Rehna	"Likewise, in an effort to detach themselves from the suicide incident and the resulting stigma, some suicide survivors were more likely to detach themselves from their support networks, relationships and being social. Subsequently, these conditions may prevent survivors of suicide from engaging in research on suicide bereavement" (p. 600 - Results)<br><br>"'Respondent (14M) shared, ''In the morning before work, as my eyes open to know that it is another relentless day, I was always overwhelmed by my restless nights of absolute exhaustion.''' (p. 600 - Results)<br><br>"Respondent (3M) shared, ''I wish to do so much but I could not, as if your arms have been cut off, and is crippled.'' He also reported, ''Every person used to talk which made me get in the habit of skepticism as if these two people are watching and talking about me, and the death of my son.'' Respondent (3F) shared, ''Time has slipped from our hands. From my hands.'' " (p. 601 - Results)<br><br>"One respondent (3M) discussed in detail impact of religion on his grief process. He shared, ''I prayed a lot, prayed for my child's forgiveness,'' ''Religion teaches us a lot, everything is written in our Holy Book,'' ''There is a hope and this hope comes from religion,'' ''This whole world is running because of trusting in God's plans. He is running the system. Humans endure and show patience,'' ''There's strength in prayers. Allah listens to all our prayers and fulfill them at the right time. That is why do not shun prayers for your beloved ones who have left this world.'' " (p. 601 - Results)<br><br>"Respondent (3F) further shared, ''Religion teaches us to have patience during grief. This difficult time comes from Allah and I have read and learned that Allah only chose His noble people to face the challenge.'' "(p. 601 - Results)	Ali, U., & Rehna, T. (2023). Grief Reactions and Suicide Bereavement in the Context of Stigma among Parents: An Interpretative Phenomenological Analysis. *Annales Médico-Psychologiques, 181* (7), 598–603. https://doi.org/10.1016/j.amp.2022.04.016
Azorina et al.	"The majority of codes under this theme described social withdrawal as a negative outcome of their bereavement, relating this to having become more insecure, less outgoing, lacking self-esteem, and having difficulty making friends. Many described avoiding social events, especially in large groups of people, even if they had previously enjoyed these types of social situations or considered themselves to be quite "sociable". A difficult combination of feelings was often described as underlying this process. Despite withdrawing, several people mentioned a simultaneous longing for connection. "I tend to withdraw myself from others (people in general) most of the time whilst at the same time feeling a need to be really close to them."—Eighteen-year-old female whose close friend had died by suicide 5 years prior" (p. 6)<br><br>"A minority of codes under this theme described using withdrawal as a means of coping with grief by making deliberate decisions to withdraw from relationships and spend more time alone. They reported needing more time or space for themselves, often as a way of processing emotions privately." (p. 7)<br><br>"Overprotectiveness towards Others: Those who became more protective of others described the strain of keeping partners or relatives happy or avoiding upsetting them, as part of preventive efforts. By maintaining vigilance for symptoms of depression in close relatives, and lowering their threshold for offering help, they hoped to be able to intervene as early as possible. The greater intensity of these relationships appeared to be associated with quite significant emotional strain, on top of the work of grief. Any additional closeness gained from these relationships with partners or close relatives was often therefore at some cost. "Anxious about making him (partner) happy and making sure he would not get depressed like my uncle."—Twenty-nine-year-old female whose uncle had died by suicide 3 months previously "I am more quick to offer help and comfort when they (partner or potential partners) have depressing feelings."—Twenty-year-old female whose close friend had died by suicide 5 years prior. There was a sense in which this sub-theme applied primarily to family and partners because of a greater sense of responsiblity for their well-being, and because the prospect of their loss would be the most devastating. However, where this sub-theme applied to friendships, there was evidence that respondents were proactive in helping out friends with mental illness, monitoring friends' well-being, and offering help even to acquaintances." (p. 8-9)	Azorina, V., Morant, N., Nesse, H., Stevenson, F., Osborn, D., King, M., & Pitman, A. (2019). The Perceived Impact of Suicide Bereavement on Specific Interpersonal Relationships: A Qualitative Study of Survey Data. *International journal of environmental research and public health, 16* (10), 1801. https://doi.org/10.3390/ijerph16101801
Bailey E et al.	"More than half of the participants (56.8%, n = 126) had spent 2 hr or less using suicide bereavement Internet forums over the past week. One fifth of the participants (20.3%, n = 45) spent between 3 and 5 hr, 29 (13.1%) between 6 and 10 hr, and 22 (9.9%) participants spent more than 10 hr using forums." (p. 396 - Results)<br><br>"The most common activity was reading through conversations without actively contributing (90.0% do this either some or most of the time), although many reported that they also start new threads (80.2%) and reply to other people's posts (80.2%). The least common activity was seeking professional help (80.2% either rarely or never do this)." (p. 396-397 - Results)<br><br>"Participants generally rated Internet forums and Facebook groups as beneficial and there were few perceived limitations, although some individuals did experience more distress than others as a result of forum use. The 24/7 availability of the forums and their ability to reduce feelings of isolation through facilitating interaction with others were the most highly endorsed benefits." (p. 399 - Discussion)	Bailey, E., Krysinska, K., O'Dea, B., & Robinson, J. (2017). Internet Forums for Suicide Bereavement. *Crisis, 38* (6), 393–402. https://doi.org/10.1027/0227-5910/a000471
Bailey L et al.	"An interesting and unexpected finding amongst our sample was that Facebook was by far the most commonly used resource (reported by all but one of our respondents). Some had kept and sustained existing Facebook accounts belonging to the deceased, others had created new pages in memorial to the deceased. Two of our participants had developed their own websites. For all of our participants these resources served as a memorial to their loved one and as a means of bringing people together, gaining support and reaching out to others. Some also used these resources for awareness raising and fundraising. Through their narratives, participants described the various ways in which the Internet has transformed elements of their experience of suicide grief. One common aspect was to keep the deceased's page going as a way of keeping the deceased "alive"." (p. 75 - Findings / Results)<br><br>"Participants in our study talked about how the use of the sites continued beyond the immediate aftermath of the death and how the functions and natureof activity evolved. Firstly, the emotional and therapeutic functions of the site (reaching out, seeking support and bringing people together) expanded as users continued to share memories and messages long after the death had occurred. Secondly, participants used the pages for sharing information, sign posting support and raising awareness. Some, like this mother who lost her son to suicide the previous year, had initiated support forums and incorporated links to suicide prevention or suicide bereavement charity pages and fundraising events: there's always something going on there ... one of his friends is doing a fundraising event ...my sister-in-law did a sponsored walk ... Ben and his friends ... they've got a just giving page and that's already got quite a lot on it ... It's a real forum for everything, everything you think of really ... It's evolving all the time ... for me that was very much a healing process.(OSM014f)<br><br>Parents, in particular, cited the cathartic effect of keeping busy and staying pro-active. For this parent, setting up a stand-alone website in his son's memory enabled him to reach out to others and become involved in suicide prevention efforts. This had knock-on consequences for enabling him to stay connected to his son and manage his own grief in the process: I am passionate about suicide prevention and ... letting people know there is stuff out there but it's also a way of me still being involved in Harry and Harry being involved in my life on a day to day basis and I'm very proud to mention his name and to try and get across the message ... it's given me things to do ... if I needed a moment with Harry I could then do something on the site ... so that helped from that point of view ... [Harry's site] is where I put my energy into if I'm feeling shit ... and, you know ... it's cathartic. (OSM016m)<br><br>For these parents and others, utilising social media for the purposes of charitable work made them feel like they were doing something worthwhile. There was the feeling that even though it was too late for their own child, they were spurred onto prevent the deaths of other young people who may be at-risk. A common refrain was the need make their child's death meaningful, for something good to come out of an otherwise unfathomable and heartbreaking tragedy. This parent described setting up a memorial page on Facebook both in order to remember her son, and thereby continue that connection, and in order to do something for him posthumously: I wanted him to be remembered ... because he ... left so little of himself ... also it's kind of like making up to him ... and saying well I'm going to do this for you Carl ... just to ...acknowledge the pain he went through. (OSM015)" (p. 76 - 77 - Findings / Results)<br><br>"The participants were able to explore their grief with others from the comfort of their own homes. The online environment also served to free up some of the inhibitions that they otherwise felt when talking to strangers and even friends about their loss, affording them an openness that they did not get elsewhere. Some described feeling comforted by the fact that they no longer felt as alone and isolated whilst others felt that talking with other survivors gave them important insights which helped with their grieving process." (p. 77 - Findings / Results)<br><br>"A Facebook page in particular reveals authorship to be an ongoing social process in which many individuals can contribute to the establishment and maintenance of a psychic space. The ever-changing and evolving nature of the deceased's Facebook page proves to be particularly comforting for the bereaved and serves to "keep the deceased alive" " (p. 83 - Discussion)	Bailey, L., Bell, J., & Kennedy, D. (2015). Continuing social presence of the dead: exploring suicide bereavement through online memorialisation. *New Review of Hypermedia and Multimedia, 21* (1–2), 72–86. https://doi-org.myaccess.library.utoronto.ca/10.1080/13614568.2014.983554
Bartik et al.	"Alcohol Use. A total of 12 participants reported drinking alcohol at leastweekly and, on average, consuming the equivalent of seven to nine standard drinks. Six participants recorded drinking in excess of 10 standard drinks every time they drank alcohol. " (p. 485 - Results)<br><br>"Most participants identified alcohol and other drugs to help cope with their grief. Zac (14 years old) said: Alcohol, I drunk [sic] 12 days straight… I was just in my own land of what the fuck do I do now? We do mad parties, and usually we got all smashed off our faces. 'Cause that's what Dan would have wanted. That's the way I see it now, because I lost someone so close, I see every day as my last day. So I live my life as, like, a game I guess… I seen [sic] it as another one gone, it's only going to happen again. I know another one's coming… I'll see probably more than that. I worry about them." (p. 487 - Results)<br><br>"Most participants disregarded their own health, but were focused on the immediate well-being of their close friends, as explained by Kate: I think I put a lot on how other people were dealing with it. Like, I took into account how other people were feeling or might have been feeling, so I made sure that they were okay but as a result I neglected myself sort of thing." (p. 487 - Results)<br><br>"Participants reported concerning levels of alcohol use, less helpful coping skills, elevated levels of depression and anxiety, prolonged grief, and minimal posttraumatic growth." (p. 487 - Discussion)	Bartik, W. J., Maple, M., & McKay, K. (2020). Youth Suicide Bereavement and the Continuum of Risk. *Crisis: The Journal of Crisis Intervention and Suicide Prevention, 41* (6), 483–489. https://doi.org/10.1027/0227-5910/a000653
Bartik et al.	"Risky coping behavior, in several forms, was identified as the third theme within this sample. Alcohol misuse, for example, was common in the immediate period of grieving between the death and the funeral. For most participants, this continued well beyond this initial grief stage, and at dangerous levels of consumption. The problematic use of alcohol was recognized by many, including Angel:<br>Alcohol was a bad thing ... I sort of felt like I was in this vicious cycle where I would try to make myself feel better by drowning my sorrows but I wasn't drowning them, I was bringing them all to the surface and I didn't like that either.<br>Risk-taking behavior to cope with the death extended beyond alcohol to other drugs and to risky sexual behaviors for some, as described by Mae:<br>Greg's death had a really weird effect on everyone, people were doing things they shouldn't have been doing. There was increase in drinking and we were all under age so underage drinking, and smoking, and people were having sex with peo- ple that they shouldn't be and all that sort of stuff, just doing whatever they had to do to get through the days.<br>In some cases, risk-taking behavior in the form of alcohol and drugs became quite serious, leading to self-harm and suicide risk, as Jean described:<br>At that stage I was dabbling with drugs a bit. Once Keith died I did everything I could to not think about it, not feel anything. Just any reason I could think of to take drugs I would ... I ended up being off my head and taking so many drugs that they weren't even working. I would just take anything people were giving me ... I was self-harming.<br>These behaviors, and the risk that was associated with them, were potentially life threatening. Within this sample, most had engaged in these behaviors." (p. 214 - Results)<br><br>"Increased alcohol consumption in the form of binge-drinking, use of other drugs, plus increased and possibly unsafe sexual activity were all raised in the par- ticipants' narratives. While such behavior can often be considered within the realm of normal adolescent devel- opment and experimentation (Muir et al., 2009), for the young person who had experienced the suicide of a friend, these behaviors appeared to become more extreme and potentially harder to control even by their own standards. The results also suggest these risky coping behaviors could continue for a period of years, meaning that these young people were potentially at increased risk for poor health outcomes over a considerably longer period of time as a consequence." (p. 215 - Discussion)	Bartik, W., Maple, M., Edwards, H., & Kiernan, M. (2013). Adolescent survivors after suicide: Australian young people's bereavement narratives. *Crisis: The Journal of Crisis Intervention and Suicide Prevention, 34* (3), 211-217. doi:https://doi-org.myaccess.library.utoronto.ca/10.1027/0227-5910/a000185

Bell et al.	"Nine participants used the online social networking platform 'Facebook' for the purposes of memorialisation. Four participants set up a new profile account after the death of their loved one. Five participants maintained the existing Facebook profile page of the deceased – namely, an online profile that the deceased had set up. Of these five, some had chosen to convert the profile into a memorial page in order to keep the page as it had been left by the deceased. Some participants made visits to the deceased's 'in-life profile' in addition to setting up a memorial site. One participant closed his son's in-life Facebook account immediately after his death, but regretted doing so." (p. 379 - Results)<br>"Consistent with previous research in the area (DeGroot, 2012; Kasket, 2012; Maddrell, 2012; Williams & Merten, 2009), the sites enabled participants to express their grief, to communicate their feelings to family and friends and to connect with other suicide survivors who had unique insights into their suffering. As such, the sites served to bring people together both in the immediate aftermath of the suicide and for many months, sometimes years, after the event." (p. 379 - Results)<br><br>"Julia explains how she and her family derived comfort from reading the page in the immediate aftermath of his death: 'Facebook … enabled us to get together and laugh and … share some nice memories … we could look at things and laugh and cry'. They have maintained the site ever since.<br>Julia's motivation initially was 'to keep him alive, to … still be able to talk to him, his friends' and also so that his friends could keep in touch with him – which they still do, three years on. It was important for Julia to see that others were remembering Mark, continuing to post mementos and visiting him at the site. It was also important for her to attend to the site 'I don't want others to see it's neglected, he's there but he's not there but I just want to keep it nice …'" (p. 379 - 380 - Results)<br><br>"Briany lost her son Michael almost a year before the research interview. She set up a memorial site on Facebook for her family and his friends. Within hours people were posting on it. She estimated that around 120 people visited the site initially. A year after Michael's death, his memorial site is still active with around 60 people continuing to use it consistently and new content posted every few days.<br>Since Michael's death, two of his close friends have subsequently attempted suicide and a number of others have suffered depression. In response to this, Briany posts 'inspirational' material (e.g. supportive messages, quotes, poetry) on his memorial site, hoping that she will reach his friends who are vulnerable. Briany has more recently become involved with suicide prevention charity work via Michael's memorial site in order to fundraise and promote awareness:<br>… it's grown, it's evolving all the time I would say … I would say it's become more of a practical tool, whereas, initially … it was more … led by emotions …" " (p. 380 - Results)<br><br>" For the first month, when the site was highly active, it kept her busy and served as a distraction from grief, reducing her own solitude and isolation. Then Samantha began to notice a gradual decline, which was distressing for her: 'have people forgotten? Don't people care?' She described feeling pressured at this point to add content to keep it going. Five months on and the activity had almost stopped. Samantha herself was reluctant in the face of all this to continue to contribute to the page: 'don't want to put anything in case nobody responds …' She laments I think it served its purpose … to tell people she's died, for people to outlay their initial grief and then for them to know about funeral arrangements … to raise money … now … it … doesn't seem to have a function for anyone …" (p. 381 - Results)<br><br>"Lucinda lost her son Carl approximately two years prior to the research interview. About a year after his death she and one of his friends set up a memorial page on Facebook. She wanted Carl to be remembered and wanted friends and family to post things on it:<br>Because he left so little of himself … also kind of like making up to him … to acknowledge the pain he went through and just to say … I'm doing this for you … just so people know … that he's not forgotten … I … thought people would … post on it … happy memories … that sort of thing … I thought … it'll be as though he's still here … Lucinda also visited Carl's personal Facebook page after his death via his sib- ling's account where she was able to contact his friends. However, she was the only person in her family who accessed both the memorial site and the personal page of her son regularly and was the only one who posted." (p. 381 - Results)	Bell, J., Bailey, L., & Kennedy, D. (2015). 'We do it to keep him alive': bereaved individuals' experiences of online suicide memorials and continuing bonds. *Mortality (Abingdon, England), 20* (4), 375–389. https://doi.org/10.1080/13576275.2015.1083693
Bjornsdottir et al.	"All the parents felt that their psyche and body just collapsed, describing desolation, poor short-term memory, difficulty performing mental tasks, having deep existential, emotional,<br>and physical pain, as well as losing physical endurance, appetite, sleep, balance, and energy. They also described how they had lost touch with reality, all grounding, and were numb for a long time. Mia and William stated that they had no desire for food and could not think about themselves except for essentials: "You were just numb; all kinds of junk were bought for food so that we would eat because we had no appetite." (p.979).<br><br>"Helen said: "This affects the health; it is such a shock." James developed a physical illness and had to undergo an operation following the suicide, and Helen lost all physical endurance. Both described poor short-term memory after the suicide. Isabella was aching all over, had abdominal pain and a persistent knot of anxiety in her stomach, and there was considerable disturbance around her sleep. She stated: "Then I had such fits of crying that just suddenly poured over me. I just cried and cried and cried, and then I got a terrible headache." John was "tearful, sad, and sorrowful."" (p.980)<br><br>"I get an interview with them in the psychiatric ward. I open up, cry my heart out, and tell them the truth, "You know, I think I am losing it; I think I am just wholly losing my senses." You know, I was sleeping maybe three hours a day, and here I could barely stand; I vomited or had diarrhea alternately, could not hold down any food, lost weight rapidly, and was just a shadow of myself." (p.980)<br><br>"The parents felt they needed long-term support and trauma-informed care from health professionals. They felt they had to reach out for help in too many cases but could not. They called for standard operating procedures regarding suicides and suicide attempts. Helen and James became familiar with poor service and lack of support when their son made a severe suicide attempt, which led to hospitalisation in a psychiatric ward with a visit to the emergency room, where they were not offered any trauma-informed services. The parents also called for less expensive psychological services that would ensure that parents would receive long-term professional support following such major trauma. (p.980)	Bjornsdottir, E. A., Sigurdardottir, S., & Halldorsdottir, S. (2024). Excruciating existential suffering and complicated grief: The essence of surviving the suicide of a son or daughter. *Scandinavian Journal of Caring Sciences, 38* (4), 973–983. https://doi.org/10.1111/scs.13289
Black & Heo	"I cannot possibly go back to doing what I was doing before my son died. I don't think I can look at my students and talk about something as mundane as how to properly construct a sentence. I mean my world is not the same – how can it be? I lost my son, my only child. I cannot be the same person I was before. … Once I thought my job was very was very important; now nothing about the job seems that important." (p.8)<br><br>"When Elliott died, I was so lost. It took me several years of thinking and grieving before I could even think of making any kind of meaning in his death. I finally decided that I had to take his death and figure out what I could do to help another family avoid suicide. I am going to establish a nonprofit to assist with community mental health for adolescents and young adults. I don't want Elliott's death to be in vain." (p.9)<br><br>"Somedays it is like I am looking down on my life and I can't figure out how to move forward – actually, I do not know how to move in any direction. I just sit at home and cry a lot. I do not know how to push through this. I don't know if I want to push through this." (p.10)<br><br>"Let me tell you what losing my daughter has done to me – it has made my life pure hell. Every day I force myself to get out of bed and go through the motions of life, but I am really just in hell. There is no relief from hell. I live in hell, I eat in hell, I dream in hell, I work in hell. Everything I do is done in hell. My life is just hell, plain and simple. I don't see any way out of this hell." (p.11)	Black, V., & Heo, S. (2023). When a Child Dies by Suicide: An Interpretative Phenomenological Analysis Study. *Omega* , 302228231194213. Advance online publication. https://doi.org/10.1177/00302228231194213
Blaze & Roberts	"Almost all participants attended some form of bereavement support group after the loss of their sibling. The first bereavement services participants attended were general bereavement groups, attended with their families. Participant 4 said that "Mum had gotten in touch with the suicide bereavement support organisation, and they had a support group for people who have lost someone to suicide. It was more a parent's group." He added that the "sibling group that I go to, it's almost like, a little bit lighter, if that makes sense. Nobody really goes into those triggering points as much, which I find to be more helpful…I guess it's more a sense of belonging in that group than, than it is in the other one." (p.6)<br><br>"Participants also shared that this desire for understanding wasn't limited to support groups, but their friends and families, as well as professional mental health clinicians. Participant 10 shared frustrations when searching for a psychologist, mentioning that she would "ask the doctor, like, is there someone that specifically deals with suicide and siblings?… I don't want them to not understand." Participant 7 shared that "It took a long time though for me to get a psychologist that recognised that it wasn't just grief… it was deep trauma. […] She went on to note that professional services were useful" (p.7)<br><br>"Participants revealed that support and a space for their grief was facilitated well by their employers. […] Similar feelings were reported by other participants "My work was probably one of my biggest supports…Even when I returned to work, they took a lot off my plate.""(p.8)<br><br>"Participants also spoke of how they found professional services, such as psychologists, psychiatrists, and counsellors helpful in their bereavement, not just in terms of holding space for their grief, but also in providing education and insights into how their sibling might have felt, and how their own grief might affect them. " (p.9)<br><br>"Participants mentioned that already seeing a psychologist allowed them to access support for their sibling's death more easily "There's my psychologist. It was easy enough to contact him. It was via email, including setting up the session to see him." (Participant 4) and "I'd been seeing a therapist for years up to that point. So I was, you know, started seeing her more regularly after my brother died." (Participant 8)." (p.9)<br><br>"Conversely, participant 3 said that "We loved each other. Like it was like no other siblings. There was no bad blood. We never fought." She went on to mention that she had taken up his hobbies – "I learnt how to play piano… he gets me out of my comfort zone. He always has and continues to." (p.10)<br><br>"She also described her grief journey saying she was able to engage well with services, and was able to understand her grief as well "The counselling is very good. Through uni she was amazing" (p.11)<br><br>"Participants spoke about how their relationship with their parents changed as a result of the death of their sibling. Participant 3 spoke about how she became parentified, saying "I had to sit there and pick out my brother's casket, had to pick out what he was going to wear on the day, what flowers are going to be every single detail… I actually made the decision to shelter my parents because I'm just like, well, they're just useless at the moment." She was not alone in this change of relationship, participant 1 remarked that "I felt I had to kind of keep it together to help them get through. The funeral and all the administrative stuff that you just have to do…, I'll make the dinners and I'll walk the dog and I'll help do the funeral stuff." […] He went on to say that "I planned out his funeral because Mum wasn't doing well and he had to have a funeral. You know, it wouldn't be right not to give him one."" (p.11)	Blaze, P., & Roberts, R. M. (2023). Support After Suicide: A Thematic Analysis of Siblings' Experience. *Omega* , 302228231195922. Advance online publication. https://doi.org/10.1177/00302228231195922

Bottomley et al.	"Shannon (P8) explained that she practiced this sustained connection routinely in the wake of the loss of her father: "I go out to the cemetery once a week just to talk to him... whenever I'm out there and we're talking and a butterfly lands on my knee, it's like, there's my dad ... it's comforting—knowing he's there and now I'm here. We're together." (p. 96 - Results)<br><br>Linda (P7) said, "I thought about going back [to school] and getting my social work master's degree and specializing in suicide grief, and doing nothing but support people who are dealing with suicide grief." She (and others) further explained that the decision to participate in an intensive interview study of suicide loss was informed by a desire to better the lives of others who will be subjected to the pain of this particular form of loss: "I want to do this [interview] because it's very important to me. It's very important to me that people understand better, that the shock and grief and trauma of suicide is totally different than the normal grief " (Linda). (p. 97 - Results)<br><br>"Other participants described that directly helping others, such as establishing and engaging in peer support organizations and websites, has aided in their adaptation to loss and expressed gratitude for the opportunity to positively impact other survivors. Katherine (P6), who facilitates a peer support website and is surviving the loss of her daughter stated, "I feel very fortunate. I feel very humbled a lot by the contacts I make, and when I see the difference I've made to people, I feel so good that I'm able to do it, but I don't feel like it's because of me particularly, but because I happen to be the lucky one who has the opportunity to help." (p. 97 - Results)<br><br>"Micah (P3) stated, "After I went to the support group, I found out I wasn't the only person in the world that this kind of thing happens to, and that I wasn't crazy." Monica (P1), who lost her sister to suicide nearly 2years prior to the interview, commented on her recent experience with being in the midst of other survivors: "I'm wanting to connect more with people who are survivors of suicide because unless you've gone through it there's a certain camaraderie with anyone who's dealt [with] suicide, that I don't know that people that haven't could quite grasp unless you've lived it yourself."" (p. 97 - Results)	Bottomley, J. S., Smigelsky, M. A., Bellet, B. W., Flynn, L., Price, J., & Neimeyer, R. A. (2019). Distinguishing the meaning making processes of survivors of suicide loss: An expansion of the meaning of loss codebook. *Death Studies, 43* (2), 92–102. https://doi.org/10.1080/07481187.2018.1456011
Causer et al.	"we all got together and just sat and laughed and cried and laughed and cried. And, you know it was nice to be together, support one another. (Nurse) Many participants described being together with colleagues, sharing space, and talking as vital." (p.5)<br><br>"The strategies that participants engaged in response to these gaps included sharing grief and remembrance with peers; self-supporting, change-making, organizing, and rationalizing; wrangling processes, such as meaning-making; going into professional mode; not speaking and self-protection; and doing things differently." (p.6)<br><br>"The loss of a colleague is a shared experience; having the time to talk about that loss collectively appeared crucial for some participants through the earliest days following the death. The importance of talking, remembering, and crying together came through in several accounts, but more important was being physically present with each other. Participants felt that sharing helped them to process, release difficult feelings, and reconcile with what had happened. Those who were able to be together found this helpful. "And so people just shared stories, expressed their sadness, um, might have been a little bit of ventilation, it helped" " (p.6)<br><br>"There was a felt need by participants to remember their colleague. If facilitation of remembrance was absent, participants created ways to do so informally, like a chat in the workplace, formally, through activities such as visiting their colleague's grave, or through actions like creating a space in honor of their colleague. 'there was a tree right in the corner of the car park, and we, we made it [our colleague's] tree, and we went and planted things and put flowers in the tree. You know, just made it really beautiful.' (Therapist)" (p.6)<br><br>"Staff employed a range of proactive behaviors to support themselves and their colleagues. They made requests for counseling, accessed their GPs, found information online, drew on personal support networks, and found support via other organizations. [...] For instance, one nurse recalled arranging a get-together with colleagues." (p.6)<br><br>"By employing this range of proactive behaviors, participants created the space and time they needed to attend to tasks such as checking in on each other, organizing memorials, and working through their ideas and understanding about the death. 'We helped each other. We'd meet, we'd meet for a coffee somewhere and chat and say how we was feeling. I think we got more comfort from that.'" (p.6)<br><br>"I just completely burnt out and had to have some time off, yeah, everything just kind of piled up. Erm, and yeah, I definitely became depressed and I just, well my tank was empty, that's how it felt [...] Like I'd be sat at my laptop but physically couldn't do anything. So yeah I had to take [some time] off. (Assistant therapist)" (p.7)<br><br>"Participants described career impact, with some leaving their jobs: 'we're working with high levels of risk on a daily basis [...] I've found it really difficult, I'm, I'm actually, I'm leaving the team in the near future. So I'm I'm, moving out of mental health services.' (Allied health practitioner)" (p.7)<br><br>"There were more positive changes for other participants: 'I think what [their] death did, did do, as [my colleague] said is that it changed our personal culture of you know how we are with each, how we look after each other on the unit. How we, we check in with each other a lot more.' (Doctor)" (p.8)	Causer, H., Spiers, J., Chew-Graham, C. A., Efstathiou, N., Gopfert, A., Grayling, K., Maben, J., van Hove, M., Riley, R. (2024). Filling in the gaps: A grounded theory of the experiences and needs of healthcare staff following a colleague death by suicide in the UK. *Death Studies* , 1–12. https://doi.org/10.1080/07481187.2024.2337202
Čepulienė et al.	"Participants revealed that experiencing compassion and responsibility for their children or other people was a way to find the strength to live on: It was really difficult at first, but it's like I said two little kids and it's not okay. You have someone to take them, you can't just put them aside, you must stand up, stand tall and move forward. So in this case my focus is on the first days. (Elena, 34)" (p. 8 - Results)<br>"The wish to help others who suffered after similar losses helped regain some of the control over one participant's life: The journey continues even now slowly, like the muscles, the spiritual ones, they are stronger now, the first step shows this, that I want to do that group (self-help group) so I believe that this is a sign that if I can help others, that it's a sort of healing process. If that energy, when I start to move and I'm ready to share with others, the balance is being restored. (Liepa, 49)<br>"The wish to participate in the current study was also perceived to help others who suffer: Right after the loss, I received one questionnaire, then another, and my mom said stop, let it go, why do you need this, to reopen the wound, she said, don't you understand, I rethink the event over and over and over again, and a thousand times more, and I said it's not getting any better, and . . . if I'm doing a good job by sharing my thoughts, if they are worthy or not, but I say, maybe it'll help someone, maybe someone will see that moment, when it happens. (Dalia, 62)" (p. 8 - Results)<br>"Five participants spoke of the need and active ways by which to connect to spirituality during grief. For example, the ways to connect with spirituality during grief involved active praying: I never knew how to pray, because it always seemed, that there are some kind of rules, how you should address . . . If it's God, or the earth, or what you believe and somehow I thought I'm not doing it right and then I asked my grandmother, so how should I pray now, in your own words, how you want to, that's how you should pray, you'll be heard anyway, or so I would say a worldly prayer, the one for the dead of course, because I thought, that it is necessary. And then in my own words I'd just pray, I actually would just ask, not pray, that I wouldn't go crazy, that I'd be given strength, to be here, but I prayed to him. Yeah. For an easy path, I prayed for his forgiveness, somehow my prayer was related to his easy departure. (Laima, 28)<br>As well as spending time in a sacral place: And that is why, why I say that it would happened in church. I would want to kneel, to lean on those wooden things, to lay my head and cry. And somehow that atmosphere would provoke that reaction, those organs, when they play. And the priests' sermons, it always seemed that it was talking about me, or about him, or about our family. Somehow everything seems to be happening at the right time and place. (Rasa, 41)<br>Or listening to music, which started to have spiritual meaning after the loss: It has a special effect on me even now, mass and the sacral music, singing, it's unbelievable, maybe it's even painful, the music. I always, it tears me up. (Laima, 28)" (p. 8-9 - Results)<br>"Participants spoke of the many different forms personal rituals can take such as travelling and being in nature: So those journeys and world knowledge and new knowledge of nature, that you climb the mountain, it means those special paths right, and you get to the top and there is this huge wind, and you can't think of anything else and it's a new place for you and a place you can't find in Lithuania, you start to think about the world differently, new colors appear. You understand that . . . there is more of everything and it's different from what you're used to. Or when you stand up, on salt, the lake of salt right where it is white and there is no horizon, and you are in the middle of nowhere. You just hang in the middle and you understand that there is something . . . Something magical. (Nida, 35); creating something which could be related to emotional expressions: My nature is creative, and I am creative, so I searched through drawing and my hobby allowed me to find that, what I did in my childhood, I drew a lot, was picky, sewed and still I came back to that after the funeral, I found wool, I found wool and looked at the process in a more creative way. (Liepa, 49)<br>meditating and doing yoga: In that hour and a half when you do yoga, you just, translate stress, anxiety, tension, thoughts through the body, because when you do it, you don't think about other things, you just think how to do yoga. How to bend your legs, how to bend your body and other stuff. And in the end, there is shavasana, where you just lay ten minutes, it's called the dead pose. You must disconnect and not think of anything. So, this is the best thing, when you lay down, open your body, and not see or hear anything anymore. That is equal to meditation actually. And yeah. This thing was taught by the teacher and told us that when you come to yoga it's like a new page, you forget everything that is around you, and start from scratch. Actually, that helps a lot. (Jurga, 49)" (p. 10 - Results)<br>"Lastly, religious rituals such as attending mass every week for several participants were found to be helpful after the loss: This is about being religious, or how to say it, but I know now how much it snowed, how much it winded . . . but I walk there . . . and my knee joint is week, I have a hard time walking, fifty meters is a lot . . . I must go to church . . . so I had to walk a kilometer and three hundred meters. (Dalia, 62)" (p. 11 - Results)<br>"Other spirituality or religiosity related beliefs were also helpful, such as accepting the uncontrollability: As for spirituality, in the sense of religion, I discovered specific philosophical points in Buddhism. Through literature, I started to read more books that helped me . . . I found that the whole theory of mindfulness came from Buddhist philosophy . . . The simplicity of philosophy and the simplicity of being has helped me restore that spiritual balance . . . I am suffering because I wanted everything. It is that desire to have a person, the desire to be attached . . . the desire to control that situation . . . Well, it does not happen that way. This understanding of mind through mindfulness . . . and all these fundamental truths and points of reference in Buddhism may have helped restore that spiritual balance little by little . . . some kind of mantra, as some kind of reminder that no one has promised you anything. (Nida, 35)" (p. 11 - Results) "For some, the ritual of visiting the grave was a method to remind oneself of the fact of death: I need some kind of process, some kind of ritual, which would help me understand and accept. So, I drove to his grave. Traditionally, I lit a candle. (Egle, 31)" (p. 13 - Results)<br>"Our findings revealed and expanded the understanding of the role of spiritual activities, such as rituals, during suicide bereavement. Personally meaningful rituals were the most helpful for the participants to live day by day after the loss. This is a conscious choice to take a specific action [8] which has a personal meaning for the bereaved. The rituals vary from connecting to the deceased or relating to God, lighting candles to calming oneself down before sleep with gratitude, meditation, yoga, or an analysis of the day." (p. 20 - Discussion)	Čepulienė, A. A. & Skruibis, P. (2022). The Role of Spirituality during Suicide Bereavement: A Qualitative Study. *International Journal of Environmental Research and Public Health, 19* , 8740. https://doi.org/10.3390/ijerph19148740
Čepulienė et al.	"Dreams also helped the bereaved to find spiritual meanings during bereavement. [...] During the memorial service, I wore the same dress I had dreamt of. And then you think that there are Higher powers who maybe regulate everything" (p.1029)<br><br>"I dream of him, I dream that he is alive, that I communicate with him, I dream that I am still his wife, but I don't overestimate those dreams. After his death, I dreamt many nightmares that he is alive, that I come and tell him, you are not alive, I try to convince him that he is dead. < ... > I don't communicate with him anymore, but more because of a conscious decision not to have any relationship with the dead." (p.1030)	Čepulienė, A. A., & Skruibis, P. (2023). The functions of the dreams of the deceased: A qualitative study of women bereaved by partner's suicide. *Death Studies, 48* (10), 1025–1034. https://doi.org/10.1080/07481187.2023.2297063

Čepulienė et al.	"Several participants described spirituality through relationships with a higher power, others, and oneself. For example, Agnė, who became an active member of a charismatic Catholic movement after the loss, thought that holding onto a relationship with a higher power is an everyday task. During bereavement, it was Agnė's way to live day by day focusing on her relationship with God and not the one with grief" (p.6)<br><br>"Dalia, who leaned on Catholic traditions after the loss, but also searched for ways to regain trust in good humanity, thought that spirituality is for everybody because it is related to our nature" (p.7)<br><br>"“I always believed, but I did not pray every evening or participate in mass . . . However, I would go to the church and light a candle. For five minutes, I would have this silence during the day, be in peace . .. The relationship [with spirituality] changes . . . Now it is again something else. Now I pray every evening for my child” (p.8)<br><br>"Spirituality requires active involvement, such as reading, reflecting, praying, or attending mass. This involvement, according to Dalia, was rewarded by the relief about her husband's soul, provided by higher powers: “It was a real grief, every Sunday we went to church . . . we prayed, and she (the friend) helped me to remain, and the first year went by, and it was a relief . . . as if I helped him (the husband) to obtain the keys from St. Peter." (p.8)<br><br>"Spirituality by many participants was described as an action, including such spiritual practices as rituals, prayers, yoga, meditation, writing, and serving others. For example, Liepa practiced yoga and meditation to search for peacefulness during bereavement: “You can use Christianity. I practiced yoga, meditated . . . meditation is concentrating and getting peaceful with oneself, in here and now.” (p.8)<br><br>"“I need to be in the church alone . . . And these organs . . . these benches . . . I searched for therapy to release my pain. I went to my garden to take care of the flowers . . . The flowers tore the skin on my hands, and I felt that I want the flowers to hurt me, that I want to feel pain . . . It changes the focus from the pain in the heart . . . And in the church . . . I wanted to kneel . . . and cry . . . It would provoke me, the organs, when they start to play . . . The sermons of the priest seemed to be about him and me and our family.”" (p.9)	Čepulienė, A. A., & Skruibis, P. (2023). What is spirituality for women bereaved by a partner's suicide: A qualitative study. *Cogent Psychology, 10* (1). https://doi.org/10.1080/23311908.2023.2183676
Chan & Cheung	"Participant B: When [my wife] passed away, I always thought about the reasons that she killed herself. I kept thinking about it. When I started living by myself after she died, I had to deal with the day to day responsibilities. My wife did all the housework. But now, I need to have my routine. ... There are a lot of things that are out of my control .... I didn't know how much I loved her when she was still alive.... When she passed away, I realized I need her.<br>The men in this study had difficulties managing tasks that the decedent was previously responsible for doing, and did not accept help from other people." (p. 1847 - Results)<br><br>"Participant D: I'm afraid to talk to my friends because they will ask me about my children, so I avoid them. If we do see each other, I avoid the topic by changing the subject. I can't eat with them at the same table and don't know how to, and I don't want to tell anyone. Everyone is different. Some men just don't like to share with others. I don't like to share my feelings with others either. But in a group, the environment forces you to talk about it." (p. 1848 - Results)<br><br>"The participants who had lost a child reported a high degree of fatherly pride. The loss of their child was the same as losing a body part because the child was their flesh and blood. They attempted to reduce the pain of the loss by denying their grief. This denial was also related to their desire to improve family relationships. They redefined their responsibilities to protect the rest of the family and educate others about mental disorders. The participants who had lost their wife had to assume her responsibilities, including additional household chores such as running errands, buying groceries, and cleaning the home which they previously took for granted. In doing so, they became more attentive to their family. With the increase in responsibilities and labor burden, they felt the loss of and missed their wife even more." (p. 1850 - Discussion)	Chan, T. M. S. & Cheung, M. (2022) The “men in grief” phenomenon among suicide bereaved Chinese men in Hong Kong, *Death Studies, 46* (8), 1845-1852, DOI: 10.1080/07481187.2020.1855609
Chen M et al.	"I didn't want to work. An important people in my life had been lost due to suicide and I wanted to go with her.” (p.4426)<br><br>"“After my mother passed away, no one took care of my children. I can no longer go out to work (migrant temporary job in a city) and earn money. Life is very difficult.” " (p.4428)	Chen, M., Zhang, X., & McCormack, B. (2023). The lived experience of family members of older people who have died by suicide in rural China. *Nursing open, 10* (7), 4424–4431. https://doi.org/10.1002/nop2.1684
Chen Y & Laitila	"However, W's reconstruction mainly focused on external causal explanations for L's death and lacked self-reflection, thereby appearing incomprehensive. W's self-exploration started through reading books, pondering about his family and observing himself." (p.5)<br><br>"W relocated to live alone at some point between the third and fourth interview. He said that emotionally he could “come to terms with reality”, i.e., his wife's suicide. He had also taken action on his future. W summarized some of the things that had helped his recovery, including his hobbies, his zest for reading and thinking, his friends, the bereavement support group, etc. He remembered what L was like and thought about her personalities dialectically." (p.6)<br><br>"While Song was clear about the impact of the suicide on her and her family, she was “puzzled” about what areas she could work on and how to cope with her intense emotions in practice. Her current life arrangements and future plans had changed. She spent a lot of energy caring for her parents and had also had to postpone making decisions on her career development and romantic relationship due to her “too tired/exhausted” state." (p.8)	Chen, Y., & Laitila, A. (2024). Initial-Stage Suicide Bereavement Experiences: A Case Study. *OMEGA - Journal of Death and Dying, 89* (4), 1514-1534. https://doi.org/10.1177/00302228221095905
Chen Y & Laitila	"Life is like a game or a farce...... more often there are no rules......We consider ourselves the center of the universe, in fact we are just one......(rationalizing: 0.8) when I saw my wife's bone ashes (accidental death: 2.9), I thought about this, one day I will also be like this (choking with sobs)...... That's why I like reading some philosophical stuff......- psychology......Vipassana......philosophy......(rationalizing: 0.8) then you will find the human brain is a rational self. These kinds of things actually have rationality in the backstage" (p.12)<br><br>"I don't know the starting point of your question. It must be a massive blow to my private life, but it has no impact on my career, for my family and life, it is a...... The impact on the emotions is relatively large (uncontrollable emotions: 2.5)." (p.13)	Chen, Y., & Laitila, A. (2023). Longitudinal Changes in Suicide Bereavement Experiences: A Qualitative Study of Family Members over 18 Months after Loss. *International Journal of Environmental Research and Public Health, 20* (4), 3013. https://doi.org/10.3390/ijerph20043013
Contessa et al.	"Consequently, they describe all kinds of efforts to avoid those disturbing experiences, trying very hard not to think about the death and the deceased, as well as avoiding places and routines that are associated with the deceased." (p.735)<br><br>"I want to see if I can get a medical leave due to these persisting memories. It is not something fleeting for me, it is very much present in my day-to-day routine... It's like all the time, you know? It comes all the time, all the time, all the time, all the time. Every hour, every second, you know? When I go to the bathroom sometimes, I get scared to go to the bathroom alone, you know? Then my hands get all sweaty, my feet sweat ..." (p.735-736)<br><br>"I don't feel like getting out of bed anymore. I don't feel like working anymore. I come to work counting the hours for me to go home. I lost the joy in things. I don't celebrate anything else. I don't feel like celebrating birthdays, Christmas, new year. Nothing. Nothing else interests me, nothing more. I don't like to go out, I don't like to talk. People irritate me. It's like I hate life. I fret about everything. I get irritated by everything. Sometimes I don't even want to be talked to, I just want to be quiet. I just want to be in a corner alone. Isolated." (p.736)<br><br>"In the first days after the event, there is insomnia, hypertension, nausea, shortness of breath, and palpitations. Among the symptoms that persist, there are difficulties in sleep and weight control. “I gained 22 lb. I lost focus on things like that, mainly in relation to my weight and such.” - report of a sister . “I got really sick ...I got sick, I used to pass out, feel dizzy, couldn't eat, vomiting a lot... Early on, I couldn't eat and vomited a lot. But now I'm anxious so I eat a lot.” - report of a granddaughter. (p.736)	Contessa, J. C., Padoan, C. S., Silva, J. L. G. D., & Magalhães, P. V. S. (2023). A Qualitative Study on Traumatic Experiences of Suicide Survivors. *Omega, 87* (3), 730–744. https://doi.org/10.1177/00302228211024486
Creuzé et al.	"Parents or children who have been bereaved by suicide highlighted the difficulties in managing parental roles after the death (supporting children, providing affective reinsurance, managing day-to-day details), so that some children can assume responsibility as parents or adults and thus reverse parent–child interactions.<br>“I said to myself ‘I am no longer capable of educating my three other children,’ so it was even harder. What to do? What must we not do? What must we say? What must we not say? And so, we revisited everything that in fifteen years we should perhaps have done or not done. We no longer knew how to educate our children. Should we overprotect them? Should we, on the contrary, leave them completely free? So, all the educational benchmarks were shattered for quite a while; I think at the family level, we no longer knew what to do.” (P6)<br>“Your parents, they are not available for you because they think about the one who is no longer there (. . .) In addition to the one who is no longer there, they idealize him. So, how do you do exist? Who are you, what are you for? And it goes further, because in fact there is also the fact that maybe it would have been better if it were you who died.” (P4)<br>Parents who have lost a child by suicide notably doubt their ability to care for their remaining children. This doubt can also lead to a silencing of the grieving process in children who do not want to worry their parents. The “silent bereavement of siblings” has been reported by several participants who were bereaved by the suicide of a brother or sister.<br>“It's also good that you're going to take care of your parents because it's them who are suffering, it's not you, so in fact the relationship, well, the relationship is completely reversed (. . .) Your parents, they suffer so much that you shut up.” (P4)<br>“We often forget siblings in suicides; we often think of parents, and we often forget brothers and sisters.” (P7)" (p. 10 - Results)<br><br>"The participants reported the global improvement of family members over time, with posttraumatic growth occurring among members of their families. This growth led to the opportunity for families to restore balance to the family, including improved communication among family members or the restoration of parent-child interactions.<br>“I would say, overall, that it was an event in our lives that broke us, and like everything that doesn't kill us, it strengthened us and then made us go down other paths.” (P8)<br>“Today, we can have family meals, simple, peaceful family meetings; we can laugh, we can bicker, we can argue like a family, we can put things into words.” (P1)" (p. 11 - Results)	Creuzé, C., Lestienne, L., Vieux, M., Chalancon, B., Poulet, E. & Leaune, E. (2022). Lived Experiences of Suicide Bereavement within Families: A Qualitative Study. *International Journal of Environmental Research and Public Health, 19* , 13070. https://doi.org/10.3390/ijerph 192013070
Cutrer-Párraga et al.	"While trying to make sense of her father's death, Denise simultaneously witnessed her mother's emotional reaction to the suicide. “I just remember, afterwards, um, just watching the sadness of my mom and watching her cry and wanting to comfort her.” Following the suicide, as time passed, this pattern of Denise wanting to reach out, support, and comfort her mother continued. It is interesting that Denise's memories included supporting her mother and her mother supporting her. A lot of times I would sleep in her bed if I was scared or if I thought she was lonely. If she was lonely, I'd go and sit by her, and ask her if she needed anything and stuff. If she was sad and crying, I would just sit there and give her a hug." (p. 1848 - Results)<br><br>"Denise also felt supported by classmates, teachers, neighbors, and people in her school community. She explained, Once they heard what I had been through, I remember fellow students giving me gifts and writing me notes. I remember teachers putting their arms around me, you know telling me that they loved me [crying]. It helped to go back to school and know that everyone wasn't just talking about me. They were on my side." (p. 1850 - Results)<br><br>"Although Justin did not talk extensively about school or community support, he suggested that returning to school quickly helped to normalize his life after the suicide. He shared the following memories. “I remember going back to school. Going back to school must've helped to just normalize things because I felt pretty normal then.”" (p. 1850 - Results)	Cutrer-Párraga, E. A., Cotton, C., Heath, M. A., Miller, E. E., Young, T. A., & Wilson, S. N. (2022). Three Sibling Survivors' Perspectives of their Father's Suicide: Implications for Postvention Support. *Journal of child and family studies, 31* (7), 1838–1858. https://doi.org/10.1007/s10826-022-02308-y

Doehring et al.	"I experienced the spiritual practice of listening to sacred choral music as my central resource in grieving my son's death. I know that many other aspects of my life sustained me in this traumatic loss as well as past losses: a stable and loving marriage and family, meaningful work, a supportive church family and academic community, financial stability, and intercultural spiritual care and behavioral healthcare. Without all of these resources, my spiritual practice would not been as effective." (p. 246 - Unspecified)<br><br>"Listening to sacred choral music helped me experience self-compassion amidst grief. After Alex's death, I immersed myself in listening to sacred choral works on death as soon as I awoke each morning to the raw anguish of grief. Choral music has been a spiritual practice for me since singing in a choir in third grade, when I experienced through music a transfiguring sense of beauty that held me." (p. 247 - Unspecified)<br><br>"In recent years, I have realized that listening to choral music is a mind-altering spiritual practice that shifts me out of a habitual anxious orientation to stress and life in general into an orientation to self and world energized by love. Accessing compassion amidst grief helps me be compassionate toward myself and Alex and helps me receive the love and support that has poured in around me. Listening to choral music about death after Alex died helped me search for meanings in integrated ways that connected my physical and emotional grief with the meanings grief had for me. This integrative process of connecting body to heart to head helped me identify my religious struggles as I began to envision a memorial service for Alex. For example, I was immediately drawn to a choral work sung by the choir Seraphic Fire that I happened to be listening to at the time of Alex's death: "Good Night, Dear Heart" by Dan Forrest. The piece incorporates words from a poem by Robert Richardson that were chosen by<br>Mark Twain for the tombstone for his daughter Sarah, who died at age 20:<br>Warm summer sun, shine kindly here,<br>Warm summer wind, blow softly here,<br>Green sod above, lie light, lie light;<br>Good night, dear heart, good night, good night.<br>Choosing to have this choral work sung at Alex's memorial service was the first step in beginning to plan his service. I knew this music would provide an experience of beauty that connected heart to head. Addressing aspects of nature was a way to acknowledge how, especially in his last months, Alex often wrote haiku poems that began with an image from nature. The farewell words "Good night, dear heart" expressed my love and my wish that his death was, indeed, a good night of release from struggles. I hoped that the beauty of such music would generate for the assembled congregation at Alex's memorial service a sense of home that transcends death. As pastoral theologian James Nelson (2004), writing about his struggles with alcohol addiction, said so simply, "Beauty can draw us home" (p. 168)." (247 - Unspecified)<br><br>"Beginning each day by listening to sacred choral music helped me pay compassionate attention to the struggles I experienced with beliefs about Alex's suffering and death, in particular with many traditional Christian beliefs about death used in funeral liturgies" (248 - Unspecified)<br><br>"In retrospect, I can see how my daily spiritual practice of listening to sacred choral music helped me become more compassionate toward myself. Music helped me trust the ways I reacted to beliefs that were not meaningful at this time. Music helped me trust that others would be compassionate toward my struggles. The self-transcendent experiences fostered by music helped me become flexible in my search for meanings that were complex and integrated around my core spiritual experiences of compassion, love, and benevolence." (p. 248 - Unspecified)	Doehring, C. (2019). Searching for wholeness amidst traumatic grief: The role of spiritual practices that reveal compassion in embodied, relational, and transcendent ways. *Pastoral Psychology, 68* (3), 241–259. https://doi.org/10.1007/s11089-018-0858-5
Dransart et al.	"Dealing with the impact of suicide revolved around reconstructing a routine from which the deceased was absent and reshaping relationships within the family. The absence of the deceased person had to be integrated into both daily activities and family life and history. This was gradually achieved by modulating physical and mental energy and reconfiguring roles in the family" (p. 997 - Results)<br><br>"The suicide as a destructive and central event marked an inner shut down of the survivors' whole being. Survivors reported giving up or drastically reducing most activities. For a year, a mother might neglect housework and only go shopping once a week, 10 minutes before closing time to minimize her contact with the outside world." (p. 999 - Results)<br><br>"While making necessary adjustments, survivors felt the need to carry on with their life as much as possible. Activities continued to take place, yet for a time, their frequency and intensity were modulated. For example, a father continued to participate in the church choir's weekly rehearsals to enjoy friendly interactions and support from his colleagues but refrained from performing at concerts for the first few months following the suicide. Survivors drew on the relative stability of their outer life to find the necessary strength and resources to journey on an inner path of grief and growth." (p. 1000 - Results)<br><br>"These survivors also sought more informal help from support groups, where they found comfort and guidance. After a certain amount of time, they often started leading such groups." (1000 - Results)<br><br>"The suicide of their loved one caused survivors to deeply question their priorities in life, giving up or only partially keeping up some of their usual activities… We lost some friendships, even longstanding ones, of course we had changed, we were not the same persons as before, we had a very heavy heart, people could not talk to us like before anymore, visit us for a cup of tea, I wasn't baking anymore, nobody was there when we needed it. (mother)" (1001 - Results)	Dransart D. A. C. (2017). Reclaiming and Reshaping Life: Patterns of Reconstruction After the Suicide of a Loved One. *Qualitative health research, 27*(7), 994–1005. https://doi.org/10.1177/1049732316637590
Dransart et al.	"For some survivors, suicide became a driving force (commitment type). Honouring the memory of the loved one meant living one's own life to the full and dedicating it to the service of others, such as by helping the cause of suicide prevention or persons who are in a similar situation. The memory of the loved one became a motive for a commitment to civil society in memory of him/her. This gave meaning to their life and their future: This is why we are active in this group [of survivors] to raise awareness and support survivors ... , so that his death is not in vain (YAE, father)." (329 - Results)]	Dransart, D. A. C. (2013). From sense-making to meaning-making: Understanding and supporting survivors of suicide. *British Journal of Social Work, 43* (2), 317–335. https://doi.org/10.1093/bjsw/bct026
Eng et al.	"Many participants described how they felt they had lost control over drug or alcohol use after the suicide, or that they had had to make deliberate efforts to control their use. For those who felt they were using drugs or alcohol above recommended limits it was clear that they saw this as a problem, with potentially harmful effects. For those who limited their use, this was out of a fear of the effects of drugs or alcohol on their emotions or their health, and in some cases a fear of this leading to their own suicide.<br>(1) Loss of control over drug or alcohol use. Many participants were aware of having lost control of their substance use since the suicide, for varying periods of time. The terms they used ("stupid amounts"; "binge drinker"; "out of hand"; "I had a problem") indicated an awareness of the harmful effects of the behaviour.<br>"I cannot go out anymore for a couple of drinks instead I drink stupid amounts" (30 years old female, 2 years since close friend's suicide)<br>"Before the death I would hardly ever drink. In the 2 months after the death I drank every day and began smoking-again something I never did and hated. I have since tried drugs and was entirely against the use of them before the bereavement." (20 years old female, 5 months since close friend's suicide)<br>A number of participants looked back on this as a distinct period after the loss, following which they had either regained control or no longer needed this intoxication. The implication was that daily or binge drinking or drug misuse had served a purpose over that period, but then the benefits had ceased to outweigh perceived harms.<br>"I was pretty much drunk for 3 or 4 months last year, wound up in AA. Since then though I found my mood switched again and I've barely drunk alone or in large amounts since." (20 years old female, 6 years since father's suicide)<br>"I went through a stage of drinking almost every evening. I have since dealt with this but the worst thing about it was I would drink at home as opposed to going out with friends and having 'a good time'" (23 years old female, 12 years since mother's suicide)"<br><br>(2) Restraint over use of drugs or alcohol. In contrast to those who perceived a loss of control, a separate group of participants described how they controlled their use of substances by consciously limiting it. Many reported that the bereavement had changed their attitudes towards drugs and alcohol. They were either more conscious of not allowing themselves to become intoxicated or had set specific restrictions on their substance use. Such restraint was often described as having arisen from an awareness of having lost control over drug or alcohol use in the immediate aftermath of the death. This sub-theme therefore overlapped with the previous sub-theme describing transient excessive use.<br>"For a year after I didn't drink because I didn't want to use alcohol to solve a problem since I knew this would only make it worse. My brother who was 16 then, however, did increase his drinking." (21 years old female, 2 years since father's suicide)<br>"stopped taking drugs altogether. Do not drink as heavily as feel too overwhelmed now when I do, (I) get anxiety and an urge to want to keep drinking so I now avoid drinking heavily as much possible. (30 years old female, 1 year since close friend's suicide) Sometimes the reported motivation for reining in substance use was a fear of losing control over emotions or over continued use. Some described an awareness of being able to feel their emotions more when they were drunk or high, but finding this overwhelming. The fear of becoming engulfed by emotions, or of disclosing hidden feelings to peers, lay behind efforts to restrict use. This lay in contrast to theme 2 below, in which other participants used drugs or alcohol to numb their emotions or escape from them.<br>"For 2 years after the suicide I stopped drinking alcohol as it made it harder for me to control my emotions." (27 years old female, 3 years since ex-partner's suicide)<br>"I have got extremely drunk accidently since the death and ended up crying and running away from people I am with to be on my own, although I only vaguely remember these nights. I sometimes feel like drinking more than normal, but sometimes avoid it for fear that hidden feelings may come out while drunk." (21 years old female, 6 months since grandfather's suicide)<br>For some participants their restraint over use of alcohol or drugs related to a strong aversion to its use, seeing this as associated with the suicide. In some cases this involved avoidance of the substance used in overdose. For others, notably those bereaved due to parental suicide, it was more indirect and related to their experiences of growing up with an alcoholic parent. Although it was not always clear whether alcohol was implicated in their parent's suicides, the avoidance of alcohol seemed to relate to an aspiration that their own life would not "turn out like that".<br>"My cousin was found with weed in his system, so actually I think I have developed a fear of drugs and avoid them more than I perhaps would have." (22 years old female, 10 years since cousin's suicide) Active avoidance was not true for all respondents for whom alcohol misuse had particular resonance. One 22 years old woman noted that her alcohol intake was unchanged since her father's suicide four years previously, and that this was "quite ironic considering my father died from alcohol poisoning."" (p. 5-6 - Results)	Eng, J., Drabwell, L., Stevenson, F., King, M., Osborn, D., & Pitman, A. (2019). Use of Alcohol and Unprescribed Drugs after Suicide Bereavement: Qualitative Study. *International journal of environmental research and public health, 16* (21), 4093. https://doi.org/10.3390/ijerph16214093

Eng et al. Continued	"Many participants were clear that they used drugs or alcohol for a specific purpose, and therefore as a coping mechanism. This theme was broadly divided into those who had increased their use of drugs or alcohol to block out emotions, to relax or to sleep, and those whose intake had not changed but for whom drinking took on a special significance in relation to the deceased.<br>(1) Coping with overwhelming thoughts and emotions. Many participants described using alcohol or drugs to cope with the emotions they were experiencing, either to reduce their impact by blocking them out, or to escape from them. This contrasted with those who used drugs or alcohol to slow thoughts down to a manageable pace, as described below. Participants who did not want to feel anything after the bereavement used substances as a conscious means of blocking emotions, to avoid feeling overwhelmed." (p. 7 - Results)<br><br>"Whilst many reported using alcohol and drugs to cope with their emotions, a small minority of participants described using substances to relax and specifically to help them sleep. This was more apparent in relation to drug use. Achieving relaxation either meant feeling a release or slowing down one's thoughts. Those who suffered from insomnia used drugs to slow down their thoughts and relax in order to help them sleep. The accounts of some suggested that in the longer-term their drug use had led to apparent dependence." (p. 7 - Results)<br><br>"(2) Honouring the memory of the deceased. A few participants reported no change in their use of alcohol and drugs after the bereavement but described how they regarded alcohol or drug use as a way to emulate or honour the deceased. This was more apparent in relation to alcohol use. By engaging in behaviour characteristic of the deceased, such as drinking their favourite drink or going out to party, they felt they were able to respect their memory." (p. 7 - Results)<br><br>Theme 3: Attribution of Drug or Alcohol Misuse to External Factors. Although most people acknowledged some agency over their use of drugs or alcohol, a minority of participants attributed their increased use to external influences, particularly the prevailing peer culture. The social acceptability of heavy drug or alcohol use in local peer groups, particularly when transitioning to new social groups at university or at certain times of the year (Christmas, exams), was viewed as highly influential on substance use. (p. 7 - Results)<br><br>"It seemed it was hard for respondents to judge the extent to which the bereavement had affected their drug or alcohol use in the context of these social influences and life transitions. It was also hard to interpret the extent to which respondents were in denial about their own agency over substance use, suggesting a tendency to an external locus of control." (p. 8 - Results)<br><br>"Our analysis of qualitative data describing the impact of suicide bereavement on use of drugs and alcohol identified a variety of patterns in substance use following the loss. The majority of participants described a change in their use; whether increased or decreased, transient or long-standing. We also identified a broad range of rationales for such changes in use, as captured in our three main themes. Overlying these themes was a tension between two dimensions: degree of control over use, and awareness of the potential for harm. The latter was apparent in participants expressing concerns about the degree to which they were relying on substances, an awareness of the health consequences, and of the effects on their emotions" (p. 8 - Discussion)	Eng, J., Drabwell, L., Stevenson, F., King, M., Osborn, D., & Pitman, A. (2019). Use of Alcohol and Unprescribed Drugs after Suicide Bereavement: Qualitative Study. *International journal of environmental research and public health, 16* (21), 4093. https://doi.org/10.3390/ijerph16214093
Entilli et al.	"Many participants who looked for support (43.9%) stated they did not find obstacles in reaching for help. Lastly, only 20 participants out of 123 (15.2%) received a direct outreach from mental health services." (p.6)<br><br>"The most used support service was a psychologist or psychotherapist (72.3%) and a General Practitioner (59.4%). Psychiatrists were indicated in 42.6% of cases, and there was no significant difference between the use of formal support groups (37.6%) or mutual aid groups (39.6%)." (p.6)<br><br>"Only 18.1% of participants had used online services, and the most employed online tool were online forum groups, both administered by a professional (10.6%) and by other survivors (10.6%); telephone services and live-chat services were used respectively by 8.7% and 7.7% of the respondents, while the least used was a support via email (96.2% declared they have not used it)" (p.6)	Entilli, L., Ross, V., De Leo, D., Cipolletta, S. & Kõlves, K. (2021). Experiences of Parental Suicide-Bereavement: A Longitudinal Qualitative Analysis Over Two Years. *International Journal of Environmental Research and Public Health . 18* , 564. https://doi.org/10.3390/ ijerph18020564
Entilli et al.	"Excessive working: It's not paid work but I'm always busy doing something and that's the way I operate. (P7, Father)" (p. 4 - Table 1 under codes observed at 6, 12, and 24 months)<br><br>"Drinking excessively: I can come home, and I get very, very depressed, very much like my son. If I come home and I have two drinks, and I feel—I love living, I love life, so happy. But then I just, that experience, I just like having a few more and a few more and a few more. I just want people to hurry up and get home so we can eat so I can stop drinking basically, that's really what I do . . . I don't really understand that, but it makes me feel very, very happy, I'm telling you. The next day, yes . . . I have been a bit depressed in the mornings, especially with the work I've got and whatever. (P8, Father)" (p. 5 - Table 1 under codes observed at 6, 12, and 24 months)<br><br>"Difficulties in sleeping: Dissatisfied [with the quality of sleep], I wouldn't go to very yet because I'm not cranky about it but it's becoming a bit of a worry. (P6, Mother)" " (p. 5 - Table 1 under codes observed at 6, 12, and 24 months)<br><br>"Self-care: There's kind of two dates that stick out in my calendar, one is the day he died and the other is his birthday. Both occasions of the two years, I've taken a day off work if I've been at work, if it's not a weekend, and I've taken a drive off to the beach or up to the hills, and I suppose wanted to not do the normal stuff. So, I wanted to mark the occasion by breaking out of the ordinary routine, and I wanted to take time to reflect on where things have got to. That's been a really positive thing. (P9, Father)" (p. 5 - Table 1 under codes observed at 6, 12, and 24 months)<br><br>"Memorialization: I talk to him, like I'm writing. I don't know what you call it, but I've got a book and I write, and I talk to him quite often in the book. I've said all that to him, but it just seems like you're really going now, no one is sort of, not cares about you, but you've sort of disappeared now, yes, you're off on your own little journey type thing . . . that is the best thing. The two great things that I've felt I've done, one is that, and the other one is going to church and reading the bible. (P9, Father)" (p. 5 - Table 1 under codes observed at 6, 12, and 24 months)<br><br>"Faith: I've been a fence sitter; I've always been a fence sitter. I used to take the family to church when they were young and they enjoyed that, but I've always been a fence sitter. But since he went I thought, hang on a minute, even if it's not true, I want to believe in it. I've fallen off the fence to the god side. I'm going to church once a week, they've actually got me stuck into moving the chairs for them twice a week, because you know how the church like get people to help them and all that sort of stuff. But now I'm the chairman of church, because I move the chairs twice a week. (P8, Father)" (p. 5 - Table 1 under codes observed at 6, 12, and 24 months)<br><br>"Practical support, Withdrawal" (p. 5 - Table 1 under codes not observed at 24 months)	Entilli, L., Leo, D. D., Aiolli, F., Polato, M., Gaggi, O., & Cipolletta, S. (2023). Social Support and Help-Seeking Among Suicide Bereaved: A Study With Italian Survivors. *OMEGA - Journal of Death and Dying, 87* (2), 534-553. https://doi.org/10.1177/00302228211024112
Entilli et al. Continued	"Several adaptive and maladaptive coping strategies were observed: some similar to those encountered previously. Avoidance of discussing the death was still present (for females and males), although several parents described feeling able to disclose more about the death than previously. Parents reported being able to talk about the death with relatives and friends but had occasional difficulties with their partners and significant struggles with their children. Avoidance through excessive working was still observed in males. Another maladaptive coping strategy was described by one woman, who reported binge gaming as what she believed was her only available resource for socializing and keeping her days full. Drinking excessively was still a recurrent behavior in males; however, some reported they had significantly reduced their alcohol consumption in order to improve their health. Somatic dysregulations, such as difficulties in sleeping, were observed, but the subsequent use of drugs or alcohol to induce sleep (as previously described) were not reported. A number of health issues, related to the age of the participants, had appeared in the last year or seemed to have been aggravated. Parents who were dissatisfied with health professionals reported their own non-compliance with their practitioners' directions, in some cases being hostile towards the use of prescribed antidepressants, and reluctant to seek help from a mental health professional when feeling overwhelmed. Parents whose children had used antidepressants prior to their suicide said they wanted to avoid what they perceived as negative effects, or declared that they believed the pharmacologic treatment was ineffective because it had led to their child's death. Another maladaptive coping mechanism that emerged for the first time was the belief in paranormal events involving signs from the deceased child. One father talked of how he and his wife approached a medium, in an attempt to contact their child, and two mothers described events in which they believed they could hear the presence of their child or had received messages from them. Numerous examples of adaptive coping strategies and new positive behaviors were reported. The most frequently observed was self-care: parents reported starting to take better care of their physical health (exercising more and reducing alcohol consumption) and, in several cases, spoke about their new ability to make time and space for themselves, especially closer to the anniversary of their loss. Some parents described how being able to listen more to their own needs had a positive impact on different levels of their life; for example, several fathers decided to leave their job and find a less stressful occupation. Others reported taking up enjoyable hobbies that helped to distract them during the day, and several described a generally more positive attitude towards life's events. Parents described the benefits of their faith and participating in their church's social life. Some reported becoming closer to their church after having distanced themselves soon after the loss, and described how the group helped them to keep a routine and obtain informal support. Parents also reported new rituals of memorialization, carried out by their child's friends, for example, erecting a commemorative plaque and creating a video memorial. Continuing a personal relationship with their child through keeping a journal or writing letters was described by some fathers, who reported this allowed them to maintain a direct, intimate, and authentic relationship with the child, to whom they wrote using the same tone and attitude they used when they were alive. The journal was also considered a testimony to show to children and grandchildren. Some parents reported regretting some of the actions they adopted as coping strategies in the early months after their loss, such as cleaning the child's room and throwing away most of their belongings. In contrast, another parent reported the action of tidying the child's room and keeping only the most personal belongings as a step closer towards acceptance and a new connection with the surviving children, who had chosen some important personal items to keep, such as their deceased sibling's t-shirts. Within the family environment, some participants still report frequent conflicts with their partner; with some couples having divorced since their loss. Some parents spoke about their difficulty in reconnecting their relationships with their surviving children, who do not want to discuss the death of their sibling. In one case, a mother reported feeling her living child directly blamed her for the suicide of her brother. Overall, compared to the previous interviews, some parents believed that the loss has brought the family closer together, describing how siblings have strengthened their relationships and maintained more regular contact with their parent/s. Lastly, experiences with professional support through attending individual counseling and/or support groups were rarely reported. Some participants said they had ceased going to individual counseling or do not consider it useful. However, two mothers reported the benefits of support groups, where they described feeling understood and finding insights through their own personal posttraumatic growth." (p. 7-8 - Results)	Entilli, L., Leo, D. D., Aiolli, F., Polato, M., Gaggi, O., & Cipolletta, S. (2023). Social Support and Help-Seeking Among Suicide Bereaved: A Study With Italian Survivors. *OMEGA - Journal of Death and Dying, 87* (2), 534-553. https://doi.org/10.1177/00302228211024112

Eskin et al.	"Several individuals mentioned that they encountered changes in their social interactions after a suicide loss. For some, it entailed navigating shifts in roles and taking on extra responsibilities. For others, it meant they had to focus on helping others who were also bereaving instead of attending to their own needs. One participant elaborated on that: My mother's and father's physical problems have increased a lot.... I am the only person in the house to take care of them." (p.4)<br><br>"Another participant, whose husband died by suicide, shared how her family chose to stand by her, leaving everything behind to move in with her and her son. This significant decision led to role changes within the family, as her father assumed a paternal role within the household to provide support and care in the absence of her husband" (p.4)<br><br>"One participant even opted to extend their forgiveness further, which led to reconciliations: "When I faced the reality of death, I established closer relationships with my other relatives and started to ignore minor problems or issues"" (p.4)<br><br>"Some individuals who experienced suicide loss became more selective in their social circles and chose to distance themselves from certain individuals. They stopped seeing other people for various reasons, such as being reminders of the deceased, not providing support during their grief, and becoming a burden on the bereaved individuals" (p.4)<br><br>"Since I lost my uncle one year after my father's death, my life returned to the sadness and depressive state of that time, and it became challenging to recover. As I tried to cope with my previous sadness and the new one simultaneously, my daily duties were utterly disrupted, like school exams. Even basic household chores, like cooking, became challenging to manage." (p.6)<br><br>"It was suffocating. I knew they had good intentions, but I didn't particularly appreciate being pressured constantly. I socialized with others to avoid coming out as impolite when I wanted to be alone myself. The fact that they were more interested than usual created a sense of emergency. In fact, I wanted to return to the flow of everyday life as soon as possible." (p.7)<br><br>"Due to the lack of support groups in Türkiye, one participant had to join a support group in another country as this support was unavailable in Türkiye: "Definitely, the support is not sufficient. I remember signing up for a suicide support group from England" (p.7)	Eskin, M., Karkin, A. N., Eyisoylu, E., Seker, E., Yilmaz, E., Sevin, G., ... Ranjbar, H. A. (2024). The experiences and support needs of Turkish individuals bereaved by suicide: An online qualitative investigation. *Death Studies* , 1–13. https://doi.org/10.1080/07481187.2024.2386062
Feigelman et al.	"Our respondents showed somewhat higher propensities to smoke cigarettes (21% as compared to 17% in the NSDUH survey) and to consume marijuana (27% as compared to 14%) and to drink alcohol socially (76% as compared with 53%)." (p.8)<br><br>"Table 2 suggests more of our bereaved respondents, smoked cigarettes, at a somewhat higher rate of four percent higher, more used marijuana, at a 13% higher rate, and more used any alcohol at a 23% higher rate." (p.9)<br><br>"Our findings show consistency with what has been found in prior studies of bereavement and substance use, namely higher rates of drug use and misuse for the bereaved, as compared to the non-bereaved (Andrade et al., 2023). This was noted in a 4 percent higher rate of cigarette smoking, a 13% higher rate of marijuana use and a 26% higher rate of social drinking of alcohol, comparing our respondents to nonbereaved adults in the 2023 National Survey of Drug Use and Health." (p.13)	Feigelman, W., Cerel, J., McIntosh, J. L., Brent, D., & Gutin, N. (2019). Suicide Bereavement and Differences in Religiosity. *Crisis, 40* (3), 176–185. https://doi.org/10.1027/0227-5910/a000546
Feigelman et al.	"Two associations were statistically significant: 82% of the bereaved reported praying at least weekly or more often compared with only 75% for the nonbereaved; and, also significant, 85% of the bereaved believed in an afterlife compared with only 78% of the nonbereaved." (p. 178 - Results)	Feigelman, W., Cerel, J., Gutin, N., McIntosh, J. L., Gorman, B. S., Bottomley, J. S., & Edwards, A. (2024). Examining the Associations Between Substance Misuse and Suicide Bereavement. *OMEGA - Journal of Death and Dying.* https://doi.org/10.1177/00302228241254133
Ferlatte et al.	"Flowing from these different forms of stigma, Nicholas and Sheldon were reticent to seek professional help to assist them with their grief in the first few months following the death of their partners. Instead they found alternative ways of coping with the emotional pain they experienced. [...] Immediately after their partners' deaths, both participants recalled how they engrossed themselves in their work as a distraction to avoid thinking about the tragic events that had just unfolded." (p. 1283 - Findings)<br><br>"I worked the next day, because I couldn't bear to be at home. I told my boss I'd be late, because of what happened and everything, but I kind of needed to be at work. For me, that helped me. I still thought about it, it wasn't like I was putting it out of my mind, but it helped me have some distractions." (p. 1284 - Findings)<br><br>"Additionally, Sheldon described that while he was unable to discuss his emotions with someone else, he did express himself by doing art: 'I bought a canvas, and I painted, started painting a mural about him being upset, and calling me, and that kind of helped'. He also described how he felt the need to dissever the bonds with Vincent, which he did by removing mementos from their relationship: 'One of my ways of dealing with it was I burned all the letters he send me, not out of anger, but just to try to get it out of my head kind of thing'." (p. 1284 - Findings)<br><br>"In contrast, Nicholas tried holding on to the memories of his deceased partnered by remaining in the apartment they had shared for several years. At the start, he saw some benefits in being alone in the space they had shared many beautiful moments together. Often, Nicholas would talk to Clayton. While this coping strategy may have been helpful to him in the first few weeks of his grief, he realised after a while that it had become unhealthy. He described how he was unable to recover from the loss of his partner and that he both needed professional help and a change of scenery to move on" (p. 1284 - Findings)	Ferlatte, O., Oliffe, J. L., Salway, T., & Knight, R. (2019). Stigma in the bereavement experiences of gay men who have lost a partner to suicide. *Culture, Health & Sexuality, 21* (11), 1273–1289. https://doi.org/10.1080/13691058.2018.1556344
Fraccaro & Tosini	"Similarly, another mother, who had lost her daughter to suicide, said: "In the meantime I go on the Internet to find other similar cases. It's difficult to talk to others. It's not the same as when you lose a child to disease." (p.8)<br><br>""The group has been the only place where I could talk about my brother freely and about my experience [...] This is where I identified with other people [...]. And I believe that the group is one of the most important aids for the people who have been through this, more than a psychotherapist, a psychologist, or a priest. It depends on the person, but for me, it was. [...] Before joining I had obsessive thoughts, I obsessed over this every day: over my brother's death, over what I could have or not have done, over what my family could have or not have done. And these thoughts sucked a lot of energy out of me, they didn't allow me to live peacefully. I needed a group to vent these thoughts."" (p.12)<br><br>"One thing that I find extraordinary is to have been able to engage in very strong relationships and bonds of affection with these people. So much so that even now that I don't attend the group's meetings anymore, because my path has somehow ended, I am still in contact with these people [...] I consider them friends. I believe that this has been the most positive thing, because the forging of significant bonds is a return to life" (p.17)	Fraccaro, D., & Tosini, D. (2023). The suicide support group as a signifying agent and emotion transformer: A contribution from a micro-sociological perspective. *Death Studies, 48* (6), 550–560. https://doi.org/10.1080/07481187.2023.2246037
Froese & McDermott	Nicole, alongside her husband and a young relative who had stepped in to help farm after her father's passing, dedicated herself to building a stone wall for her garden. The process offered a stillness within herself where she could relinquish her emotional turmoil into the garden. [T]hose two things come together for me: dad dying that spring and that stone wall, that beautiful stone garden wall happened the same year. And there were a lot of tears shed there, there was a lot of swearing, and there was a lot of laughter and hard work...[T]he garden has always been – I really think that's where I started saying that, that I solve all my problems and most of the world's. It doesn't matter if you whack away at that weed, and and, hoe it to death. Like, you can do that ... I was never somebody that was going to use a punching bag, but um, hoeing those weeds, um, that I can do. And I could get some of the aggression and the anger I felt out that way. By physically exerting herself, or as Nicole described it "just hard manual labor," through constructing her garden wall and gardening, she externalized her inner emotional pain. (p. 415 - findings and discussion)<br><br>Rebecca similarly experienced relief by worshiping "with all [her] heart," vocally projecting and letting go of some of her pain. "Singing his favourite hymns" nourished contemplative reminiscences and cultivated an affirmative spirit in the good- ness of reality she believed God provided. (p. 416 - findings and discussion)<br><br>Embodied, contemplative leisure activities such as gardening, sing- ing, and tattooing provided them ways to externalize their hardships and opportunities to connect with the ruinous aspects of their loss, and powerfully release them through "hoeing the weeds to death," "singing with all of [their] heart," or marking the body. (p. 416 - findings and discussion)<br><br>For Nicole and Rebecca, their faith played an integral role in navigating the emotional chaos from their father/brother's suicide. Through praying, reading the Bible, or having others verbally affirm them of God's love, they brought positive emotions and hope inwards to themselves, feeling comforted, strengthened, and freed from the guilt they felt for being unable to prevent his death. (p. 418 - findings and discussion)	Froese, J., & McDermott, L. (2024). "When All the Wheels Fall off": Leisure's Potential Role in Living with Suicide Loss. *Leisure Sciences, 46(* 4), 405–424. https://doi.org/10.1080/01490400.2021.1985663
Gallardo-Flores et al.	"It's all good. We call each other, we are in a WhatsApp group, all of us women, together, but we often call each other privately if we see that something is up, because we know each other so well that, from the way we write, we already know how other people are doing." (Results, pg 11)<br><br>"The mistake I'm making - I know it, I don't need to be told - is that I completely distance myself from everyone. I withdraw. I don't want to talk to friends, or to relatives. I don't want to talk to anyone, and now I'm very afraid of detachment." (Results, pg 13)<br><br>"I work with my own students. As I said, I'm a high school teacher and I started to work on grief with my students. When I told them what had happened to me, it connected with their pain as well. When I joined the class the children gave me so much life! My students gave me so much strength. I worked with them on the pain they had inside, and that helped me to create what I call the 'Scar Club' in my free time." (Results, pg 14)	Gallardo-Flores, A., Morán-Carrillo, J.-M., & García-Carmona, M. (2023). The Detection of Resilience in Families Grieving Over a Suicide. *Omega: Journal of Death and Dying* , 302228231219047–302228231219047. https://doi.org/10.1177/00302228231219047

Genest et al.	"What was important was how each individual family member managed after the death, but also that their reactions were respected. For example, in one family where the adolescent jumped in front of a subway train, the mother knew the station where the suicide had occurred, and she visited it each year on the anniversary of her child's death. On the contrary, the father did not want to know the station, nor did he wish to visit it on the anniversary. Nevertheless, he respected his wife's wishes and, in turn, she respected him for not wanting to know or visit. The support and mutual respect between family members allowed for the expression of emotions among them." (p. 299 - Results)<br><br>"Support availability refers to the informal and formal support that existed in the family environment after the suicide. For three families, they already had formal support from a professional prior to the suicide. For example, one father had been diagnosed with bipolar disorder and was being followed by a family doctor and psychiatrist before the suicide. He reported that the support that he received from these physicians had been beneficial for him and his family in the aftermath of the adolescent's suicide. He said, "If I hadn't had them [the physicians] . . . this kind of support helped me progressively to gain control over the situation."" (p. 299 - Results)<br><br>"For all families, it was important to remember the deceased. Rituals were helpful as they kept the memory alive, but also structured the grieving process. Cultural rituals also structured the grieving process. Structured moments such as funerals and anniversaries (e.g., 100 days, 1 year) were important for the family members, as they felt supported during each step and could remember the adolescent." (p. 299 - Results)<br><br>"Creating a special place, such as a box with important mementos, was another way that families kept the memory of their family member alive. They said that this box would stay with them forever and, although they did not need to look at it every day, it was important for them to know it existed. As one mother explained, "I've asked everybody for pictures of her, I've annotated them, those albums are still in her room, on her desk, two beautiful albums and sometimes I look at them and it comforts me." This box usually contained positive memories associated with the life of the adolescent." (p. 299 - Results)<br><br>"If verbal communication was difficult, families had to discover new ways to communicate. For example, one sister said that when she wanted to talk to her mother about her feelings or about the suicide, she preferred to write a letter. She said, "When I wanted to talk to my mother, I wrote a letter that I left in her room and the last sentence was always "don't come talk to me after." We were only talking about it sometimes after like two or three weeks"" (p. 299-300 - Results)<br><br>"Another experience that took place within families was the regaining of homeostasis. When the adolescent's suicide happened, it usually disrupted the equilibrium of the family system. Therefore, for transformation to occur, families first needed to regain homeostasis or achieve a new form of homeostasis. [...] Having a shared goal or a shared project was another way for the family to regain homeostasis. The shared project could be a family trip, whereas for others it was a new house." (p. 299-300 - Results)<br><br>"Making sense of the suicide usually took place within the family, but also happened outside the family. Some individuals looked for answers in books. As one mother said, "I seek information, I read a lot, a lot of books on bereavement, it helps me understand." Others searched for answers by talking to the adolescent's friends, to professionals, or to other suicide survivors. But in the end, as one mother offered, "we have to stop asking questions and looking for answers. When you are able to do so, you are able to move forward." (p. 300 - Results)<br><br>"Some participants stated that they occasionally needed to force themselves to take part in activities that they used to enjoy. It might have been difficult to accept that life would go on and that they could be happy but participating in activities was an essential action to move forward in the process of transformation." (p. 300 - Results)	Genest, C., Gratton, F., O'Reilly, T., Allard, É., & Maltais, N. (2021). Emerging Despite the Indelible Wound: A Grounded Theory of Family Transformation Following Adolescent Suicide. *Journal of family nursing, 27* (4), 295–303. https://doi.org/10.1177/10748407211006183
George, M.	"I have worked with a wonderful therapist to navigate through the dark waters of my mother's decision to end her life and am finally able to feel slightly less guilt than I used to about admitting the chaos that left along with my mother has been beneficial." (p.9)<br><br>"I have now worked for the South Texas Veterans Health Care System in Out Patient Mental Health for two years and have enjoyed my time with the veterans I serve as well as with my Nurse Manager and coworkers. My mother was a veteran and being able to assist with veterans in crisis has helped to fill a bit of the void left. Perhaps I can help to prevent another daughter from experiencing the same tragic life event I did."	George, M. (2023). Flipping up the board: Losing my mother to suicide. *Archives of Psychiatric Nursing, 47* , 7–9. https://doi.org/10.1016/j.apnu.2023.10.003
Ghetti et al.	"As a musician, Jeremy had always experienced singing as coming from a "happy place in [his] soul and heart." After his son died, he was not sure that place existed anymore. Though it was difficult, on the first marking of his son's birthday following his death, Jeremy sang the song "In Loving Memory" by Alter Bridge (2004) for a heartbeat re- cording: "That song spoke to me so much, I really felt I needed, I wanted to and I needed to record that song." On this challenging occasion, the song acted as motivation for Jeremy to connect with his own musical resources. It meant a lot to Jeremy that he managed to express himself musically at such an emotional time and could constructively use music in his grieving process." (Findings, pg 371)<br><br>"Jeremy perceives that it is difficult and awkward to talk to people about what happened with his son and share his own related emotions, but the songs serve as a "jumping off platform" to do so. The recordings express the gravity of the situation and help people relate without requiring Jeremy to go into detailed descriptions. Jeremy "quickly goes to [his] phone" to share the recordings with new people he meets and finds that people can "grab onto" the music and be impacted by it, without needing to have the full story each time. Jeremy is able to share and convey deep emotions without over- whelming himself or the other person, and thus he can more genuinely forge a connection with others." (Findings, pg 371)	Ghetti, C. M., Schreck, B., & Bennett, J. (2024). Heartbeat recordings in music therapy bereavement care following suicide: Action research single case study of amplified cardiopulmonary recordings for continuity of care. *Action Research (London, England), 22* (4), 362–380. https://doi.org/10.1177/14767503231207993
Gordon, E., & Mcelvaney, R.	"Their grief was so intense it paralysed them. One mother described wanting to get professional help but being unable to initiate this, appreciating the outreach nature of the liaison service, "because there was no way I would have got up out of the chair and gone to see someone, you know, I wouldn't have. You are vulnerable."" (p. 400 - Findings)<br><br>"In the midst of this intense grieving process, the participants described feeling a burden of caring for others. According to a mother, "The weight on your shoulders... you're a mother number one"; and an aunt, "I felt I had to take on everyone's pain". One mother described how she supported her son's friends, "... got them to talk about the good things in their lives, you know and to see that it wasn't all... I mean I was concerned for those young lads". They sought professional help for family members as they witnessed signals such as denial of the suicide, and signs of distress. (p. 400-401 - Findings)<br><br>"One mother described how her husband found "peace" in the garden, another described how the visiting professional (liaison worker) engaged with her husband around his work interest, "She just interacted with my husband and chatted about the farm and... you know, normal things... that made him feel at ease". She went on to describe how, without discussing it directly, he overcame his initial reluctance to attend a peer support group" (p. 401 - Findings)<br><br>"Some participants described modelling help seeking and demonstrating the benefits of support and counselling to others, as one mother noted, "Yes, and it is not a lone act, it is kind of by walking the walk and I am showing the others that there is somewhere to go"." (p. 401 - Findings)<br><br>"Participants were aware of their own desire and that of others to block out the memory and emotions associated with the suicide but were also aware that this was unhelpful. Thus, they took responsibility for keeping the memory of their loved one alive for everyone, which took different forms such as displaying photographs, organising a memorial activity, or ensuring that the deceased was incorporated into conversation." (p. 402 - Findings)<br><br>"A mother spoke of how her strong religious faith helped her cope with the distress and to make sense of it all, bringing her some spiritual relief, ... my faith never waned, you know, I didn't get angry with God or anything, because some people will... I would have a crucifix or rosary or something in my pocket and when things would get bad in my head and I would be out for a walk I would squeeze on it and say some prayers and it never failed to lift me out of it." (p. 403 - Findings)	Gordon, E., & Mcelvaney, R.. (2022). Directing from the shadows: Women's experiences of male relative suicide bereavement. *Journal of Family Therapy, 44* (3), 396–407. https://doi.org/10.1111/1467-6427.12388
Goulah-Pabst et al.	"Sarah lost her younger brother in 1986 when she was 23 years of age and embarking on a career in education. "It really killed any social life I had because I didn't want to do anything...I didn't have the energy ... my social life was in a holding pattern."" (p. 777 - Results)<br><br>"Sasha lost her mother to suicide in 1985, on the second attempt, when she was a 19-year-old college student. Her mother suffered from cancer and the prognosis was not good. "I cried a lot...I never went to class again. I just retreated into myself which was pretty deep."" (p. 777 - Results)<br><br>"Doggo was 41 when he lost his brother, a doctor, to suicide after a protracted battle with mental illness in 2004. "I went into a pretty steep depression. I withdrew." Roye, a therapist, was 57 when her son, a doctor, took his life in 2001. "I really didn't want to see anybody because I knew ... then everybody hugs you and you all start crying ... it was too overwhelming to go anywhere where people would know me and rush up to me."" (p. 777 - Results)<br><br>"Ernie was a religious leader, living a very public life requiring him to travel and preach. "It took me several months to kind of get back to connecting with people again. I just withdrew from things for a few months."" (p. 778 - Results)<br><br>"Chilli, having responsibilities in the home and at work found the need to not only retreat from her social milieu but "Life just kept going on ...I just kept on doing my job and doing my whatever", to maintain equilibrium." (p. 778 - Results)<br><br>"Sarah, a budding educator, had no choice but to start her new job. "The funeral was on Monday, Tuesday I went back (home), Wednesday was the first day of this job at this school." (p. 778 - Results)<br><br>"Marta was 47 years old when she lost her husband to suicide in 2005. She had a secure and beloved life in a beach community in California when her loss occurred. "All of a sudden I found myself in northern New Jersey, the place that I wanted to get the fuck out of since high school ...I'm with my parents...I found jobs, I worked in the city, my parents looked after my son". (p. 778-779 - Results)<br><br>"Anita, married to Ernie and living the public life of a pastor's wife found that, "I was really pulled back into life very quickly" due to the obligations of church events" (p. 779 - Results)<br><br>"Beth "just kept moving forward ...I kept working and saving money and planning my move ...I kind of went through in a fog."" (p. 779 - Results)<br><br>"Jon was 58 when he lost his nephew to suicide after a troubled childhood. "My work is very public so it's something I can't hide from ... (I had to) swallow and push on."" (p. 779 - Results)<br><br>"At the age of 78, Rose lost her husband (Chilli's father) to suicide. "I continued to entertain our group of friends which was really like family ... we didn't talk about it. We just carried on. I stayed busy." Keeping up conventions, carrying on and maintaining our social reality allows the loss survivor to avoid questioning conventions and to escape realizing how arbitrary social order, and the loss of it is" (p. 779 - Results)	Goulah-Pabst, D. M. (2021). Suicide Loss Survivors: Navigating Social Stigma and Threats to Social Bonds. *OMEGA - Journal of Death and Dying, 87* (3), 769-792. https://doi.org/10.1177/00302228211026513

Goulah-Pabst et al. Continued	""Athena's loss of her father propelled her into a new role. "I was just showing up as a person who was full of gratitude and acceptance and strength ...I felt that I needed to be that for my mother and my brothers." [...] Athena remembers, "When I look back at the photos, I have on beautiful dresses, my nails are done." Goffman (1959) suggests that the managing of emotions includes the "consistency between appearance and manner" (p. 25). In Athena's case, her beautiful dresses and well-manicured nails helped her to exude the strong person she wanted to appear to be to her social milieu. When Athena played the part of the strong sister, she was impressing on those around her that she felt as she appeared to feel." (p. 779-780 - Results)<br><br>"Participants shared that private therapy positively affected them. "The therapist saved my life." Bob. "That young man helped me ...really helped me a lot." Rose. "I don't think I would have survived what I went through without her." Beth. Anita and Ernie began counseling immediately after their son's death with his private counsellor." (p. 781 - Results)<br><br>"Jon, Doggo and Ernie turned to their religious beliefs and prayer to reassess their lives and losses. Doggo, after six months of losing his faith, "started attending Calvary Community Church" where he embarked on a spiritual life and social activities. Jon belonged to the Unity Church movement. "I went to my minister (immediately), to my spiritual leader and to two prayer chaplains... and that gives me a lot of support." (p. 782 - Results)<br><br>"Getting back to one's life played a large part in Bob's recovery. "I could say getting back involved with my work ... getting engaged in life again ... and being present in things that I really love to do, that made life better again."" (p. 782 - Results)<br><br>""Participating in the support group ... took a lot of pressure off." Chilli found that she "had to be in a protected group to talk about... the details." She looked for "a specific group related to suicide."" (p. 782 - Results)<br><br>"Sarah finds that "my involvement in suicide loss survivor groups... has been very healing for me ... has been a springboard to ... being more involved socially" because "it's a place where I can belong". Sasha "called AFSP (American Foundation of Suicide Prevention), they were having a walk ...I volunteered ... the second I got there my life changed ... people were talking about suicide ...I could say my mother killed herself... it felt so good because I was helping."" (p. 782 - Results)<br><br>"Roye was told about Didi Hirsch's (a mental health services and suicide prevention center) eight-week grief group. "I was with eight or nine people that had experienced a suicide (loss)... going once a week for eight weeks helped me survive ... we could talk about things you could never talk about with other people." " (p. 783 - Results)<br><br>"Anita went to a convention of Compassionate Friends (a grief group for parents) nine months after her son's suicide. "There was a group of about thirty people who had all lost children by suicide ... one of the most meaningful events or group things that I have gone to." Anita found that the "significant sharing" helped her realized "you're not alone". Her husband Ernie had the same experience. He found safety in groups, "to talk about that with other people ... let's get it out ... let's talk about it ... let's be open!"" (p. 783 - Results)<br><br>"Beth "gradually started to volunteer... as a way to stay connected to the loss." She found that "doing the AFSP events... these other people can understand ... the grief is very unique ... that's definitely been very helpful." Athena "started going to two groups every Saturday." She has found that "out of everything I would say the groups have been, I mean just an absolute godsend ... the friendships... the comradery ... it's empowered me."" (p. 783 - Results)	Goulah-Pabst, D. M. (2021). Suicide Loss Survivors: Navigating Social Stigma and Threats to Social Bonds. *OMEGA - Journal of Death and Dying, 87* (3), 769-792. https://doi.org/10.1177/00302228211026513
Goulah-Pabst et al. Continued	"Twelve of the fourteen survivors interviewed participated in support groups specifically designed for suicide loss survivors and had overwhelmingly positive experiences." (p. 783 - Results)<br><br>"Athena's positive experiences with suicide loss survivor groups and the many deep friendships she developed through the groups inspired her to "have the conversation with civilians." Her life's mission "has now become to erase the stigma." Athena puts her experience to work in her writing to "normalize grief and to make grieving mainstream.""(p. 783 - Results)<br><br>"Some participants have gone on to become active leaders in suicide loss groups (Sarah, Athena and John) and in the American Foundation of Suicide Prevention (Sasha, Sarah, John, Roye, Beth, Doggo, Anita and Ernie). Some have started non-profits of their own (Roye and Athena) and many continue to attend meetings for suicide loss survivors to give hope to the newest survivors." (p. 783-784 - Results)<br><br>"Roye, as a therapist, continues to work with AFSP and Didi Hirsch Mental Health Services. "I met some wonderful people through the organizations that I never would have met in a million years."" (p. 784 - Results)<br><br>"Marta feels strongly that her "goal is to become a grief therapist". Through engaging in diligent selfcare in her search for relief, Marta "made the decision to become an optimist", studying the psychology of happiness, practicing yoga, meditation, talk therapy, group support, exercise and reclaiming her beloved beach community." (p. 784 - Results)<br><br>"Sasha has embarked on the production of a documentary bringing the family and friends of her deceased mother together to share thoughts and feelings of her life and death that had never been shared." (p. 784 - Results)	Goulah-Pabst, D. M. (2021). Suicide Loss Survivors: Navigating Social Stigma and Threats to Social Bonds. *OMEGA - Journal of Death and Dying, 87* (3), 769-792. https://doi.org/10.1177/00302228211026513
Groos & Shakespeare-Finch	"People often articulated an early period of seeking answers or trying to fix the past, ''trying to blame someone'' [1], dealing with self-blame, and a gradual move toward greater manageability of remembering and a return to self-care" (p. 13 - Results)<br><br>"Categories or concepts mentioned by fewer participants, but also linked to the suicide bereavement experience of meaning making and personal change, were a return to self-care and engaging in activities to connect with the deceased, their interests, and, more generally, remembering them. Only one participant mentioned spirituality as an important element of the process of remembering and making meaning, while another talked about her personal belief system she did not do so with a particular reference to the suicide death." (p. 14 - Results)<br><br>"The experience of being bereaved by suicide clearly made people value and take greater care of those close to them, which is a facet of the central category of relationships. Others chose to continue to invest in important intimate relationships, and thinking through prior family issues was an important component of dealing with the loss. One participant was about to commence a new job in the welfare sector at the time of the interviews that represented a shift in values and goals. Another person clearly articulated a need to use the loss for greater good. This concept was also linked to a sense of frustration with existing resources." (p. 14-15 - Results)<br><br>"The group also helped to address particularly the concerns of parents in terms of gaining a perspective on the loss from the view of a sibling and the commonly felt experience of hypervigilant parenting expressed by several mothers in the group. For example, ''Every day, checking that the girls are ok, if anything would start going wrong ... you know, you're super vigilant of your other children''" (p. 14 - Results)	Groos, A. D., & Shakespeare-Finch, J. (2013). Positive experiences for participants in suicide bereavement groups: a grounded theory model. *Death studies, 37* (1), 1–24. https://doi.org/10.1080/07481187.2012.687898
Hafford-Letchfield et al.	"The bereaved person's caring role often took precedence over their own immediate needs. One man talked about his mother who deteriorated quickly after the suicide with cognitive decline and died soon after: "*she was diagnosed with Alzheimer's in February and I truly believe, it was bought on by the shock of my brother's death. . . . she went down so quickly, it was shocking* " (female, 62 years, sibling)." (p. 6 - Results)<br><br>"Another participant (male, 78 years, partner) was concerned about their daughter, who at the time of losing her mother by suicide, had four girls all under four including twins of three months. Another commented: "*So, the first few weeks really was me being there for them and cooking and cleaning and doing all the things that she couldn't do. I had to be there and I had to be strong and look after my granddaughter because her mother wasn't capable of doing it at the time, she was in such grief that she couldn't do anything. My daughter couldn't go to work, she was in very, very, deep depression . . .* " (female, 68, grandparent). These intense caring responsibilities sandwiched between ageing parents and their own children, found participants caught in the firing line. (p. 6-7 - Results)<br><br>"The third theme reflected poignant and significant experiences in the personal journey of the bereaved person in later life. This included reflections about their own future, motivation, mortality, and accounts of help seeking. Some described periods of transformation often coinciding with activism and leadership with their peers through shared lived experience. (p. 7 - Results)<br><br>"One woman living alone (aged 74) was now using psychotherapy. She reflected on the COVID-19 national lockdown and how she had 'always been locked down' since her daughter's death. This was an important time for reparation and she did a 'lot of (positive) sorting and thinking' about her daughter. She observed the potential for a greater understanding of the impact of death following the huge loss of people during the pandemic as positive. She also used a telephone befriender service which she found helpful and comforting and described herself as "*emerging again like a metamorphosis, like a butterfly, but it's a long, long process* ". (p. 7 - Results)<br><br>"Our participants expressed a strong orientation to the importance of the shared lived experiences with others who had lost a loved one through suicide and learning to adapt to such a significant loss. These comprised of transformational moments through helping others. They described secondary gain from this sense of agency and control in one's own life, through interactions with their peers, actively seeking out others with similar experiences and recognising the wider meaning of their own lives within the context of societal responses to suicide. Key organisations were named as hosting and enabling networking and peer support:" (p. 9 - Results)<br><br>"One participant took direct action by campaigning locally and setting up an information point at the station where his son died. Such instances of activism and raising public awareness enabled appropriate support based on direct experience being provided at both the prevention and postvention levels." (p. 9 - Results)<br><br>"Another talked about becoming a mental health first aider, which had transformed her life in a positive way. Other turning points were exemplified in finding new relationships and love such as one man had done at the age of 72 yrs." (p. 10 - Results)	Hafford-Letchfield, T., Hanna, J., Grant, E., Ryder-Davies, L., Cogan, N., Goodman, J., Rasmussen, S., & Martin, S. (2022). "It's a Living Experience": Bereavement by Suicide in Later Life. *International journal of environmental research and public health, 19* (12), 7217. https://doi.org/10.3390/ijerph19127217

Heffel et al.	"Many of the changes described by interviewees related to changes in their behavior. This includes changes in behavior following the suicides, as well as behavioral change attributed to new learning experiences. In some cases (variant), the primary behavior was withdrawal, as one student describes, "I just didn't want to be around anyone." The behavioral withdrawal ranged from finding it hard to return to school to missing several weeks of classes." (p. 292 - Results)<br><br>"Interviewees also described positive changes in their behavior (typical), such as intervening when witnessing bullying and becoming active in suicide prevention (variant). One interviewee noted, "it gets you to wake up and pay attention to your friends and don't ignore a text that they're upset...awareness is essential that could save somebody's life whether you know it or not."" (p. 292 - Results)<br><br>"Interviewees described shifts in philosophical or spiritual views (typical). For some, this meant attending church more often, or new appreciation for life and other existential concepts. For others, it strengthened their existing belief system, such as, "now I feel like the skies have opened and I'm more free because doing drugs and having so much stress and always trying was hard and then once this happened I was even closer to God, which opened everything up for me."" (p. 293 - Results)<br><br>"It was typical for interviewees to describe feeling closer and more connected to friends and family, particularly fellow students and community members. As one student states, "the only good thing is that it brought everyone together." Another student described a desire to decrease negative behaviors to increase connection with family members, "It makes me think before I do stuff. Like I was always rude to my family, but it makes me want to spend more time with them and just be a better person to everybody." Tension in relationships with family or friends was a variant response." (p. 293 - Results)<br><br>"Regarding online reminders of the deceased, one interviewee noted, "it would have been better for people to block or remove him rather than keep him as a friend and seeing people keep putting stuff on his page, keep putting more sadness that you're going to see over and over again" One interviewee described deactivating his/her own account to avoid seeing reminders of the deceased, but subsequently felt socially disconnected." (p. 294 - Results)<br><br>"Students sought support from family and peers (typical), online (typical), and from mental health professionals (variant). Interviewees also mentioned it was helpful to provide support to others (variant). They reported feeling support from viewing the responses of other students online, like "everybody actually cared for him and was there for each other." In person, they described feeling more comfortable seeking support from peers who had a similar relationship to the deceased and felt most understood by people with similar experiences" (p. 294 - Results)<br><br>"Interviewees described emotional, behavioral, cognitive, and online activities they engaged in to avoid the grief reaction. Behavioral avoidance was variant and ranged from minimal disengagement (i.e., low motivation to clean room or attend class) to missing 3 weeks of school. Cognitive avoidance included preference to avoid thinking about the situation and was variant among interviewees. Similarly, emotional avoidance was a variant response style in interviewees. Avoidance of interaction online or via SMS text was rare, but was salient to the interviewee(s) discussing it." (p. 294 - Results)<br><br>"The process of coming to terms with the death and realizing the loss was a rare response. Attending the gravesite, going to the funeral, and visiting the Facebook page of the deceased were identified as activities the interviewee(s) engaged in during their process of realizing the death." (p. 294 - Results)<br>"Interviewees maintained a connection with the deceased by talking to the deceased and visiting the grave (variant) and communicating to the deceased online (typical). Online communication included viewing pictures, writing messages to the deceased, and acknowledging the deceased's birthdays/anniversaries . As one student described, "when I miss [the deceased] I can read all the posts and pictures of the times we shared."" (p. 294 - Results)<br><br>"It was typical for interviewees to preserve memories and celebrate the deceased's life. This included sharing information and memories on the deceased's Facebook page and creating an online memorial site (typical), attending student organized school events (variant), and/or attending the deceased's funeral or gravesite (rare)." (p. 294 - Results)	Heffel, C. J., Riggs, S. A., Ruiz, J. M., & Ruggles, M.. (2015). The Aftermath of a Suicide Cluster in the Age of Online Social Networking: a Qualitative Analysis of Adolescent Grief Reactions. *Contemporary School Psychology*, *19* (4), 286–299. https://doi.org/10.1007/s40688-015-0060-z
Honoré et al.	"The participants reported on both the inner processes enabled by beliefs, values, and a relationship with a higher entity, as well as a more concrete and grounded dimension of spirituality. Additionally, most of the participants were accustomed to being in nature, and some had become even more attuned to nature since the suicide of their loved one. They visited natural places such as the sea and mountains, especially for hikes. Some people tended to their gardens or brought flowers to the graves of loved ones who died by sui- cide. Being in nature activated their senses and helped them feel less overwhelmed by their thoughts. Nature also served as a reminder of the power of life, which continued despite the suffering." (Results, pg 6)<br><br>"Moreover, for several participants, their grief was enacted through their bodies. This embodied expression of grief took the form of singing, walking, breathing, etc. One participant associated walking with meditation/: "I find it really healing. It takes up a bit of the meditative side." [P10]. Another participant spoke of the importance of walking in his rebuilding process: "Physically, it gets you going again." (Results, pg 6)<br><br>"Finally, most participants mentioned the importance of spiritual practices and approaches in their grief process. These practices could be individual, such as meditation, personal prayer, sophrology, relaxation, qigong, etc. They also included collective forms for some participants, such as the celebration of mass, mantra chants, Freemason meetings, yoga, and times of sharing around the Bible. Several participants took part in a spiritual retreat or pilgrimage, which was a time to review and take a step back from their own lives, helping them to make sense of them: It's a retreat where you review the life you've had, but in the light of God. So it brought me a lot of things, and I really came out of it at peace." (Results, pg 6)	Honoré, F., Lestienne, L., Vieux, M., Bislimi, K., Chalancon, B., & Leaune, E. (2024). "A sign that I am not alone": A grounded theory-informed qualitative study on spirituality after suicide bereavement. *Death Studies* , 1–12. https://doi.org/10.1080/07481187.2024.2355250
Hultsjö et al.	"During the immediate period after the suicide, the survivors felt shocked, as if they were living in a different world. This period mostly consisted of carrying out practical chores, such as dealing with housing organizations and organizing the memorial service. Survivors found this both healing and burdensome. During this time many survivors had difficulties eating and continuing to live their ordinary lives. It was common that survivors suffered with mental health problems and were on sick leave, but they still had to handle the situation from what they described as "the absolute bottom."" (p. 5 - Findings)<br><br>"Eventually they started to feel grief and had to deal with many emotions. However, even if the grief was constantly present, it changed over time. Being forced to live with grief instead of living in grief was perceived as the goal. A quick return to everyday life was perceived as healing. Getting started with training and exercise and talking to friends or support groups of other survivors was found helpful. 'A duty; you have to do what you have to do. Monday I was away, Tuesday I worked, and I have worked all the time since then. The job is the most normal place. I functioned there; then you could forget it for a little while.' (Participant 10)" (p. 5 - Findings)<br><br>"The potential for moving on was improved when they had the opportunity to say goodbye. Attending funerals and memorial services was valuable because they felt the relative had a dignified end. Some of the survivors found support organizations helpful. These organizations were available around the clock, and many survivors called them for support. The survivors described how they afterwards felt compelled to do something for others in the same situation. 'I joined a support group... The longer I was there, the more I could do for others. Because he should not have died in vain.' (Participant 9)" (p. 5 - Findings)	Hultsjö, S., Ovox, S. M., Olofsson, C., Bazzi, M., & Wärdig, R. (2022). Forced to move on: An interview study with survivors who have lost a relative to suicide. *Perspectives in psychiatric care*, *58* (4), 2215–2223. https://doi.org/10.1111/ppc.13049
Hunt et al.	"All participants mentioned addictions or compulsive behavior as a way of coping with their pain. Six participants discussed their drug and alcohol abuse as a coping method. One discussed his compulsion with masturbation and sex after the death, one his fears of seeing himself become a hoarder like his deceased brother, one her binge-eating to cope with her emotion, and another about how she has seen herself, at times, neglect her family in work and religious responsibilities." (p.338-339 - Findings)<br><br>"Each participant mentioned times of when they felt alone or they withdrew from some of their support system. Participant 6 said, "For 10 years, I didn't talk about my brother's death. I kept it inside and it nearly killed me." Participant 5 discussed her isolation within her own family, "I felt so alone... and [my husband] didn't know how to help because I didn't stand up every day and say, hey, this is how I'm feeling and this is what I want you to do."" (p. 339 - Findings)<br><br>"Each participant mentioned their loved one's death as a signifcant and motivational factor in their current life. Four of the participants reported returning to school to become teachers or counselors specifcally to help others that struggle like their loved one; the other two used their grief to help others that are bereaved. Participant 6 said: "I became a therapist because I wanted to help people like my brother and like me."" (p. 341 - Findings)	Hunt, Q. A., Young, T. A., & Hertlein, K. M.. (2019). The Process of Long-Term Suicide Bereavement: Responsibility, Familial Support, and Meaning Making. *Contemporary Family Therapy*, *41* (4), 335–346. https://doi.org/10.1007/s10591-019-09499-5
Hybholt et al.	"The husband had lost meaningful direction in life, as he could no longer pursue the concern as a supportive father for his son. [...] From the mother's perspective, she had not only lost her son and his participation in her imagined later life, she had also lost her well-functioning, happy husband. As the conduct of everyday life is negotiated in interaction with co-participants, her husband's condition significantly impacted her daily life. Their son's suicide had replaced their shared concern for his wellbeing with a daily struggle for their own existence. It was in the wife's interest to be concerned about them both and their adjustment to their broken notion of late-life living. She revealed that she would not be able to bear to lose him as well. Furthermore, she also wanted to resume the ability to fulfill her role as a useful mother and grandmother" (p. 5-6 - Findings)<br><br>"The death by suicide changed the mother's social self-understanding; she no longer experienced herself as the same strong person. Her decreased health affected her possibilities to act as wanted toward her remaining descendants. Thus, her notion of late-life living was broken, and her altered state meant that her personal concern of motherhood was no longer natural. Indeed, it had become a struggle in her conduct of everyday life with her other children and grandchildren." (p. 6 - Findings)<br><br>"They ruminated over signs that seemed obvious in hindsight. They searched for information about the life of the deceased through dialogue with people in close contact with them. This retrospective inquiry was often combined with seeking general knowledge about suicidal behavior or mental disorders through professionals or literature. Pursuing the meaning-seeking concern completely consumed some participants' mental activity. Still they resumed daily activities such as housekeeping, leisure activities, and volunteer work, which could lead to other people praising them for coping well. However, from their perspective, nothing was the same, regardless of seemingly returning to regular activities." (p. 6 - Findings)<br><br>"Others kept seeking meaning to attempt to adjust to their broken notion of late-life living. For example, a woman, aged 72, whose husband unexpectedly took his life 5 years earlier: [...] I have visited a clairvoyant twice. . . What I wish to know is why he did it, but he doesn't really want to talk about it, the clairvoyant says. so I will go back to ask again and see if I might learn more [...] This bereaved spouse was convinced it would be in her own and her granddaughter's best interest to be able to forgive the deceased. It had not been possible because they were unable to understand why he died by suicide. She pursued this concern by planning a third visit to a clairvoyant, even though her late husband had refused to answer the "Why?" question at earlier afterlife meetings." (p. 6 - Findings)<br><br>"The participants kept their memory alive by incorporating regular memorial activities into daily living at home, such as talking to a picture of the deceased or lighting a candle. They also pursued the concern outside the home at various formal occasions; for example, by mentioning the deceased in speeches, ritual visits at the graveyard, or celebrating the deceased's birthday with relatives and friends. Others shared stories and knowledge about the deceased, ensuring that their history propagated." (p. 7 - Findings)<br><br>"Most participants remained silent to avoid bothering other people with their concern. More than half opted to participate in activities provided by the two non-profit organizations who offered professional counseling and peer support to people bereaved by suicide." (p. 7 - Findings)<br><br>"The concern of regaining life despite loss was characterized by organizing everyday life by balancing mourning and taking part in daily living (i.e., not mourning). [...] Others participated in grief groups or voluntary organizations for those bereaved by suicide. Some participants had to prioritize caretaking activities for family members who needed their attention. They felt that fulfilling their obligation for a loved one's wellbeing helped them briefly set aside their own grief." (p. 7 - Findings)<br><br>"One mother who had unexpectedly lost her only son 7 months earlier threw herself into housework, even though she had severe hip pain and was waiting for surgery: I have really cleaned! ALL bookcases. Every corner, the bedroom closet, the dressers. I have practically taken apart the kitchen and washed it down. I set myself the goal to stay busy, in particular when my husband was out. [...] This mother attempted to regain her life despite the loss, using exaggerated housekeeping as a way to survive the intense grief and reasoned that it was a way to achieve good late-life living" (p. 7 - Findings)	Hybholt, L., Berring, L. L., Erlangsen, A., Fleischer, E., Toftegaard, J., Kristensen, E., Toftegaard, V., Havn, J., & Buus, N. (2020). Older Adults' Conduct of Everyday Life After Bereavement by Suicide: A Qualitative Study. *Frontiers in Psychology*, *11* . https://doi.org/10.3389/fpsyg.2020.01131

Hybholt et al.	"The peer-led support group was perceived as a transformative space because it enabled participants to process the change and find new ways to come to terms with their grief. It also helped them handle the expectations and reactions of people in their social network 'outside' the support group. For example, Alexis describes how she often had to take on a role in daily living, pretending she was doing fine, but felt devastated inside: Alexis: I think I would be able to get an Oscar statuette for best acting performance. Because you go on and live and manage your life. You manage your life and you play some character, but in the peer group the masks drop completely. They are not needed at all among peers (Danish focus group 2)." (p. 7 - Results)<br><br>"The peer-support group was described as an empowering space where participants could disclose and display their grief, which helped them handle societal expectations in<br>their daily lives. They learned to understand their grief by listening to and comparing themselves with the narratives of peers, for example, how they dealt with needs and challenges in the aftermath of suicide. They learnt not only how to process their grief by re-telling their own stories, reading books and listening to podcasts recommended by the group, but also how to keep the memory of the deceased alive without being overwhelmed by it."(p. 7 - Results)<br><br>"They experienced the group as a non-judgmental space, which confirmed that everything could be shared; no feelings, reactions or thoughts were wrong or unacceptable. Further, it was also a space that provided the opportunity to openly deal with the grief, which they often had to conceal in their daily lives. [...] Ashley: When you are in that space [the support group] where you have the opportunity<br>to deal with your grief, where you can have your grief and you enter this space of grief. Not that you put on a facade, but you simply do not have room for your grief in a working life—or at least I do not in mine [. . .] To me, what has been important is that you are active with the organization, and then you sort of withdraw and go back to your 'normal' life where your grief is a bit of a taboo. Here you need to live life the way you always have and then kind of be allowed to return to your grief." (p. 9 - Results)	Hybholt, L., Higgins, A., Buus, N., Berring, L. L., Connolly, T., Erlangsen, A., & Morrissey, J.. (2022). The Spaces of Peer-Led Support Groups for Suicide Bereaved in Denmark and the Republic of Ireland: A Focus Group Study. *International Journal of Environmental Research and Public Health, 19* (16), 9898. https://doi.org/10.3390/ijerph19169898
Jackson et al.	"During the period of the suicidality of his uncle, as the only remaining male in his family, Joseph felt he had responsibility for the well-being of his mother and family and felt he had to step into the role of 'man of the family'. Thus, even at his early age, he dealt with agencies at the time of William's suicide." (p. 497 - Method)<br><br>"In the initial post-suicide period, Joseph became quite isolated, and he described avoiding engaging with people, even non-verbally. 'That image of him ... stayed with me, that stayed on my mind for a period of six or 12 months after. Every time I looked at a person, that was what I saw. It was such a horrendous sight because every time I talked to someone it would bring it back to that experience. So I just ended up walking around, looking down, not engaging in eye contact, not looking at people. That was how I sort of dealt with it. It wasn't obviously an ideal way to deal with it but that's what I had to do to get by, through my daily functioning.'" (p. 499 - 500 - Results)<br><br>"Furthermore, as the lone male in the family, even as a young child, Joseph felt deep responsibility for the welfare of his family. 'After that incident my focus shifted to my Mum. I was so incredibly concerned that this would lead to a chain suicide. I didn't think that Mum was going to be able to cope with it. So after it happened it was almost like suicide watch again. I would stand outside Mum's room every night and listen to her sleep, I would listen to her breathing until I knew that she was asleep before I went to bed. She wouldn't know that I did that but that's what I had to do to ensure that she was well.''" (p. 500 - Results)	Jackson, D., Peters, K., & Murphy, G. (2015). Suicide of a close family member through the eyes of a child: A narrative case study report. *Journal of child health care : for professionals working with children in the hospital and community, 19* (4), 495–503. https://doi.org/10.1177/1367493513519297
Karatay & Gürarslan Baş	"It has been observed that in many cases there is a sense of familial guilt due to the actions or inaction of their loved ones. Especially in fathers, the feeling of guilt was more intense. "His father is worse off than me, he's constantly crying, he's increased his alcohol intake. He's suffering from a guilty conscience … . (Case 3)" (Results, p 737)<br><br>"During the interviews, it was observed that the relatives of individuals who had died by suicide showed psychiatric symptoms requiring support, such as social isolation, inability to cope with memories, numbness/inability to cry, episodes of anger, increase in alcohol use, depressive symptoms, talking to oneself, and increase in obsessive behaviors. However, while some were completely unsupported, a small number were able to obtain psychiatric help on their own efforts." (Results, p737)<br><br>"A father who lost his very young son said, "I can't cry, if I could cry I would feel better. I've increased my alcohol consumption to cope with this pain" (Case 6)."(Results, p 737)	Karatay, G., & Gürarslan Baş, N. (2023). Understanding Suicides through the Processes of Bereaved Relatives: A Phenomenological Study: Understanding Suicides. *Journal of Loss & Trauma, 28* (8), 727–744. https://doi.org/10.1080/15325024.2023.2217032
Kasahara-Kiritani et al.	"Thoughts of the suicide intruded on their daily lives, they got emotionally overwhelmed, and they ended up using anticipatory withdrawal, all of which made them feel "my life is out of my hands." In dealing with this suffering, often by chance, they learned striving skills to survive in everyday situations: letting out the emotion, deliberately burying the suffering, interpreting suicide repeatedly, and manipulating difficult situations. Occasionally, participants did not have enough energy to use these skills; then they resorted to recuperative conditioning, such as making themselves feel good, confirming connectedness, or staying in shape. Gradually, the participants started integrating the skills to regain their lives intentionally and as a result experienced longer moments in which they were not suffering. Some reached the point where they were able to destigmatize suicide, appreciate the absence of the deceased, and verbalize the suicide experience without experiencing suffering for a long time. Though life was never the same, it was good. Those families started living a redefined life. However, most participants continued to struggle with not knowing how long the suffering would last or if it would ever lessen." (p. 449)<br><br>"The immediate issue after the event of suicide was the perception that the bereaved person could not complete their ordinary schedules, however desperately they longed to do so. We named this "my life is out of my hands." Participants could not maintain their daily routines, such as going to work, being in therapy, working and child-rearing, housekeeping, owning a business, or studying. Under this theme, participants reported intrusive thoughts of the suicide, overwhelming emotions, and anticipatory withdrawal behaviors. All of these made participants feel "my life is out of my hands."<br>Intrusive thoughts included the reasons and meaning of the death, self-blame, and inability to concentrate. For example, ID4, who lost her husband, said, "If I were not working, I would spend a day, a whole day, thinking, thinking, and thinking again [about Name's death], and it would drive me mad, I guess."<br>Emotions were so devastating they could not verbally express their feelings, such as anger or sadness. They reported that they suddenly burst into tears at work or school, which they considered inappropriate. The anticipatory withdrawal involved becoming mentally unavailable when asked unwanted questions. ID20 was in high school when her father killed himself. She did not talk about her father's suicide because she did not want to embarrass her classmates. She thought that her friends' embarrassment would disturb her school life: "When I told my friends that my father killed himself, they seemed shocked and stunned. I was sad that our conversations ended there. They thought suicide was abnormal. ... I hated seeing others acting like that."" (p. 449)<br><br>"Those who already had someone with whom they could open up, did so. Others had to find someone. Some visited self-help groups, and if the people there confirmed connectedness, they let out the emotion." (p. 450)<br><br>"There were times when participants deliberately buried the suffering. At first, participants incidentally realized that they had forgotten about suicide during certain activities. Then, they started engaging in those activities deliberately when wanting to avoid such thoughts. Participants removed themselves from things that reminded them of the deceased person. For example, ID18 (lost daughter) moved out of the apartment where he had spent a long time with his deceased daughter. Some participants searched for activities to keep busy and concentrate on, such as working, studying, exercising, joining social activities, or having a busy schedule: "During exercise, I completely forget about my son. I can change my mood" (ID17, lost son). Sleeping was another way to shut out the suffering. ID4 (lost husband) said she cried to tire herself and slept as long as she could: "When I am very angry or very sad, I just cry. Cry, cry, cry, and sleep. It's not that I forget everything when I get up, but the next day starts and I need to go to work, which is good. I have liked this."" (p.450)	Kasahara-Kiritani, M., Ikeda, M., Yamamoto-Mitani, N., & Kamibeppu, K. (2017). Regaining my new life: Daily lives of suicide-bereaved individuals. *Death Studies, 41* (7), 447–454. https://doi-org.myaccess.library.utoronto.ca/10.1080/07481187.2017.1297873
Kasahara-Kiritani et al. Continued	"Occasionally, participants had insufficient energy to use these skills actively, so they resorted to passive withdrawal, such as by doing things to feel good, confirm connectedness, or stay in shape. After resting, they eventually regained the energy and started learning striving skills again. We named these behaviors "recuperative conditioning."<br>Feeling good happened on occasion, such as when engaged in a hobby. Participants reported surprise when this happened. It seemed to help the participants to feel good physically, psychologically, and emotionally to restore themselves. ID3 (lost husband) noticed how good it felt to have a breeze stroking her cheeks while riding a bicycle and was surprised that she could still feel good. She had assumed that she could never feel joy in her life again. Once they experienced feeling good, they intentionally repeated the activity.<br>Confirming connectedness, such as in conversations or cooking with colleagues, friends, and family provided a sense of security. Confirming connectedness included the sense of being able to trust others and be open with others. Relationship building or its strength is essential for confirming connectedness, and already having someone close facilitated this sense. For example: "I shared my secret with someone I trust ... because I wanted him/her to understand me and for us to be close to each other. Yeah, that's why I told my worries to them. I was telling them, "I trust you," without actually saying it in words" (ID20, lost father).<br>Participants instinctively decided whether they could build long-lasting connections with different individuals. The criteria for the decision differed across individuals.<br>Staying in shape, such as by adhering to a regular schedule or undergoing medical treatment, helped some recuperate. "I improved my eating habits and started swimming and being woken up by my cat in the morning. All those helped me maintain my health, I think" (ID12, lost younger brother). Psychiatric treatment was one way to stay in shape when they could build a trusting relationship with the psychiatrist: I became mentally sick and that's how I got to know the medical service personnel and peer supporters ... They helped me and, gradually, I could speak about my brother's death. Eventually I admitted that I had been afraid to talk about the matter for a long time; I think ... If I had missed the chance to meet them, my life would have been more difficult. Moreover ... I had both rational thoughts and strong feelings of guilt that I had killed my brother. The inseparable mixture of thoughts had tormented me.(ID7, lost younger brother)" (pp. 450-451)<br><br>"Dialogers let out their emotion and often listened to others. Dialoguers proactively absorbed others' ideas and opinions, and repeatedly reinterpreting suicide. For example, ID18 (lost daughter) deliberately buried the suffering before; he had trouble talking to other bereaved families and avoided explicitly discussing suicide. However, he worked for a self-help group for families bereaved from suicide and felt comforted through listening to others' talk, and he began to express his feelings to the self-help group members, although indirectly. ID18 also talked about his daughter's suicide with a stranger when he was looking for a confidante: I don't like talking in a self-help group ... but I also want to speak out ... I dropped by an "izakaya" [a Japanese pub], which I had never been to, I got drunk and told a strange man that my daughter killed herself. It happened a few times ... Being a staff member suits me. I am not good at relating to my experience, but I can talk from an outsider's perspective. Some dialoguers expressed their feelings through lecturing or writing: When I am too panicky and emotional that I am about to cry ... I force myself to write ... I start writing "what is happening right now?" I start writing about myself first. The reason I am in such a state. As I keep writing, readers appear inside me and then I regain control of myself. (ID5, lost son and wife)" (p. 451)<br><br>"Many mood changers used deliberately burying the suffering simultaneously with recuperative conditioning through hobbies. ID16 (lost husband) relocated because she was afraid of rumors spreading and being distracted. In the new city, she was a working single mother and could forget those worries. She maintained her wellbeing by watching and enjoying her children's growth for 10 years." (pp. 451-452)<br><br>"Making meaning of the death for the bereaved individual was another pathway to destigmatizing suicide. ID3 (lost husband) took her husband's suicide death as an example of a social problem and started working for other bereaved families who lost their relative to suicide due to similar social issues. She gave meaning to her husband's death by thinking that supporting bereaved persons was her mission. This mission helped her to move past the persistent question of why he killed himself and whether suicide is a sin. When participants could fully convince themselves that suicide was not a sin, they began to make meaning of suicide and their lives. Participants who did not make meaning were still struggling." (p. 452)<br><br>"Our participants described how they sought to live a life that was more than just dealing with routine activities, and how they struggled to regain their life, although their life was a new one. This study is an important attempt to elucidate such actual coping skills after the suicide of their relative to maintain their daily routines and further regain their daily lives" (p. 452)	Kasahara-Kiritani, M., Ikeda, M., Yamamoto-Mitani, N., & Kamibeppu, K. (2017). Regaining my new life: Daily lives of suicide-bereaved individuals. *Death Studies, 41* (7), 447–454. https://doi-org.myaccess.library.utoronto.ca/10.1080/07481187.2017.1297873
Kaur H & Singh	She had started a tiffin service and her younger daughter has started her own boutique as she was well versed in stitching. Her younger son has started working in a shop part time. "My children are helping me, and that is heartwarming, I am not alone" (Results 486)	Kaur, H., & Singh, A. (2023). Six Thinking Dupatta Skills for Problem Solving: Case Study of Suicide Prevention of Wives in Grief. *Journal of Creativity in Mental Health, 18* (4), 477–492. https://doi.org/10.1080/15401383.2021.2000910

Kaur R & Stedmon	"Suicide-loss-survivors reported shutting out traumatic grief for up to many decades, which protected them from experiencing their trauma. The drive to do 'something' in order to survive was instinctual and not always conscious. Survival methods included throwing oneself into study, work, burying grief and taking on a confident persona" (p. 62 - Results)<br><br>"I often think . . . have I grieved properly for him because a month after I threw myself into a course and I've been at uni' ever since, this is my fifth year of study . . . have I thrown myself into studies to avoid really grieving for him? I don't really know if I have. And then I worry if I didn't grieve back then, will I be grieving forever? [Stephanie]" (p. 62 - Results)<br><br>"Sienna described attempting to correct the wrongs of her father and in this process, soothing herself by ensuring that her children know how much they are loved. 'I tell the boys all day every day how much I love them and they can have as many kisses and cuddles as they want because I can do it and I want them to know that I can do it. I won't be . . . choosing to go anywhere . . . I think that sense of security is perhaps as much for me as it is for them'" (p. 64 - Results)<br><br>"Three suicide-loss-survivors expressed some (unintended) positive experiences resulting from their respective losses. For example, Carlie expressed how her father's suicide strengthened her familial relationships and influenced her to pursue work in the voluntary sector. Whilst Carlie values these aspects of her life, she reported experiencing considerable amounts of guilt for loving a life which she believes resulted from losing her father." (p. 65 - Results)<br><br>"Unfortunately, surviving grief and the secondary impact of bereavement by suicide was even more of a struggle at times when relying upon short-lived relief efforts. 'I . . . sometimes binge on stuff. Which I think is . . . about me trying . . . to make myself feel better but actually doesn't or like a punishment . . . I also over shop sometimes, just to get that buzz of getting something new, I'm trying to put a plaster on something that I can't. [Ellen]" (p. 67 - Results)<br><br>"Carlie accessed an assortment of therapies over the last two decades and had reached a point of contentment with herself; 'I am enough on my own, but it's taken . . . 30years . . . '. Joanne resigned from her job in the last year, hoping to have more fun in life, which was previously lacking; 'After going to Survivors of Bereavement by Suicide [SOBs] and speaking to the grief counsellor, I've been waking up most mornings and saying what am I going to do that's fun for me today' (p. 68 - Results)	Kaur, R., & Stedmon, J. (2022). A phenomenological enquiry into the impact of bereavement by suicide over the life course. *Mortality, 27* (1), 53–74. https://doi.org/10.1080/13576275.2020.1823351
Leaune et al.	"A total of 247 participants (61.6%) reported using social media after suicide bereavement. Facebook was the most frequently used social media (91.9% of the participants), followed by WhatsApp (15.8%) and Instagram (13.0%). Nearly two-thirds of the participants (61.6%) reported their use of social media as beneficial or very beneficial for their grief process. A total of 6.6% reported their use<br>as nonbeneficial." (p.4)<br><br>"Social media were mostly used by the participants to reach peers bereaved by suicide (76.5%) and to memorialize their loved one (58.3%). Nearly half of the participants also used social media to find information on suicide and suicide bereavement (49.4%), and a minority used social media to receive or find counseling (4.0%)." (p.4)<br><br>"Parents bereaved by the suicide of their child were more prone to use social media to contact peers (multivariate odd ratio (OR): 5.62; 95% confidence interval (95% CI): 1.91–16.48; p=0.002) and to obtain information on suicide and suicide bereavement" (p.4)<br><br>"Participants bereaved by the suicide of their partner were more likely to use social media to find information (OR: 3.68; 95% CI: 1.61–8.94; p=0.008) but were less prone to use social media to memorialize" (p.4)<br><br>"People who used social media less frequently in their daily life were more prone to use social media after the death of their relative to find counseling support (OR: 16.27; 95% CI: 2.79–94.95; p=0.002) and less likely to contact peers on social media" (p.4)<br><br>"Younger participants were more likely to use social media to commemorate the memory of their loved one" (p.4)	Leaune, E., Rouzé, H., Lestienne, L., Bislimi, K., Morgiève, M., Chalancon, B., Lau-Taï, P., Vaiva, G., Grandgenèvre, P., Haesebaert, J., & Poulet, E. (2024). The use of social media after bereavement by suicide: results from a French online survey. *BMC Psychiatry, 24* (1), 306–310. https://doi.org/10.1186/s12888-024-05761-9
Lee E	"Theme 1. A Sense of Defeat Living in a Broken World<br>The bereaved family thought they had lost the "present" they had with their family and the "future" that they would experience over the next days. They were spending their days meaninglessly in a reality that seemed to be trapped in a tunnel with no end. They came to realize the finiteness and the futility of life, and in their deep sorrow, they could do nothing. "What are you going to do by being alive? I have no reason to live without him. Even so, I'm really trying to eat and live again because I have a fixed-term job at this city hall . . . My son died, but I want to buy and eat valuable ingredients. Even if I learn anything much, I've lived without acting badly to the other people. Why has it happened?" (Gukjin)." (p.7-8)<br><br>"Theme 1. Struggle to Escape from the Bondage of Memories with a Loved Family<br>As the participants recalled the deceased, they were sucked into an uncontrollable abyss of sorrow; hence, they tried to immerse themselves in work or live busy daily lives. They consciously focused on work and stopped thinking about the deceased, trying not to face pain.<br>"It's okay when I work. Only when I work. It did not last long. Also, I think where my son will come from, but I forget it when I work." (Mija).<br>"No matter how close I am, it is correct to distance myself from other people. For now, I'm only talking with my daughter and my husband. I try to train myself harshly and have a strong heart. When a tough thought comes up, I stop it. I work with my mind focused and don't want to think about anything else. If I think of my son, I may make mistakes." (Mija).<br>"I go to the temple whenever I have time. I bow down 108 times when I feel frustrated because I think of my child. There are times when I just feel frustrated and upset. At that time, after I did it, my feelings subsided a bit." (Eunjeong).<br>"I can't erase my memories with my child. I pray to the Buddha to be with the child in the next life." (Sungmi)." (p. 8)<br><br>"Theme 3. Hope Gained by Responsibility for Family<br>Even though the whole family had suffered a terrible thing like the Blue Army, they were trying to get out of despair while concealing each other's pain, thinking about the family member who would be more affected. They were anxious about losing another family member, and they tried to take better care of each other. They believed they had the strength to endure the difficult time because of the surviving family, and received comfort and support from each other. They were planning their future careers, holding on to new hopes, and gaining the strength to live again.<br>"I'm more concerned about my child's father than myself. Even that day, my husband sank to the floor. He seems to be okay with his work, but he is hurting a lot now." (Mija).<br>"My husband suddenly lost 7–8 kgs. I'm very worried about him. He only keeps thinking of our dead son. He says he doesn't know how to raise our other child. He says he's doing his best right now, but he doesn't know what to do. But we still have a second child, so we try to strengthen ourselves." (Jiwoo).<br>"When my wife was alive, I planned to spend time with her when I retire, but now, I wonder how I will survive my remaining life? I plan to work more. I thought about starting a company based on what I was doing when I retired. When my son graduates, I plan to get my son a job at my company and work with him." (Sungmin)" (p. 9)<br><br>"participants tried to control their negative emotions or sadness by keeping themselves busy in daily life. In Lee and Choi's study [12], the bereaved families by suicide tried to live separately from the deceased through meditation, yoga, or religious activities. They perceive themselves as having to endure suffering by being busy in life, in order to be separated from the pain of suicide in their family [19]. However, it is not desirable for them to be overactive or to engage in maladaptive behaviors such as excessive drinking [19]" (p. 10)<br><br>"In addition, the participants approached new hopes by making plans for the future and had the will to take care of the remaining family members. The bereaved family members try to protect the rest of the family despite suffering, because they fear that one of them may commit suicide like the deceased [12]. The bereaved family members seek a way to comfort each other, strive to play the role of the deceased, oppress the painful feelings, and choose to dedicate and sacrifice their lives for the rest of the family [28]" (p.11)	Lee E. (2022). Experiences of Bereaved Families by Suicide in South Korea: A Phenomenological Study. *International journal of environmental research and public health, 19* (5), 2969. https://doi.org/10.3390/ijerph19052969
Lee E et al.	"Junghwa further explained how her altered sense of priority shaped her behaviors and attitudes toward others and her own self: 'After my husband's suicide, I realized what the most important thing in life was, and I made family a priority. I used to criticize my child instead of trying to understand him when he did not listen to me, now I try to understand my child and see him the way he is." (p. 424 - Results)<br><br>"Our participants expressed their desire to help other recent survivors of suicide, especially those who were not able to receive any help. They felt that they should return the help that they themselves had received from long-time survivors of suicide or support groups by helping others in distress. Junghwa described, ''I received help from other suicide survivors through online and offline support groups, so I want to help other survivors, even those who are unable to attend the support group.''" (p. 427 - Results)<br><br>"Junghwa's goal of making the ''pain stop in our generation'' underlies the logic that dominated the narratives of our participants who described that they wanted to have a positive impact by preventing future suicides in honor of their deceased family member and thus prevent other families and future generations from experiencing their pain. For example, Sunyoung who lost her husband 18 years ago expressed her desire to help: ''I am trying to help prevent suicide because I wish other people to avoid my experience. I also participated in lectures addressed to help children who experienced suicide ideations.''" (p. 429 - Results)	Lee, E., Kim, S. won, & Enright, R. D. (2019). Beyond Grief and Survival: Posttraumatic Growth Through Immediate Family Suicide Loss in South Korea. *OMEGA - Journal of Death and Dying, 79* (4), 414-435. https://doi.org/10.1177/0030222817724700
Lee Y et al.	"18 (42.9%) and 30 (73.2%) parents had clinically significant insomnia and depression, respectively." (p. 5 - Results)<br><br>"1) The psychological pain of parents who have lost an adolescent to suicide is considerable. Clinically significant suicidal ideation, depression, and insomnia were identified in 75.6%, 73.2%, and 42.9% of parent survivors, respectively. 2) The severity of depression of the surviving parents was positively correlated with their insomnia severity, the deceased adolescents' depressive symptoms, and the incidence of traumatic events. Overall, the higher the incidence of traumatic events in adolescents who die by suicide, the greater is the severity of insomnia and depression in the parent survivors." (p. 7 - Discussion)<br><br>"Parent survivors face complex and challenging issues such as stigma, shame, guilt, and anger.12 They regard their offspring's suicide as their failure as parents to raise, nurture, and protect. The feeling of hurt in parents is greater than that experienced by close relatives or friends.12 Therefore, it is estimated that our study reported higher suicidal ideation than other studies because the participants of our study were all parents." (p. 7 - Discussion)<br><br>"Our study confirmed that 42.9% of parent survivors had clinically significant insomnia. Previous studies10,59 also support our findings by confirming insomnia in approximately half of the bereaved families. Many suicide survivors have shorter sleep durations and poor sleep quality.18 Sleep changes during mourning are associated with decreased activity, lifestyle changes related to disengagement from usual activities, and psychological discomfort such as rumination and anxiety due to grief from the loss of a loved one.55 Furthermore, insomnia adversely affects the metabolic, endocrine, and immune systems and increases mortality.13,56-58 Therefore, suicide survivors should evaluate their insomnia to assess their psychological and physical health." (p. 8 - Discussion)	Lee, Y. J., Kweon, Y. S., & Hong, H. J. (2023). Suicidal Ideation, Depression, and Insomnia in Parent Survivors of Suicide: Based on Korean Psychological Autopsy of Adolescent Suicides. *Journal of Korean Medical Science* , *38* (5), e39–e39. https://doi.org/10.3346/jkms.2023.38.e39

Leichtentritt et al.	"they described how they were also responsible for other family members. After their mother's suicide, these responsibilities grew as they now had to take upon the role of mothering their bereaved father and siblings as well: 'He [father] was left with four kids, the oldest being 23 and my youngest sister only 11 ... He couldn't take it – he was devastated, he couldn't take care of himself, let alone us...I made sure that everyone went on functioning, going to school, doing their homework, going to work ...I used to get up very early to get everything organized ... made sure my father left for work.' (Emily)" (p. 558 - Results)<br><br>"Notably, the interviewees' perception of themselves as ''everyone's mother'' is evident in their occupational choices. Almost all are working or have worked in therapeutic or semitherapeutic professions such as nursing, social work, occupational therapy, art therapy, or teaching" (p. 559 - Results)<br><br>"I often don't think I am capable ... Whenever there is something wrong with one of the kids, I tend to give up and let others care for them ... in terms of health, education ... I am not one of those confident mothers. (Julie) (p. 562 - Table 3 - Results)<br><br>"'I'm constantly consulting professionals – they laugh at me behind my back, I know that – but just in case there is something wrong with my kids, I want to be able to nip it in the bud, and not to wait for the major episodes... to catch it when you can still control it.' (Julie) 'There is this big black cloud over our heads – mine and the kids'. I keep worrying about their mental health, checking how they feel, what their moods are ... I worry. With my family history you can't be too careful.' (Laura) (p. 566 - Table 3 - Results)<br><br>"Some interviewees (n = 7) saw their children as needing protection, guidance, and assistance, and so these women assumed an overly mothering role toward their children [...] By contrast, others in the study (n = 5), knowing the devastating consequences of being weak or dependent, stressed the importance of raising independent, self-reliant children who can care for themselves" (p. 567 - Results)	Leichtentritt, R. D., Leichtentritt, J., & Mahat Shamir, M. (2018). Shadows from the past: The mothering experience of women survivors of maternal suicide. *Journal of Social Work, 18* (5), 548-577. https://doi.org/10.1177/1468017316656091
Leichtentritt et al.	"Every Saturday, years after she died, I would drive to Jerusalem to visit my parents and curse her on the way ... 'you moved them to Jerusalem, next to you, just so you could kill yourself'?! ... I would curse her all the way. I hated driving there; I hated the fact that because of her they lived so far away." (p.1109 - Results)<br><br>"Nadav talked at length about the various strategies he employs to maintain a close relationship with his deceased sister: every Friday, he puts flowers on his sister's grave; he has her car and drives it daily; he holds onto the notes and CDs he found in her car, keeps her driving license in his wallet and makes a conscious effort to involve his mother and others who knew her in discussions about her." (p.1111 - Results)<br><br>"I talk with him mainly on his memorial day.... We have a stable structure to the ceremony; it is the same every year ... I am involved in choosing the poems and often help the person who writes and reads.... Every year before that day I find myself thinking about ____ [the deceased] and what he would he have liked. What captures his character the most? What suits him? This annual process makes me think about him. I even find myself talking with him—updating him with all the changes that have occurred throughout the year...." (p.1113 - Results)	Leichtentritt, R. D., Yerushalmi, A., & Barak, A. (2015). Characteristics of the Ongoing Bond. *British Journal of Social Work, 45* (4), 1102–1118. https://doi.org/10.1093/bjsw/bct171
Machado & Swank	"In addition to taking responsibility and ruminating on what she could have said or done differently, Valerie also reported fatigue, difficulty sleeping, difficulty functioning in her career as a teacher, and absence of previously experienced enjoyment in traveling with her husband. With the encouragement of the counselor, Valerie began attending a local support group for survivors of suicide death of a child, and concurrent individual counseling." (p. 631 - Case illustration)<br><br>"As Valerie completed her initial planting of the three flowers, she shared with the counselor family memories, which included narratives of family dinners, vacations, holidays, and celebrations. Valerie explained that the three flowers represented her, her husband, and her son. Valerie placed Sean's flower in the middle between the other two flowers. In the months that followed, Valerie started every session by watering her flowers and discussing moments that she felt Sean's presence in the garden. The counselor facilitated Valerie's healing journey through encouraging storytelling, meaning-making, and developing rituals in the garden. Regularly, Valerie left messages for Sean, such as by drawing a heart on the ground for him" (p. 631 - Case illustration)<br><br>"Valerie reflected on her gardening work often throughout counseling, sharing that gardening served as both a "distraction from her pain and a cure for her pain." As counseling progressed, Valerie reported that she was becoming herself again." (p. 631 - Case illustration)	Machado, M. M., & Swank, J. M. (2019). Therapeutic gardening: A counseling approach for bereavement from suicide. *Death Studies, 43* (10), 629–633. https://doi.org/10.1080/07481187.2018.1509908
Maple et al.	"So we have daffodils and they're our special thing, we have friends, new friends we have met through [support service], they live in the town where Leigh is buried and they go out and put daffodils on his grave because they know what it means to us, so daffodils are our little symbol, and you cling to little things like that. We light the candle that was at his memorial service, we have that and we light that each Christmas day, March the 1st and his birthday, and have that going in the lounge for a little while. We still have several phone calls on his birthday and March the 1st. It is very important that he is part of our lives, there are traditions and it might not work for everybody, I don't know but for us they are terribly important to me they are. Yes, he is still a part of our family, very much so." (p. 55 - Jane and Alan's Story)<br><br>"On learning of their child's death, parents were unsurprisingly, overwhelmed by grief. A period followed where parents recalled feeling disoriented and separated from the day-to-day activities of others. Whether or not the parent anticipated the suicide, they had to incorporate this tragic occurrence into their life. During this initial period the parents had to plan a funeral. After this task was complete, they initiated rituals individually or within the family to create a space where they could spend time with their deceased child, continuing to include them in their daily life." (p. 60 - Findings)<br><br>"Memorials were important to all the parents involved in this study. The ways in which these were held varied from family to family, parent to parent. Some parents planted special gardens in memory of their child, or had special photographs and candles on a mantelpiece in their homes. Louise talked about how she found solace in returning to isolated places on their large rural acreage that her son, Charles, liked to frequent; rock outcrops and bush hideaways. She told how this provided a special connection to Charles, as she knew that she was the only one who was aware of the location of these places." (p. 62 - Findings)<br><br>"While most families spoke of practices that were generally private either individually or within the family, for some it also remained important to keep the child memorialised outside the family. For example, Wanda and her family donated a prize to a music college, as their son Lance had been a keen and talented musician there. While originally Wanda thought it would be important for her to present the prize to the winning student each year, this has not eventuated. She personally presented it once and now other family members share the duty each year." (p. 62 - Findings)<br><br>"Some parents also continued external memorialisation by becoming involved in suicide prevention programs or helping others bereaved through suicide. This allowed the legacy of their child to be continued" (p. 62 - Findings)<br><br>"Every Christmas, [my daughter] and I, we've got this candle that was given, everyone was given one at the suicide support group. It was just a red candle and it's got a Santa face on one side, but we were told every Christmas to light it and think about the person and anyone else who had suicided and that and just light it and so every Christmas when [daughter] comes to get me, or she doesn't come over she expects me to take my candle with me, and we just sit quietly by ourselves somewhere and we light them together" (p. 63 - Findings)<br><br>"Parents gave themselves permission to continue their connection with their child. Paradoxically, they also felt both compelled and restricted by themselves and the community from talking about their suicide-deceased young adult son or daughter (Maple et al., 2010). While rituals provided space for remembering their child, such an activity often remained personal and private." (p. 64 - Findings)	Maple, M., Edwards, H. E., Minichiello, V., & Plummer, D. (2013). Still part of the family: The importance of physical, emotional and spiritual memorial places and spaces for parents bereaved through the suicide death of their son or daughter. *Mortality, 18* (1), 54–71. https://doi.org/10.1080/13576275.2012.755158
Marek & Oexle	"In one case, friends of a participant's deceased son established a commemorative association in his honor, which aimed to educate adolescents about mental health and support-seeking. This initiative, viewed by the participant as a form of "grief therapy" (P12) for the deceased's friends, was cherished as a means to maintain connections with them." (p. 6 - Results)<br><br>"Furthermore, participants detailed challenging situations in their workplace where they felt a lack of genuine support from their supervisors, who seemed to expect a predictable grief process from their employees. These interactions were perceived as dismissive and neglectful of SLS' emotional needs during their bereavement journey: P10: "Yes, so my supervisor already told me to take the time that I needed. But when I come back, I should be, well, ready and consistent, just as an employer would like, fit for work again." P6: "I felt the need to explain to my boss why I was missing work more often. And she had somehow reacted really stupidly. I told her what had happened, that it was suicide. This was about two months later. But she then said that we would need to organize a replacement and that the team would have to coordinate better to make it work. And that was it, basically. It really stuck with me. How cold her reaction was. I'm not even sure if she offered her condolences or anything." "(p. 8 - Results)<br><br>"Echoing previous findings, peer interactions emerged as a valued component of social support. These interactions not only foster a sense of belonging but also help reduce stigma, isolation, and self-blame [34]. Connecting with peers gives SLS hope and empowers them to cope with their grief and the changes that result from their loss [34]. Interestingly, our research suggests that the benefits of peer support extend beyond formal groups, as examined in previous studies [34–37], to include informal networks and connections with others who have experienced similar losses or life challenges." (p. 9 - Discussion)<br><br>"As shown in our study, these norms can shape expectations around the grief journey and coping mechanisms, leading to challenges at work and unsolicited advice by others." (p. 9 - Discussion)	Marek, F., & Oexle, N. (2024). Supportive and non-supportive social experiences following suicide loss: a qualitative study. *BMC Public Health, 24* (1), 1190–13. https://doi.org/10.1186/s12889-024-18545-3
McDaniel et al.	Another identified theme was disruption in their own family members' behavior and roles. Family members left behind after a suicide found disruption in their roles in the family, which eventually lead to disruptions in the roles of other members within the family. For example: "My dad was scared out of his mind and worked three jobs" (Michael); "I had to grow up fast and support my brother and mom" (Dan). Participants described adapting to additional roles and responsibilities at home and in the work environment. These changes were emotional and had an affect with stress and finances. In addition to less organized family gatherings participants recalled behavioral and role changes among various family members. These concerns often affected subsequent family generations. For example, about raising their own children, participants reflected: Now I am extra cautious with my own kids" (Alice); "I will never get over my mom and brother's suicide attempt together, as my mom lived, and my brother did not" (Heather). (p. 1495)<br>Participants often reported wanting to give back by volunteering or donating to non-profit mental health organizations. One participant stated he started a non-profit mental health organization on a college campus. Another participant sought out mental health educational opportunities in their community, eventually attaining the role of an Applied Suicide Intervention Skills Trainer (ASIST). Yet, another participant reflected on her journey to a counseling degree following the loss of her brother to suicide: "I decided to go back to school for counseling. I received the support I needed while taking classes for my master's degree in counseling" (Jennifer). (p. 1496)	McDaniel, B. M., Daly, P., Pacheco, C. L., & Crist, J. D. (2022). Experiences With Suicide Loss: A Qualitative Study. *Clinical Nursing Research, 31* (8), 1491–1499. https://doi.org/10.1177/10547738221119344

Mead	Sam was distraught in the initial weeks after her boyfriend's death. She felt disabled and tasks such as eating and commuting to work took extreme effort. She came to counseling weekly and after 8 to 9 months got a new professional job and moved to the city, where she started to feel better and less in need of counseling. The excitement of a new career and new apartment allowed her to engage more with friends and new coworkers, and she spoke about not wanting her boyfriend's death to be part of her main identity. (p. 159 - Discussion)<br><br>Amy was unable to complete her college coursework after her brother passed away. Her sadness and grief consumed her and left her with little energy for other things. Eventually she graduated, but the length of time it took was a source of embarrassment, and it affected her confidence in securing post graduate employment. This also fueled preexisting depression and anxiety. She expressed feelings of being stuck for several months with no energy to make plans for herself. Being able to remember her brother became an important piece of her identity and an important piece of her career development. After much processing, she enrolled in mortuary school, where she could use her skills and talents to help other grieving individuals. She identified this as a "calling" for her and felt like she was making meaning of her brother's suicide. (p. 160 - Discussion)<br><br>As clients are attempting to explore identity through the areas of work, love, and worldview, many express feelings of resentment because the person's suicide death has prevented them from completing these developmental tasks in their assumed time frame. Many talk about yelling and arguing with significant others at significant others, drinking too much with friends and losing control, and being stuck in a job that they hate. Some clients feel that the emotions related to grief are so intense that they have prevented them from attaining the next developmental goal. Holly describes her brother as being unable to maintain a relationship because he is so angry about the suicide loss of their father. Emerging adults tend to present for counseling when a problem related to relationship satisfaction or career development occurs. (p. 160-161 - Discussion)<br><br>Young grievers speak of anger toward their parent(s) (most often in cases of parent or sibling loss) because they are no longer able to continue as the parents that they knew before. In the case of sibling loss, the young griever can become the forgotten griever. Many are sent the message that they should be strong for their parents; one client stated, ''many people came up to me at the funeral and told me that I needed to be strong for my parents; what about me?'' Emerging adults are forced to maneuver new roles within the parent/ child relationship, relying less on their parents for support than they would have ordinarily received. (p. 163 - Discussion)<br><br>The topic of getting excessively drinking and losing the ability to regulate emotion is a common expressed experience. Kristen said, ''I realized that I needed to come to counseling because I would drink too much and then become hysterical. It was embarrassing.'' Because social normalcy is still very important at this age, alcohol use allows emerging adults to appear normal and also distracts from the pain. The alcohol use tends to be consumed in a peer setting and may serve as a distraction for the short term until the effects of alcohol trigger an intense buried grief reaction. (p. 164-165 - Discussion)	Mead, J. (2020). Competing Developmental Demands Among Suicide-Bereaved Emerging Adults. *Omega: Journal of Death and Dying, 81* (1), 155–169. https://doi.org/10.1177/0030222818764528
Michaud-Dumont et al.	After looking for explanations for the suicide (see first theme), participants questioned their own visions of life and some took concrete action in order to reconstruct their views and get through the mourning process. For example, participant A described how the event had pushed him to study gerontology so he could increase his knowledge about mental illness and make sense of this loss in his life. He also found a job with older adults thereafter in order to get involved with this population and help them. "I'm the only one in my family who said I'm going into gerontology. I want to understand more about what happened. So, with these courses, I have a better understanding of mental illness. It's hard to understand something when you do not have the knowledge, you do not know the theories about it. It has helped me a lot in personal terms too [...] I'll tell you, that helped me get through my mourning [...] I sort of experienced another vision of life. You could say that I was looking for something: a meaning. It made me question things. It brought out a lot of things that I had inside me. You know, philosophize about life and all that. Become more aware of what life is" (p. 5 - Results)<br><br>In addition to the sorrow that is common after all losses, two suicide survivors showed high levels of distress in several domains of their functioning. "I had the impression that I vegetated for months. [...] I was very, very rattled by the whole thing. [...] I never felt like going to my Tai Chi class. I did not feel like it. And I could not concentrate. There were even times when I had trouble reading. [...] I stopped eating. I've gained back two pounds, but I lost 20 after my father died. My appetite is not very good. [...] I was assessed by a psychiatrist, who told me that it was impossible for me to go back to work" (p. 6 - Results)<br><br>They clearly stated that their priority was to take care of their own needs. "So, the way I was functioning, I'll tell you, for the ... first 3 or 4 months, it was clear, it (my own well- being) took precedence. My priority was more ... to take care of myself"(Interviewee A, grandfather). Another had to take care of her mother, who had Alzheimer's disease, which influenced her grieving process. "I asked myself the other day, ... I said to myself, and if Mom had not been there, how would I have gotten through it? It would have been totally different, if you do not have someone to look after 24/7" (p. 7 - Results)	Michaud-Dumont, G., Lapierre, S., & Viau-Quesnel, C. (2020). The Experience of Adults Bereaved by the Suicide of a Close Elderly Relative: A Qualitative Pilot Study. *Frontiers in Psychology, 11* , 2331–2331. https://doi.org/10.3389/fpsyg.2020.538678
Mirick & Berkowitz	"One participant, a twenty-year old woman, described her response to the death, "I ignored everything and started taking care of his best friends." Another participant, an eighteen-year-old high school student, described the months after her friend's death: I needed to do something for him. So, I was like, I'm going to keep an eye on his friends, by caring for them ... I talked to two of his friends a lot ... I was texting his best friend and making sure he was okay. I think that's what kept me from going off, completely shutting down. This drive to help and support others was powerful and created a new sense of purpose for some participants. For example, a 20-year-old college student, whose casual friend died by suicide, described how this experience led her into roles supporting others, "Another thing that I took away was wanting to help, which is why I joined Samaritans. Two of my peers from the school also did it. I think, for some people, mental health became something that they were passionate about." Instead of providing direct support, others applied this passion academically by devoting time to learning more about mental health and suicide through college courses or research projects. Their understanding of suicide and mental health shifted and grew, through their experiences and through reading and exploring the topic more." (p. 344 - Results)<br><br>"Some participants found their perspective shifted over time, as the suicide death receded into the past. Some understood the experience in a different way, with an adult's understanding and knowledge. One participant described a new perspective on priorities that was counter to the culture in his high-performing high school. He learned, "There's more to life than academics. There's also a need for balance in life. I think that hadn't been aware to a lot of kids." Healing and meaning-making occurred from this new perspective." (p. 345 - Results)<br><br>"Another participant described the way that the student leadership group worked to shift the culture following several student suicide deaths, starting with greeting students at the door each morning and holding the door for them. He said: "We would hold doors every Friday. It became something that was really quite impactful. We utilized that and then we added things to it ... We did the surprise donut day. We did a similar thing for St Patrick's day, and just surprised kids. These pop-up events just increased campus climate and make kids feel ... just brightened their day a little bit. We held a day in the gym. We had service dogs. We had all these fun things ... the group did a lot of stuff that focused on campus climate and wellness." (p. 345 - Results)<br><br>"A group identified long-term changes and negative meaning-making following the death. Some made changes in their lives so that they could avoid reminders of the death. This avoidance could last for years. One participant described her response to the suicide death of an "idolized" church youth group member at 16 years old, "I stopped going to that church after that. I never went back. I still haven't gone back ... I cut everything after that happened."" (p. 346 - Results)<br><br>"Participants provided examples of finding a sense of purpose and meaning in supporting the well-being of others (Bartik et al., 2020), particularly those closest to the deceased." (p. 347 - DIscussion)<br><br>"Several described searching for more information on suicide and some strove to support others better through this experience. Others identified a new, deeper understanding of mental illness and suicide. For two participants, this growth and desire to help others led them to volunteering to answer phones at local crisis centers." (p. 347 - DIscussion)	Mirick, R. G. & Berkowitz, L. (2023) After a Suicide Death in a High School: Exploring Students' Perspectives, *Journal of Social Work in End-of-Life & Palliative Care, 19* (4), 336-353. doi: 10.1080/15524256.2023.2256481
Mirick & Berkowitz	"Participants described using the following social media platforms: Instagram (n = 5), Facebook (n = 4), and YikYak (n = 1). Most participants (79.4%, n = 27) said that social media impacted their processing of the death, including 84.6 percent of girls (n = 22), 0 percent of boys (n = 1), and 75.0 percent of nonbinary participants (n = 3). Those who found that social media impacted their experience described this impact as positive (42.3%, n = 11), neutral (34.6%, n = 9), or negative (23.1%, n = 6). Participants in middle school at the time of the death (33.3%) were more likely than those who were in high school (12.5%) to report that social media had a negative impact [$\chi 2$ (3) = 8.545, $p = .036$]. Similarly, 22.2 percent of the middle school student participants felt social media had a positive impact compared to 37.5 percent of high school student participants." (p. 6 - Results)<br><br>"Of the qualitative responses, 92.3 percent (n = 12) indicated that social media impacted their experience. Participants described the use of the following social media platforms: Facebook (n = 10), Instagram (n = 6), Snapchat (n = 5), Twitter (n = 1), YouTube (n = 1), and Tumblr (n = 1). As indicated in table 2, four themes emerged from the qualitative data about the role of social media following the suicide death of a peer: communication (92.3%, n = 12), commemoration (61.5%, n = 7), support and comfort (46.2%, n = 6), and ways that social media made the experience more challenging (76.9%, n = 10)." (p. 6 - Results)<br><br>"Commemorating the Deceased<br>Social media was used to commemorate the deceased, process the death, share memories, and acknowledge the loss (61.5%, n = 7). Social media sites became memorial pages. Participants' social media sites were flooded with photos and comments commemorating the deceased. One participant said, "People posted all sorts of things like 'we miss you' and 'we love you' and 'I'm sorry you had to go this way'—all the cheesy stuff that people say after a suicide—. . . . 'You were in too much pain.'" Participants described being inundated with comments and photos of the deceased via social media: "There's this one picture of him in a football uniform. That was the main picture that was used over and over again."<br>Some participants liked online commemoration. It could be comforting to see the deceased remembered, and they appreciated this public processing. One participant shared, "It was nice to see the people who knew him commemorate him in a way that honored his spiritedness, his humor, and his friendliness." For others, online memorials allowed them to maintain a connection with the deceased. One participant described the months after the death of her friend: "I was writing on it [her Facebook page] regularly as a form of communicating with her." For some, this communication lasted years. One female participant said,"There's a group of 10 of us that still to this day post on her wall every once in a while. It's a way for us to have her included." Students used social media to plan memorials, vigils, or other events. This was particularly helpful if the family did not have a public service or there was no school organized commemorative activity. As an example, one participant said, "People changed their profile pictures to all black on social media as a sign of solidarity and grief." These efforts were primarily viewed as positive, supporting students' grief process.<br>On the other hand, for some participants online memorials elicited strong negative feelings. The constant barrage of commemorative comments and photos was overwhelming, frustrating, or upsetting. One female participant described her reaction: "When I would see posts about people mourning him it made me mad and I'm not exactly sure why; it just made me mad and then [I was like] all right, I can't keep looking at these, because it was post after post." Memorial pages continued for years, with posts on important dates, including graduation or the anniversary of the death. For some, the ability to use social media to memorialize the deceased was appreciated and valued but for others it was distressing and difficult." (p. 8-9 - Results)	Mirick, R. G., & Berkowitz, L. (2023). "Their Facebook posts were going viral": Social media in the aftermath of an adolescent suicide. *School Social Work Journal, 48* (1), 1–18.

Mirick & Berkowitz Continued	"Support and Comfort<br>Some participants (46.2%, n = 6) found social media helpful and supportive, creating a place to find comfort and receive support. One participant recounted, "I downloaded a ton of pictures and looked through my chats when I missed her too much. I also received a lot of support from friends outside of town through social media." Some participants described online messaging from people offering support to others, even students they did not know well. Participants appreciated and valued this supportive caring climate: After three [suicide deaths] in a row, there were definitely a lot of posts on Facebook. . . . There was a lot of "if you need someone I'm here," which was nice. Especially when you're in the middle teenage years, it can be nice if you're struggling to think like oh this person maybe will be there for me if I need it. Another participant had a similar experience: "It was kind of helpful because you knew everybody's there and everybody's looking out for everybody now." Participants described using social media to organize the community, engage in suicide prevention awareness, and spread information about mental health. One participant summarized her thoughts on the use of<br>social media during and after the death of her friend: In a way, social media helped spread awareness about mental health issues in general. I remember everyone posting on their Instagram stories "[school name] Strong" after the incident, or people saying that they were here if anyone, whether they knew them or not, needed to talk. This fostered a sense of community among us students, so I think overall social media played a net positive role in processing this experience.<br>Overall, social media could be helpful in terms of creating a supportive community, a place for distraction or comfort, or a venue for maintaining a connection with the deceased." (p. 10 - Results)<br><br>"Challenges<br>For some participants (76.9%, n = 10), social media made the experience of grief and loss more difficult, impacting their ability to cope and exacerbating distress. For some, the onslaught of continuous content about the death was too much and felt inescapable. One participant described social media as "really problematic . . . it sensationalized the issue." Another said, "Seeing all the posts was heartbreaking and I spiraled into a mental breakdown because of it." The constant barrage of online content could include processing about the reasons for the death, such as scapegoating or blaming other students. Friends of the deceased knew this information was inaccurate or distorted, and this made them angry and sad. Online expressions of grief from adolescents who were not close to the<br>deceased felt fake, superficial, and attention-seeking to friends... The public online conversations about the death were challenging for students who were trying to process the unexpected death of their classmate. " (p. 11 - Results)	Mirick, R. G., & Berkowitz, L. (2023). "Their Facebook posts were going viral": Social media in the aftermath of an adolescent suicide. *School Social Work Journal*, *48* (1), 1–18.
Mirick & Berkowitz	"One participant described how she felt after the death of her close friend: "When he died my entire world broke. I was diagnosed with complex grief. I didn't want to live, didn't want to interact with anyone. I wanted to go with him, kill myself too . . . to this day I still long for him." " (p. 236)<br><br>"Understand Students Have Different Postvention Needs. Participants identified the importance of understanding the diversity of student needs, acknowledging that some responses could be supportive for some students but triggering for others. For example, one participant said, "Social worker and counselor were hopping around each [seventh grade] classroom, and it triggered me [to think] about the student's death." Memorials were one example of supports where students had a variety of needs. One participant provided an example from her school, which hung a floor-to-ceiling commemorative mural in the school entrance: I'm sure it helped some kids, but it hit me like a ton of bricks when I was already having a hard time returning to school. . . . It would have been better if instead it was in a place where I could choose to go see it when I was ready. . . . Think about your front entrance as a place [where] some students might have a hard time getting back through . If you have memorials hung up, give students the choice to walk to a place that is not the entrance to see them. . . . The mural was an attempt for the community but a miss for me, specifically."" (p.238 - Results)<br><br>"Remember the Deceased. Participants wanted the school to remember the deceased student, especially on important dates (e.g., anniversary of the death, graduation), identifying this as a component of their grief process. One participant explained, "His memory should always have an impact on the school community forever." For a few, the lack of opportunities to remember, honor, and commemorate the deceased created distress, sadness, and anger. In describing her friend's death in eighth grade, one participant shared, "I wish they allowed us more space to mourn her. There was nothing done in her memory when we graduated and it's almost like something we don't acknowledge happened." Commemoration opportunities were valued and appreciated by participants." (p. 238 - Results)<br><br>"Flexible School Requirements. Finally, participants (n ¼ 10) wanted schools to be flexible with school requirements following a death, allowing for class cancellations, schedule changes, students to leave class, or turn in work late. For example, participants wished "they gave us a day to mourn his passing" and wanted "time to be with friends instead of schoolwork for at least a day" or "a period of time for mourning when students aren't responsible for homework or exams, even if it's just a few days." Students wanted the school routine to pause briefly, even just for a day or two, to acknowledge the death and allow students time to process and grieve . (p. 238 - Results)	Mirick, R. G., & Berkowitz, L. (2023). School-Based Postvention Services: Exploring the Perspectives of Students. *Children & Schools, 45* (4), 233–242. https://doi.org/10.1093/cs/cdad020
Morrissey et al.	"Provides a range of activities: Café evenings (open groups), where people meet, and share their experiences, peer-support via the telephone, walk and talk, access to books on relevant topics<br>seminars/workshops" (p.3)<br><br>"Coffee mornings, walk and talk, workshops, symposiums, access to a library on suicide bereavement for members, support via telephone, email and online platforms, outreach support to families, if needed, in their own homes and peer-led support group meetings." (p.3)<br><br>"While compassion and a will to understanding was important within the group, people were also motivated and oriented toward actions that nurtured and continued the bonds of friendship outside the group context. Participants were eager to help and support others when significant anniversaries/birthdays of the deceased occurred." (p.8)<br><br>"Just following up on that and what was said, even when it comes to maybe a birthday or anniversaries coming up and different events come up, messages go around, text message...we're thinking of you...it has become an extension of my family, we're there for each other. (Participant 10)." (p.8)<br><br>"Some of these actions were focused on providing emotional support at times when no one else was there, such as in the middle of the night or was focused on providing practical support by accompanying the person to coroner hearings. 'You can pick up the phone and talk to someone...last week (names person) was saying that she can be up at 4 o'clock in the morning, wondering who can I text and them someone says well I'm always awake at that time...you can text me.' (Participant 12)." (p.8)	Morrissey, J., Higgins, A., Buus, N., Berring, L. L., Connolly, T., & Hybholt, L. (2024). The gift of peer understanding and suicide bereavement support groups: A qualitative study. *Death Studies* , 1–12. https://doi.org/10.1080/07481187.2024.2378354
Murray-Swank	"This experience has also broadened my perspective on the importance of self-care and personal-professional integration in teaching, training, and supervision. As a result, I am making a more intentional effort to address these areas of competency and professional development. For example, I now routinely integrate these topics into teaching and attend to these areas of competency in my supervision practice." (p. 191)<br>"I try to more intentionally bring these elements into my work as a psychotherapist. As part of this process, I find that I have shifted in my focus as a psychotherapist to open up with more awareness and acceptance of my own internal expe- rience in the service of my clients and work in psychotherapy. For example, I find that I more frequently utilize experiential exercises or inter- ventions that facilitate accepting difficult experiences in the process of psychotherapy, such as mindfulness-based techniques that emphasize observing difficult experiences with compassion." (p. 193)	Murray-Swank, A. B. (2019). The Cracks Where the Light Gets In: Exploring Therapist Transformation Following the Loss of a Family Member to Suicide. *Journal of Psychotherapy Integration, 29* (2), 188–196. https://doi.org/10.1037/int0000147
Nolan	"The participant also reported poor coping behaviors in the form of social isolation, sleep depriv- ation, and excessive drinking. For example, the participant stated, "I go to work, come home, sleep" and "sometimes I'll completely and totally drink until I'm throwing up and passed out, so I don't feel anything."" (p. 524)<br>"The participant further explained, "I've been trying to go to church because that's what everybody does; and at one point in time, I liked going to church." In the same vein, however, the participant stated, "they still teach up here [rural area where bereaved resides] that being gay is wrong and stuff ... they [church con- gregation] know about me [gender identity/sexual orientation], but they don't accept it [gender identity/ sexual orientation]."" (p. 526)	Nolan, R. D. (2020). Transgender and gender non-conforming bereavement (TGNC): A case study on complicated grief experienced and the effect of partner suicide on interpersonal relationships and subsequent partnerships of the bereaved. *Death Studies, 44* (8), 521–530. https://doi.org/10.1080/07481187.2019.1586796
O'Connell et al.	"Some participants reported a negative impact on their relationships with others. This was primarily related to differing views on the death or blame regarding the death and often occurred in the context of family relationships. 'I think after suicide the whole family changes, so the dynamics shift, and nothing is the same again. It takes a while to build up, not even the trust but, to know who's right and who's wrong. Like your parents think one way or your mother might think one way. (Sinead);" (p.179)<br><br>"Participants spoke of how they had considered or tried other bereavement support groups (e.g., for spouses or parents) but mostly did not feel they belonged there given the unique experience of being bereaved by suicide and the emotions associated with it, including guilt and anger." (p.179)<br><br>"Participants valued that they could contact members of the group outside of the scheduled meeting times. Mostly participants recounted experiences of their shared group messaging platform. On this platform, someone could reach out if having a bad day, at the time of an anniversary, or people also shared quotes or ideas that they believe are helpful to others." (p.180)<br><br>"To a lesser extent, participants spoke of both benefits of sharing practical information within the groups and activities to commemorate the deceased. Participants valued opportunities to commemorate the deceased and discussions within the group that helped them to prepare for anniversaries or occasions. " (p.181)<br><br>"Most participants had experiences with other formal support such as counseling/therapy and many attended peer support during the same time periods as counseling" (p.182)	O'Connell, S., Troya, M. I., Arensman, E., & Griffin, E. (2023). "That feeling of solidarity and not being alone is incredibly, incredibly healing": A qualitative study of participating in suicide bereavement peer support groups. *Death Studies, 48* (2), 176–186. https://doi.org/10.1080/07481187.2023.2201922
Okami et al.	"She attended the funeral with family members. However, she did not have the opportunity to mourn. Her family hid her brother's suicide from those around them. The patient believed that expressing her grief would be a psychological burden on her family members, who were also grieving her brother's death privately. Therefore, she did not tell anyone and she mourned privately." (p.2)<br><br>"At 25 years old, she had been working as an engineer after one career change. She did not talk to people at work even when she had trouble with her job because she did not want to bother the people in her busy workplace with her talk. However, her coworkers told her that "You are difficult to talk to." She continually experienced challenges in socialization at work." (p.2)<br><br>"In addition, she still had difficulty expressing her negative feelings about her deceased brother to her family. Nevertheless, she had more emotional interactions with them and occasionally talked about the memories of her deceased brother. It was difficult for the patient to talk to people around her with a sense of security because she was afraid that she might bother them by talking them. However, after psychological education for PDD and communication practice, the patient gradually began to feel more secure in her interactions with others, and her emotional interactions with her family and friends increased ." (p.2)	Okami, T., Toshishige, Y., Kondo, M., Okazaki, J., Mizushima, H., & Akechi, T.. (2023). Interpersonal psychotherapy for comorbid prolonged grief disorder and persistent depressive disorder in a Japanese patient: A case report. *Psychiatry and Clinical Neurosciences Reports, 2* (4). https://doi.org/10.1002/pcn5.161

Oulanova et al.	"Becoming a peer counselor and supporting other survivors in this role represented a transformative process for the participants as they described altering their experiences with suicide loss through supporting others, as well as being changed on a personal level through volunteering. Two of the participants explained this in the following way:<br>Taking that negative, taking that dark moment of time, that nightmare, and how to turn it into a positive, through volunteering, to help somebody. By doing what I am doing with volunteer work, I am taking that situation and I am just trying to make some good come from that situation. (Lucy)<br>And the other thing that was really important to me, when I was doing this voluntary work. . . . I don't want this suicide to have been totally in vain. I want to have some meaning come out of it. (Ann)" (p. 156)<br>"Another challenge involved stigmatizing attitudes and family silence. As one participant shared:<br>I had to quit my full-time job 18 months after my son took his life because of the stigma in the workplace. (Patricia)" (p. 157)	Oulanova, O., Moodley, R., & Séguin, M. (2014). From Suicide Survivor to Peer Counselor: Breaking the Silence of Suicide Bereavement. *Omega: Journal of Death and Dying, 69(* 2), 151–168. https://doi.org/10.2190/OM.69.2.d
Peters et al.	"It's making that phone call and then, going to that meeting [suicide bereavement support group], I thought I'd be sick, I was that nervous. Then, realized the minute I walked in that room, I just knew everybody had an understanding. In their eyes, – you knew that they understood, which is why I still go to the meetings (Participant 7)" (p. 420)	Peters, K., Cunningham, C., Murphy, G., & Jackson, D. (2016). Helpful and unhelpful responses after suicide: Experiences of bereaved family members. *International Journal of Mental Health Nursing, 25(* 5), 418–425. https://doi.org/10.1111/inm.12224
Peterson et al.	"Specifically, we found the Low Symptom class had significantly lower severity than the Anxious class and Substance Use class across a range of mental health and substance use symptoms including but not limited to depression, anxiety, prescription drug use, and insomnia symptoms" (p.73)<br>"The Anxious class also had significantly elevated symptoms of anxiety and insomnia compared to the Substance Use class, which had significantly higher rates of head injuries in addition to higher rates of prescription drug abuse, illicit substance use, and alcohol use." (p.73)	Peterson, A., Chen, J., Bozzay, M., Bender, A., & Chu, C. (2024). Suicide risk profiles among service members and veterans exposed to suicide. *Journal of Clinical Psychology, 80* (1), 65–85. https://doi.org/10.1002/jclp.23592
Pettersen et al.	"I wanted to leave home. Everybody was so sad there, that it was impossible to talk [about the suicide], and I wanted to sit down and have a conversation with a psychiatrist . . . with someone who understands depression. It was important for me. It was my solace. It was what I needed. I changed jobs and I was forced to play happy. So, I went to a psychologist once a week and there I was able to just pour out all my sadness and then go back to work and just be myself. Without carrying all this with me." (p. 325)<br>"Three participants also described avoidant behaviors such as not being able to sleep in the sibling's bedroom or not being able to take a train after the suicide. They therefore recom- mended that professionals investigate such behaviors in bereaved siblings." (p. 328)	Pettersen, R., Omerov, P., Steineck, G., Dyregrov, A., Titelman, D., Dyregrov, K., & Nyberg, U. (2015). Suicide-Bereaved Siblings' Perception of Health Services. *Death Studies, 39* (6), 323–331. https://doi.org/10.1080/07481187.2014.946624
Pitman et al.	"School were brilliant—didn't hassle me for not going to lessons, extended all my deadlines, let me drop some lessons, but had meeting with tutor once a week to see how I was getting on, but (nicely) encouraged me to start doing work when the exams were coming up . . . really helpful." (21-year-old woman, bereaved four years previously by the suicide of her sister)" (p. 11)<br>"The best help that I could have had would have been help from family and friends to do my housework and look after my children. I felt so tired because my baby did not sleep at night and I was pregnant and breastfeeding." (30-year-old woman, bereaved six years previously by the suicide of her brother-in-law)" (p. 11)<br>"I stopped (counselling) around the time of my exams as it was better to ignore what had happened and concentrate on revising, than to have everything stirred up." (27-year-old woman, bereaved four years previously by the suicide of her mother)" (p. 14)	Pitman, A., De Souza, T., Khrisna Putri, A., Stevenson, F., King, M., Osborn, D., & Morant, N. (2018). Support Needs and Experiences of People Bereaved by Suicide: Qualitative Findings from a Cross-Sectional British Study of Bereaved Young Adults. *International Journal of Environmental Research and Public Health, 15* (4), 666-. https://doi.org/10.3390/ijerph15040666
Pitman et al.	"I've lost all focus and drive. I no longer want to be at university, I feel it's futile . . . .I've gone from being a high achiever, always busy, doing well to not doing anything. I have no focus, I can stare at the same page, not being able to read it and suddenly the entire day has gone by without me realising. Even when I manage to write something it's not very good because my brain's not firing in the way it used to and isn't making all the proper connections to write good work." (Male, 21 years old, in part-time paid work, bereaved less than a year previously by the suicide of his partner)" (p. 7 -Results)<br>"Motivation was such a problem for some people that their grades fell, or they dropped out of school, college, or university. This resulted in what were perceived to be significant departures from the trajectory they might otherwise have achieved. "A suicide changes how you see the world, especially if you are relatively young and not used to serious turmoil. This obviously has an effect on how positive a person can remain, or whether positivity is even a reasonable consideration. I still study, but my aims have changed. I only study in the hope that I will make money out of what I can learn. I have no real interest in what I am doing anymore, and I don't find inspiration in many things." (Male, 30 years old, no information on student/staff status, bereaved two years previously by the suicide of a close friend)" (p. 7 - Results)<br>"Managing emotions at work was an additional struggle for some respondents, and crying in front of colleagues or clients was described as particularly embarrassing. Others described having become more sensitive to criticism or negative feedback, resulting in thoughts and sometimes expressions of anger. These emotional outbursts could make working in a team very challenging. Managing a more general anger at the world was also linked to low motivation, and was cited as a reason for dropping out of jobs or courses. "I was at University at the time when it happened and I dropped out. I just became angry with the world and drank a lot in the evenings." (Female, 36 years old, no information on student/staff status, bereaved 12 years previously by the suicide of her father)" (p. 7 - Results)<br>"I rarely attended sixth form and often took time off uni when I was anxious/depressed although they rarely understood and I was punished for attendance several times" (Female, 21 years old, full-time student, bereaved four years previously by the suicide of her father)" (p. 7 - Results)<br>"I was in my last year at University when the bereavement occurred and became extremely stressed and depressed. It meant that trying to complete my dissertation project and prepare other coursework became extremely difficult. I was supported by my partner though and was able to complete my degree satisfactorily (at one point I had contemplated dropping out)." (Female, 26 years old, in full-time paid work, bereaved four years previously by the suicide of her uncle) " . . . my cousin's suicide was a factor in me developing anorexia. I believe it was partly, especially because I took on too many problems and weigh myself down. As a result of my illness I missed a year of school and didn't take any GCSEs. I also had to take a break from university." (Female, 22 years old, Full-time student, bereaved 10 years previously by the suicide of her cousin)" (p. 8 - Results)<br>"the combination of putting my feelings into a box for a year and starting uni did not combine well and caused me to break down a bit and the box opened with fairly disastrous consequences on my happiness for a period and this affected my concentration at uni. Many times I couldn't raise the energy to get up to go to lectures or when I was there I was so stressed and upset that I couldn't see the slides properly as I was so anxious." (Male, 21 years old, full-time student with part-time job, bereaved three years previously by the suicide of a close friend)" (p. 8 - Results)<br>"At the time my teaching suffered, I was less communicative with my colleagues and cared little about what I was doing." (Female, 27 years old, full-time student, bereaved three years previously by the suicide of her ex-partner) "The death has made me lack confidence more than I did, I do not believe in my capabilities in the work-place and often get stressed in tricky situations with customers (I work in retail). I also feel less able to relate to and interact with my work colleagues." (Female, 23 years old, full-time student with part-time job, bereaved two years previously by suicide of her father)" (p. 8 - Results)<br>"As a hairdresser I couldn't talk to people, so regulars wanted to ask me and I hated them for it, and when a client didn't know I was annoyed they seemed heartless. Had to quit working due to emotional distress, bad work performance, also no motivation." (Female, 33 years old, no information on student/staff status, bereaved nine years previously by the suicide of her partner)" (p. 9 - Results)	Pitman, A., Khrisna Putri, A., De Souza, T., Stevenson, F., King, M., Osborn, D., & Morant, N. (2018). The Impact of Suicide Bereavement on Educational and Occupational Functioning: A Qualitative Study of 460 Bereaved Adults. *International Journal of Environmental Research and Public Health, 15* (4), 643-. https://doi.org/10.3390/ijerph15040643
Pitman et al. Continued	"Losing this support meant losing someone who had normally encouraged the respondent to pursue their studies, or had looked after the home and family to facilitate work and student roles. For some it meant taking on a caring role within the family, with the associated costs of time, energy, and emotion. In a few cases it meant quitting jobs or courses to live nearer the family or to earn more to support them. These sacrifices, although felt to be the right decision, did significantly disrupt progress in work or education. "My school work suffered at the time, both because my home life was disrupted and also because my mother, who was suffering the most, was the person who would check up on me, my homework etc...She was totally absent during this time." (Female, 25 years old, full-time student with part-time job, bereaved 16 years previously by the suicide of an uncle)" (p. 10 - Results)<br>"For many of this group, work offered distraction from difficult emotions and traumatic memories. They described focussing energies on exam revision, a degree course, or projects at work, finding the structure and familiarity of work containing. Usually, it was reported that performance had improved due to the extra hours and attention devoted to work. "I think for me, because I liked to study and generally liked being at school, when my mother died this became a kind of support for me. I threw everything into my studies; it was what I knew best, it was a distraction too. This continued through college, university and is probably a reason why I am now a lecturer at a university—it's almost like the education system has offered structure, meaning and support to me; these were lacking at home after my mother died." (34 year old woman, in full-time paid work, bereaved 22 years previously by the suicide of her mother)" (p. 10 - Results)<br>"These respondents often described using the distraction of work as an escape from dealing with their grief, primarily by blocking out feelings. "I worked very hard on my finals to block out thoughts of her death. I felt a bit guilty for this at the time, but knew she wouldn't want us all to fail. I therefore put everything into my academic work as I have always done." (Female, 36 years old, full-time student, bereaved 15 years previously by the suicide of a close friend) "I went numb and focussed purely on my degree, and studies I think in a way to block out the traumatic events." (Female, 29 years old, full-time student with part-time job, bereaved seven years previously by the suicide of her sister)" (p. 11 - Results)<br>"The experience of a loved one's suicide often motivated an interest in careers related to mental health or the care of vulnerable individuals, sometimes prompting a complete career change." (p. 11 - Results)<br>"It changed how I view life.. . . . I left a well-paid career on a career break and did not return, choosing instead to work with vulnerable young people and adults for 2/3 of my previous salary." (Female, 36 years old, full-time student with part-time job, bereaved 10 years previously by the suicide of her partner)" (p. 12 - Results)<br>"I have always wanted to nurse, and after my husband died it spurred me on to do my degree and follow my dreams for me and for my family. . . . .it's changed me a great deal, maybe because I can empathise a lot more with relatives and patients in a very different way, I am a harder worker now than I ever was!" (32 year old female, full-time student, bereaved 11 years previously by the suicide of her partner)" (p. 12 - Results)<br>"This study adds the necessary context to our previous findings of a greater probability of drop-out from work or education in people bereaved by suicide. It is clear that specific aspects of grief, such as tearfulness, anger, reduced motivation, poor concentration, and anxiety, have a major impact on educational and work performance. Loss of motivation is likely to influence a decision to drop out of a course or job. Severe emotional distress, including the emergence or recurrence of mental health problems, risks interrupting or ending a planned trajectory, despite best ambitions. Having to move home and lacking appropriate support are also factors likely to explain higher drop-out rates." (p. 12 - Discussion)	Pitman, A., Khrisna Putri, A., De Souza, T., Stevenson, F., King, M., Osborn, D., & Morant, N. (2018). The Impact of Suicide Bereavement on Educational and Occupational Functioning: A Qualitative Study of 460 Bereaved Adults. *International Journal of Environmental Research and Public Health, 15* (4), 643-. https://doi.org/10.3390/ijerph15040643

Powell & Matthys	"Several years after he died I started working in the suicide prevention and after care field and started working for a crisis line and support groups for others who had lost someone to suicide. And I try to explain to people about mental illness and seeking help, so that's the only positive thing I can think that came out of his death is the help I have provided to other people based on my experience. (36:4)" (p. 333 - Results)<br><br>"All of the survivors had reached out to other survivors of suicide via the Internet or through face-to-face support groups. Connecting with other suicide survivors via online support groups "gave me a purpose," said one participant who lost her brother five years ago (11:3). "I knew that I couldn't just go on being a sister who lost her brother and best friend to suicide. I had to do something positive about it or it was going to take the best of me away. Once I found out about the overnight walk I really wanted to do that for my brother and do something positive," she said (11:3)" (p. 333 - Results)	Powell, K. A., & Matthys, A. (2013). Effects of Suicide on Siblings: Uncertainty and the Grief Process. *Journal of Family Communication, 13* (4), 321–339. https://doi.org/10.1080/15267431.2013.823431
Praetorius & Rivedal	"Here is Barb's description of the aftermath of her brother's death: So my grief journey began, one for which I was not prepared. I searched for answers to the ''whys'' by going to conferences, researching suicide, and educating myself as much as I could. During the 1980s there was very little written about suicide, and not many people were talking about it . . . The day John ended his pain, our pain began. I was determined to not let this destroy me as it did my father. Tragedies can make you bitter or better—it's a choice. I was going to be a better friend, mentor, and person in every way. When someone suicides, it changes you, but it doesn't have to destroy you. (Rivedal, 2016, p. 142)" (p. 354 - Results)<br><br>"Ashley's description illustrates how escaping to shore isn't always easy: Shortly before his twentieth birthday and after six months of being completely clean, Dane lost his struggle with heroin. I soon felt my depression blanket encase me so tightly that I felt that I couldn't breathe. My best friend was gone. Sleep was foreign to me, but I couldn't make myself get out of bed. (Rivedal, 2016, p. 146)" (p. 354 - Results)<br><br>"As Ashley shared, ''while I managed to finish my freshman year of college, I was completely lost. Why couldn't he [Dane] have kept a shred of the love he held for other people, for himself?'' (Rivedal, 2016, pp. 146–147). Marcia similarly describes her ocean after Doug's death: How does a parent feel when they lose a child to suicide? I felt like my world had come to an end. I was just a normal, regular person. I had buried one child, and one child was severely brain damaged. Why me? I was on a planet all by myself. How could this happen to me? What would I do now? I was totally clueless as to what to do to continue on with my life without Doug. (Rivedal, 2016, p. 158)" (p. 355, Results)<br><br>"Marcia describes how the help of friends and solidarity with other survivors helped her right her ship: My friends helped me find support groups with other normal parents like me who had also lost their children to suicide. They were on this strange new planet with me. I went to every in-person support group available. I joined an online support group ... We grieved together, we supported each other, and we understood each other. We were in a club that no one wanted to be in. (Rivedal, 2016, p. 158)" (p. 355, Results)<br><br>"And Jennifer's story depicts that one is not always equipped at first with the necessary skills for righting the ship. Navigating out of the ocean of why did not immediately lead her to therapy; there was a detour of unhealthy coping: There were many times when, after tucking the kids in bed, I would retreat to my computer, put on my headphones, pour one, two . . . four glasses of wine, and just lose myself in music . . . Music was the very beginning of a means to process what my soul was begging me to deal with . . . My survivor's guilt had turned into self- loathing and binge drinking. My soul was crying out for help, begging and finally demanding for me to deal with my own pain. Music was the medium my soul used to wake me. (Rivedal, 2016, pp. 137–138)" (p. 356 - Results)<br><br>"Barb spearheaded services for those like her: I started a support group, Survivors of Suicide, for loved ones left behind. I learned so much from the participants who were willing to share their stories—but still this wasn't enough. I thought, We can sit around this table talking about the 'whys' and our grief for the next twenty-five years, or we can take our grief and educate others. (Rivedal, 2016, p. 142–143)" (p. 356 - Results)<br><br>"Ultimately, with each of the storytellers, this included work toward helping others, usually those bereaved by suicide. Judy became advocate for those who have lost their ability to advocate for themselves; Barb started a support group for SOS Loss; Marcia stated that giving back and helping others is the only way to give meaning to Doug's death; and Ashley declared that as a professional teacher she will incorporate a true sense of community and self-love into her classroom." (p. 358 - Discussion)	Praetorius, R. T., & Rivedal, J. (2020). Navigating Out of the Ocean of "Why"—A Qualitative Study of the Trajectory of Suicide Bereavement. *Illness, Crisis, and Loss, 28* (4), 347–362. https://doi.org/10.1177/1054137317741714
Quayle et al.	""I think I probably have more negative images than positive images. . . .I suppose one thing that I tried to kind of do . . . I would look through photos that we had from holidays, . . . his Facebook profile and all the images and posts on there. . . .I kind of force myself to review those to try and remember the happy times . . . I'd say generally the images that come to mind are associated with his death rather than his life."" (p.8)<br><br>"Eleven participants spoke about the negative impacts that intrusive imagery had had on their everyday lives, with the distress causing them to feel agitated, distracted, or unable to sleep,<br>and hampering functioning. "I had them for months and months and months, until I was back at work, I realised that these imaginings were destroying me. I was getting quite careless at work."" (p.8)<br><br>""That image can be so powerful, and the immediate kind of emotional response means that somehow or another you're, you know, quasi-paralysed. . . . It's so traumatic as to sort of freeze you on the spot. . . . I have been known to stand still in the street for no particular reason.""	Quayle, K., Jones, P., Di Simplicio, M., Kamboj, S., & Pitman, A.. (2023). Exploring the phenomenon of intrusive mental imagery after suicide bereavement: A qualitative interview study in a British sample. *PLOS One, 18* (8), e0284897. https://doi.org/10.1371/journal.pone.0284897
Ratnarajah et al.	"After a suicide death has occurred in a family, a task for all family members is to renegotiate their relationships with the remaining family members. All have been affected in various ways by the tragedy and the actual way the family system works may have to be realigned as the members adjust to the gap in the family. Those families that have an open communication style and are able to discuss the tragedy and their reactions to the loss will process this transition more healthily than those with a difficult or conflicted family relationship. This adjustment process is one of the tasks that may be facilitated by either a counselor or other professional support. It can also be one of the reasons bereaved family members seek out suicide-bereavement support groups. Two of the narratives illustrate various ways the participants sought help to rebuild their family. Delma and her son-in-law Philip both spoke of the help that was provided to the family after Colin's death by the police support officer and then, later, Delma joined a bereavement support group and for many years led a group herself. Similarly, another participant, Emma, sought Internet bereavement support when no support group was available in her capital city and then started and led a support group. She was also most concerned for her three sons, all of whom withdrew from her and each other following their father's suicide death." (p. 47 - Results)<br><br>"Information relating to the spiritual beliefs of participants or their deceased family members came spontaneously as they narrated their experiences. While such information was not specifically asked, of the 18 participants, 8 spoke about either their family's traditional belief-system allegiance or their own connection to either a church or spiritual practice. Events in the lives of some participants caused them to have critical thoughts about the church to which the family belonged or where they had previously worshiped" (p. 52 - Results)	Ratnarajah, D., Maple, M., & Minichiello, V. (2014). Understanding Family Member Suicide Narratives by Investigating Family History. *Omega: Journal of Death and Dying, 69* (1), 41–57. https://doi.org/10.2190/OM.69.1.c
Rivart et al.	"Another maladaptive coping strategy adopted by suicide loss survivors was increased alcohol use. One participant explained engaging in "excessive drinking and drugs" (participant 134) and another reported that their loss had "increased my alcoholism" (participant 225). Whilst this was not as prevalent as experiences of suicide ideation or self-harm, it was one of the most reported high-risk behaviors." (p. 5 - Results)<br><br>"In some cases, this intense anger resulted in other maladaptive coping behaviors: one participant reported that their "upset and anger about the suicide as well as other problems led to dangerous driving" (participant 226). Overall, these maladaptive coping strategies seemed to be an initial response to the experienced loss, similar to acute grief." (p. 5 - Results)<br><br>"Participants reported a range of maladaptive coping strategies including harmful behaviors, increased alcohol use, and anger issues as a result of suicide bereavement. Participants also reported that their understanding of the suicide had been largely influenced by emotional processes following the latter which, for most participants, was guided by feelings of guilt and abandonment, leading to further distress." (p. 8 - Discussion)	Rivart, P., Wainwright, V., Flynn, S., Hunt, I. M., Shaw, J., Smith, S., McGale, B., & McDonnell, S. (2021). Ethnic Minority Groups' Experiences of Suicide Bereavement: A Qualitative Exploratory Study. *International Journal of Environmental Research and Public Health, 18* (22), 11860-. https://doi.org/10.3390/ijerph182211860
Rogerson & Carson	A year after his death I moved to Brighton, I wanted to get away from where everyone knew me, and knew what had happened. I got a job, bought a flat and tried to move on with my life. But I had not dealt with his death, and struggled with bouts of depression. If anyone asked how my dad had died, I had to lie because I did not want anyone who had not known him, to judge him for what he had done. I had heard others say before that people who take their own lives are selfish, and that used to make me so angry. I never felt angry with him (even though one counsellor said I would!), I always understood how much he must have been suffering, and was just so sad that he had not been able to share it with me and get the help that he needed. Having personal experience of depression does help with that understanding, and after my last bout I found a really good therapist and had a course of Cognitive Behavioural Therapy. Although he touched on my dad's death, he mainly worked on my fractured relationship with my mother, which I then viewed was one of the main reasons behind my depression. But I was failing to recognise that I was still waking up with nightmares, and that hearing sirens or seeing police cars would bring back images of that day. I also constantly felt on edge, avoided going to certain places and disconnected myself from people, which I thought was either part of the depression, or just part of my personality. (p. 209)	Rogerson, S., & Carson, J. (2017). Remarkable lives: Suzanne Rogerson in conversation with Jerome Carson. *Mental Health and Social Inclusion, 21* (4), 208–212. https://doi.org/10.1108/MHSI-06-2017-0026
Roitman	Mark, a twelve-year-old boy, was referred to psychotherapy by social services. He was a bereaved brother; his elder sibling, a drug addict, had committed suicide, and Mark was the first person to find the young man's dead body. Shortly after, Mark became obsessed with thoughts and images of children committing suicide or dying, and of their dead bodies. He began compulsively drawing pictures relating to these themes. His functioning at school declined drastically; he isolated himself, became electively mute, and gave up all his friendships. (p. 406 - The brother in Bardo)<br><br>At first, Mark compulsively drew pictures of the horrors he was obsessively preoccupied with, concerning a murdered child or a child killing himself. He would draw each picture on a separate page: a child being stabbed, jumping to death, burning to death, not wanting to live, thinking he doesn't deserve to live, etc. After finishing a picture, he would absentmindedly but violently throw the page onto the floor. (p. 407 - The usual suspects)<br><br>Positive changes began to occur; Mark's emotional state significantly improved; he began to study better and made new friends. Our playing became calm and quiet,<br>involving mostly board games. Unfortunately, the therapy ended earlier than expected when social services sent Mark to a boarding school in another city, judging it to be better for his development. (p. 410 - The way to reduction)	Roitman, Y. (2021). A play for a bereaved brother: mutually playing with the child survivor of a sibling suicide. *Journal of Child Psychotherapy, 47* (3), 402–414. https://doi.org/10.1080/0075417X.2021.2015797
Ross et al.	Some participants described feeling "too raw" to seek emotional assistance early in their grief, but once their feelings of numbness and confusion had subsided, they benefited from support such as counseling and support groups. (p. 5 - Results)<br><br>The first time I went I told (the counsellor) my situation, I walked out going "Oh, he's no help. He doesn't understand." But 6 months later I was talking to somebody and they said "You really do need help. Could you please just go and see somebody?" When I went and saw the psychologist the next time and I did all the sessions. He helped greatly . . . I'm more aware now of what happened, why it happened, why I couldn't help it and why I couldn't save this man. (Female) (p. 6 - Results)<br><br>The Internet as both useful and distressing. Some participants described how they found Internet-based groups such as Facebook groups and some online forums to be a useful way to connect with other suicide-bereaved persons. While a number of participants described the useful role of online forums in connecting and identifying with others, some found these to be less helpful after some time.<br>Others highlighted the negative aspects of online forums on suicide loss, saying that these tended to be too intense and they found it distressing to read other stories of loss. Facebook groups and online forums and things (were helpful) . . . because initially hearing people's stories and identifying similarities and—I found that really comforting. But then after a while, you hear the stories of the loss and everything and it was just more upsetting . . . (Female) (p. 8 - Results)<br><br>Participants also discussed their experiences in connecting with others bereaved by suicide through face-toface suicide-bereavement support groups. Although there were mixed opinions on the value of support groups, most participants spoke of the positive experience of connecting with others; in particular, the value of talking with others who have lived through similar experiences. These shared experiences were not only in relation to their grief and loss but also the strain of the caregiver burden (i.e., being on "suicide watch" in the lead-up to the suicide, as described by a<br>number of participants). (p. 9 - Results)	Ross, V., Kõlves, K., & De Leo, D. (2021). Exploring the Support Needs of People Bereaved by Suicide: A Qualitative Study. Omega: *Journal of Death and Dying, 82* (4), 632–645. https://doi.org/10.1177/0030222819825775

Ross et al.	Another example of avoidance was shown in fathers who reported working excessively in order to avoid the pain of thinking about their loss. Several parents also mentioned that they were drinking excessively, and for some this problem appeared to be increasing. Others spoke of their difficulties sleeping and their subsequent use of alcohol and/or marijuana in order to help them sleep at night. One mother described how she was only just coping, and described her pain as something that she simply had to endure. (p. 4 - Results)<br><br>“It’s the weekly, every day drinking in the week that’s definitely increased. Whereas before, we’d try not drink for three days . . . but now it’s definitely, at least one bottle to myself, every night.” (Father: 6 months). (p. 4 - Results)<br><br>Several fathers told of how they found it helpful to keep a journal where they wrote letters to their child. Some parents described the importance of celebrating their child’s birthdays (which was generally thought to be far more positive and preferable to marking the day of their loss). Others maintained a connection with their child through visits to their loved one’s gravesite or resting place. Some parents (both male and female) described how their faith/religion and attending church had helped them cope. Keeping occupied and maintaining a routine through work and other interests were also cited as coping strategies. (p. 4 - Results)<br><br>“If I was to say there were two things that have helped a lot in me just processing what’s going on in life and where I’m at and reflect on myself—going for walks and thinking and<br>a little bit of talking out loud . . . But also, writing a kind of journal, which is typically just like a brain dump of where I’m at, what I’m thinking. On occasions, I’ve done a letter to Edward and they’ve been good ways for me to step back and evaluate where I’m at.” (Father: 12 months). (p. 5 - Results)<br><br>“I go (to the cemetery) every week. I pick up the flowers on a Friday because I’ve got a standing order at the florist.” (Mother: 12 months). (p. 5 - Results)<br><br>Some parents (both mothers and fathers) described how attending individual counselling and suicide bereavement support groups had been critical to their ongoing coping and recovery. When discussing support groups, participants spoke of their sense of relief at being able to talk to others who understood and had suffered the same loss. In addition, one mother recounted how her workplace had been greatly supportive of her loss (e.g., helping her to access counselling, and showing empathy and consideration regarding her need to take time off), which she felt helped with her ability to cope. (p. 5. - Results)<br><br>Others spoke of how they had learnt to change their priorities, placing more value on life and not taking everyday things for granted. Participants described a wide variety of ways in which they were making their lives meaningful again to enable them to move forward. Making a positive contribution through work, helping others through charity work and fundraising, connecting with nature through walks and camping trips, and simply being open to enjoying experiences and friendships were all cited as ways in which parents were beginning to move forward with life. However, it should be noted that not all parents reported such positive responses, with some still struggling with their grief and unable to move forward at the 12 month time period. (p. 6 - Results)<br><br>“I tend to think more about being deliberate in how I use my time to enjoy it and to take in new experiences, or revisit experiences I’ve enjoyed that I might have let fall by the wayside over the years. So a drive to the beach, a walk on the beach, trip in the country, just being on the open road, all those are things that I haven’t done much in recent years.” (Father: 12 months). (p. 6 - Results)	Ross, V., Kõlves, K., Kunde, L., & De Leo, D. (2018). Parents’ Experiences of Suicide-Bereavement: A Qualitative Study at 6 and 12 Months after Loss. *International Journal of Environmental Research and Public Health, 15* (4), 618-. https://doi.org/10.3390/ijerph15040618
Salom et al.	I started taking courses, keeping my mind occupied; needed to be busy. I left my job, left everything to take care of myself. I began practicing meditation and going for long runs to let out my anger. Now ’m giving lectures and talks, something that would have been unthinkable for me a few years ago. wouldn’t have even imagined it. We play his favorite music to remember him. We planted a tree in his memory. I have a picture of her, and say good morning every day. We talk about them a lot, sharing photos—some that others don’t have—and even videos. Going to the cemetery brings a sense of closeness. (p. 92-93)	Salom, R., Layrón, J. E., Neimeyer, R. A., & Pérez, S. (2024). Construction of meaning in survivors of suicide loss: A Spanish translation and application of the Meaning in Loss Codebook. *Death Studies, 49* (1), 87–99. https://doi.org/10.1080/07481187.2024.2438412
Sanford et al.	"After several sentences describing the extent of his exposure, he said it “has an ongoing and lasting effect to cops as well, and has contributed a great deal to why I am no longer a [Location] police officer and have been diagnosed with PTSD with all of the usual symptoms and signs.”" (p.9)<br><br>"A train driver described the significant impact of her exposure: “As a train driver, it has effected [sic] me at work and in many life situations. The impact has created a reaction to certain physical movements that people make when I am driving a car, a train, a truck or even riding a bike. It has made me super sensitive to mitigating danger to individuals. It has effected [sic] me emotionally but it’s hard to put in words” (p.11)<br><br>"Repeat exposure to suicide deaths for one frontline responder caused PTSD severe enough to cause disruption to his work. He shared that he “attended numerous suicides in my role over 21 years” and “was medically discharged from a job I loved due to PTSD Post Traumatic Stress Disorder directly related to my work as a firefighter”" (p.12)<br><br>"One participant shared, “Given my family’s history of suicide in the young Aboriginal male per generation, I worry for my own teenage child. He has had adverse childhood experiences, I have made every effort to keep him engaged and connected socially . . .I worry that I haven’t been able to support him developing sufficient resilience to persist in this world and he might be next. It weighs on my mind constantly” (p.13)<br><br>"Another respondent was impacted by her concern for the well-being of their granddaughter following the death: “The effect on me and my life has been through the devastating and probably lifelong effect it has had on my granddaughter, who is now suffering from PTSD and is having treatment at this time. It has effected [sic] us as a whole family and remains to do so” (p.14)<br><br>"A family exposure to suicide and the questions surrounding the experience pushed one participant to pursue a career in suicidology: “I was totally confused as to why my Uncle died by his own hand . . . because of my experience I went on to obtain an extra qualification [in suicidology]. I have spent 12 years as a telephone counsellor with one organization and spent 4 years with another organization as a Trauma Response worker. All of this generated out of my experience when I was younger”" (p.15)<br><br>"Following her father’s death, another participant’s family was fearful of suicide transmission, resulting in vigilance over a family member, which is similar to the quote in the past theme. Here however, communication and strengthening of familial bonds contributed to assuaging these fears, and efforts to disrupt family patterns and fear of transmission created motivation to work in suicide prevention: “I found out my father’s death was in my late 20s and at that time I was unaware that he died of suicide. It was a time that I had thoughts of suicide myself. I have a twin sister who has been unwell (depression) over the years and has been suicidal and all of this makes us as family extra vigilant. However, I believe that over the years my sisters and I have spoken about many things and this has helped us enormously. I now work in the area and am eager for the myths about suicide and for the conversations to continue”" (p.15)	Sanford, R. L., Frey, L. M., Thind, N., Butcher, B., & Maple, M. (2023). Unpacking the meaning of closeness, reconsidering the concept of impact in suicide exposure, and expanding beyond bereavement: “Just, I hope you don’t forget about us.” *OMEGA: Journal of Death and Dying, 0* (0), 1–29. https://doi.org/10.1177/00302228231196616
Sanford et al.	An important finding from this study was that participants who entered therapy less than 3 months following the death reported greater benefit of therapy than those who initiated therapy later. While it may be that active treatment seekers had better outcomes, it also suggests that intervention as soon as possible following the death may be most beneficial for loss survivors. (p. 556 - Discussion)	Sanford, R., Cerel, J., McGann, V., & Maple, M. (2016). Suicide Loss Survivors' Experiences with Therapy: Implications for Clinical Practice. *Community Mental Health Journal, 52* (5), 551–558. https://doi.org/10.1007/s10597-016-0006-6
Schotanus-Dijkstra et al.	Poetry was the only direct expression of creativity in the messages. Participants also mentioned that drawing or painting, listening to certain music, writing a book, or writing letters to the deceased helps them in their grieving process. “It helps me to listen to good music and just be sad, but also to write letters to my husband. I write my anger, worries, love, and sorrow to him.” Creative expression was mentioned in 5% of the messages. (p. 32 - Results)<br><br>The messages show that friendships can develop. Participants engaged in conversations for many weeks and asked each other how they were doing. (p. 32 - Results)<br><br>Participants expressed greater satisfaction with (online) support groups, specialized psychologists (e.g., victim care or a bereavement counselor), and spiritual intermediaries than with general psychologists and general practitioners. (p. 33 - Results)	Schotanus-Dijkstra, M., Havinga, P., van Ballegooijen, W., Delfosse, L., Mokkenstorm, J., & Boon, B. (2014). What do the bereaved by suicide communicate in online support groups? A content analysis. *Crisis: The Journal of Crisis Intervention & Suicide Prevention, 35* (1), 27–35. https://doi.org/10.1027/0227-5910/a000225
Shields et al.	Mothers who have lost a child to suicide continue on in the role of mother, even though their child had died. This process involved protecting their children from any blame associated with the act of suicide by minimizing the role of the son in the suicide. This was accomplished by directing the blame inward onto themselves or directing it outward onto services, alcohol, medication, or other people. (p. 182 - Results)<br><br>The search for answers was paralleled by a search for a deeper level of understanding concerning the loss of their child. There was a sense that developing an understanding of the suicide and finding a meaning within it would aid participants in the healing process. For example, Marie described her ways of trying to make sense of the suicide: I’ve been reading about it and talking to other people. Like I read a lot of books about it (pause) you know after it happened. To try and understand why.” (p. 185-186 - Results)<br><br>Sue illustrated how the search for answers and understanding led some participants to seek the services of mediums in an attempt to answer some of their questions: ‘‘I run to them mediums for years trying to get answer and spent a fortune on phone calls trying to get them, trying to get answers. But no answers. (p. 186 - Results)<br><br>Participants struggled to find a place in which their emotions could be expressed in a safe, nonjudgmental environment in which they could be themselves without worrying about the impact on others. The support group which the participants attended seemed to fill this void. (p. 187 - Results)<br><br>Attending a support group with others who had been bereaved by suicide and in which others understood their pain and had gone through similar experiences provided participants with a place where they felt that they belonged: It’s like your own wee world sort of thing, you know (pause) and I suppose you feel safe in it and you just feel comfortable that you can share and talk it over. (Sue) (p. 190 - Results)	Shields, C., Russo, K., & Kavanagh, M. (2019). Angels of Courage: The Experiences of Mothers Who Have Been Bereaved by Suicide. *Omega: Journal of Death & Dying, 80* (2), 175–201. https://doi.org/10.1177/0030222817725180

Silvén Hagström	Their primary advice to themselves from the program was to engage in different activities such as: "go outdoors and ride a bike," "bake cookies," "build with Lego" or "play with a friend," aimed at distraction to avoid thinking about the deceased parent. These and similar distraction strategies were being used. Ivar is a case in point: "I don't know, I try not to think about it (laughs). I do something else like scroll on YouTube or something. Focusing on something else is good." Selma, 9 years old, explained how she tried to activate herself to counteract painful thoughts, but on other occasions allowed herself to be sad. Sometimes I just walk around the apartment and: "okay, what can I do?" Then I start watering the flowers or something. . . and I make drawings and put glitter on and stuff. . . I want to be alone. Or I go to bed and cry a little bit. . . then I fix with my mobile phone. (p. 9 - Children's Strengthened Agency and Management of Grief)<br><br>Finally, Vanja described her coping strategy in grief. "When I'm sad I listen to Sofia and Alio." She had earlier explained that it had been helpful to listen to the other children's narrated experiences at the grief support camp and she had continued to listen to others' grief experiences in the form of song texts. In the interview, she played specific songs that she found had a healing effect on her. She reflected: "When I listen so Sofia, I feel like it's me who's singing. It's a beautiful song (she exhales). If something is worse though, like with her, you can really feel 'what a good life I have'." (p. 9 - Children's Strengthened Agency and Management of Grief)<br><br>Overall, participation in the grief support program was said to have contributed to a process of destigmatization, which made both parents and children feel more comfortable about talking about their parental loss in their social networks and less sensitive about the responses of others. (p. 10 - Open Communication Within the Family and Social Network)	Silvén Hagström, A. (2021). A Narrative Evaluation of a Grief Support Camp for Families Affected by a Parent's Suicide. *Frontiers in Psychiatry, 12,* 783066–783066. https://doi.org/10.3389/fpsyt.2021.783066
Silvén Hagström	Emelie described how she used to visit her father's grave to let him know about her anger with him. She yelled at him in heaven and smoked and stubbed out her cigarettes on his gravestone. Eventually, she felt that "he has got what he deserved" and she reconciled with him. She could feel his presence during her singing performance at her graduation and she knew that he was proud and happy for her. (p. 118 - A continuing bond with the deceased parent)	Silvén Hagström, A. (2019). "Why did he choose to die?": A meaning-searching approach to parental suicide bereavement in youth. *Death Studies , 43* (2), 113–121. https://doi.org/10.1080/07481187.2018.1457604
Silvén Hagström	In many cases, the children are preoccupied with the matter of forgiveness and reconciliation, that is, the child's capacity or readiness to forgive the parent for the suicide. This daughter expresses the ambivalence caused by this interpretation: Dad!!! Now you have been away from me for 4 years. Even now it feels unreal, but still not .... Sometimes I am so angry with you. I just want to yell at you . Sometimes I want to tell you how much I love you . But the fact that you betrayed me so much! I will probably never get over this. Maybe I am selfish, but I will probably never forgive you for this! I miss you! This account and others like it show that this Internet arena for social support offers the opportunity to communicate not only with other bereaved, but also with the deceased parent in what has been described as a 'continued bond', which can work as a resource in the children's meaning construction (Klass et al., 1996; Neimeyer, 2014; Silve´n Hagstro¨m, 2014)	Silvén Hagström, A. (2017). 'Suicide stigma' renegotiated: Storytelling, social support and resistance in an Internet-based community for the young suicide-bereaved. *Qualitative Social Work : QSW : Research and Practice, 16* (6), 775–792. https://doi.org/10.1177/1473325016644039
Spillane et al.	Some participants attributed their low energy levels to 'the emotion' and 'turmoil' associated with their grieving, while others felt it was due to their disrupted sleeping patterns. Reported problems with sleeping in the immediate aftermath varied in severity and duration. One participant described how they 'couldn't sleep at all in the beginning' and another described how they tried to tire themselves during the day with walks in an attempt to sleep at night. (p. 5 - Results)<br><br>Loss of appetite was reported by some participants as a psychosomatic reaction which often led to weight loss. Reasons for loss of appetite varied, including nausea due to flashbacks of finding the body or feelings of depression and despondence following the death: 'Food-wise, I'm never hungry, I could stay without it all day...if I have a cup of tea and a bit of bread in the morning, I'm grand...Since himself has gone, you're just getting up in the morning doing the odd old thing, sure what's the point in doing it like' (spouse) (p. 5 - Results)<br><br>Their health status was often influenced by their health behaviours. Some family members noted 'everything stopped, the world stopped that day' and tried but failed to resume their normal physical activity. For others, negative health behaviours including excessive alcohol consumption and overeating were used as a coping mechanism: 'I'd drink I'd say [pauses] a bottle of vodka a day and a few pints as well... it's [the alcohol consumption] got a bit worse... I don't know if it's directly related to it or whether I'm using it as an excuse' (parent) (p. 5 - Results)<br><br>Importantly, some family members experienced an improvement in health behaviours, including increasing their levels of physical activity which benefited fitness levels, healthy weight loss and aided the grieving process: 'I went out to the dancing on a Wednesday night, I said make new friends you know...ya I've got fitter... That was a big boost for me to chat to people and pass away the week' (spouse) (p. 5-6 - Results)<br><br>Another participant sought formal support as they 'needed to speak to somebody outside of my family because I was upsetting everybody when I wanted to talk'. Seeking formal support was imperative 'to get the counselling, just taking time to reflect on everything and deal with it'. (p. 5-6 - Results)<br><br>Some participants spoke about seeking new relationships following their partner's death. One participant spoke about how her friends and her counsellor broached the topic of a new relationship with her and she felt 'why not...I have an awful lot of love to give'. Seeking new relationships and friendships was an important aspect of moving forward for some participants as 'there was lots of times where I wouldn't go out...but eventually I got it into my head, I went out to the dancing on a Wednesday night, I said make new friends...and then I met this new girl last year before Christmas' (p. 7 - Results)<br><br>One participant noted that simple things like turning on the radio so there's 'something on in the house' or watching a DVD with his children helps as he 'enjoys it when we're all together'. Various other social activities and past-times such as walking and gardening were endorsed by some as helping during the grieving process. One participant spoke about how she uses yoga as a means of 'being present' and to tell herself that she's 'ok' even when 'there are still images in my head' after finding the deceased. A further participant stated they were 'very positive' and engaged in walking and 'a bit of photography' which helped him in 'hanging together fairly well'. (p. 7 - Results)<br><br>Part of this reconstruction was also about reappraising what was important to them and how they thought about life. Some participants chose to make big life changes after the death, including moving homes, changing jobs or completely disengaging from the work environment: 'I haven't gone back to my old job in [big city], you know life has changed and I was working long days and didn't really have a life, now, I'm looking back and saying, there's a little bit more to life than that you know?' (spouse) (p. 7 - Results)	Spillane A., Matvienko-Sikar K., Larkin C., Corcoran P., & Arensman E. (2018). What are the physical and psychological health effects of suicide bereavement on family members? An observational and interview mixed-methods study in Ireland. *BMJ Open, 8* (1), e019472. https://doi.org/10.1136/bmjopen-2017-019472
Stewart & Thomas	It wasn't until I began psychotherapy training that I realised that: I have a heightened attunement to any threat of abandonment'. [Every college break] felt like the world was ending. It felt unfamiliar. It impacted me as I began seeing clients also. I noticed a familiar threat of abandonment triggered by my first client's desire to end the work: I ... shifted down into my abandoned child ... defensively merged ... to maintain contact with her (p. 557- Findings)	Stewart, K., & Thomas, V. (2020). A trainee psychotherapist's heuristic exploration of losing a mother to suicide. *British Journal of Guidance & Counselling, 48* (4), 552–562. https://doi.org/10.1080/03069885.2018.1519181
Stirling	For the purpose of this study, I began to engage with regular yoga practice for the first time in my life. This continued for 4 months, and I built a collection of experiential notes throughout. I took part in various style of yoga, across several locations, to gather an eclectic array of practice. I wrote about each session as fully as I could. Even at times of injury, wrote about the impact of not doing yoga. I also wrote about life outside of yoga, in order to make connections with how the practice was affecting me in real ways, and to establish macro- and microlinkages (Laslett, 1999). (p. 281 - Methodology, Ethics, and a Difficult Brunch)<br><br>The yoga itself was slow and easy, I found paths around by bodily aches and found a gentle focus. After each pose, we dedicated a few minutes to noting changes in our body. By the time mindful meditation came around again, I was able to engage in a completely different way. (p. 282 - Inside Out and Upside Down: Engaging in Yoga)	Stirling, F. J. (2016). Yoga and Loss: An Autoethnographical Exploration of Grief, Mind, and Body. *Illness, Crisis, and Loss , 24* (4), 279–291. https://doi.org/10.1177/1054137316659396
Strouse et al.	The AGS is designed to meet not only group but also individual survivors' needs in a meaningful way. For example, one survivor who had witnessed her husband's suicide used collage shreds to process her husband's suicide. One couple honored their son, an Olympic bound weightlifter, in the pages of a small watercolor book, embossed with affirmations of his military life and interspersed with black barbells. (p. 2 - Artful grief studio)<br><br>Putting together and taking apart bits and pieces of magazine images (Strouse, 2020), and found objects (Bat-Or & Megides, 2016; Siano, 2016), enables the deconstruction and reconstruction processes (Neimeyer, 2019) required for grief restoration work (Stroebe & Schut, 1999). For example, a father who had lost his son and created a collage said: "I found refuge in the calm quiet space of the Artful Grief studio. I had not been in an environment where it was ok to express myself emotively, from my heart not my mind. I was encouraged by strangers and peers. I was given tools to reclaim myself, to begin again, anew a deeper reflection on who I am and a more profound understanding of my love for my son." (Fig. 2). (p. 3 - Collage and assemblage)<br><br>No negative comments about unhelpful aspects of the program were reported. Participants stated that all aspects of the studio were helpful. The essential contributions of the art were identified as: the importance of a variety of materials (i.e. dolls, 3D materials for collage like books and boxes), activities (i.e. collage, mobiles, healing sticks, doll-making), environment, and overall experience. Codes that suggested relaxing experiences included: a sense of calm, warmth and positive emotion, and a present moment awareness (Table 2). (p. 7 - Results)	Strouse, S., Hass-Cohen, N., & Bokoch, R. (2021). Benefits of an open art studio to military suicide survivors. *The Arts in Psychotherapy, 72* , 101722-. https://doi.org/10.1016/j.aip.2020.101722

Sugrue et al.	"Everyone thought I was coping as I hid it well, but I just went to rock bottom and ended up drinking and taking an overdose of tablets. (2)" (p. 119 - Results)<br><br>"Another mother spoke in a more positive way about how the death of her child led her to completely reevaluate her life and how she became less upset about things. Likewise, another reported how she had reevaluated her relationship with her remaining children and her role as a mother. She no longer believed that her children would bring their problems to her. One of the mothers used a powerful metaphor: ''It was like if you look through one of those kaleidoscopes and you see a pattern and you think it is beautiful and you do not want to move it, and then it moves and it all changes and that's what happened in my life'' (4)." (pg. 120 - Results)<br><br>"Several participants felt that the death impacted their role as parents to their surviving children. Some described how they were not available to their children because of their continuing pain. For instance, one mother described how she was alerted to her surviving son's pain whilst another was jolted back to the reality that she had other children who needed her support: ''It was not until one of his tears fell on my cheek that I woke up to the fact that my other son was crying every night of the week'' (2); ''One of them said 'Ma what about us, you are putting all your love into him and he is dead. We are here too''' (1). The narrative of ''failure as a mother'' is striking in its direct contrast to that of ''love and forgiveness'' described earlier; the mothers had no difficulty in finding forgiveness for their children and yet appeared to struggle to forgive themselves." (p. 120-121 - Results)<br><br>"Notably, all of the mothers indicated that they had experienced mental health problems following the suicide, including depression, nightmares and insomnia, poor concentration, and numbness or shock. Some also alluded to physical health issues such as heart problems and eating disorders. One mother described her pain as follows: ''I sat looking at a wall for weeks and had to go on medication. It's a hole in my stomach that doesn't go away and it's like someone putting their hand in your body and pulling your heart out'' (p. 121 - Results)<br><br>"A further significant finding from this study was that despite the construction by mothers of positive narratives regarding their lost child, the most common narrative they constructed around themselves was one of ''failure as a mother'' (Theme 3). This may perhaps be best understood in the context of McGoldrick, Anderson, and Walsh's (1991) argument that a child's psychological flaws become the exclusive responsibility of the mother and can lead to mothers carrying blame. This concept is further developed by Forcey (1987) who argued that we are told by social commentators that, behind every violent, mentally ill or merely unfulfilled male, there is a mother. Therefore, although shame was absent from the findings in this study, blame and guilt were clearly not. As noted by Seguin et al. (1995), the current clinical focus on suicide prevention tends to reinforce feelings of guilt and blame in parents who fail to prevent their children from dying by suicide, although Maples (2005) claimed that certain components of suicide risk assessment (e.g., familial factors), potentially contribute to feelings that parents may have failed their children. The guilt expressed by the mothers at having outlived their offspring is also consistent with findings from Farnsworth and Allen (1996), whereas Forcey (1987) stated that the suicide of a child becomes, in the mind of the mother, the ultimate violent failure of mothering. It is not surprising, therefore, to find that ''failure as a mother'' was such a predominant narrative in this study" (p. 122 - Discussion)<br><br>"An additional key finding in the present study was the mothers' abuse of medication and or alcohol to help them cope; alcohol use is not incorporated in many studies as an outcome variable and it is interesting to note that this was spontaneously raised by the mothers here. Where alcohol use in bereavement has been assessed (Vance, Boyle, Najman, & Thearle, 2002), elevated use was demonstrated amongst sudden infant death bereaved fathers but not mothers." (p. 122 - Discussion)	Sugrue, J. L., McGilloway, S., & Keegan, O. (2014). The experiences of mothers bereaved by suicide: an exploratory study. *Death studies* , *38* (1-5), 118–124. https://doi.org/10.1080/07481187.2012.738765
Supiano et al.	"Compassion" was frequently expressed and included both compassion for the deceased and expressions of self-compassion. I feel for all of you (group participants) and love you so much. We loved them all (the deceased) and we did the best we could. I can eat again now, I can finally laugh at my husband's bad jokes—if I can, we all can. (p. 558 - Results)<br><br>For a minority of participants, we did not observe a reduction in expression of distressing physical symptoms. Even as participants made progress in self-care, and even as sleep improved for those previously troubled by intrusive and disturbing dreams, physical distress continued to be endorsed by these participants at end of treatment. (p. 559 - Discussion)	Supiano, K. P., Haynes, L. B., & Pond, V. (2017). The transformation of the meaning of death in complicated grief group therapy for survivors of suicide: A treatment process analysis using the meaning of loss codebook. *Death Studies* , *41* (9), 553–561. https://doi.org/10.1080/07481187.2017.1320339
Tosini & Fraccaro	They generally desired some kind of intimate reconcilement between preserving a positive memory of their loved one and the horror associated with the act of suicide. In those circumstances, certain rituals helped in handling their suffering: The first year was very hard: for the first two months, I continued to see him hanging [referring to her son who died by suicide] […] Then, every day, before going to work, I had to relive the same moment; which means I would go upstairs and pretend to say hallo to him. I had to do this every day, for over a year (Interview, case 1, mother) (p. 989 - Results)<br><br>The grieving process was generally accompanied by efforts to reconcile the perception of inevitable differences between "Us" (survivors) and "Them" (others who have not been through this tragedy) with a new openness toward social life—for example, getting involved in volunteering or other kinds of projects: I continued to commit myself to volunteering, really a lot of time. My life is made for those kinds of things […] and I believe, after living through [this] experience [of the son who died by suicide], that I have a different kind of sensitivity, that I look at things differently, at real life I mean […] It changes you entirely, your values are at another level (Interview, case 11, mother). (p. 991 - Results)<br><br>[In my experience] the group meetings were more useful than psychotherapy. Heavens! I worked there too [i.e., during psychotherapy], but to have so many points of view all together [i.e., among group participants] … yes, you have more hues […] While instead, individually, you have only one head, exactly, and only one life experience, in short. Instead there you are given lots of them […]. There is true emotional closeness […] The group is my space: it allows me to release this thing [i.e., her trauma], to process it, because otherwise I suppress it (Interview, case 2, ex-fiancee). (p. 991 - Results)	Tosini, D., & Fraccaro, D. (2022). "Like climbing a glass wall": Suicide survivors in an Italian province. *Death Studies* , *46* (4), 987–995. https://doi.org/10.1080/07481187.2020.1795746
Turner et al.	Isolation included isolating self from family, friends, and support systems, while avoidance included avoiding emotions, people, or memories of the deceased. This theme occurred in all eight narratives. One participant stated, "I am still not comfortable in large crowds and I've become a lot more withdrawn … I don't have [a support system] and I feel a little bit like my friends have not been there for me, but they don't know how to." Another participant said, "Sometimes, I just want to go to sleep so that I don't feel pain. I don't want to die; I just don't want to feel the pain anymore." Work and using alcohol were identified ways to avoid emotions. One participant wrote in her journal, "I [know] I shouldn't drink but I [can't] help it, especially when I [find] out sad news. I'm too vulnerable." (p. 428 - Results)<br><br>Other participants rejected held religious beliefs and stopped attending worship services. Only one participant voiced a strengthening of spiritual beliefs immediately following the suicide (p. 429 - Results)<br><br>Five of eight participants improved their self-care. One changed therapists and sought psychiatric assistance. Another participant called her personal trainer and began exercising: I am trying to do for myself more and take care of myself. I went and I got a pedicure [and a] massage and the whole thing. I did start walking this week and I am trying so hard to cut down on my intake. (p. 429 - Results)<br><br>Seven of eight participants reported breath therapy helped them let go of negative emotions and assumptions. One reported, The breath session released all these feelings. I am not tormented by [them] anymore. It was his time. And it is nobody's fault. It was just his time and I am at peace with that now. I did what I could. Before I mean I felt so guilty. I just felt like I wanted to die. But I don't feel that now. I don't feel guilty anymore because he died. (p. 429 - 430 - Results)<br><br>Advocacy through giving back and helping others was a theme in seven of eight narratives. One participant voiced a desire to advocate for others by volunteering for the suicide prevention hotline: "I seeing this is preventable and if I could prevent one person, some mother from going through what I am going through." One directly related his decision to become a therapist to his father's suicide as he wanted to help others, and another participant became a volunteer at a bereavement group. Two other participants voiced the need to give back but did not have a specific plan for doing so. (p. 431 - Results)<br><br>An overall theme of reconnection emerged. Two participants expressed a reconnection to church and faith. One stated, I'm intrigued by the spiritual side and it's something that's missing in my life that I don't think I have. And I think that's the thing that I need, and I feel like something has been calling me back to the church. So, I think this experience has made me realize that I have to find it. It's missing. (p. 431 - Results)	Turner, R., Wooten, H. R., & Chou, W.-M. (2019). Changing Suicide Bereavement Narrative Through Integral Breath Therapy. *Journal of Creativity in Mental Health* , *14* (4), 424–435. https://doi.org/10.1080/15401383.2019.1625839
Wardle	As Alex attended more sessions, he continued to use and be guided by the natural setting to connect to his personal experience. He experimented with making art in different ways such as drawing and leaf rubbings. Soon he became more exploratory and playful in sessions. (p. 177) Alex agreed to do this and was able to share his experience more clearly through art, play and talking in sessions after we had been outdoors. (p. 178) His mother shared that she felt the sessions had helped him. She shared that he had less behavioural concerns in school and therefore was able to return to a fuller school day. She also shared that he had started to discuss his family member's death more at home, which had allowed them to do rituals to remember his family member together. (p. 178)	Wardle, A. (2024). Landscape of loss: Art therapy outdoors and traumatic bereavement. *International Journal of Art Therapy, 29* (3), 174–180. https://doi.org/10.1080/17454832.2023.2267109
Watson et al.	Participants also related how helpful it was for them to keep regular routines as they moved forward in the healing process. Justin remembered going back to school: It was a preschool. It was at this lady's house up the street in her basement. She had a classroom. Going back to school must've helped to just normalize things because I felt pretty normal then. (p. 12 - Results)<br><br>I wasn't a good boy for a long time, I strayed and that was primarily because of the pain that I had. I mean, it's a lame excuse but it's, it's, it's really true. Like, if, if you have all that pain, that's the core problem if you don't solve it. You can see all these other actions are made manifest because of that problem. And so, I think a lot of people that have had, had parents or loved ones commit suicide, they primarily start doing drugs, they do all these other things, it's because they're trying to self-medicate to cover up that pain. I did. For a long, long time and it was difficult to get out of. (p. 14 - Results)<br><br>Participants described books as helpful when the book appeared to be gentle; when the book seemed positive; when the book contained bright colorful images and illustrations; when the book helped them to feel connected to a loved one; when the book addressed fears; and when the book acknowledged the challenges of losing a parent to suicide. (p. 16 - Results)	Watson, C., Cutrer-Párraga, E. A., Heath, M., Miller, E. E., Young, T. A., & Wilson, S. (2021). Very Young Child Survivors' Perceptions of Their Father's Suicide: Exploring Bibliotherapy as Postvention Support. *International Journal of Environmental Research and Public Health* , *18* (21), 11384-. https://doi.org/10.3390/ijerph182111384
Williams & Gubi	All participants received therapy at some point following their bereavement. Aaliyah described being able to connect with her sense of self in therapy, "only after years, and being in therapy, I realised that whatever was happening, that it wasn't right." (p. 664) Della had a need for constant physical intimacy. Della's description of feeling trapped and lonely, when physical intimacy broke down in her marriage, could illustrate the extent to which physical intimacy was able to relieve feelings of isolation. Della explained that alcohol and drugs were ways of coping with her grief (p. 669)	Williams, S. A., & Gubi, P. M. (2024). The self-perceived impact of parental suicide in adolescence, of a father, on his daughter's intimate, heterosexual relationships in adulthood. *Illness, Crisis & Loss, 32* (4), 659–676. https://doi.org/10.1177/10541373231186608

Zavrou et al.	Thoughts about their child were constantly on the participants' minds, whether it was morning or evening. However, these were more intense at night-time: "[. . .] I can't sleep at night [. . .] you have all the time this 'why' in your mind." (P5). (p. 7) "I used to hang out with colleagues from work [. . .] Until I heard 'Why are you crying for a drug addict? Isn't it better that he's gone?' [. . .] So, I stopped talking."(P7). (p. 8) Interestingly, the participants described their struggle to construct meaning about their child's suicide in their efforts to seek relief from the pain of loss: "God wanted him (the deceased) [. . .] when I go to church, I feel calm (P5). (p. 8) An additional behavioural coping strategy described was the necessity to be in contact with nature: "I found my way out . . . be in the quiet, in the woods" (P9). The participants described that gardening, going into the woods, as well as going to the shore and even swimming had a therapeutic effect on them, helping them to forget, relax, or find solace: "Working in the garden, with the flowers, makes you forget somehow." (P4). Noteworthily, the participants mentioned that through gardening and contact with the soil they felt that they were nearheir dead child: "I work with the soil in the garden because I feel that I'm close to my son."(P5). Cemetery was also described as the place to be connected to the deceased: 'I go to the cemetery every day. If I don't go once, I think that my son will get sad.' (P10); it also seemed to provide them with a cozy place to externalize emotional burden: My only way out was going to the cemetery and cry" (P3); most of all, it seemed to offer them the therapeutic opportunity to do things for their dead child: "I feel good by doing things over the grave." (P5). (p. 8) Playing or watching mind games, such as chess, also seemed to be effective in keeping the mind away from disturbing thoughts, as described by a male participant: "There is only one thing that I watch and relax [. . .] being focused on chess." (P6). (p. 9) Overall, the participants, very clearly, described their effort to build upon self-motivation in order to support themselves. For instance, a participant used self monitoring techniques to support herself: "[. . .] to see if I was getting better or worse, I was saying out loud my feelings while recording them. After some time, I played the recordings back to see if I got worse, and I felt that in time the sobbing and heartbreak were fading away. And I said to myself that I was ok." (p. 9)	Zavrou, R., Charalambous, A., Papastavrou, E., Koutroubas, A., & Karanikola, M. (2023). Qualitative inquiry into the experience of suicide loss, aftereffects and coping strategies of suicide-bereaved Greek-speaking parents in Cyprus. *International Journal of Qualitative Studies on Health and Well-Being, 18* , 2265671. https://doi.org/10.1080/17482631.2023.2265671
Zuromski et al.	In terms of how to help families and units following a soldier suicide, NOK and SUP suggested using mental health treatment or religious support. Many NOK and SUP stated that they felt supported by the military resources that were in place when they experienced their soldier's suicide (e.g., for NOK, casualty assistance officers) and suggested other families and units utilize those existing resources. (p. 446)	Zuromski, K. L., Wilks, C. R., Al-Suwaidi, M., Wittler, E., Scherban, L., Hite, B., Raymond, L., Dempsey, C. L., Stein, M. B., Ursano, R. J., Benedek, D., & Nock, M. K. (2024). Perspectives of suicide loss survivors: Qualitative analysis of data from a psychological autopsy study of U.S. Army soldiers. *Suicide and Life-Threatening Behavior, 54* (3), 437–449. https://doi.org/10.1111/sltb.13052
