# Supplementary material for: Engagement in meaningful activities post suicide loss: A scoping review
Source: PLoS One. 2025 Nov 17;20(11):e0336640. doi: 10.1371/journal.pone.0336640 (PMC12622850; doi:10.1371/journal.pone.0336640)
Supplement: S4 Appendix — (PDF) [file pone.0336640.s004.pdf]

S4 Appendix

Comprehensive Coding Table for Inductive Content Analysis

Content Category	Description of content category	Sub-category	Description of sub-category	Number of Codes	Codes	Code Frequency
Activity of everyday living (AEL)	Any activity related to self-care, productivity, and leisure “that is performed with some consistency and regularity” (1 p. 19, 2)	Self-care	Includes activities related to taking care of oneself, getting ready and getting around.<br><br>Groupings include: personal care, community management, and general (3)	10	Personal care:<br>- Eating<br>- Sleep<br>- Alcohol and drug use, risky and sexual behaviours<br>Community management:<br>- Driving<br>- Taking the transit<br>General<br>- Meditation<br>- Long-term support groups and professional support<br>- Regular visits to mediums<br>- Religious activities<br>- Self-care (general)	 11<br>24<br>52<br><br>4<br>1<br><br>4<br>96<br>1<br>39<br>7
		Productivity	Includes activities related to earning a living, maintaining a	13	Paid or unpaid work:<br>- Caregiving or parenting	 49

Content Category	Description of content category	Sub-category	Description of sub-category	Number of Codes	Codes	Code Frequency
			home and family, providing service to others, and/or developing one's capabilities.<br><br>Groupings include: paid or unpaid work, household management, and school or play. (3)		- Mental health advocacy activities (general)<br>o Film production<br>o Peer group facilitation<br>o Attending conferences<br>o Joining advocacy groups or organizations<br>o Research participation<br>o Volunteer work (advocacy)<br>- Volunteer work (general)<br>- Work<br>Household management<br>- Housework, chores<br>- Taking on role(s) of family members or a change in family life<br>School or play<br>- School or studies	13<br>1<br>7<br>2<br>6<br>2<br>4<br>3<br>80<br><br>9<br>20<br><br>44
		Leisure	Includes activities an individual	24	Quiet recreation<br>- Arts and crafts<br>- Baking	 8<br>1

Content Category	Description of content category	Sub-category	Description of sub-category	Number of Codes	Codes	Code Frequency
			performs when freed from the obligation to be productive.<br><br>Groupings include: quiet recreation, active reaction, socialization and general. (3)		- Gaming<br>- Music, radio, television<br>- Photography<br>- Reading (to heal)<br>- Reading (general)<br>- Sewing<br>- Writing (journaling, letter writing)<br>Active recreation<br>- Physical activity<br>o Swimming<br>o Tai Chi<br>o Exercise<br>o Walking<br>o Yoga<br>o Dancing<br>- Camping<br>- Gardening<br>- Travelling<br>Socialization<br>- Internet sites and social media<br>- Social activities (general)<br>General<br>- Activities to connect with lost one (hobbies enjoyed by lost one)	1<br>13<br><br>1<br>10<br>2<br>1<br>12<br><br><br>2<br>2<br>1<br>4<br>8<br>6<br>1<br>1<br>10<br>3<br><br>28<br><br>31<br><br><br>2

Content Category	Description of content category	Sub-category	Description of sub-category	Number of Codes	Codes	Code Frequency
					- Enjoyed activity, hobbies (general)<br>- Shopping	3<br><br>1
		Generic or unspecified	Includes activities that did not fall into the above categories or were generic or non-specific when referenced (e.g., daily activities)	3	Activities related to creation of a new life<br><br>Everyday activities (general)<br><br>Routines and rituals related to the lost one	2<br><br>15<br><br>30
Engagement status	The status of an individual's participation in an activity, specifically stating how occupied or involved an individual is, potentially describing change in activity as well (2)	Change in frequency or method of engagement (abbreviated for coding purposes to: change in engagement)	An altering or modification of an activity through adaption, restructuring, reconstruction, or a change in the frequency of engagement in an activity (2,4)	31	Change in engagement (alcohol and drug use, risky and/or sexual behaviour)<br><br>Change in engagement (caregiving and parenting)<br><br>Change in engagement (dancing)<br><br>Change in engagement (driving)<br><br>Change in engagement (eating)<br><br>Change in engagement (everyday activities)	45<br><br>39<br><br>1<br><br>3<br><br>9<br><br>5

Content Category	Description of content category	Sub-category	Description of sub-category	Number of Codes	Codes	Code Frequency
					Change in engagement (exercise)	2
					Change in engagement (gaming)	1
					Change in engagement (gardening)	3
					Change in engagement (housework and chores)	2
					Change in engagement (internet sites and social media)	7
					Change in engagement (long-term support groups and profession support)	4
					Change in engagement (music, radio, TV)	6
					Change in engagement (photography)	1
					Change in engagement (physical activity)	1
					Change in engagement (reading – general)	1

Content Category	Description of content category	Sub-category	Description of sub-category	Number of Codes	Codes	Code Frequency
					Change in engagement (reading – to heal)	9
					Change in engagement (religious activities)	21
					Change in engagement (routines and rituals related to lost one)	2
					Change in engagement (school or studies)	22
					Change in engagement (self-care general)	2
					Change in engagement (shopping)	1
					Change in engagement (sleep)	24
					Change in engagement (social activities)	1
					Change in engagement (swimming)	1

Content Category	Description of content category	Sub-category	Description of sub-category	Number of Codes	Codes	Code Frequency
					Change in engagement (taking on roles of family members, change in family life)	16
					Change in engagement (taking the transit)	1
					Change in engagement (travelling)	1
					Change in engagement (walking)	3
					Change in engagement (work)	34
					Change in engagement (yoga)	2
		Continued engagement	Continued involvement and participation in an activity (2,4)	12	Continued engagement (alcohol, drug use, risky and/or sexual behaviour)	2
					Continued engagement (arts and crafts)	1
					Continued engagement (caregiving and parenting)	3
					Continued engagement (gardening)	3

Content Category	Description of content category	Sub-category	Description of sub-category	Number of Codes	Codes	Code Frequency
					Continued engagement (housework, chores)	1
					Continued engagement (long term support groups and professional support)	1
					Continued engagement (music, radio, TV)	6
					Continued engagement (religious activities)	7
					Continued engagement (routines and rituals related to lost one)	1
					Continued engagement (school or studies)	2
					Continued engagement (social activities)	2
					Continued engagement (work)	9
		Disengagement	Abandoning or no longer involving oneself in / participating in an activity (2,4)	17	Disengagement (alcohol and drug use, risk and/or sexual behaviour)	3
					Disengagement (baking)	1

Content Category	Description of content category	Sub-category	Description of sub-category	Number of Codes	Codes	Code Frequency
					Disengagement (caregiving and parenting)	3
					Disengagement (eating)	1
					Disengagement (everyday activities)	7
					Disengagement (housework, chores)	3
					Disengagement (internet sites and social media)	1
					Disengagement (long term support groups and professional support)	3
					Disengagement (physical activity)	1
					Disengagement (reading – general)	1
					Disengagement (religious activities)	1
					Disengagement (school or studies)	12

Content Category	Description of content category	Sub-category	Description of sub-category	Number of Codes	Codes	Code Frequency
					Disengagement (self-care – general)	1
					Disengagement (social activities)	18
					Disengagement (tai chi)	1
					Disengagement (taking on roles of family members, change in family life)	1
					Disengagement (work)	21
		Re-engagement	Resuming involvement and participation within an activity previously engaged in (2)	22	Re-engagement (activities to connect with lost one – hobbies enjoyed by lost one)	1
					Re-engagement (alcohol and drug use, risk and/or sexual behaviour)	1
					Re-engagement (arts and crafts)	1
					Re-engagement (camping)	1
					Re-engagement (driving)	1
					Re-engagement (eating)	1

Content Category	Description of content category	Sub-category	Description of sub-category	Number of Codes	Codes	Code Frequency
					Re-engagement (enjoyed activity, hobbies - general)	1
					Re-engagement (everyday activities – general)	3
					Re-engagement (exercise)	1
					Re-engagement (housework, chores)	1
					Re-engagement (long term support groups and professional support)	2
					Re-engagement (religious activities)	1
					Re-engagement (school or studies)	2
					Re-engagement (self-care – general)	2
					Re-engagement (sewing)	1
					Re-engagement (social activities)	2
					Re-engagement (swimming)	1

**Content Category**	**Description of content category**	**Sub-category**	**Description of sub-category**	**Number of Codes**	**Codes**	**Code Frequency**
					Re-engagement (taking on roles of family members, change in family life)	1
					Re-engagement (travelling)	1
					Re-engagement (volunteer work – general)	2
					Re-engagement (walking)	2
					Re-engagement (work)	7
		Engagement in a novel activity	Involvement and participation within a novel activity (2,4)	33	New engagement (activities related to creation of a new life)	2
					New engagement (activities to connect with lost one – hobbies enjoyed by lost one)	1
					New engagement (alcohol and drug use, risky and/or sexual behaviour)	1
					New engagement (arts and crafts)	6
					New engagement (attending conferences)	2

**Content Category**	**Description of content category**	**Sub-category**	**Description of sub-category**	**Number of Codes**	**Codes**	**Code Frequency**
					New engagement (caregiving and parenting)	5
					New engagement (enjoyed activities, hobbies - general)	2
					New engagement (exercise)	2
					New engagement (film production)	1
					New engagement (gardening)	4
					New engagement (housework and chores)	2
					New engagement (internet sites and social media)	21
					New engagement (joining advocacy groups or organizations)	5
					New engagement (long-term support groups and profession support)	88
					New engagement (meditation)	4

Content Category	Description of content category	Sub-category	Description of sub-category	Number of Codes	Codes	Code Frequency
					New engagement (mental health or suicide loss advocacy activities)	13
					New engagement (music, radio, TV)	1
					New engagement (peer group facilitation)	7
					New engagement (regular visits to mediums)	1
					New engagement (religious activities)	9
					New engagement (research participation)	2
					New engagement (routines and rituals related to lost one)	28
					New engagement (school or studies)	6
					New engagement (self-care general)	2
					New engagement (social activities)	8

Content Category	Description of content category	Sub-category	Description of sub-category	Number of Codes	Codes	Code Frequency
					New engagement (taking on roles of family members, change in family life)<br><br>New engagement (travelling)<br><br>New engagement (volunteer work – advocacy)<br><br>New engagement (volunteer work – general)<br><br>New engagement (walking)<br><br>New engagement (work)<br><br>New engagement (writing)<br><br>New engagement (yoga)	3<br><br>1<br><br>4<br><br>1<br><br>3<br><br>13<br><br>12<br><br>4
Associated meanings	Referring to the meaning an individual is generating / generated or associating / associated with engaging in an activity (can be linked to any	Surviving loss	Meaning making or meaning made to survive and/or endure the bereavement and grief associated with suicide loss (5).	6	Searching for answers<br><br>Surviving grief (activities to drown, distract, avoid, survive)<br><br>Obligations<br><br>Addressing stigma	14<br><br>100<br><br>8<br><br>6

Content Category	Description of content category	Sub-category	Description of sub-category	Number of Codes	Codes	Code Frequency
	engagement status listed above).				Fear of dying by suicide<br><br>Intrusion of grief (grief taking over, unintentional)	1<br><br>75
	Meaning can be seen as both a process (meaning making) or an outcome (meaning made). Both types of meaning were considered during coding(5)	Managing and processing loss	Meaning making or meaning made to manage and process the bereavement and grief associated with suicide loss (5).	11	Managing and processing grief<br><br>Creation of normalcy and routine<br><br>Taking care of self<br><br>The need to believe in something bigger (higher power, ultimate plan)<br><br>Realizing death<br><br>Re-evaluating personal capabilities<br><br>Re-evaluation of roles<br><br>Protect and care for surviving loved ones<br><br>To foster connection with others	75<br><br>11<br><br>7<br><br>14<br><br>2<br><br>18<br><br>12<br><br>35<br><br>42

**Content Category**	**Description of content category**	**Sub-category**	**Description of sub-category**	**Number of Codes**	**Codes**	**Code Frequency**
					Openly share loss experience (without fear of hurting others)<br><br>Realizing the importance of the lost one after death	7<br><br>2
		Moving forward	Meaning making or meaning made to move forward after suicide loss (5).	7	Moving forward through enjoyed activities<br><br>Want to learn, help and advocate about MH and suicide loss<br><br>Constructive use of experience<br><br>Regaining life despite loss<br><br>Suicide loss and self-identity<br><br>Reflecting on beliefs, values and goals or priorities<br><br>Keeping the memory alive and protecting connection to lost one	4<br><br>38<br><br>13<br><br>3<br><br>4<br><br>21<br><br>48

4
