# Supplementary material for: Engagement in meaningful activities post suicide loss: A scoping review
Source: PLoS One. 2025 Nov 17;20(11):e0336640. doi: 10.1371/journal.pone.0336640 (PMC12622850; doi:10.1371/journal.pone.0336640)
Supplement: Attachment — Submitted filename: Response to Reviewers.pdf [file pone.0336640.s006.pdf]

Dear Dr. Sanja Batić Očovaj, Academic Editor,

Thank you for the review of our manuscript, entitled "Engagement in meaningful activities post suicide loss: A scoping review" for publication in PLOS One and for all the helpful comments. We are pleased to submit an updated manuscript with tracked changes.

Below, please find responses to each of the editor's / reviewers' comments and our response.

1) Editor Comments and Responses

Issue raised: Please ensure that your manuscript meets PLOS ONE's style requirements, including those for file naming.
Response: The formatting of the manuscript has been changed throughout to meet PLOS ONE style requirements.
Specific changes: Changes to formatting of the manuscript: title page, abstract, headings, titles of table/figures, and body. Changes to supplementary information document titles.
Page #: 1-47, S1 Appendix, S2 Appendix, S3 Appendix, S4 Appendix

Issue raised: We noticed you have some minor occurrence of overlapping text with the following previous publication(s), which needs to be addressed:
https://pmc.ncbi.nlm.nih.gov/articles/PMC9408753/?
In your revision ensure you cite all your sources (including your own works), and quote or rephrase any duplicated text outside the methods section. Further consideration is dependent on these concerns being addressed.
Response: Thank you for highlighting this in our manuscript, although any overlap was unintentional, we cross-referenced Kaspersen and colleagues (2022) with our own work and noted some similarities in sentence structure due to the use of a similar and very structured methodology (scoping review using JBI framework), as well as some conceptual similarities due to the small literature base on suicide bereavement (e.g., using similar references, common definitions and statistics). Changes have been made to the abstract, introduction and methods to address these. This paper is referenced in the introduction of this paper. In addition, the protocol paper (Gill et al., 2024) published related to this scoping review was revisited and compared side-by-side. Changes were made within the abstract and introduction, as well it has been cited more thoroughly throughout to assist in avoiding any potential self-plagiarism.
Specific changes related to Kaspersen and colleagues (2022) paper titled **"***Use of Health Services and Support Resources by Immediate Family Members Bereaved by Suicide: A Scoping Review"***:**

1. Abstract: "Globally, more than 720,000 people die by suicide each year, leaving grieving individuals in their wake. Research indicates that individuals who lose a loved one to suicide face heightened risks for negative health outcomes."
   "While references to meaningful activities of everyday living appear in the bereavement literature, they typically are discussed within the background rather than central research aims."
2. Introduction: "Globally, more than 720,000 deaths occur due to suicide every year leaving families, friends, and communities to cope with this sudden loss [1]."

"Within the bereavement literature, the term "survivors of suicide loss" is often used to describe those who are bereaving a suicide; therefore, this review will remain consistent with this terminology [9]"

3. Methods: "A scoping review was completed in accordance with the Joanna Briggs Institute (JBI) guidance [26]."

Page #: Abstract: p. 2-3; Intro: p. 3; Methods: p. 7

Specific changes related to Gill and colleagues (2024) paper titled "*Engagement in meaningful activities post suicide loss: A scoping review protocol*":

1. Abstract: "Recent studies show that taking part in meaningful activities can help protect health emphasizing the importance of exploring engagement in meaningful activities of everyday living among those bereaved. Currently, there has not been a review of the bereavement literature exploring the nature of, and extent to which, meaningful activities of everyday living are discussed."
2. Introduction: The introduction has been rewritten. Please see document.
3. Methods: References made and added to credit original published protocol.

Page #: Abstract: p. 2-3, Intro: p. 3-6, Methods: p. 7-8

Issue raised: Please note that funding information should not appear in any section or other areas of your manuscript. We will only publish funding information present in the Funding Statement section of the online submission form. Please remove any funding-related text from the manuscript.

Response: All funding information has been removed from the manuscript.

Specific change: Deleted text - "Funding Source. M. Gill is a PhD Candidate at the University of Toronto, and was supported by the Ontario Graduate Scholarship, Toronto Rehabilitation Institute Student Scholarship, Theresa and Miron Polatajko Graduate Award, Dalton Whitebread Scholarship Fund, the Dawson Family Scholarship, and the Peter Rappolt Family Scholarship for Research in Occupational Performance and Wellbeing in relation to this work. The funders did not have a role in study conceptualization, design, data collection, analysis, decision to publish, or preparation of the manuscript."

Page #: 33

Issue raised: We note that your Data Availability Statement is currently as follows: All relevant data are within the manuscript and in Supporting Information files.

Please confirm at this time whether or not your submission contains all raw data required to replicate the results of your study. Authors must share the "minimal data set" for their submission. PLOS defines the minimal data set to consist of the data required to replicate all study findings reported in the article, as well as related metadata and methods (https://journals.plos.org/plosone/s/data-availability#loc-minimal-data-set-definition).

For example, authors should submit the following data:

- The values behind the means, standard deviations and other measures reported;
- The values used to build graphs;
- The points extracted from images for analysis.

Authors do not need to submit their entire data set if only a portion of the data was used in the reported study.

If your submission does not contain these data, please either upload them as Supporting Information files or deposit them to a stable, public repository and provide us with the relevant URLs, DOIs, or accession numbers. For a list of recommended repositories, please see https://journals.plos.org/plosone/s/recommended-repositories.
If there are ethical or legal restrictions on sharing a de-identified data set, please explain them in detail (e.g., data contain potentially sensitive information, data are owned by a third-party organization, etc.) and who has imposed them (e.g., an ethics committee). Please also provide contact information for a data access committee, ethics committee, or other institutional body to which data requests may be sent. If data are owned by a third party, please indicate how others may request data access.
Response: Confirming that, at this time, this submission contains all raw data required to replicate the results of this study.
Specific change: Addition of gender data to S2 Appendix - Data Extraction Table. Additional S3 Appendix – Data Extraction Table: Direct Quotes for Inductive Content Analysis added to Supplemental Information, previous S3 Appendix C relabelled to S4 Appendix.
Page #: S2 Appendix, S3 Appendix and S4 Appendix

Issue raised: The reviewers gave you suggestions that would improve your work. The introduction should give more information about MActEL, but also about the occupation theories that you mentioned later in the text.
Response: Thank you for this summary comment of reviewer feedback. This has been addressed in the introduction through the addition of three paragraphs discussing the occupational perspective, MActEL and occupational models / frameworks.
Specific change:

"The documented health consequences and the unique challenges post suicide loss, contribute to difficulty adjusting to life [15,17]. This difficulty adjusting is often called complicated grief, which has been shown to lead to challenges in engagement in meaningful activities of everyday living (MActEL) [18]. However, such challenges and difficulty engaging in MActEL can also occur after a suicide loss even without a diagnosis of complicated grief [19]. Currently, there is a need for further exploration on how MActEL are discussed within the suicide bereavement literature.

This scoping review was completed from an occupational perspective, defined as "a way of looking at or thinking about human doing" [20. p. 233]. This perspective is rooted in the occupational science and occupational therapy field where MActEL are also referred to as "occupations" [21]. MActEL or occupation is defined as any activity related to self-care, productivity, or leisure "that is performed with some consistency and regularity, that brings structure, and is given value and meaning by individuals and a culture" [22, p. 19]. The categorization of MActEL into self-care, productivity and leisure stems from the Canadian Occupational Performance Measure (COPM), an outcome measure designed to identify and evaluate an individual's self-perceived performance in MActEL [23]. The COPM is based on the Canadian Model of Occupational Performance and Engagement (CMOP-E), which provides the theoretical foundation for the COPM highlighting the interaction between the person, environment, and occupation [22,23].

Building on this perspective, Connor Schisler and Polatajko's work within occupational science speaks to the dynamic nature of engagement in MActEL as influenced by environmental factors while being mediated by the person [24]. They highlight that MActEL can be newly

adopted, changed, abandoned, or remain constant in response to environmental and personal factors [24]. Thus, using an occupational perspective to explore the suicide bereavement literature and MActEL is in line with the shifting need identified in the field, specifically moving beyond biomedical and clinical perspectives, towards a focus on broader contextual factors to understand suicide and suicide bereavement.

The word "meaningful" is used intentionally in relation to activity, as a central assumption in occupational science and occupational therapy is that all occupations are inherently meaningful, with meaning assigned by an individual or culture [22]. Other fields have also explored the concept of meaning making. In psychology, for example, Neimeyer has extensively studied meaning making in his meaning reconstruction model, which proposes that reconstruction of meaning is essential following loss [25,26]. He describes meaning reconstruction as "a central process in grieving… the attempt to reaffirm or reconstruct a world of meaning that has been challenged by loss" [27]. As mentioned above, within this scoping review, MActEL refers to activities to which meaning is assigned by an individual or culture [22]. However, it is important to recognize that following loss, these meanings may shift or be reconstructed, as outlined in Neimeyer's model [25,26]. This idea aligns with the occupational science and occupational therapy models and frameworks adopted in this work (e.g., Connor Schisler and Polatajko's work on the dynamic nature of engagement) [22–24].

Bhullar, Sanford and Maple, as well as Miklin and colleagues emphasized the importance of focusing on meaning making and perceived individual impact following a suicide loss or suicide death exposure [28,29]. Previous research supports engagement in MActEL as an avenue to facilitating meaning in life, fulfilling basic psychological needs. and supporting physical and mental health recovery through fostering hope, identity, and connectedness [30–32]. Together, these findings further highlight the need for a literature review emphasizing the value of synthesizing and mapping the current suicide bereavement literature as it relates to MActEL."
Page #: 4-6

Issue raised: The discussion could be enriched with an in-depth analysis and comments on the results from the perspective of meaning-making. The existentialist framework can be applied in the interpretation of data.
Response: Thank you for this great suggestion. Although this work stems from the occupational perspective from the occupational science and occupational therapy literature, a paragraph was added to speak to how suicide bereavement literature is rooted in the field of psychology and thus meaning making is often discussed in line with tenets of existentialism.
Specific change: "This understanding for the need of meaning making following the suicide loss experience stems from the research that highlights meaning making or meaning reconstruction as an essential step in moving through loss, specifically in existentialist frameworks within psychology and, thus, echoed within the bereavement literature [25–27,75,181]. Much of the suicide bereavement literature pulls from tenets of existentialism. Existentialism encompasses many perspectives but is often understood as the pursuit of living one's most authentic life while facing universal human challenges [182]. Within this framework, scholar Yalom identified four "givens" or ultimate challenges including death, freedom, isolation, and meaninglessness, which shape the human experience [181]. Building from this, much of suicide bereavement literature has focused on meaning making and meaning reconstruction as an essential step in processing grief and living with loss [25,178–180]."
Page #: 27-28

Issue raised: In the sentence "Studies used qualitative (80%), quantitative (9%) and mixed (11%) methods." use frequency instead of percentages since the number of studies is small.
Response: This has been edited to reflect frequencies rather than percentages.
Specific change: "Studies used qualitative (n=90), quantitative (n=10) and mixed (n=12) methods."
Page #: p. 2

Issue raised: Check the required format of the table for Plos One and increase the quality of figures.
Response: As per comment above, the formatting of the manuscript has been changed throughout to meet PLOS ONE style requirements.
Specific changes: Changes to formatting of the manuscript: title page, abstract, headings, titles of table/figures, and body. Changes to supplementary information document titles. All figures were run through PACE and NAAS software to ensure compatibility and figure titles removed. Quality was improved of all images.
Page #: 1-47, S1 Appendix, S2 Appendix, S3 Appendix, S4 Appendix, Fig 1-4

2) Reviewer 1 Comments and Responses

Issue raised: The introduction to MActEL was a bit scattered. Please rewrite it to make the introduction more fluent.
Response: The introduction, in its entirety, was rewritten to improve flow and better introduce MActEL.
Specific change: See introduction in manuscript.
Page #: 3-6

Issue raised: You acknowledge that most included studies are from high-income countries, but there is limited discussion on cultural factors in shaping MActEL post-suicide loss. Consider discussing how sociocultural, gender, and systemic inequities influence engagement in meaningful activities during bereavement.
Response: Thank you for this comment, as it is an important consideration. We have added a paragraph to address this.
Specific change: "In addition to the need to focus on the reciprocal relationship between the suicide loss experience and engagement in MActEL, further research is needed to explore the socio-cultural, systemic, and individual (e.g., gender, socioeconomic status, etc.) contextual factors that shape this engagement. Much of the literature included in this review originates from high-income, Western contexts, where individualized coping and work-related re-engagement are often emphasized [193,194]. In contrast, in collectivist cultures, such as those in parts of Asia and South America, family obligations, rituals, and stigma may act as barriers or structured opportunities for MActEL following loss [143]. From a systemic perspective, concerns such as the criminalization of suicide and the consequential stigma, as well as the lack of public health measures addressing contributing factors can also influence whether and how bereaved individuals re-engage in MActEL [195,196]. Suicide remains criminalized in more than 20 countries worldwide, disproportionately affecting people in low- and middle-income contexts

where stigma and fear of legal repercussions limit access to support [196,197]. Limited availability of culturally safe, affordable resources and uneven distribution of mental health services further compound these inequities [198]. At the individual level, gendered expectations, socio-economic status, inequities, among other factors may shape MActEL engaged in post suicide loss. Ultimately, these dynamics highlight the need for future research to capture more diverse cultural perspectives and to examine how intersecting identities influence engagement in MActEL following suicide loss globally."
Page #: 29-30

Issue raised: You note that their secondary question on barriers/facilitators could not be fully answered. While this is transparent, consider suggesting ways future studies could explicitly capture barriers/facilitators in relation to MActEL.
Response: Thank you for this comment. This has been added to the manuscript.
Specific change: "Future research could identify potential facilitators and barriers to engagement in MActEL by exploring the components of this reciprocal relationship in further detail, specifically studying engagement status, AEL and associated meaning as separate components." **Page #:** 30

Issue raised: Figure are in low quality.
Response: Thank you for noting this. All figures have been reassessed for quality and ran through PACE and NAAS software to ensure compatibility.
Specific change: See Fig 1-4
Page #: Fig 1-4

3) Reviewer 2 Comments and Responses

Issue raised: The affiliations of two authors are missing: Shania Pierre and Larine Joachim.
Response: Apologies for this oversight. The affiliations of both authors have been added to the title page.
Specific change: See title page for affiliations.
Page #: 1

Issue raised: The introduction does not sufficiently justify this scoping review of the published literature from the perspective of meaning-making after loss and the role of activities of daily living in that construction. I understand that it is essential to establish the scope of the activities of daily living of survivors of suicide loss as a destroyed life space that must be redefined.
In this sense, I believe that this perspective of meaning-making should be included more explicitly in the discussion.
Response: Thank you for this comment. We have rewritten the introduction in its entirety and specifically discussed the notion of meaning making in relation to MActEL. We hope the revised introduction justifies the need for a scoping review in this area. We have also related it back to the occupational science and occupational therapy literature as this review was written from an occupational perspective.
Specific change: See introduction in manuscript.
Page #: 3-6

Issue raised: In Table 2, you should complete the matrix with the "n" and "%" for each element to have all the information in the table.
Response: Unfortunately, we cannot do this. This table was constructed using data coded during the inductive content analysis. Each category (n=3) consisted of subcategories (n=11) and many separate codes (n=6-33). This would involve pulling each code (n=6-33) for each subcategory (n=11) and determining the frequency of the code as it overlaps with other codes in different categories. For reference, code frequencies ranged from 1-100. However, to enhance transparency, we did provide the following supplementary information including: 1) S4 Appendix includes the individual code frequencies and their relation to the larger subcategories and categories; 2) S3 Appendix includes direct quotes extracted from each article; and 3) S2 Appendix includes the categories and subcategories that were coded for each separate article.
Specific change: No change made.
Page #: NA

Issue raised: In Figure 4, the model represented is not sufficiently explained in the text. What is specifically expressed by the intersection of the circles? What meaning do the bidirectional arrows have? Is everything connected and related to everything else?
Response: Thank you for this comment and for identifying this omission. The thematic map has been described in more detail in the manuscript.
Specific change: "In addition, content categories with their respective subcategories are mapped in Fig 4 [66]. This map is a visual representation of the categories presented above. Within this thematic map, AEL, engagement status and associated meaning are overlapping categories that make up the components of the discussion surrounding MActEL within the suicide bereavement literature. Each category is connected to the others with a bidirectional arrow, representing the reciprocal influence they have on each other. The subcategories within each category are listed as well."
Page #: 25-26

Issue raised: Conclusions: The last conclusion lacks precision and concreteness. It seems more like a summary than conclusions based on the results.
Response: We have revised the last sentence in our conclusion to convey precision and concreteness.
Specific change: "This review identifies a need for focused research that brings the discussion on the engagement in MActEL following suicide loss and its implications on the suicide bereavement process into the foreground."
Page #: 31

Thank you again to the editor and both reviewers for their commitment in reviewing this manuscript. We hope that the updated manuscript is seen as an improvement and that the responses above are clarifying.

All authors continue to declare no conflict of interest with this research study or publication.

Thank you for your consideration.

Respectfully submitted,

Monique Gill, PhD Candidate, OT Reg. (Ont.), MScOT, BHSc
PhD Candidate, Rehabilitation Sciences Institute
University of Toronto
Email: moniquekaur.gill@mail.utoronto.ca
